# Thiol-Free Sulfenylation Redefined: A Single-Atom Transfer Pathway to Symmetrical Di(hetero)arylthioethers via B(C_6_F_5_)_3_ Catalysis

**DOI:** 10.1021/jacs.5c17932

**Published:** 2026-01-29

**Authors:** Milan Pramanik, Nusaybah Alotaibi, Tribani Boruah, Niklaas J. Buurma, Rasool Babaahmadi, Thomas Wirth, Rebecca L. Melen

**Affiliations:** † Cardiff Catalysis Institute, School of Chemistry, 2112 Cardiff University, Translational Research Hub, Maindy Road, Cathays, Cardiff CF24 4HQ, Cymru/Wales U.K.; ‡ Department of Chemistry, King Faisal University, College of Science, P.O. Box 400, Al Ahsa 31982, Saudi Arabia; § School of Chemistry, Cardiff University, Main Building, Park Place, Cardiff CF10 3AT, Cymru/Wales U.K.

## Abstract

Diarylated thioethers are privileged scaffolds found across pharmaceuticals, functional materials, and molecular electronics. Conventional approaches to these motifs, typically via C–H functionalization or C–X cross-coupling with thiophenols, disulfides, thiosulfonates, and related sulfenylating agents, remain hampered by foul odors, instability, air- and moisture- sensitivity, tedious synthesis, and poor selectivity, often producing undesired byproducts. In contrast, the few strategies that employ elemental sulfur for symmetrical thioether synthesis are largely confined to copper catalysis and lack generality. Therefore, a straightforward and sustainable route to symmetrical thioethers from readily accessible, bench-stable sulfenylating agents with broad substrate compatibility is highly desirable. Herein, we demonstrate *N*,*N*′-thiobisphthalimide as a bench-stable sulfenylating reagent enabling the synthesis of symmetrical diaryl/diheteroaryl thioethers or dibenzothiophenes in yields up to 97%. This transformation precedes via a single-atom transfer (SAT) strategy under metal-free B­(C_6_F_5_)_3_ catalysis with electron-rich arene and heteroarene substrates. Mechanistic investigations, supported by DFT calculations, cyclic voltammetry (CV), and UV–vis. studies, reveal a stepwise ionic pathway and rationalize the observed regioselectivity and substrate-dependent reactivity. Beyond synthetic value, the ambipolar redox behavior of the resulting thioethers establishes them as tunable photoredox mediators, bridging small-molecule synthesis and functional material design.

## Graphical abstract

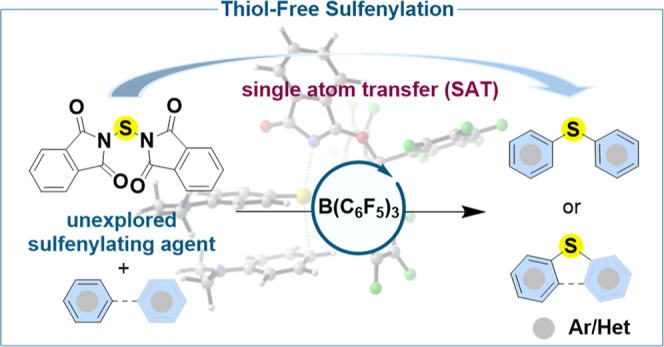

## Introduction

Thioether skeletons have appeared as cornerstone structural motifs in the design of drugs, pharmaceuticals, and materials.^1–3^ The C–S–C linkage within diaryl thioether compounds improves lipophilicity, enhancing pharmacokinetic properties such as membrane permeability, which is highly desirable in drug development.^4^ For instance, thioether-containing compounds, as indicated in Figure 1A, showcase the versatile biological and pharmaceutical activities.^5^ Beyond pharmaceuticals, thioether linkages are also pivotal in materials science, as evidenced in organic semiconductors,^6,7^ and play a crucial role in agriculture.^8^ Traditional sulfur sources for synthesizing the thioether core, however, continue to present significant limitations (Figure 1B). Thiophenols, while common, suffer from a strong odor, toxicity, and a tendency toward oxidative degradation.^9,10^ Disulfides synthesized from thiols often undergo overoxidation, restricting their applications due to the formation of undesired side products.^11,12^ Thiosulfonates derived from thiols are unstable under basic or nucleophilic environments, leading to poor selectivity.^13–15^ Similarly, *N*-arylthiosuccinimides, though more versatile, demand prefunctionalization and inevitably produce succinimide as sacrificial waste, thus compromising atom economy.^16^ Although reactive, aryl sulfonyl chlorides, are highly moisture-sensitive and corrosive, resulting in safety concerns, especially at scale.^17–19^ Alternatively, aryl sulfonyl hydrazines are prone to thermal decomposition and generate gaseous byproducts, complicating purification and handling.^20^ Commercially available potassium xanthogenate offers only a partial solution, as its use in thioetherification requires metal/ligand activation, liberates foul-smelling thiols in situ, and unavoidably generates byproducts.^21^ Inorganic elemental sulfur has likewise been utilized for the installation of sulfur into organic frameworks; nevertheless, such approaches use copper catalysts and are further plagued by poor solubility issues.^22,23^ Collectively, these persistent shortcomings underscore the need for a bench-stable, thiol-free sulfenylating reagent with a broad scope, high selectivity, and sustainable reactivity. In this context, considerable attention has been paid by synthetic chemists to discover refined synthetic routes for diaryl thioether synthesis over the years.^24^ While unsymmetrical diaryl thioethers have been widely accessed via metal catalysis, stoichiometric iodine/peroxide reagents, photoredox and electrochemical mediated C–H functionalization or C–X cross-coupling strategies (Figure 1C),^25–35^ the efficient synthesis of symmetrical diaryl thioethers remains largely elusive. The challenges in advancing efficient synthetic routes for symmetrical thioethers using inexpensive, bench-stable and readily available sulfenylating agents highlights a compelling gap in the sulfur skeleton landscape, offering an opportunity for the expansion of novel synthetic strategies.

**Figure 1:**
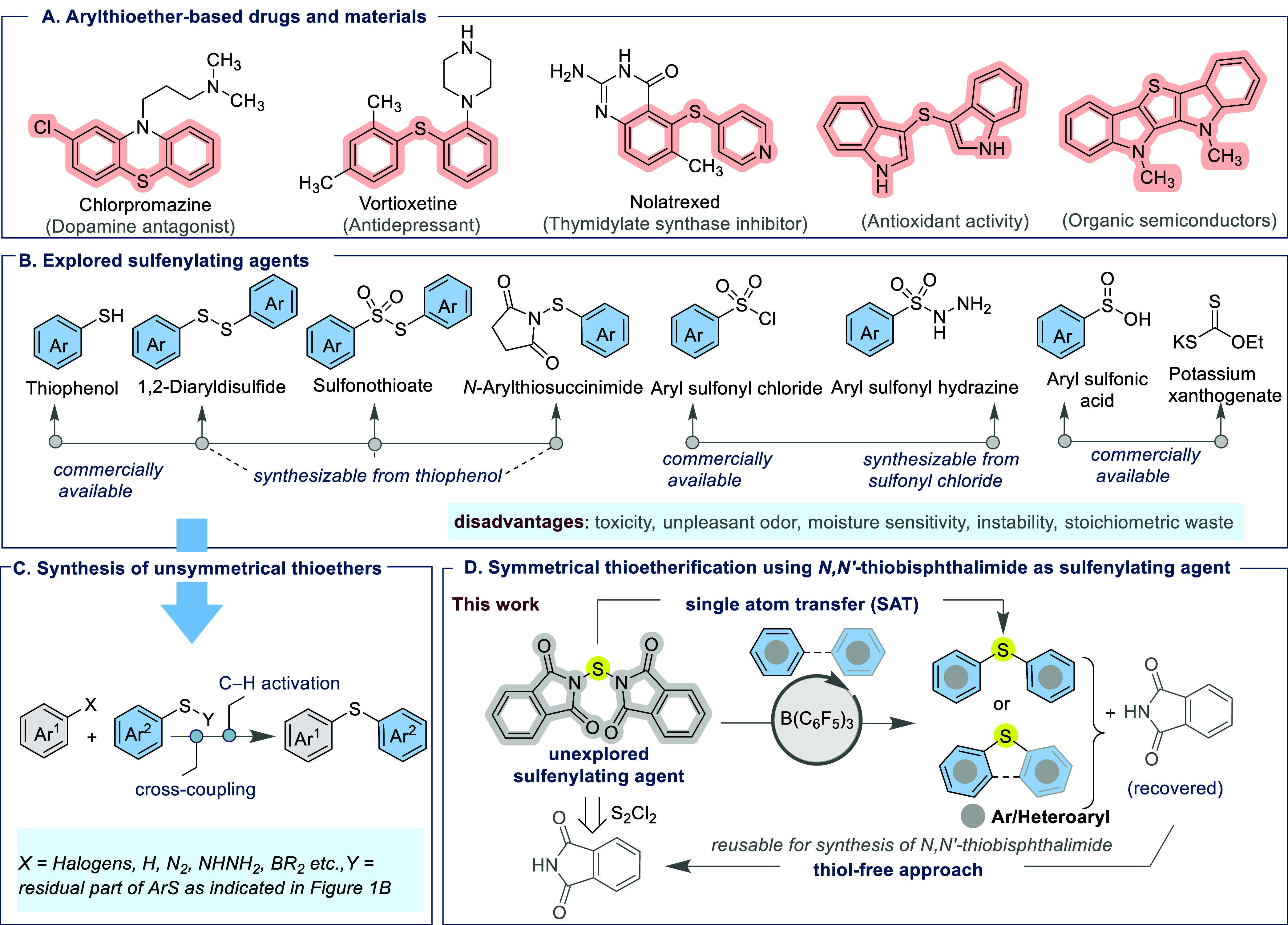
(A) Representative thioether motifs commonly found in pharmaceuticals and materials science. (B) Established sulfur-based precursors employed as sulfenylating agents. (C) Conventional synthetic strategies toward unsymmetrical diaryl thioethers. (D) This work: introducing *N*,*N*′-thiobisphthalimide as a thiol-free, bench stable sulfenylating agent for the efficient B­(C_6_F_5_)_3_-catalyzed synthesis of symmetrical thioethers.

Atom transfer reaction strategies have emerged as powerful tools in modern synthetic chemistry, enabling the precise and selective delivery of specific atoms under controlled and unified conditions. N-Heterocyclic carbene (NHC) compounds are predominantly used as “C” synthons, effectively enabling carbon atom transfer to various organic feedstocks.^36^ In particular, atom transfer or group transfer reaction strategies have recently emerged as a compelling area of research for the skeletal editing of organic molecules.^37–40^ In this context, the sulfur atom transfer strategy remains virtually unexplored, yet clutches the potential to tackle key challenges in selectivity, waste minimization, and functional group tolerance, thereby paving the way toward sustainable and efficient construction of sulfur-rich molecular architectures. In this work, we disclose that *N,N′*-thiobisphthalimide, an air-stable, odorless, and thiol-free sulfenylating reagent,^41^ can facilitate single sulfur atom transfer reaction to synthesize symmetrical thioethers using borane catalysis (Figure 1D). Beyond small-scale laboratory implementation, avoidance of foul-smelling sulfur-based reagents is highly substantial for large-scale applications, where volatility and foul smell of commonly used sulfur reagents present inherent disadvantages, including potential health hazards. These issues pose challenges related to safety, ventilation supplies, and regulatory compliance. Thus, the use of odorless, bench-stable, environmentally friendly, benign sulfur sources in our present method, therefore, improves its practicality and scalability for industrial implementation and large scale thiother synthesis.

The reactivity of tris­(pentafluorophenyl)­borane [B­(C_6_F_5_)_3_], a quintessential electron-deficient Lewis acid, is well-recognized for promoting heterolytic bond cleavage through the formation of dative interactions with Lewis basic donor atoms.^42,43^ In this context, it has emerged as a powerful platform within metal-free catalysis, underpinned by its favorable attributes such as earth abundance, low toxicity, and the capacity to enable complementary or superior selectivity profiles.^44–49^ Our group,^50–52^ along with others, have extensively employed B­(C_6_F_5_)_3_ as a robust catalyst, and as the Lewis acidic partner in frustrated Lewis pair (FLP) frameworks to mediate a broad array of C–C and C–X bond-forming transformations.^53–55^ Nevertheless, despite its remarkable efficacy in forging carbon–element bonds, B­(C_6_F_5_)_3_-catalyzed selective C–H functionalization of arenes remains a notable scarcity within the existing literature.^52,56–58^ Leveraging the heterolytic activation mode of borane catalysts, we postulate that B­(C_6_F_5_)_3_ can polarize the N–S bond of *N,N′*-thiobisphthalimide similar to the N–S bond activation observed in our previous studies.^52^ This can be utilized to enable a streamlined single atom transfer strategy using easily prepared inexpensive starting materials. In 1972, Harpp and co-workers first reported *N,N′-*thiobisphthalimide; however, its reactivity remained largely unexplored in the subsequent decades, and it was never fully characterized.^59^ Recently, Jiang and co-workers demonstrated the use of *N,N′*-thiobisphthalimide for the synthesis of disulfides.^60^ Herein, we report a fundamentally distinct approach in sulfur chemistry, introducing *N*,*N*′-thiobisphthalimide as a sulfenylating agent in the borane-catalyzed synthesis of diverse symmetrical diaryl/diheteroaryl thioethers via a sulfur atom transfer strategy. Notably, in our reactions, phthalimide is the sole byproduct allowing it to be easily recovered and reused, enabling an atom-economical, thiol-free approach.

## Results and Discussion

In continuation of our ongoing interest in N–S bond activation within thiosuccinimide frameworks via borane catalysis,^52,61^ we applied a similar approach to the N–S bond of *N,N′*-thiobisphthalimide to enable the selective formation of diaryl thioethers. In our earlier study, arylthiosuccinimides were used as sulfenylating agents, producing succinimide as a sacrificial byproduct and also requiring foul-smelling, toxic thiols for the synthesis of the arylthiosuccinimides. In contrast, our present protocol employs *N,N′*-bisthiophthalimide as a sulfur atom transfer reagent, producing recoverable phthalimide as a benign byproduct which is reused for the synthesis of *N,N′*-thiobisphthalimide, thus enabling a thiol-free, atom-economical, and recyclable thioetherification process. *N,N′*-Thiobisphthalimide (**1a**) was prepared from commercially available phthalimide and sulfur monochloride (S_2_Cl_2_) under mild conditions (DMF at 0 °C),^60,62^ offering a straightforward and scalable thiol-free route to the sulfenylating reagent (up to 5.0 g, 62% yield). To investigate the application of *N,N′*-thiobisphthalimide (**1a**) as a sulfenylating reagent, we reacted it with *N,N*-dimethylaniline (**2a**) as the arene coupling partner (Table 1). The reaction proceeded using 20 mol % B­(C_6_F_5_)_3_ in dichloroethane at 80 °C, affording the desired diaryl thioether product (**3aa**) in 84% yield (Table 1, entry 1). Conducting the reaction at ambient temperature resulted in a very low yield of product (**3aa**, 18%), with significant recovery of unreacted *N,N′*-thiobisphthalimide (**1a**), indicating that elevated (reflux) temperatures are essential for effective N–S bond activation (Table 1, entry 2). Notably, a persistent precipitate was observed throughout the room-temperature reaction, suggesting limited solubility of *N,N′*-thiobisphthalimide (**1a**) under these conditions. In contrast, heating the reaction mixture to reflux instantly afforded a clear solution, and a white precipitate formed only at the end of the reaction. This was attributable to the precipitation of the regenerated sparingly soluble byproduct phthalimide in 1,2-dichloroethane.

**Table 1:**
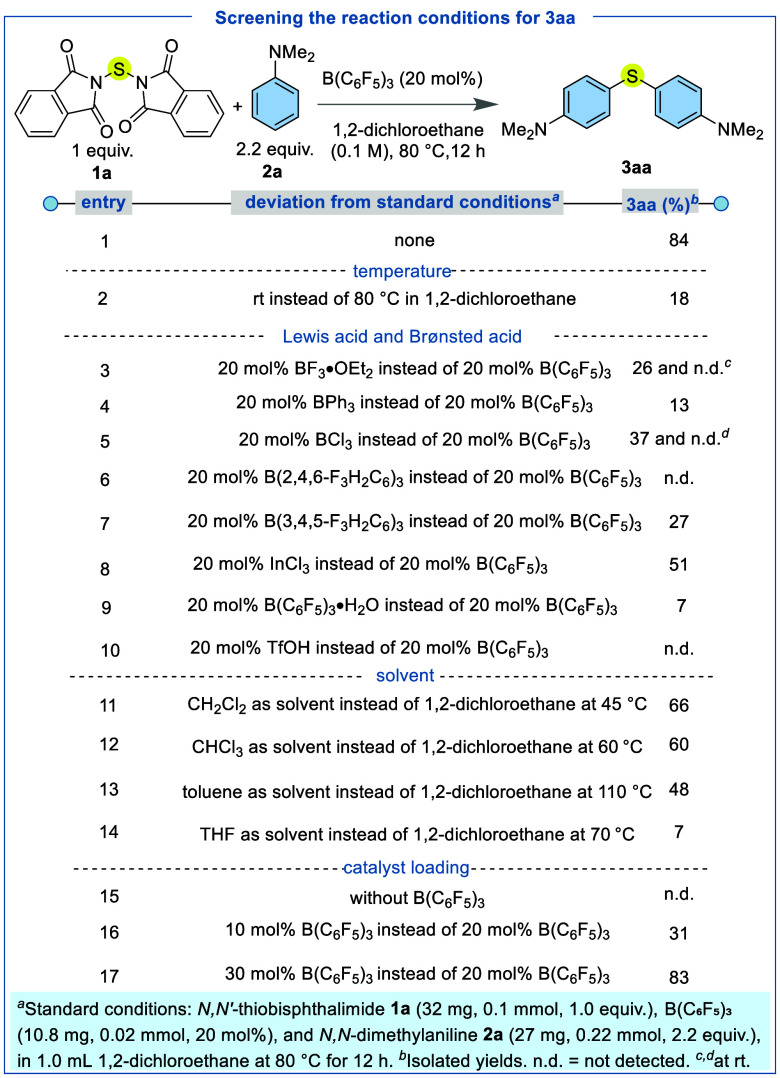
Optimization of the Reaction Conditions

To gain mechanistic insight into the Lewis acid-mediated activation of the N–S bond in *N,N′*-thiobisphthalimide (**1a**), we systematically screened several boron-based Lewis acids, including BF_3_·OEt_2_, BPh_3_, BCl_3_, B­(2,4,6-F_3_C_6_H_2_)_3_, and B­(3,4,5-F_3_C_6_H_2_)_3_ (Table 1, entries 3–7). With BF_3_·OEt_2_ and BCl_3_, low yields of **3aa** (26% and 37%, respectively) and poor selectivity resulted, with the formation of the *ortho*/*para* mixed isomer **3aa′** also being observed (11% and 18%, respectively; see Supporting Information for **3aa′** structure analysis), under reflux. At room temperature, the reactions did not lead to the final product and instead stopped at an intermediate mono C–S coupled product wherein only one phthalimide moiety was replaced by the aryl group, in addition to unreacted starting materials (confirmed by LCMS). The weaker Lewis acid BPh_3_ afforded only 13% product with predominant recovery of *N,N′*-thiobisphthalimide (**1a**), indicating poor activation of the carbonyl oxygen atom and consequent failure to promote N–S bond cleavage for single atom sulfur transfer. The reaction with B­(2,4,6-F_3_C_6_H_2_)_3_ exhibited 0% yield of **3aa**, whereas B­(3,4,5-F_3_C_6_H_2_)_3_ produced only 27% yield of the desired product. The heavier group 13 Lewis acid InCl_3_ produced **3aa** with only 51% yield (Table 1, entry 8). Brønsted acid catalysis using B­(C_6_F_5_)_3_·H_2_O was also tested^63^ but could deliver only 7% of the desired product. Likewise the reaction performed with TfOH was also ineffective (Table 1, entries 9 and 10). This suggested that B­(C_6_F_5_)_3_ is a superior catalyst for the selective C–S bond formation strategy. Other solvents, including dichloromethane, chloroform, toluene, and tetrahydrofuran (THF), were evaluated at their respective boiling points (Table 1, entries 11–14). Among these, the chlorinated solvents dichloromethane and chloroform afforded comparatively higher yields (**3aa**, 66% and 60%, respectively) than toluene (**3aa**, 48%) and THF (**3aa**, 7%). Nevertheless, none of these solvents surpassed the efficiency observed with 1,2-dichloroethane (DCE), which remained the optimal medium for the transformation. Exclusion of the B­(C_6_F_5_)_3_ catalyst completely suppressed product formation, demonstrating that thermal activation alone is insufficient to induce N–S bond cleavage and underscores the critical role of the borane catalyst in facilitating the sulfur transfer process (Table 1, entry 15). Reducing the catalyst loading to 10 mol % resulted in a significantly diminished yield of **3aa** to 31%, whereas increasing the loading to 30 mol % did not enhance the reactivity further, affording a comparable yield (**3aa**, 83%) (Table 1, entries 16 and 17).

Scaling the reaction of *N*,*N*′-thiobisphthalimide (**1a**) with *N*,*N*-diethylaniline (**2b**) to 2 mmol furnished the desired thioether **3ab** in 81% isolated yield (532 mg, 1.62 mmol). Notably, phthalimide, the only byproduct generated in the reaction, was recovered in near-quantitative yield (247 mg, 1.68 mmol, 84%). This demonstrates its recyclability for the regeneration of *N*,*N*′-thiobisphthalimide and highlights the sustainability of this sulfur transfer strategy (Figure 2A). Interestingly, treatment of *N,N*′-thiobisphthalimide (**1a**) with *N,N*-dimethyl-[1,1′-biphenyl]-2-amine (**2c**) at room temperature afforded the mono C–S coupling product **4** with 40% yield, wherein only one phthalimide moiety was replaced by the aryl group (Figure 2B). This outcome points to a stepwise mechanism, with compound **4** serving as a likely key intermediate in the reaction. As previously demonstrated in copper-catalyzed systems, elemental sulfur can serve as a sulfur source for the synthesis of unsymmetrical thioethers albeit there are issues such as overoxidation, poor selectivity, and the generation of side products.^64^ Given the accessibility of elemental sulfur as a commercial feedstock, we envisioned that the B­(C_6_F_5_)_3_ catalyst might enable its activation for thioetherification. To underscore the necessity of our sulfenylating agent, the optimized reaction conditions were applied to *N,N*-dimethylaniline (**2a**) in the presence of elemental sulfur, aiming to generate the corresponding symmetrical thioether. However, the reaction proved unproductive, thereby ruling out elemental sulfur as a viable sulfur source with the current catalytic system (Figure 2C). We further extended our single sulfur atom transfer strategy to enable the synthesis of sulfur heterocycles. Notably, the dimethylamino substituted biaryl substrate **5a** ultimately afforded a substituted dibenzothiophene (DBT) derivative **6aa** with 37% yield through single atom transfer from **1a** (Figure 2D). The process is proposed to initiate via an initial intermolecular C–S bond formation, followed by an intramolecular cyclization in a one-pot process. This intramolecular C–S transformation showcases an efficient route to five-membered sulfur-containing heterocyclic frameworks from simple biphenyl precursors. Unfortunately, however, the dibenzothiophene derivative **6ab** could not be achieved from **1a** and methoxy substituted biaryl substrate **5b**. The UV–vis. spectrum of the *para*-NMe_2_-substituted dibenzothiophene derivative (**6aa**) displays two absorption bands: a strong band at 331 nm, and a weaker one at 378 nm (Figure 2E). The prominent absorption at 331 nm is assigned to an allowed π → π* transition, facilitated by the rigid and planar conjugated framework of dibenzothiophene and the electron-donating nature of the NMe_2_ groups. The minor band at 378 nm is attributed to a *n* → π* transition involving the electron-rich NMe_2_ substituents and the dibenzothiophene core.^65^ In contrast, the analogous diaryl sulfide derivative bearing two *para*-NMe_2_ groups (**3aa**) shows no significant absorption. This difference can be attributed to the lack of a rigid and extended π-conjugated system in the diaryl sulfide (**3aa**), which leads to poor orbital overlap and prevents efficient electronic transitions. This comparison highlights the critical role of the dibenzothiophene scaffold in enabling conjugation-driven optical transitions. Cyclic voltammetry (CV) also provided valuable insight for these diaryl thioether cores. In order to study the cyclic voltammic properties of the reaction products, we prepared two further derivatives bearing *p*-OMe (**3ai**) and *p*-SMe (**3aq**) groups. Under the optimized conditions, these could be prepared in 78% and 72% yield respectively. Compounds **3aa**, **3ai**, and **3aq** show three quasi-reversible oxidation and reduction peaks (Figure 2F, left), indicating ambipolar redox stability essential for robust photoredox mediators.^66,67^ Among these, **3aa** exhibits the lowest onset oxidation (0.51 V) and accessible reduction (0.42 V), offering the broadest redox window for diverse substrate compatibility and efficient electron cycling. In contrast, **6aa** shows only irreversible oxidation and no reduction peaks (Figure 2F, right), aligning with a role as a sacrificial electron donor rather than a true mediator. DFT-calculated HOMO–LUMO gaps support these findings (Figure 3), the smaller gap in **6aa** (6.02 eV) allows easier oxidation but limits redox reversibility, while the larger gaps in **3aa** (6.81 eV), **3ai** (7.33 eV), and **3aq** (6.91 eV) promote reversible redox cycling.^67,68^ These characteristics are in line with established photoredox principles, where the mediator efficiency depends on both redox accessibility and stable cycling.^69,70^

**Figure 2:**
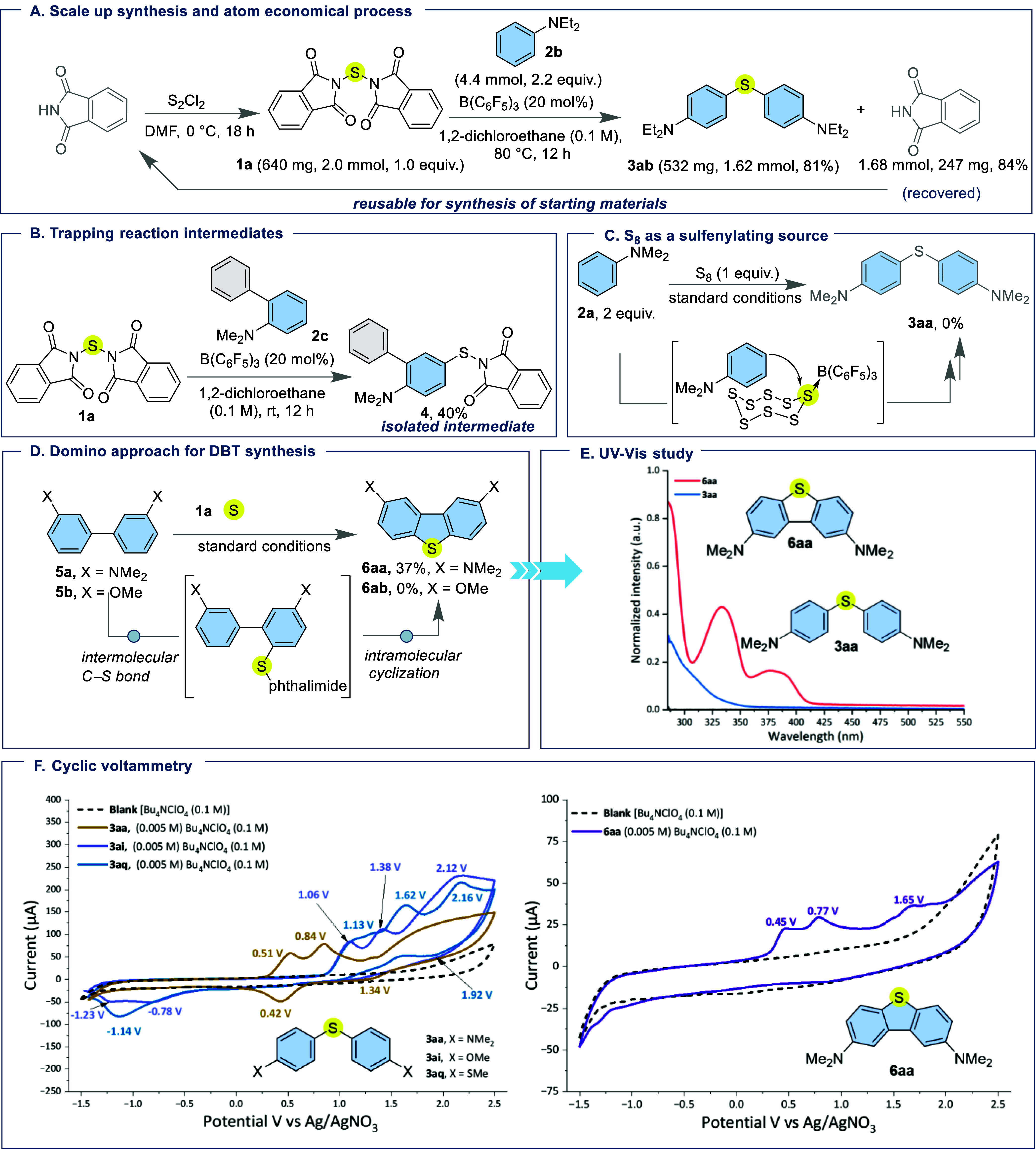
Reaction development, control experiments, and mechanistic investigations for the synthesized thioethers. (A) Recovery of the phthalimide byproduct, highlighting the atom-economical nature of the process. (B) Isolation of intermediate **4** at room temperature, supporting a stepwise pathway. (C) Control experiment employing molecular sulfur (S_8_) as the sulfur source in place of **1a**. (D) Single atom transfer strategy for the synthesis of dibenzothiophene **6aa** from biaryl **5a** and **1a**. (E) UV–vis. absorption studies of **3aa** and **6aa**. (F) Cyclic voltammetry analysis of **3aa**, **3ai**, **3aq**, and **6aa**.

**Figure 3:**
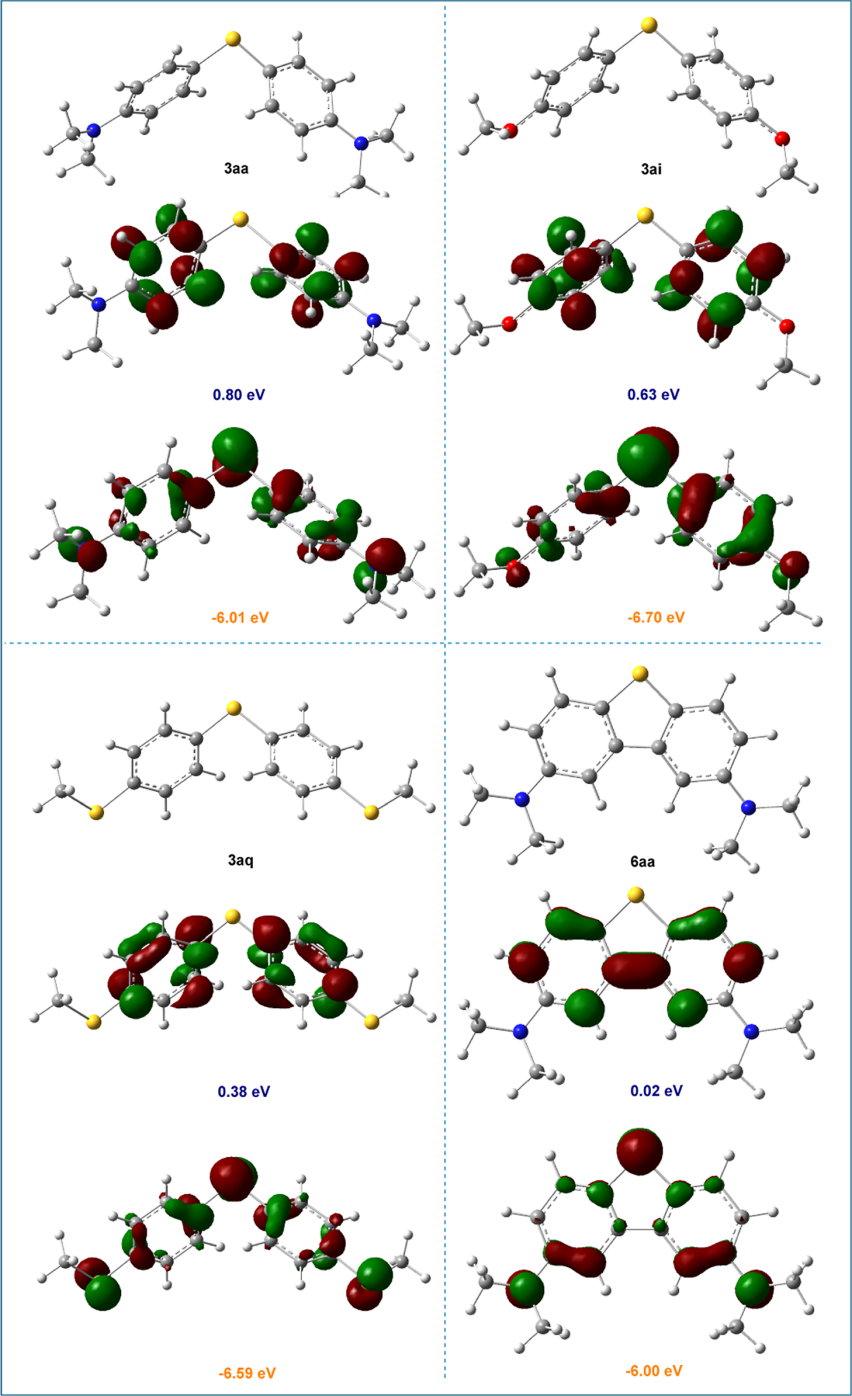
HOMO and LUMO energies of compounds **3aa**, **3ai**, **3aq**, and **6aa**, obtained from NBO analysis based on DFT calculations at the SMD/M06-2X/6-31G­(d) level in 1,2-dichloroethane. The isosurfaces represent the spatial distributions of the frontier molecular orbitals. Orange values indicate HOMO energies and blue values indicate LUMO energies.

To gain deeper insight into the mechanism of sulfur atom transfer process for the symmetrical thioetherification reaction, we carried out a comprehensive DFT study at the SMD/M06-2X/def2-TZVP//SMD/M06-2X/6-31G­(d) level of theory in 1,2-dichloroethane as the solvent (Figure 4). For our computational investigation, we selected the model reaction between *N,N′*-thiobisphthalimide (**1a**) and *N,N*-dimethylaniline (**2a**) leading to product **3aa** in the presence of B­(C_6_F_5_)_3_ as a catalyst. According to our DFT results, the reaction is initiated by the coordination of B­(C_6_F_5_)_3_ to one of the carbonyl oxygen atoms of **1a** via transition state **TS**_**1**_, leading to the formation of adduct **add**_**1**_ (Figure 4a). We also evaluated the parallel N → B coordination between B­(C_6_F_5_)_3_ and **2a**, affording adduct **add**_**1‑N**_. This pathway is energetically high, requiring 14.8 kcal mol^–1^ compared to 1.0 kcal mol^–1^ for **add**_1_, confirming the preference of adduct **add**_**1**_ over adduct **add**_**1‑N**_. Following the formation of **add**_**1**_, activation of the N–S bond renders it elongated and more labile, leading to N–S bond cleavage and concomitant C–S bond formation via **TS**_**2**_ (Δ*G* ^⧧^ = 24.6 kcal mol^–1^). This step furnishes the ion-pair intermediate **ip**_**1**_ (Δ*G* = 7.2 kcal mol^–1^). The DFT results identify this transformation as the rate determining step of the reaction, and the calculated barrier is consistent with the experimental requirement for elevated temperature to achieve efficient conversion. Given the critical role of this step, we performed a conformational search for **TS**_**2**_. The lowest-energy conformer exhibits the activation barrier of 24.6 kcal mol^–1^, while additional conformers were located with barriers in the range of 25.2–28.9 kcal mol^–1^ (Figure S3), confirming that **TS**_**2**_ remains the most relevant transition structure for the rate-determining step. The ion-pair intermediate **ip**_**1**_ subsequently undergoes deprotonation, assisted by a phthalimide anion, via **TS**_**3**_ (Δ*G* ^⧧^ = 16.5 kcal mol^–1^), affording **add**_**2**_, which contains B­(C_6_F_5_)_3_ bridged 2-(4-(dimethylamino)­phenylthio)­phthalimide and phthalimide (Δ*G* = −10.8 kcal mol^–1^). The B­(C_6_F_5_)_3_ is then transferred to the 2-(4-(dimethylamino)­phenylthio)­phthalimide through O → B interaction with phthalimide (**TS**_**4**_) with an activation barrier of 9.0 kcal mol^–1^, leading to the formation of adduct **add**_**3**_ and concomitant release of the first molecule of phthalimide (Δ*G* = −14.5 kcal mol^–1^). Notably, a related key intermediate **4** (Figure 2B, vide supra), structurally analogous to 2-(4-(dimethylamino)­phenylthio)­phthalimide, was also experimentally isolated. This finding supports our computational result that the Ar–S–phthalimide is an intermediate in the reaction. From **add**_**3**_, reaction with another equivalent of **2a** proceeds via cleavage of the second N–S bond along with C–S bond formation through **TS**_**5**_ (Δ*G* ^⧧^ = 23.2 kcal mol^–1^), yielding the ion-pair intermediate **ip**_**2**_. In addition to **TS**_**2**_, we also carried out a conformational search for **TS**_**5**_, as shown in Figure S4. The lowest-energy conformer was calculated at 8.7 kcal mol^–1^ for **TS**_**5**_, while additional conformers (**TS**_**5‑a**_ to **TS**_**5‑h**_) were identified with relative energies spanning 10.4–23.7 kcal mol^–1^, thereby confirming **TS**_**5**_ as the most relevant transition structure. A concerted pathway was also examined, in which **add**_**2**_ is directly reacted with an additional equivalent of **2a**. However, as depicted in Figure 4a, the calculated activation barrier for the concerted transition state **TS**_**4‑concerted**_ (Δ*G* ^⧧^ = 31.0 kcal mol^–1^) is significantly higher and thus not kinetically favorable compared to the stepwise pathway proceeding via **add**_**3**_. These results, combined with experimental evidence, confirm that the reaction proceeds through a stepwise, ion-pair mechanism. Following the formation of the ion-pair intermediate **ip**_**2**_, a second deprotonation occurred via **TS**_**6**_, surmounting an energy of 4.6 kcal mol^–1^, ultimately affording the diaryl thioether **3aa** as the product, accompanied by the recovery of phthalimide and the regeneration of B­(C_6_F_5_)_3_ as the catalyst with an overall reaction energy of −25.1 kcal mol^–1^. The regioselectivity of the first C–S bond-forming step was further examined by comparing the *para* and *ortho* directing approaches of **2a** to the **add**_**1**_ (Figure 4b). As mentioned earlier, the computed barrier for **TS**_**2**_ (*para*) is 24.6 kcal mol^–1^, which is 1.2 kcal mol^–1^ lower than that of **TS**_**2‑** *ortho*_ (Δ*G* ^⧧^ = 25.8 kcal mol^–1^), in agreement with the experimentally observed exclusive *para* selective C–S bond formation with *N,N*-dimethylaniline (**2a**). Natural population analysis (NPA) indicates that the out-of-plane 2p (π) orbital at the reacting aryl carbon (C^a^) is more populated in **TS**_**2**_ (*para*) (1.226) than in **TS**_**2‑** *ortho*_ (1.191). The higher π density at C^a^ implies more effective delocalization into the sulfur acceptor along the C–S bond-forming coordinate, stabilizing **TS**_**2**_ and explaining the observed *para* selectivity. The *ortho* pathway remains consistently disfavored, as **ip**_**1‑** *ortho*_ and **TS**_**3‑** *ortho*_ lie 1.3 and 4.9 kcal mol^–1^ higher in energy, respectively, than their *para* counterparts. Thus, the higher energy of both transition **TS**_**2‑** *ortho*_ and **TS**_**3‑** *ortho*_ states shows a preference for the *para* pathway, with the selectivity becoming more pronounced at the later transition state. To confirm that this trend is not functional-dependent, we performed benchmark calculations using additional level of theories to M06-2X^71^ including M06 ^1^ above, B3LYP,^72–76^ B3LYP-D3,^77^ PBE1PBE,^78,79^ PBE1PBE-D3, and ωB97X-D^80^ (see Supporting Information, Table S1). All modern dispersion-inclusive methods (M06-2X, M06, B3LYP-D3, PBE1PBE-D3, and ωB97X-D) consistently predict the *para* pathway lower in energy at **TS**_**2**_, **ip**_**1**_ **TS**_**3**_, with the energetic separation being significantly larger at **TS**_**3**_ (4.9–6.6 kcal mol^–1^). In contrast, dispersion-free functionals (B3LYP, PBE1PBE) either underestimate barriers or misrepresent the selectivity, highlighting the necessity of including dispersion for reliable predictions.

**Figure 4:**
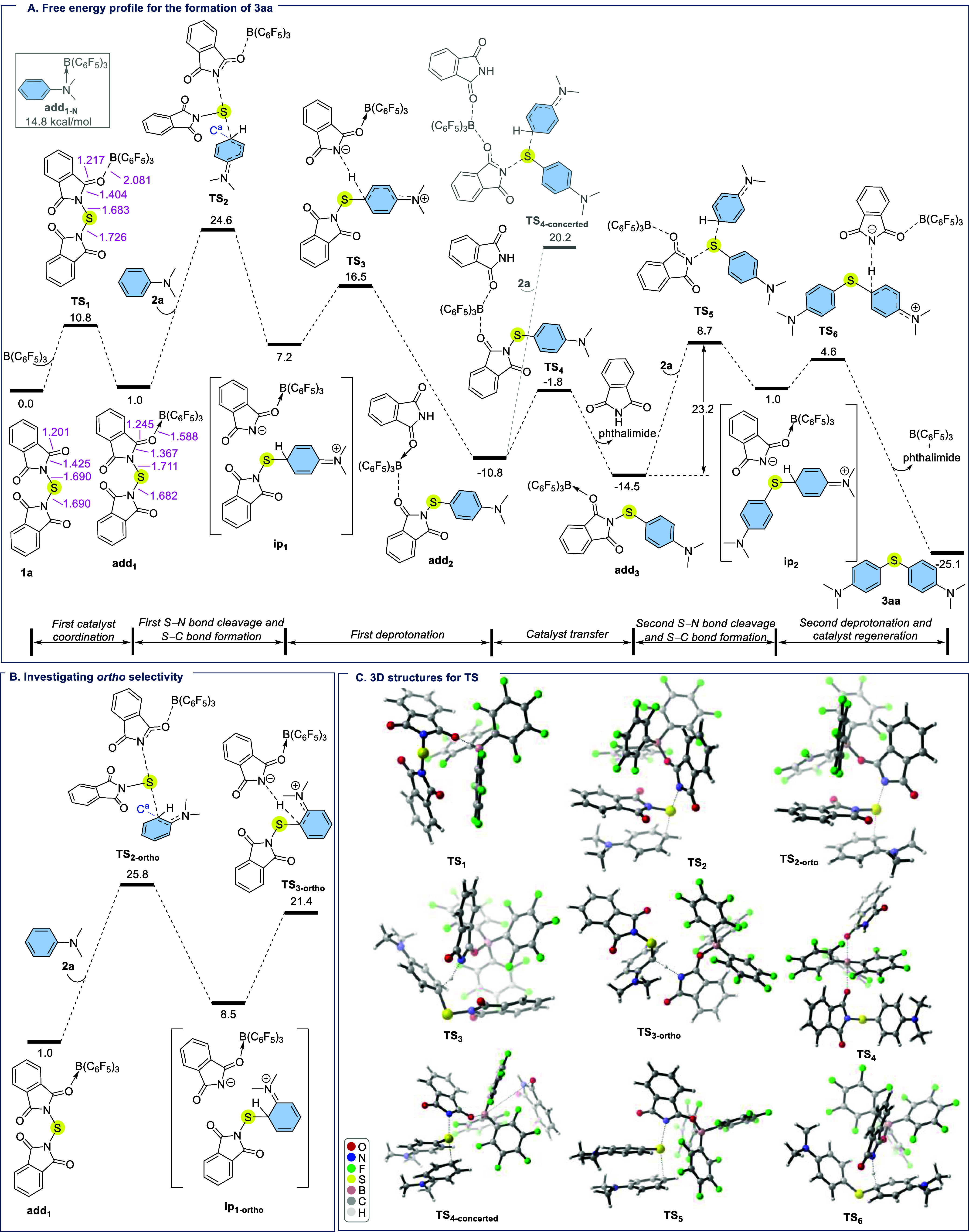
(A) Free energy profile for the formation of **3aa**. (B) Calculations into *ortho* selectivity. (C) 3D structures for the transition states located for the proposed mechanism using CYLview software calculated in 1,2-dichloroethane at the SMD/M06-2X/def2-TZVP//SMD/M06-2X/6-31G­(d) level of theory. Bond lengths (pink color) shown in Å, and relative free energies are given in kcal mol^–1^.

Next, we turned our attention to examining the reactivity between **1a** and the biaryl substrate **5a**, which produces dibenzothiophene **6aa** via an intramolecular C–S coupling. Our DFT calculations revealed that this transformation proceeds through the similar type of stepwise ionic SAT pathway as established for the formation of **3aa** (see Supporting Information, Figure S5), confirming that the proposed mechanism is general. However, as shown in Figure S5, although the rate-determining transition state (**TS**_**2′**_) for this pathway has a slightly higher activation barrier (Δ*G* ^⧧^ = 24.9 kcal mol^–1^) compared to **TS**_**2**_ in the formation of **3aa** (Δ*G* ^⧧^ = 24.6 kcal mol^–1^), the intramolecular second C–S bond formation followed by deprotonation is more energetically favorable, with relative energies of 6.9, −9.2, and −7.6 kcal mol^–1^ for **TS**_**5′**_, **ip**_**2′**_, and **TS**_**6′**_, respectively. We further examined the regioselectivity of the C–S bond-forming step for biaryl **5a**, and the results again show a clear preference for the *para* trajectory over the *ortho* pathway, in full agreement with the computed energy barriers (Figure S6, pathway A) and experiment (Figure 2D, vide supra). The calculated activation barrier for **TS**_**2′‑** *ortho*_, along with the energies of **ip**_**1′‑** *ortho*_ and **TS**_**3′‑** *ortho*_, were found to be 30.1, 14.4, and 26.1 kcal mol^–1^ higher, respectively, than those of their *para* counterparts.

We also examined the potential formation of dibenzothiophene derivative **6ab** bearing *para*-methoxy (OMe) substituents in place of the NMe_2_ groups. The calculated activation barrier for the N–S bond cleavage and C–S bond formation step (**TS**_**2′‑OMe**_) was found to be 36.2 kcal mol^–1^, and the corresponding ion-pair intermediate (**ip**_**1′‑OMe**_) lies at 22.6 kcal mol^–1^ (see Figure S6, pathway B). These values are far too high to be accessible under the experimental conditions, thereby rationalizing why the formation of product **6ab** was not observed experimentally. To further explain this result, we compared the HOMO–LUMO energy gaps between (i) the HOMO of **5a** bearing two *para-*NMe_2_ substituents, and (ii) the HOMO of **5b** bearing two *para-*OMe substituents, with the LUMO of **1a**. Our DFT calculations revealed that the interaction between HOMO of **5a** (energy gap: 4.06 eV) is more significant than from that of **5b** (energy gap: 4.89 eV). This difference accounts for the lower activation barrier observed for **TS**_**2′**_ compared to **TS**_**2′‑OMe**_.

The calculated free energy profile suggests that the step corresponding to **TS**_**2**_ is the rate-determining step (unless the reaction conditions are far from the standard state). Formation of **add**_**1**_ from **1a** and B­(C_6_F_5_)_3_ is an equilibrium before the rate-determining step, albeit an equilibrium that is anticipated to lie to the left at typical reaction concentrations. The DFT calculations, therefore, suggest a rate law of the form rate = *k* × [**1a**] × [B­(C_6_F_5_)_3_] × [**2a**]. To confirm the relatively weak interaction between **1a** and B­(C_6_F_5_)_3_, a titration of B­(C_6_F_5_)_3_ with **1a** in CDCl_3_ was monitored using ^19^F NMR spectroscopy. In agreement with the DFT calculations, tight binding was not observed. Subsequently, kinetic studies were carried out to confirm the rate law. The reaction was followed using ^1^H NMR spectroscopy for 0.1 M **1a**, 20 mol % of B­(C_6_F_5_)_3_ and 0.12 M **2a**, i.e. the standard reaction conditions, but in CDCl_3_ instead of 1,2-dichloroethane as the solvent and at 45 °C instead of at 60 °C. 1,2-Dichloroethane (0.1 M) was used as an internal standard. The concentrations of B­(C_6_F_5_)_3_ and **2a** were doubled to evaluate the reaction order (doubling the concentration of **1a** resulted in precipitation). In all reactions, the phthalimide byproduct started to precipitate after some time, resulting in unstable kinetic traces (Figure S67). We therefore restricted ourselves to determining the initial rates of the reactions (data for phthalimide formation shown in Figure 5).

**Figure 5:**
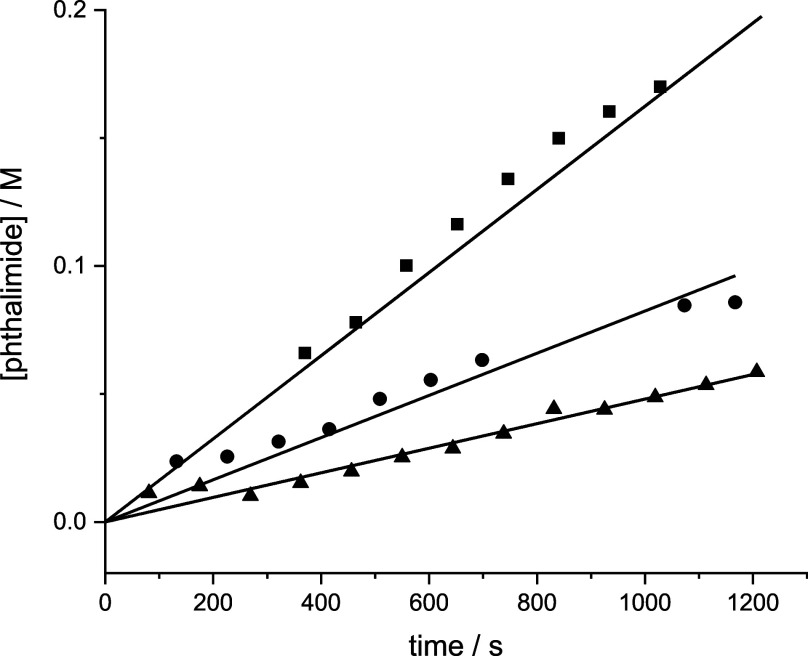
Concentration of phthalimide as a function of time for 0.1 M **1a**, 20 mol % of B­(C_6_F_5_)_3_ and 0.12 M **2a** (●), 0.1 M **1a**, 40 mol % of B­(C_6_F_5_)_3_ and 0.12 M **2a** (■) and 0.1 M **1a**, 20 mol % of B­(C_6_F_5_)_3_ and 0.24 M **2a** (▲), in CDCl_3_ at 45 °C with 0.1 M 1,2-dichloroethane as internal standard.

Figure 5 shows that the initial rate doubles upon doubling the catalyst concentration (Table S2). This is in line with the DFT calculations. On the contrary, the initial rate decreases upon doubling the concentration of **2a**. Although **2a**-B­(C_6_F_5_)_3_ complex formation (adduct **add**_**1‑N**_) is comparatively high in energy according to our DFT calculations, exposing B­(C_6_F_5_)_3_ to **2a** in the absence of **1a** shows the formation of several new peaks in ^19^F NMR spectra and results in a visible color change, suggesting a possible interaction or single electron transfer (SET) between **2a** and B­(C_6_F_5_)_3_ which is also in agreement with the previous literature.^81^ The observed decrease in rate upon increasing the concentration of **2a** is therefore attributed to partial reaction of the B­(C_6_F_5_)_3_ catalyst with **2a**. This partial deactivation of B­(C_6_F_5_)_3_ by **2a** also justifies the requirement for a high catalyst loading (20 mol %) in the reaction. We note that the reactivity of B­(C_6_F_5_)_3_ is only expected to occur for the aniline derivatives and not for the other substrates used here.

Next, we evaluated the scope of the arenes and heteroarenes for the single atom transfer thioetherification reaction, keeping 12 h reaction time for all substrates to ensure reaction completion (Figure 6). Various *N*-substituted aniline derivatives (**2a**–**2h**) were selectively amenable to produce symmetrical thioethers (**3aa**–**3ah**) with 28–97% yields. In the case of *N,N*-dimethylaniline **2a**, a minor *ortho*/*para* mixed product, 2-((4-(dimethylamino)­phenyl)­thio)-*N*,*N*-dimethylaniline (**3aa**′, 7% yield), was isolated, while no *ortho* product was detected (see SI). Notably, *N,N*-dimethyl-[1,1′-biphenyl]-2-amine (**2c**), despite possessing a biaryl framework, did not undergo cyclization to form dibenzothiophene as observed for biaryl substrate **5a**. Instead, it selectively afforded the linear diarylthioether **3ac** in 38% yield, leaving the pendant phenyl ring unreactive. Similarly, *ortho*-bromo-*N,N*-dimethylaniline furnished the dibromo-substituted symmetrical diarylthioether **3ag** in 28% yield. The aryl C–Br bond in the product provides a versatile handle for potential functionalization through Suzuki, Sonogashira, or Heck cross-couplings, while the sulfur center in all the products can be selectively oxidized to generate sulfoxides, sulfones, or sulfoximines. The symmetrical diarylthioether **3ah** was also produced in 66% yield. Subsequently, the substrate scope was broadened to incorporate a range of aryl ether derivatives, which underwent efficient *para*-selective C–H sulfenylation to furnish products **3ai**–**3al** in commendable yields of 58–78%. Electron-rich di- and trisubstituted arenes bearing methoxy (OMe) and ethoxy (OEt) substituents also proved highly compatible under the established sulfur-transfer protocol, affording sulfenylated products **3am**–**3ap** in 44–62% yields. Prefunctionalized thioether derivatives were likewise amenable to this borane-catalyzed C–H sulfenylation, enabling the installation of an additional thioether unit within the molecular scaffold. These advanced thioether frameworks (**3aq**–**3at**), obtained in 45–72% yields. These products contain three conjugated sulfur atoms which exhibit distinctive electronic and structural attributes arising from π-conjugation and the sulfur atoms’ electron-donating character.^82,83^ This single sulfur atom transfer reaction strategy was also successful for heterocycles to produce symmetrical diheteroarylthioethers. For instance, substituted pyrrole, indole, carbazole and imidazo­[1,2-*a*]­pyridine were well tolerated to give products (**3au**–**3ax**) with 22–66% yields. Pyrrole- and imidazo­[1,2-*a*]­pyridine-based sulfides were synthesized for the first time using this protocol. While di-indole sulfides have previously been reported by Ingleson using the Xtalfluor-*E*-amine adduct,^84^ this combination is most likely to be highly unstable, not readily accessible, and lacks the flexibility for structural diversification. Furthermore, dicarbazole sulfide frameworks have traditionally been prepared by using expensive transition-metal catalysis; however, such carbazole-based sulfides are of significant interest due to their promising applications in organic light-emitting diodes.^85^ During the evaluation of the substrate scope, we observed that the nucleophilicity of arenes and heteroarenes shows a decisive role in the C–S bond formation reaction. Electronically deactivated arenes or inherently weak π-nucleophiles such as 1-chloro-2-methoxybenzene (**2y**), 9-bromo-9*H*-fluorene (**2z**), 2-methoxynaphthalene (**2z** ^**1**^), and dibenzo­[*b*]­thiophene (**2z** ^**2**^) were unreactive under the standard borane-catalyzed conditions (Figure 6C). Following this observation, we tried to evaluate other stronger Lewis acids such as BBr_3_, AlCl_3_, InCl_3_, and FeCl_3_, as well as Brønsted acids including TfOH, Tf_2_NH, and TFA for a range of electronically deactivated system to promote the reactivity; however, no productive C–S transformation was detected under these conditions (see Supporting Information, Scheme S7). We further hypothesized that increasing the electrophilicity of the sulfur center by introducing electron-withdrawing substituents (such as –F, –Cl, and –Br) onto the phthalimide framework of *N,N′-*thiobisphthalimide **1** **a** might facilitate reaction with weakly nucleophilic arenes. However, despite several efforts, derivatization of *N,N′-*thiobisphthalimide with electron-withdrawing groups was unsuccessful (see Supporting Information, Scheme S8).

**Figure 6:**
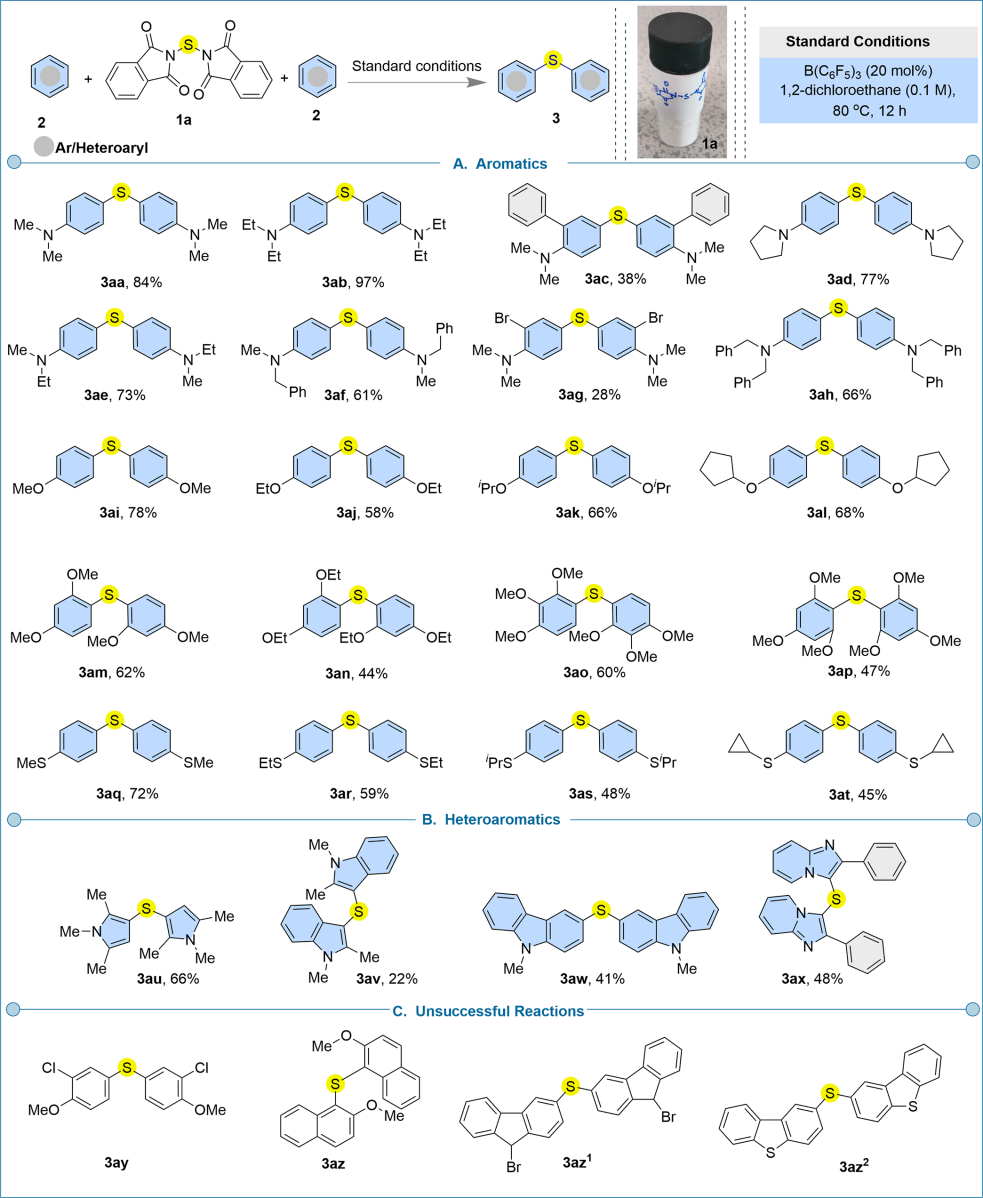
Library of symmetrical thioether derivatives synthesized on a 0.1 mmol scale under the standard reaction conditions. (A) Expanded scope of aromatic systems in symmetrical thioetherification. (B) Scope of heteroaromatic systems in symmetrical thioetherification. (C) Unsuccessful reactions.

In summary, we have unveiled *N,N′*-thiobisphthalimide as a novel, robust, bench-stable sulfenylating agent that can reveal a streamlined and metal-free approach to the construction of symmetrical diaryl/diheteroaryl thioethers and dibenzothiophenes. The strategic deployment of B­(C_6_F_5_)_3_ as a Lewis acid catalyst enables a single atom transfer pathway through a distinct arylthiophthalimide intermediate, offering an operationally simple methodology leading to reasonably high yields of symmetrical thioether products. This work not only expands the synthetic utility of *N,N′*-thiobisphthalimide but also sets the stage for future development of sulfur transfer methodologies in complex molecular settings. Further exploration of the reactivity landscape and synthetic potential of this sulfur reagent is currently underway in our laboratory.

## Supplementary Material



## Notes

This includes experimental procedures, spectroscopic data (^1^H NMR, ^13^C NMR, IR, and HRMS), and DFT calculations (PDF)
Information about the data that underpins the results presented in this article can be found in the Cardiff University data catalogue at DOI: 10.17035/cardiff.31077466. This repository contains spectroscopic data in their raw (NMR) and processed forms (mass spectrometry).
