# Supplementary material for: Thiol-Free Sulfenylation Redefined: A Single-Atom Transfer Pathway to Symmetrical Di(hetero)arylthioethers via B(C6F5)3 Catalysis
Source: J Am Chem Soc. 2026 Jan 29;148(5):5325–37. doi: 10.1021/jacs.5c17932 (PMC12903844; doi:10.1021/jacs.5c17932)
Supplement: Supplementary file 1 [file ja5c17932_si_001.pdf]

Supporting Information for

Thiol-Free Sulfenylation Redefined: A Single-Atom Transfer Pathway to Symmetrical Di(hetero)arylthioethers via $B(C_6F_5)_3$ Catalysis

Milan Pramanik,[†a] Nusaybah Alotaibi,[†a,b] Tribani Boruah,[a,c] Niklaas J. Buurma,[c] Rasool Babaahmadi,[a] Thomas Wirth,*[c] and Rebecca L. Melen*[a]*

[a] Ms Nusaybah Alotaibi, Dr Rasool Babaahmadi, Dr Milan Pramanik, Ms Tribani Boruah, Prof. Dr Rebecca L. Melen

Cardiff Catalysis Institute, School of Chemistry, Cardiff University, Translational Research Hub, Maindy Road, Cathays, Cardiff, CF24 4HQ, Cymru/Wales, UK. Email: MelenR@cardiff.ac.uk.

[b] Ms Nusaybah Alotaibi

Department of Chemistry, King Faisal University, College of Science, P.O. Box 400, Al Ahsa 31982, Saudi Arabia.

[c] Prof. Dr Thomas Wirth

School of Chemistry, Cardiff University, Main Building, Park Place, Cardiff, CF10 3AT, Cymru/Wales, UK. Email: Wirth@cardiff.ac.uk

[†] Equal contribution

Table of contents

1. Experimental Details	S3
2. Synthesis and Spectral Characterization	S3–S17
3. Control Experiments	S17–S19
4. Optoelectronic and CV Studies	S19–S20
5. Unsuccessful Reactions	S20–S21
6. Computational Details	S21–S115
7. References	S115–S116
8. NMR Spectra	S117–S174
9. Kinetic Experiments	S175–S177

1. Experimental

1.1 General experimental

All reactions and manipulations were carried out under an atmosphere of dry, O_2-free nitrogen using standard double-manifold techniques with a rotary oil pump. A nitrogen-filled glove box (MBraun) was used to manipulate solids including the storage of starting materials, ambient temperature reactions, product recovery and sample preparation for analysis. Solvents were dried by employing a solvent purification system MB SPS-800 and stored under a nitrogen atmosphere. Anhydrous (with Sure/Seal™) 1,2-dichloroethane was purchased from Merck and dried over molecular sieves before use. Chemicals were purchased from commercial suppliers and used as received. All the triarylfluoroboranes were prepared as per the standard literature report.[1] Thin-layer chromatography (TLC) was performed on pre-coated aluminum sheets of Merck silica gel 60 F254 (0.20 mm). ^1H, ^{13}C and ^{19}F NMR spectra, including kinetic experiments, were recorded on a Bruker Avance II 400 or Bruker Avance 500 spectrometer. All coupling constants are absolute values and are expressed in Hertz (Hz). ^{13}C NMR spectra were measured as ^1H decoupled. Yields are given as isolated yields. Chemical shifts are expressed as parts per million (ppm, δ) downfield of tetramethylsilane (TMS) and are referenced to $CDCl_3$ (7.26/77.16 ppm) as internal standard. The description of signals includes s = singlet, d = doublet, t = triplet, q = quartet, p = pentet and m = multiplet. All coupling constants are absolute values and are expressed in Hertz (Hz). All spectra were analyzed assuming a first order approximation. IR-spectra were measured on a Shimadzu IRAffinity-1 photo-spectrometer. Mass spectra were measured on a Waters LCT Premier/XE or a Waters GCT Premier spectrometer. Ions were generated by the Atmospheric Solids, Analysis Probe (ASAP), Electrospray (ES) or Electron Ionization (EI). The molecular ion peaks values quoted for either molecular ion (M^+), molecular ion plus or minus hydrogen ($M{+}H^+$, $M{-}H^-$), molecular ion plus sodium ($M{+}Na^+$).

2. Synthesis and Characterization

2.1 Synthesis of *N,N'*-Thiobisphthalimide, 1a[1]

General Procedure a: In an oven-dried two-necked round-bottom flask equipped with a magnetic stir bar, phthalimide (7.36 g, 50 mmol, 1.0 equiv.) was dissolved in dry DMF (40 mL). To this solution, sulfur monochloride (4.00 mL, 50 mmol, 1.0 equiv.) was added dropwise at 0 °C. After 2 h, a white precipitate formed, and the reaction mixture was stirred overnight at

0 °C. The resulting solid was filtered, thoroughly washed with Et_2O, and collected to afford compound **1a** as a white solid (5.0 g, 15.42 mmol, 62%).

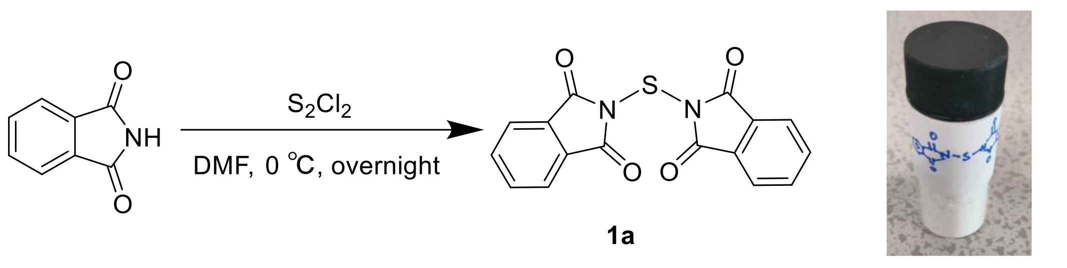

Scheme S1. Synthesis of *N,N'*-thiobisphthalimide*,* **1a.**

2.2 Synthesis of Biaryls[2]

***General Procedure b*:** An oven-dried two-necked round bottom flask was equipped with a magnetic stir bar and activated magnesium turnings (0.255 g, 10.5 mmol, 1.05 equiv.). The flask was sealed, evacuated, and backfilled with nitrogen, and dry THF (5 mL) was added. A solution of 3-bromoarene (10 mmol, 1.0 equiv.) in dry THF (10 mL) was added gradually to the suspended solution by a syringe at room temperature over 30 min. After the addition, the reaction mixture was stirred at 50 °C for 2 hours. In another oven dried two-necked round bottom flask equipped with a magnetic stir bar, $FeCl_3$ (0.487 g, 0.3 mmol, 3.0 mol%) was added and dissolved in dry THF (15 mL). 1,2-Dichloroethane (0.594 g, 6 mmol, 0.60 equiv.) was added to the $FeCl_3$ solution. The prepared solution of the Grignard reagent in THF was added dropwise via filter cannula to the $FeCl_3$ solution. The reaction mixture was left at room temperature. After 1 hour, the reaction was quenched with 20 mL of water. The desired product was extracted with CH_2Cl_2 (3 × 30 mL). The combined organic layers were dried over anhydrous $MgSO_4$. All volatiles were removed under vacuum, giving the crude product, which was purified via column chromatography using silica gel (pretreated with 1% NEt_3 in hexane) and hexane/ethyl acetate (95:5) as eluent.

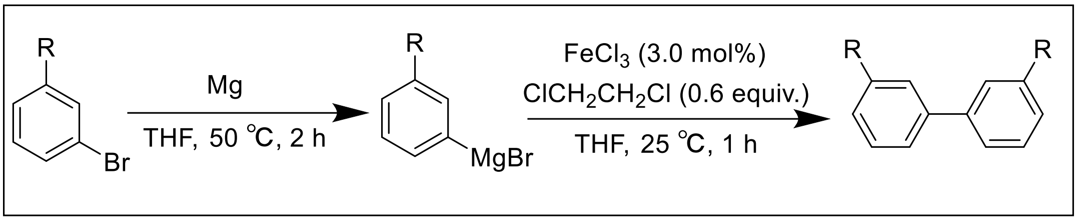

Scheme S2. Synthesis of biaryls.

2.3 Synthesis of Di(hetero)arylthioethers

***General Procedure c*:** Inside the glovebox, three glass microwave vials were charged separately with the desired arene or heteroarene (2.2 equiv.), *N,N′*-thiobisphthalimide **1a** (32 mg, 0.1 mmol, 1.0 equiv.), and $B(C_6F_5)_3$ (10.8 mg, 0.02 mmol, 20 mol%), and were then capped with a septum. The three vials were brought outside the glovebox and 1.0 mL of dry 1,2-dichloroethane was divided and added to each vial. The *N,N′*-thiobisphthalimide solution was added to the $B(C_6F_5)_3$ solution and stirred at 80 °C until a clear solution appeared. The solution of arene or heteroarene was then added to the mixture under vigorous stirring. The reaction mixture was then continued to stir at above said temperature for 12 h (a white precipitate reappeared during the course of the reaction). All volatiles were then removed in vacuo, and the crude compound was purified via preparative thin-layer chromatography using hexane/ethyl acetate as eluent.

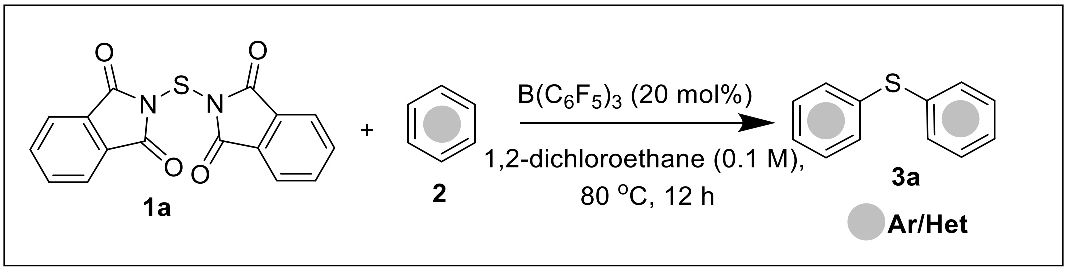

Scheme S3. Synthesis of di(hetero)arylthioethers.

2.4 Synthesis and Spectral Characterization of *N*-Dithiophthalimide, 1a

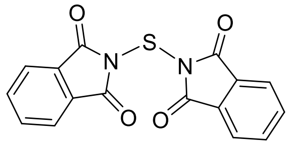

Synthesized in accordance with *General Procedure a* using phthalimide (7.36 g, 50 mmol, 1.0 equiv.) and sulfur monochloride (6.75 g, 50 mmol, 1.0 equiv.). The desired compound **1a** was filtered, washed with Et_2O and collected as a white solid. Yield: 5.0 g, 15.42 mmol, 62%. ^1H NMR (400 MHz, $CDCl_3$, 298 K) δ: 7.99–7.91 (m, 4H, Ar–CH), 7.84–7.75 (m, 4H, Ar–CH); ^{13}C NMR (101 MHz, $CDCl_3$, 298 K) δ: 166.2, 135.3, 131.6, 124.8; IR ν_{max} (cm^{-1}): 2245, 1752, 1239, 1028, 905, 717; (ES+) $[M+H]^+$ [$C_{16}H_9N_2O_4S$]: calculated 325.0283, found 325.0287; Melting point: 305–310 °C.

2.4 Synthesis and Spectral Characterization of *N³,N³,N³′,N³′*-Tetramethyl-[1,1'-biphenyl]-3,3'-Diamine, 5a[2]

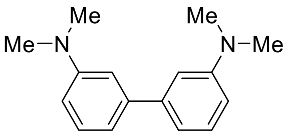

Synthesized in accordance with *General Procedure b* using of 3-bromo-*N*,*N*-dimethylaniline (1.44 mL, 10 mmol, 1.0 equiv.). The crude product was purified via preparative thin-layer chromatography using hexane/ethyl acetate as eluent (95:5). The desired compound **5a** was obtained as a yellow liquid. Yield: 0.671 g, 2.79 mmol, 56%. ^1H NMR (400 MHz, $CDCl_3$, 298 K) δ: 7.40–7.30 (m, 2H, Ar–CH), 7.05–6.95 (m, 4H, Ar–CH), 6.83–6.74 (m, 2H, Ar–CH), 3.04 (s, 12H, Me); ^{13}C NMR (101 MHz, $CDCl_3$, 298 K) δ: 151.0, 143.5, 129.4, 116.3, 112.1, 111.7, 40.9 (Me).

2.4 Synthesis and Sectral Characterization of Di(hetero)arylthioethers, 3a

Synthesis of 4,4'-thiobis(N,N-dimethylaniline), **3aa**[3]

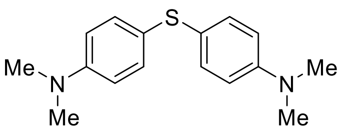

Synthesized in accordance with *General Procedure c* using *N*,*N*-dimethylaniline (28 µL, 0.22 mmol, 2.2 equiv.). The crude product was purified via preparative thin-layer chromatography using hexane/ethyl acetate as eluent (80:20). The desired compound **3aa** was obtained as a yellow solid. Yield: 23 mg, 0.08 mmol, 84%. ^1H NMR (400 MHz, $CDCl_3$, 298 K) δ: 7.20–7.14 (m, 4H, Ar–CH), 6.61–6.53 (m,

4H, Ar–CH), 2.85 (s, 12H, Me); ^{13}C NMR (101 MHz, $CDCl_3$, 298 K) δ: 149.8, 132.7, 123.1, 113.3, 40.7 (Me); IR ν_{max} (cm^{-1}): 2889, 2810, 1593, 1504, 1443, 1358, 1225, 1192, 808; HRMS (EI) [M] [$C_{16}H_{20}N_2S$]: calculated 272.13417, found 272.1340; Melting point: 100–105 °C. A

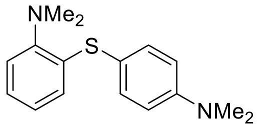

very minor amount of ortho/para mixed product 2-((4-(dimethylamino)phenyl)thio)-*N,N*-dimethylaniline (**3aa'**) was also isolated as a white solid. Yield: 2 mg, 0.007 mmol, 7%. ^{1}H NMR (400 MHz, $CDCl_3$) δ: 7.42–7.40 (m, 2H), 7.08–7.03 (m, 2H), 6.87–6.83 (m, 1H), 6.75–6.73 (m, 2H), 6.65 (dd, J = 7.9, 1.2 Hz, 1H), 3.01 (s, 6H, Me), 2.82 (s, 6H, Me); ^{13}C NMR (101 MHz, $CDCl_3$) δ: 150.9, 150.0, 137.4, 136.9, 126.2, 125.0, 123.9, 119.1, 116.7, 113.2, 44.6 (Me), 40.4 (Me); HRMS (ES+) $[M+H]^+$ [$C_{16}H_{21}N_2S$]: calculated 273.1425, found 273.1432.

Synthesis of 4,4'-thiobis(N,N-diethylaniline), **3ab**

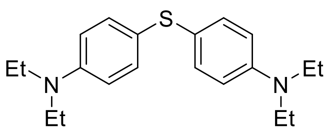

Synthesized in accordance with *General Procedure c* using *N,N*-diethylaniline (36 µL, 0.22 mmol, 2.2 equiv.). The crude product was purified via preparative thin-layer chromatography using hexane/ethyl acetate as eluent (90:10). The desired compound **3ab** was obtained as a yellow oil. Yield: 32 mg, 0.1 mmol, 97%. ^{1}H NMR (400 MHz, $CDCl_3$, 298 K) δ: 7.27–7.21 (m, 4H, Ar–CH), 6.63–6.56 (m, 4H, Ar–CH), 3.33 (q, J = 7.0 Hz, 8H, CH_2), 1.15 (t, J = 7.1 Hz, 12H, Me). ^{13}C NMR (101 MHz, $CDCl_3$, 298 K) δ: 147.0, 133.0, 121.8, 112.5, 44.5 (CH_2), 12.7 (Me); IR ν_{max} (cm^{-1}): 2967, 2926, 2360, 2342, 1589, 1497, 1396, 1373, 1263, 1192, 1076, 806; HRMS (APCI) $[M+H]^+$ $[C_{20}H_{29}N_2S]^+$: calculated 329.2051, found 329.2053.

Synthesis of 5,5''-thiobis(N,N-dimethyl-[1,1'-biphenyl]-2-amine), **3ac**

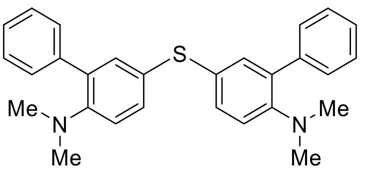

Synthesized in accordance with *General Procedure c* using *N,N*-dimethyl-[1,1'-biphenyl]-2-amine (43 mg, 0.22 mmol, 2.2 equiv.). The crude product was purified via preparative thin-layer chromatography using hexane/ethyl acetate as eluent (85:15). The desired compound **3ac**

was obtained as a colorless oil. Yield: 16 mg, 0.04 mmol, 38%. ^{1}H NMR (400 MHz, $CDCl_3$, 298 K) δ: 7.57–7.50 (m, 4H, Ar–CH), 7.40–7.35 (m, 4H, Ar–CH), 7.31–7.23 (m, 6H, Ar–CH), 6.94 (d, *J* = 8.4 Hz, 2H, Ar–CH), 2.53 (s, 12H, Me); ^{13}C NMR (101 MHz, $CDCl_3$, 298 K) δ: 150.6, 141.5, 134.8, 134.6, 131.2, 128.7, 128.5, 127.8, 126.8, 118.4, 43.4 (Me); IR ν_{max} (cm^{-1}): 2936, 2781, 2342, 1585, 1483, 1383, 1161, 945, 818, 698; HRMS (ES+) $[M+H]^+$ $[C_{28}H_{29}N_2S]^+$: calculated 425.2051, found 425.2049.

Synthesis of bis(4-(pyrrolidin-1-yl)phenyl)sulfane, **3ad**

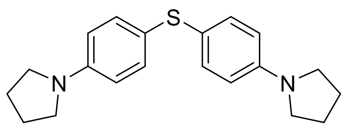

Synthesized in accordance with *General Procedure c* using 1-phenylpyrrolidine (32 μL, 0.22 mmol, 2.2 equiv.). The crude product was purified via preparative thin-layer chromatography using hexane/ethyl acetate as eluent (90:10). The desired compound **3ad** was obtained as a yellow solid. Yield: 25 mg, 0.08 mmol, 77%. ^{1}H NMR (400 MHz, $CDCl_3$, 298 K) δ: 7.27–7.21 (m, 4H, Ar–CH), 6.50–6.44 (m, 4H, Ar–CH), 3.28–3.20 (m, 8H, CH_2), 2.02–1.94 (m, 8H, CH_2); ^{13}C NMR (101 MHz, $CDCl_3$, 298 K) δ: 147.1, 133.0, 122.0, 112.3, 47.7 (CH_2), 25.6 (CH_2); IR ν_{max} (cm^{-1}): 2967, 1601, 1551, 1458, 1188, 961, 802; HRMS (ES+) $[M+H]^+$ $[C_{20}H_{25}N_2S]^+$: calculated 325.1738, found 325.1737; Melting point: 130–133 °C.

Synthesis of 4,4'-thiobis(N-ethyl-N-methylaniline), **3ae**

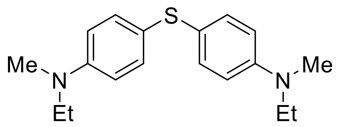

Synthesized in accordance with *General Procedure c* using *N*-ethyl-*N*-methylaniline (32 μL, 0.22 mmol, 2.2 equiv.). The crude product was purified via preparative thin-layer chromatography using hexane/ethyl acetate as eluent (90:10). The desired compound **3ae** was obtained as a yellow oil. Yield: 22 mg, 0.07 mmol, 73%. ^{1}H NMR (400 MHz, $CDCl_3$, 298 K) δ: 7.27–7.21 (m, 4H, Ar–CH), 6.65–6.61 (m, 4H, Ar–CH), 3.37 (q, *J* = 7.1 Hz, 4H, CH_2), 2.89 (s, 6H, Me), 1.11 (t, *J* = 7.1 Hz, 6H, Me); ^{13}C NMR (101 MHz, $CDCl_3$, 298 K) δ: 148.3, 134.6, 132.9, 122.5,

113.0, 46.9 (CH_2), 37.6 (Me), 11.4 (Me); IR ν_{max} (cm^{-1}): 2968, 1818, 1591, 1371, 1267, 1082, 986, 808; HRMS (ES+) $[M+H]^+$ $[C_{18}H_{25}N_2S]^+$: calculated 301.1738, found 301.1747.

Synthesis of 4,4'-thiobis(N-benzyl-N-methylaniline), **3af**

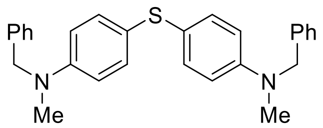

Synthesized in accordance with *General Procedure c* using *N*-benzyl-*N*-methylaniline (41μL, 0.22 mmol, 2.2 equiv.). The crude product was purified via preparative thin-layer chromatography using a hexane/ethyl acetate eluent (90:10). The desired compound **3af** was obtained as a yellow solid. Yield: 26 mg, 0.06 mmol, 61%. ^{1}H NMR (400 MHz, $CDCl_3$, 298 K) δ: 7.39–7.33 (m, 4H, Ar–CH), 7.32–7.22 (m, 10H, Ar–CH), 6.73–6.68 (m, 4H, Ar–CH), 4.55 (s, 4H, benzyl), 3.05 (s, 6H, Me); ^{13}C NMR (101 MHz, $CDCl_3$, 298 K) δ: 149.0, 138.8, 132.9, 128.7, 127.1, 126.8, 123.0, 113.0, 56.7 (benzyl), 38.7 (Me); IR ν_{max} (cm^{-1}): 2922, 2342, 1593, 1452, 1352, 1192, 945, 808; HRMS (ES+) $[M+H]^+$ $[C_{28}H_{29}N_2S]^+$: calculated 425.2051, found 425.2035.

Synthesis of 4,4'-thiobis(2-bromo-N,N-dimethylaniline), **3ag**

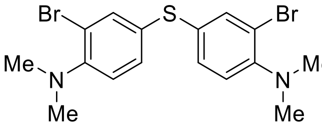

Synthesized in accordance with *General Procedure c* using 2-bromo-*N*,*N*-dimethylaniline (32 μL, 0.22 mmol, 2.2 equiv.) The crude product was purified via preparative thin-layer chromatography using a hexane/ethyl acetate eluent (90:10). The desired compound **3ag** was obtained as a yellow oil. Yield: 12 mg, 0.03 mmol, 28%. ^{1}H NMR (400 MHz, $CDCl_3$, 298 K) δ: 7.54 (d, J = 2.1 Hz, 2H, Ar–CH), 7.22 (dd, J = 8.4, 2.3 Hz, 2H, Ar–CH), 7.00 (d, J = 8.4 Hz, 2H, Ar–CH), 2.80 (s, 12H, Me); ^{13}C NMR (101 MHz, $CDCl_3$, 298 K) δ: 151.4, 136.5, 131.3, 130.2, 121.0, 119.4, 44.2 (Me); IR ν_{max} (cm^{-1}): 2941, 2781, 1483, 1323, 1163, 945; HRMS (EI) [M] $[C_{16}H_{18}N_2{}^{79}Br_2{}^{32}S]$: calculated 427.9552, found 427.9544.

Synthesis of 4,4'-thiobis(N,N-dibenzylaniline), **3ah**

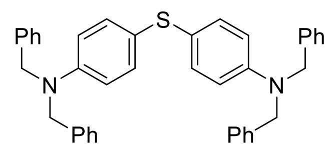

Synthesized in accordance with *General Procedure c* using *N,N*-dibenzylaniline (60 mg, 0.22 mmol, 2.2 equiv.). The crude product was purified via preparative thin layer chromatography using hexane/ethyl acetate as eluent (80:20). The desired compound **3ah** was obtained as a yellow oil. Yield: 38 mg, 0.07 mmol, 66%. ^{1}H NMR (400 MHz, $CDCl_3$, 298 K) δ; 7.37–7.31 (m, 8H, Ar–CH), 7.30–7.21 (m, 12H, Ar–CH), 7.21–7.15 (m, 4H, Ar–CH), 6.66 (d, J = 9.0 Hz, 4H), 4.64 (s, 8H, benzyl); ^{13}C NMR (101 MHz, $CDCl_3$, 298 K) δ: 148.5, 138.4, 132.9, 128.8, 127.1, 126.7, 123.1, 113.2, 54.4 (benzyl); IR ν_{max} (cm^{-1}): 3028, 2858, 2359, 1593, 1495, 1360, 1194, 1163, 808, 664; HRMS ($APCI^{+}$) $[M+H]^{+}$ $[C_{40}H_{37}N_2S]^{+}$: calculated 577.2677, found 577.2680.

Synthesis of bis(4-methoxyphenyl)sulfane, **3ai**[4]

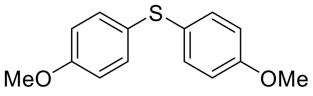

Synthesized in accordance with *General Procedure c* using methoxybenzene (24 µL, 0.22 mmol, 2.2 equiv.). The crude product was purified via preparative thin layer chromatography using hexane/ethyl acetate as eluent 80:20. The desired compound **3ai** was obtained as a colorless oil. Yield: 19 mg, 0.08 mmol, 78%. ^{1}H NMR (400 MHz, $CDCl_3$, 298 K) δ: 7.31–7.24 (m, 4H, Ar–CH), 6.87–6.80 (m, 4H, Ar–CH), 3.79 (s, 6H, Me); ^{13}C NMR (101 MHz, $CDCl_3$, 298 K) δ: 159.1, 132.9, 127.6, 114.9, 55.5 (Me).

Synthesis of bis(4-ethoxyphenyl)sulfane, **3aj**[5]

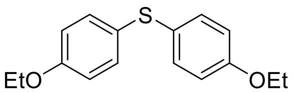

Synthesized in accordance with *General Procedure c* using ethoxybenzene (28 µL, 0.22 mmol, 2.2 equiv.). The crude product was purified via preparative thin layer chromatography using hexane/ethyl acetate as eluent (80:20). The desired compound **3aj** was obtained as a yellow oil. Yield: 16 mg, 0.06 mmol, 58%. ^{1}H NMR (400 MHz, $CDCl_3$, 298 K) δ: 7.24–7.13 (m, 4H, Ar–CH), 6.77–6.72 (m, 4H, Ar–CH),

3.93 (q, *J* = 7.0 Hz, 4H, CH_2), 1.32 (t, *J* = 7.0 Hz, 6H, Me); ^{13}C NMR (101 MHz, $CDCl_3$, 298 K) δ: 158.5, 132.8, 127.4, 115.4, 63.7, 14.9.

Synthesis of bis(4-isopropoxyphenyl)sulfane, **3ak**

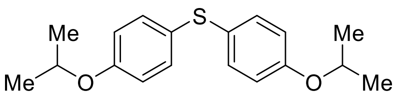

Synthesized in accordance with *General Procedure c* using isopropoxybenzene (32 µL, 0.22 mmol, 2.2 equiv.). The crude product was purified via preparative thin-layer chromatography using hexane/ethyl acetate as eluent (90:10). The desired compound **3ak** was obtained as a colorless oil. Yield: 20 mg, 0.07 mmol, 66%. ^{1}H NMR (400 MHz, $CDCl_3$, 298 K) δ: 7.29–7.23 (m, 4H, Ar–CH), 6.84–6.78 (m, 4H, Ar–CH), 4.51 (p, *J* = 6.1 Hz, 2H, CH), 1.32 (d, *J* = 6.0 Hz, 12H, Me); ^{13}C NMR (101 MHz, $CDCl_3$, 298 K) δ: 157.4, 132.8, 127.2, 116.7, 70.2 (CH), 22.2 (Me); IR ν_{max} (cm^{-1}): 2976, 1591, 1487, 1238, 1117, 953, 826; HRMS (EI) [M] [$C_{18}H_{22}O_2S$]: calculated 302.1335, found 302.1331.

Synthesis of bis(4-(cyclopentyloxy)phenyl)sulfane, **3al**

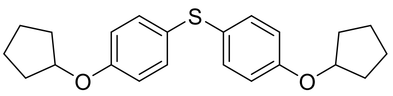

Synthesized in accordance with *General Procedure c* using (cyclopentyloxy)benzene (36 µL, 0.22 mmol, 2.2 equiv.). The crude product was purified via preparative thin-layer chromatography using hexane/ethyl acetate as eluent (90:10). The desired compound **3al** was obtained as a colorless oil. Yield: 24 mg, 0.07 mmol, 68%. ^{1}H NMR (400 MHz, $CDCl_3$, 298 K) δ: 7.32–7.18 (m, 4H, Ar–CH), 6.82–6.78 (m, 4H, Ar–CH), 4.75–4.68 (m, 2H, CH), 1.94–1.83 (m, 8H, CH_2), 1.82–1.76 (m, 4H, CH_2), 1.67–1.58 (m, 4H, CH_2); ^{13}C NMR (101 MHz, $CDCl_3$, 298 K) δ: 157.6, 132.8, 127.0, 116.4, 79.5 (CH), 33.0 (CH_2), 24.2 (CH_2); IR ν_{max} (cm^{-1}): 2957, 1591, 1236, 1166, 983, 822; HRMS (ES+) $[M+H]^+$ $[C_{22}H_{27}O_2S]^+$: calculated 355.1732, found 355.1732.

Synthesis of bis(2,4-dimethoxyphenyl)sulfane, **3am**

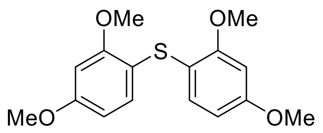

Synthesized in accordance with *General Procedure c* using 1,3-dimethoxybenzene (28 µL, 0.22 mmol, 2.2 equiv.). The crude product was purified via preparative thin-layer chromatography using hexane/ethyl acetate as eluent (70:30). The desired compound **3am** was obtained as a colorless liquid. Yield: 19 mg, 0.06 mmol, 62%. ^{1}H NMR (400 MHz, $CDCl_3$, 298 K) δ: 7.00 (d, J = 8.5 Hz, 2H, Ar–CH), 6.49 (d, J = 2.5 Hz, 2H, Ar–CH), 6.42 (dd, J = 8.5, 2.5 Hz, 2H, Ar–CH), 3.83 (s, 6H, OMe), 3.79 (s, 6H, OMe); ^{13}C NMR (101 MHz, $CDCl_3$, 298 K) δ: 160.6, 159.1, 133.0, 114.5, 105.3, 99.1, 56.0 (OMe), 55.6 (OMe); IR ν_{max} (cm^{-1}): 3001, 2359, 1595, 1462, 1300, 1250, 1161, 1030, 829; HRMS (EI) [M] [$C_{16}H_{18}O_4S$]: calculated 306.09203, found 306.0927.

Synthesis of bis(2,4-diethoxyphenyl)sulfane, **3an**

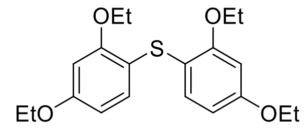

Synthesized in accordance with *General Procedure c* using 1,3-diethoxybenzene (38 µL, 0.22 mmol, 2.2 equiv.). The crude product was purified via preparative thin layer chromatography using hexane/ethyl acetate as eluent (80:20). The desired compound **3an** was obtained as a colorless oil. Yield: 16 mg, 0.04 mmol, 44%. ^{1}H NMR (400 MHz, $CDCl_3$, 298 K) δ: 7.05 (d, J = 8.4 Hz, 2H, Ar–CH), 6.45 (d, J = 2.5 Hz, 2H, Ar–CH), 6.38 (dd, J = 8.5, 2.5 Hz, 2H, Ar–CH), 4.08–3.95 (m, 8H, CH_2), 1.40 (t, J = 7.0 Hz, 6H, Me), 1.35 (t, J = 7.0 Hz, 6H, Me); ^{13}C NMR (101 MHz, $CDCl_3$, 298 K) δ: 159.8, 158.5, 133.3, 115.1, 105.8, 100.6, 64.4 (CH_2) 63.8 (CH_2), 15.0 (Me), 14.8 (Me); IR ν_{max} (cm^{-1}): 2978, 2363, 1740, 1572, 1390, 1298, 1248, 1151, 1034, 914, 818; HRMS (EI) [M] [$C_{20}H_{26}O_4S$]: calculated 362.15463, found 362.1539.

Synthesis of bis(2,3,4-trimethoxyphenyl)sulfane, **3ao**

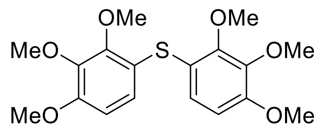

Synthesized in accordance with *General Procedure c* using 1,2,3-trimethoxybenzene (37 mg, 0.22 mmol, 2.2 equiv.). The crude product was purified via preparative thin-layer chromatography

using hexane/ethyl acetate as eluent (80:20). The desired compound **3ao** was obtained as a colorless oil. Yield: 22 mg, 0.06 mmol, 60%. ^{1}H NMR (400 MHz, $CDCl_3$, 298 K) δ: 6.80 (d, *J* = 8.6 Hz, 2H, Ar–CH), 6.60 (d, *J* = 8.8 Hz, 2H, Ar–CH), 3.88 (s, 12H, OMe), 3.83 (s, 6H, OMe); ^{13}C NMR (101 MHz, $CDCl_3$, 298 K) δ: 153.5, 152.8, 143.0, 126.8, 121.2, 108.1, 61.13 (OMe), 61.07 (OMe), 56.2 (OMe); IR ν_{max} (cm^{-1}): 2936, 2359, 1578, 1456, 1404, 1288, 1219, 1144, 1088, 920, 795, 694; HRMS (EI+) $[M+H]^+$ $[C_{18}H_{23}O_6S]^+$: calculated 367.1215, found 367.1216.

Synthesis of bis(2,4,6-trimethoxyphenyl)sulfane, **3ap**[6]

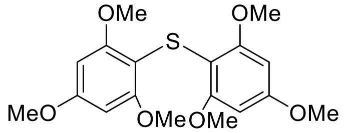

Synthesized in accordance with *General Procedure c* using 1,3,5-trimethoxybenzene (37 mg, 0.22 mmol, 2.2 equiv.). The crude product was purified via preparative thin layer chromatography using hexane/ethyl acetate as eluent (70:30). The desired compound **3ap** was obtained as a yellow oil. Yield: 17 mg, 0.05 mmol, 47%. ^{1}H NMR (400 MHz, $CDCl_3$, 298 K) δ: 6.06 (s, 4H, Ar–CH), 3.77 (s, 6H, OMe), 3.74 (s, 12H, OMe); ^{13}C NMR (101 MHz, $CDCl_3$, 298 K) δ: 161.3, 160.7, 91.2, 90.8, 56.3 (OMe), 55.4 (OMe); IR ν_{max} (cm^{-1}): 2999, 1738, 1454, 1408, 1366, 1227, 1159, 1123, 742; HRMS (EI) [M] $[C_{18}H_{22}O_6S]$: calculated 366.1137, found 366.1140.

Synthesis of bis(4-(methylthio)phenyl)sulfane, **3aq**

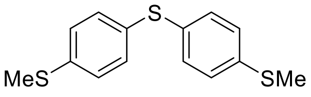

Synthesized in accordance with *General Procedure c* using methyl(phenyl)sulfane (26 µL, 0.22 mmol, 2.2 equiv.). The crude product was purified via preparative thin layer chromatography using hexane as eluent. The desired compound **3aq** was obtained as a yellow solid. Yield: 20 mg, 0.07 mmol, 72%. ^{1}H NMR (400 MHz, $CDCl_3$, 298 K) δ: 7.27–7.22 (m, 4H, Ar–CH), 7.21–7.15 (m, 4H, Ar–CH), 2.47 (s, 6H, Me); ^{13}C NMR (101 MHz, $CDCl_3$, 298 K) δ: 138.0, 132.4, 131.6, 127.4, 16.0 (Me); IR ν_{max} (cm^{-1}): 2918, 1576, 1435, 1389, 1260, 1099, 905, 802, 729; HRMS (EI) [M] $[C_{14}H_{14}S^3]$: calculated 278.02521, found 278.0247; Melting point: 130–131 °C.

Synthesis of bis(4-(methylthio)phenyl)sulfane, **3ar**

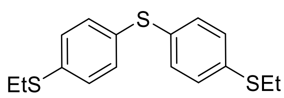

Synthesized in accordance with *General Procedure c* using ethyl(phenyl)sulfane (30 μL, 0.22 mmol, 2.2 equiv.). The crude product was purified via preparative thin layer chromatography using hexane as eluent. The desired compound **3ar** was obtained as a colorless oil. Yield: 18 mg, 0.06 mmol, 59%. ^{1}H NMR (400 MHz, $CDCl_3$, 298 K) δ: 7.28–7.20 (m, 8H), 2.93 (q, J = 7.4 Hz, 4H), 1.32 (t, J = 7.4 Hz, 6H); ^{13}C NMR (101 MHz, $CDCl_3$, 298 K) δ: 136.2, 133.0, 131.6, 129.6, 27.7, 14.4. IR ν_{max} (cm^{-1}): 2974, 2928, 1554, 1466, 1437, 1869, 1251, 1089, 1001, 810; HRMS (ES+) $[M+H]^+$ $[C_{16}H_{19}S_3]^+$: calculated 307.0649, found 307.0648.

Synthesis of bis(4-(isopropylthio)phenyl)sulfane, **3as**

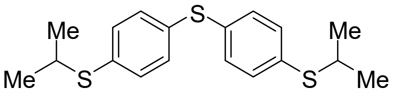

Synthesized in accordance with *General Procedure c* using isopropyl(phenyl)sulfane (35 μL, 0.22 mmol, 2.2 equiv.). The crude product was purified via preparative thin layer chromatography using hexane as eluent. The desired compound **3as** was obtained as a colorless oil. Yield: 16 mg, 0.05 mmol, 48%. ^{1}H NMR (400 MHz, $CDCl_3$, 298 K) δ: 7.34–7.29 (m, 4H, Ar–CH), 7.28–7.23 (m, 4H, Ar–CH), 3.38 (p, J = 6.7 Hz, 2H, CH), 1.31 (d, J = 6.6 Hz, 12H, Me); ^{13}C NMR (101 MHz, $CDCl_3$, 298 K) δ: 135.1, 133.9, 132.3, 131.4, 38.3 (CH), 23.2 (Me); IR ν_{max} (cm^{-1}): 2959, 2361, 1574, 1474, 1366, 1240, 1099, 812, 743; HRMS (EI+) $[M+H]^+$ $[C_{18}H_{23}S_3]^+$: calculated 335.0962, found 335.0965.

Synthesis of bis(4-(cyclopropylthio)phenyl)sulfane, **3at**

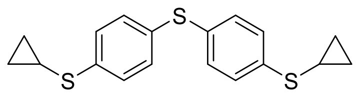

Synthesized in accordance with *General Procedure c* using cyclopropyl(phenyl)sulfane (31 μL, 0.22 mmol, 2.2 equiv.). The crude product was purified via preparative thin-layer chromatography using hexane/ethyl acetate as eluent (98.5:1.5). The desired compound **3at** was obtained as a colorless oil. Yield: 15 mg, 0.05 mmol, 45%. ^{1}H NMR (400 MHz, $CDCl_3$, 298 K) δ: 7.26–7.14 (m, 8H, Ar–CH),

2.14–2.04 (m, 2H, CH), 1.03–0.96 (m, 4H, CH_2), 0.65–0.58 (m, 4H, CH_2); ^{13}C NMR (101 MHz, $CDCl_3$, 298 K) δ: 138.4, 132.3, 131.6, 127.3, 12.1 (CH), 8.7 (CH_2); IR ν_{max} (cm^{-1}): 3082, 2924, 2361, 1574, 1389, 1099, 806; HRMS (EI+) $[M+H]^+$ $[C_{18}H_{19}S_3]^+$: calculated 331.0649, found 331.0646.

Synthesis of bis(1,2,5-trimethyl-1H-pyrrol-3-yl)sulfane, **3au**

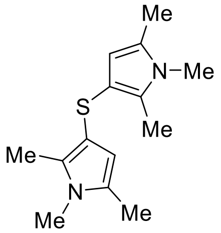

Synthesized in accordance with *General Procedure c* using 1,2,5-trimethyl-1*H*-pyrrole (46 µL, 0.22 mmol, 2.2 equiv.). The crude product was purified via preparative thin-layer chromatography using hexane/ethyl acetate as eluent (90:10). The desired compound **3au** was obtained as a colorless oil. Yield: 16 mg, 0.06 mmol, 66%. ^{1}H NMR (400 MHz, $CDCl_3$, 298 K) δ: 5.89 (s, 2H, Ar–CH), 3.33 (d, J = 1.5 Hz, 6H, Me), 2.34 (s, 6H, Me), 2.13 (s, 6H, Me); ^{13}C NMR (101 MHz, $CDCl_3$, 298 K) δ: 130.1, 127.4, 110.8, 110.1, 30.8 (Me), 12.5 (Me), 10.8 (Me); IR ν_{max} (cm^{-1}): 2922, 2363, 1697, 1684, 1636, 1541, 1458, 1398, 912, 740; HRMS (EI) [M] $[C_{14}H_{20}N_2S]$: calculated 248.1347, found 248.1354.

Synthesis of bis(1,2-dimethyl-1H-indol-3-yl)sulfane, **3av**[7]

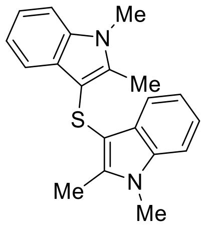

Synthesized in accordance with *General Procedure c* using 1,2-dimethyl-1*H*-indole (32 mg, 0.22 mmol, 2.2 equiv.). The crude product was purified via preparative thin layer chromatography using hexane/ethyl acetate as eluent (75:25). The desired compound **3av** was obtained as a yellow oil. Yield: 7 mg, 0.02 mmol, 22%. ^{1}H NMR (400 MHz, $CDCl_3$, 298 K) δ: 7.83–7.76 (m, 2H, Ar–CH), 7.21–7.18 (m, 2H, Ar–CH), 7.16–7.12 (m, 2H, Ar–CH), 7.12–7.08 (m, 2H, Ar–CH), 3.62 (s, 6H, Me), 2.70 (s, 6H, Me); ^{13}C NMR (101 MHz, $CDCl_3$, 298 K) δ: 140.2, 136.7, 129.8, 121.2, 119.8, 119.2, 108.8, 104.0, 30.1 (Me), 11.5 (Me).

Synthesis of bis(9-methyl-9H-carbazol-3-yl)sulfane, **3aw**

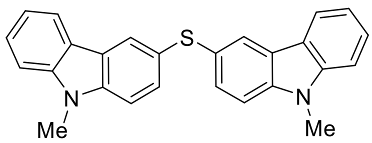

Synthesized in accordance with *General Procedure c* using 9-methyl-9*H*-carbazole (40 mg, 0.22 mmol, 2.2 equiv.). The crude product was purified via preparative thin-layer chromatography using hexane/ethyl acetate as eluent (80:20). The desired compound **3aw** was obtained as a yellow solid. Yield: 16 mg, 0.04 mmol, 41%. ^{1}H NMR (400 MHz, $CDCl_3$, 298 K) δ: 8.19 (dd, *J* = 1.8, 0.7 Hz, 2H, Ar–CH), 8.02 (dt, *J* = 7.8, 1.0 Hz, 2H, Ar–CH), 7.54 (dd, *J* = 8.5, 1.8 Hz, 2H, Ar–CH), 7.50–7.45 (m, 2H, Ar–CH), 7.39 (dt, *J* = 8.3, 1.0 Hz, 2H, Ar–CH), 7.33 (d, *J* = 8.5 Hz, 2H, Ar–CH), 7.23–7.18 (m, 2H, Ar–CH), 3.84 (s, 6H, Me); ^{13}C NMR (101 MHz, $CDCl_3$, 298 K) δ: 141.4, 140.4, 129.7, 126.6, 126.2, 123.9, 123.8, 122.4, 120.7, 119.3, 109.3, 108.7, 29.3 (Me); IR ν_{max} (cm^{-1}): 2924, 2853, 1591, 1476, 1267, 798, 745; HRMS (EI) [M][$C_{26}H_{20}N_2S$]: calculated 392.1347, found 392.1349.

Synthesis of bis(2-phenylimidazo[1,2-a]pyridin-3-yl)sulfane, **3ax**

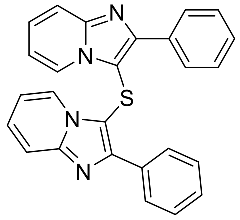

Synthesized in accordance with *General Procedure c* using 2-phenylimidazo[1,2-*a*]pyridine (43 mg, 0.22 mmol, 2.2 equiv.). The crude product was purified via preparative thin layer chromatography using hexane/ethyl acetate as eluent (30:70). The desired compound **3ax** was obtained as a yellow solid. Yield: 20 mg, 0.05 mmol, 48%. ^{1}H NMR (400 MHz, $CDCl_3$, 298 K) δ: 8.18–8.05 (m, 4H, Ar–CH), 7.66–7.59 (m, 4H, Ar–CH), 7.59–7.52 (m, 4H, Ar–CH), 7.50 (dt, *J* = 9.0, 1.1 Hz, 2H, Ar–CH), 7.10 (ddd, *J* = 9.0, 6.8, 1.3 Hz, 2H, Ar–CH), 6.35 (td, *J* = 6.9, 1.3 Hz, 2H, Ar–CH); ^{13}C NMR (101 MHz, $CDCl_3$, 298 K) δ: 151.0, 146.7, 133.9, 129.7, 129.1, 128.7, 126.5, 125.5, 117.5, 112.7, 107.6; IR ν_{max} (cm^{-1}): 2924, 2853, 1634, 1497, 1342, 1233, 756, 696; HRMS (EI+) $[M+H]^+$ $[C_{26}H_{19}N_4S]^+$: calculated 419.1330, found 419.1334.

Synthesis of N^2,N^2,N^8,N^8-tetramethyldibenzo[b,d]thiophene-2,8-diamine, **6aa**

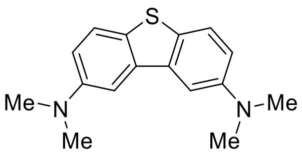

Synthesized in accordance with *General Procedure k* using N^3,N^3,$N^{3'}$,$N^{3'}$-tetramethyl-[1,1'-biphenyl]-3,3'-diamine **5a** (29 mg, 0.12 mmol, 1.2 equiv.). The crude product was purified via preparative thin-layer chromatography using hexane/ethyl acetate as eluent (70:30). The desired compound **5.3y** was obtained as a yellow oil. Yield: 10 mg, 0.04 mmol, 37%. ^{1}H NMR (400 MHz, $CDCl_3$, 298 K) δ: 7.64 (d, J = 8.8 Hz, 2H, Ar–CH), 7.39 (d, J = 2.5 Hz, 2H, Ar–CH), 6.99 (dd, J = 8.8, 2.6 Hz, 2H, Ar–CH), 3.06 (s, 12H, Me); ^{13}C NMR (101 MHz, $CDCl_3$, 298 K) δ: 148.8, 136.8, 129.3, 123.3, 114.7, 105.0, 41.7 (Me); IR ν_{max} (cm^{-1}): 2924, 2363, 1601, 1547, 1491, 1350, 1219, 798; HRMS (ES+) $[M+H]^+$ $[C_{16}H_{19}N_2S]^+$: calculated 271.1269, found 271.1276.

3. Control Experiments

Scale up synthesis of 3ab and evidence for an atom-economical process**:** Inside the glovebox, a 20 mL sealed tube equipped with a magnetic stir bar was charged with *N*,*N*'-thiobisphthalimide **1a** (640 mg, 2.0 mmol, 1.0 equiv.), $B(C_6F_5)_3$ (216 mg, 0.40 mmol, 20 mol%), and *N*,*N*-diethylaniline (0.71 mL, 4.4 mmol, 2.2 equiv.). The tube was capped with a septum and brought outside the glovebox. Dry 1,2-dichloroethane (10 mL) was then added, and the resulting suspension was stirred at 80 °C until a clear solution was obtained. The reaction mixture was further stirred at this temperature for 16 h, during which a white precipitate reappeared. After completion, all volatiles were removed under reduced pressure, and the crude product was purified by column chromatography on silica gel (hexane/ethyl acetate as eluent) to afford the desired sulfenylated product, yield: 532 mg, 1.62 mmol, 81% yield. The byproduct phthalimide was recovered with a yield of 1.68 mmol, 247 mg, 84%. The regenerated phthalimide can be utilized for the synthesis of subsequent **1a**.

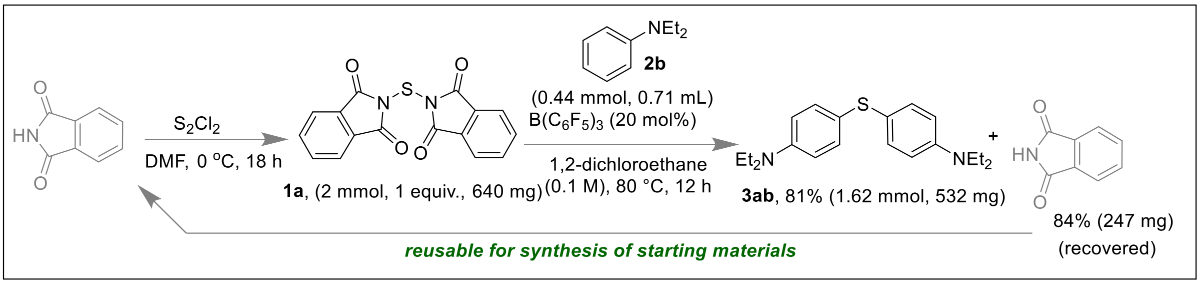

Scheme S4. Scale up synthesis of **3ab** and regeneration of starting material.

***Synthesis of intermediate* 4:** Synthesized in accordance with *General Procedure c*, except the reaction was carried out at room temperature instead of 80 °C, using *N*,*N*-dimethyl-[1,1'-biphenyl]-2-amine (43 mg, 0.22 mmol, 2.2 equiv.). The crude product was purified via preparative thin layer chromatography using hexane/ethyl acetate as eluent (70:30). The desired compound **4** was obtained as a colorless oil. Yield: 15 mg, 0.04 mmol, 40%. ^{1}H NMR (400 MHz, $CDCl_3$, 298 K) δ: 7.90–7.84 (m, 2H, Ar–CH), 7.75–7.69 (m, 3H, Ar–CH), 7.67 (d, J = 2.4 Hz, 1H, Ar–CH), 7.50–7.45 (m, 2H, Ar–CH), 7.40–7.35 (m, 2H, Ar–CH), 7.31–7.27 (m, 1H, Ar–CH), 6.91 (d, J = 8.5 Hz, 1H), 2.54 (s, 6H, Me); ^{13}C NMR (101 MHz, $CDCl_3$, 298 K) δ: 168.1, 153.3, 141.1, 138.7, 135.2, 134.6, 133.6, 132.3, 128.7, 128.5, 127.0, 124.8, 124.0, 117.7, 43.0 (Me); IR ν_{max} (cm^{-1}): 2924, 2851, 2789, 2359, 2342, 1782, 1732, 1705, 1582, 1485, 1339, 1277, 1051, 941, 864, 822, 777, 699; HRMS (ES+) $[M+H]^+$ $[C_{22}H_{19}N_2O_2S]^+$: calculated 375.1167, found 375.1163.

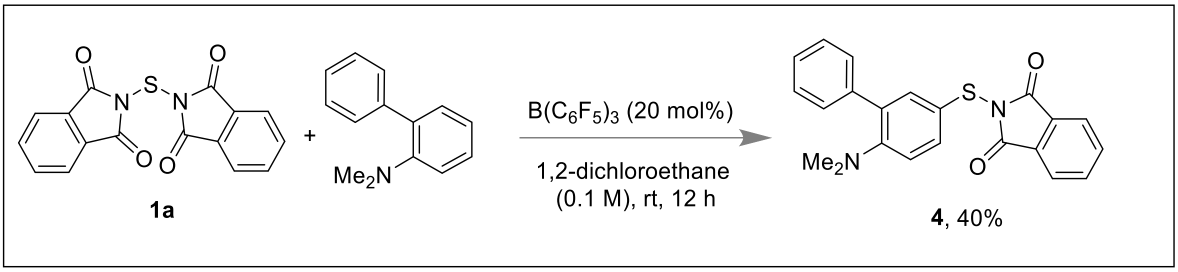

Scheme S5. Control experiment at room temperature to trap the intermediate **4**.

Experiment with S_8 instead of 1a: Inside the glovebox, a glass microwave vial was charged with *N,N*-dimethylaniline (27 mg, 0.22 mmol, 2.2 equiv.), sulfur (S_8, 8.0 mg, 0.10 mmol, 1.0 equiv.), and $B(C_6F_5)_3$ (10.8 mg, 0.02 mmol, 20 mol%), then sealed with a septum. The vial was

brought outside the glovebox, and 1.0 mL of dry 1,2-dichloroethane was added. The suspension was stirred at 80 °C until a homogeneous solution was obtained, after which the reaction was allowed to continue under vigorous stirring at the same temperature for 16 h. The desired product **3aa** was not obtained after the reaction.

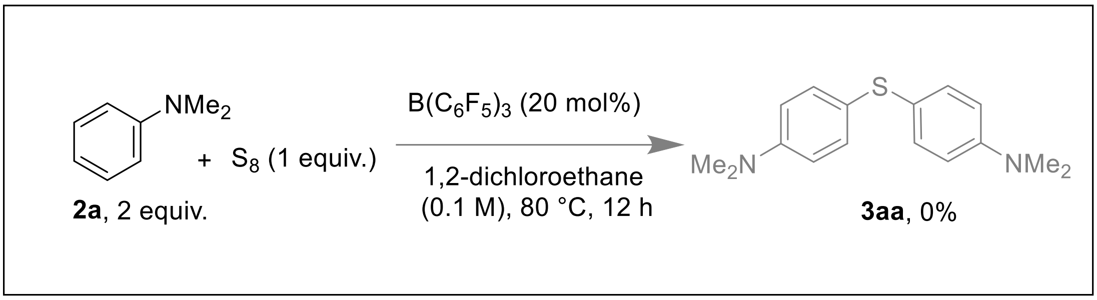

Scheme S6. Experiment with S_8 instead of **1a**.

4. Optoelectronic and CV Studies

***UV-Vis experiments*:** UV experiments were carried out for the solution of **3aa** and **6aa** (2 × 10^{-4} M in $ClCH_2CH_2Cl$. Compound **3aa** does not show any absorption band whereas two absorption bands: a strong band at 331 nm and a weaker one at 378 nm were found for **6aa**.

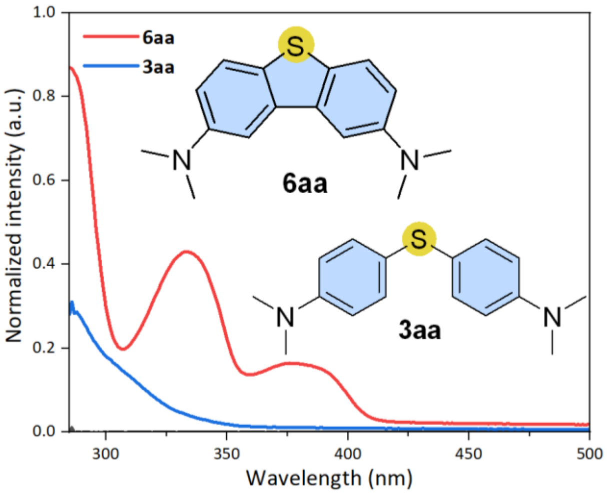

Figure S1. UV spectrum of **3aa** and **6aa**

Cyclic voltammetry: All cyclic voltammetry (CV) studies were recorded using PS trace 5.8 potentiostat with an electrode set-up of GC (glassy carbon) disk (immersed surface area: 0.03 cm^2), Pt wire as counter electrode, 0.01 M $Ag/AgNO_3$ as reference at a scan rate of 100 mV s^{-1}, electrolyte $^n Bu_4NClO_4$ (0.1 M), solvent dichloromethane (DCM), **3aa** (0.005 M), **3ai** (0.005M), **3aq** (0.005 M), **6aa** (0.005 M).

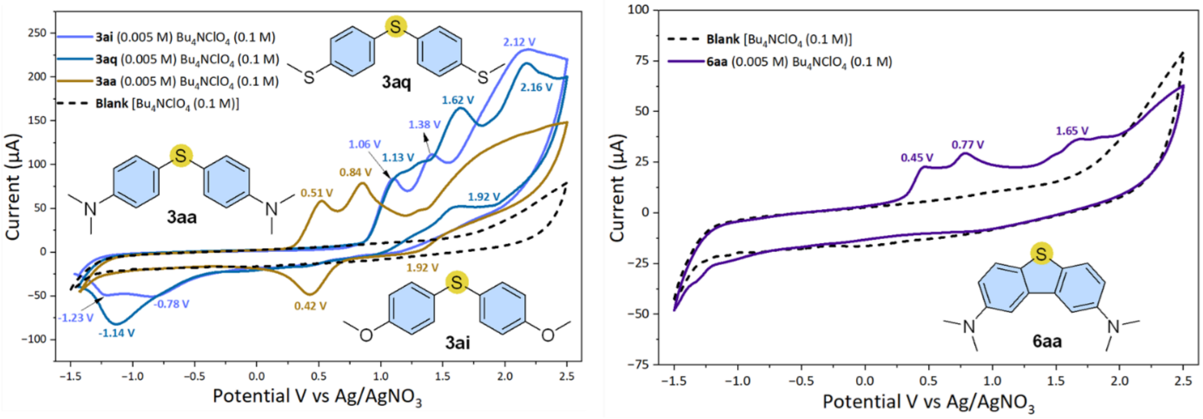

Figure S2. Cyclic voltammetry (CV) plots of **3aa**, **3ai**, **3aq** and **6aa**

5. Unsuccessful Reactions

Other Lewis acids $AlCl_3$, BBr_3, TFOH, TFA, Tf_2NH, $FeCl_3$, $InCl_3$ (20 mol%) were attempted in the reaction with several substrates (Scheme S7, top) and failed. Likewise various Lewis acids were trailed with various electron rich arenes (Scheme S7, bottom).

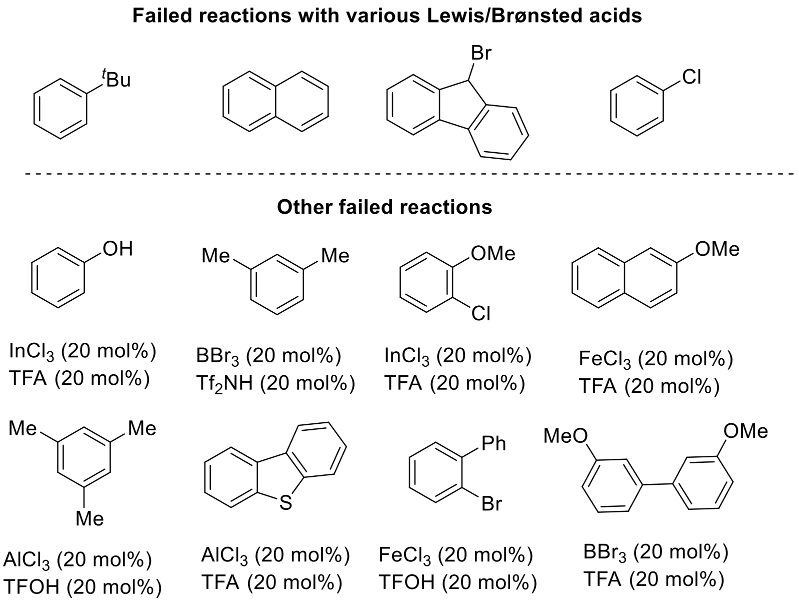

Scheme S7. Unsuccessful arenes and heteroarenes were screened with various Lewis acid (LA) and Brønsted acid (BA) catalysts (20 mol%).

Following *General Procedure a*, the synthesis of several other sulfur transfer reagents were attempted from the corresponding phthalimide. The synthesis of these reagents was unsuccessful (Scheme S8).

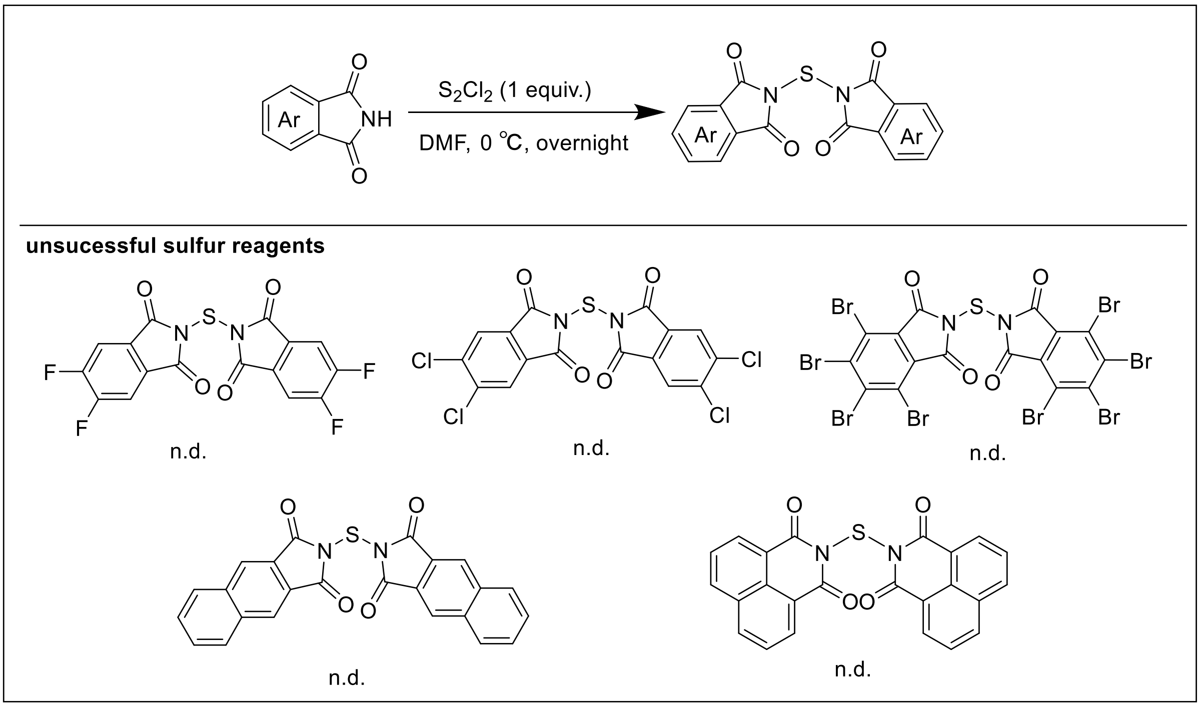

Scheme S8. Unsuccessful synthesis of sulfur precursors.

6. Computational Data

6.1 Computational Details.

All density functional theory (DFT) calculations were performed with the Gaussian 09 program.[8] Geometry optimizations and vibrational frequency calculations were carried out using the M06-2X functional with the 6-31G(d) basis set[9] for all atoms, in combination with the SMD solvation model[10] to describe dichloroethane. Frequency analyses verified each stationary point as either a minimum (no imaginary frequencies) or a transition state (one imaginary frequency). Refined electronic energies were obtained from single-point calculations at the SMD/M06-2X/def2-TZVP level of theory[11] Gibbs free energies were computed by adding zero-point, thermal, and entropic corrections from the M06-2X/6-31G(d) frequency calculations to these single-point energies. Benchmarking across additional density functionals (B3LYP, B3LYP-D3, M06, PBE1PBE, PBE1PBE-D3, and ωB97X-D) was performed exclusively as single-point energy calculations on the M06-2X-optimized geometries, using the same M06-2X frequency-derived corrections. To adjust relative free energies from the gas-phase standard state (1 atm) to the solution-phase standard state (1 M), a correction of 1.89 kcal mol^{-1} was applied.[12] All reported Gibbs free energies are given in kcal mol^{-1}, and all bond lengths in Å. 3D molecular structures were generated with CYLview,[13] and NPA charges were obtained using the NBO module integrated in Gaussian 09.

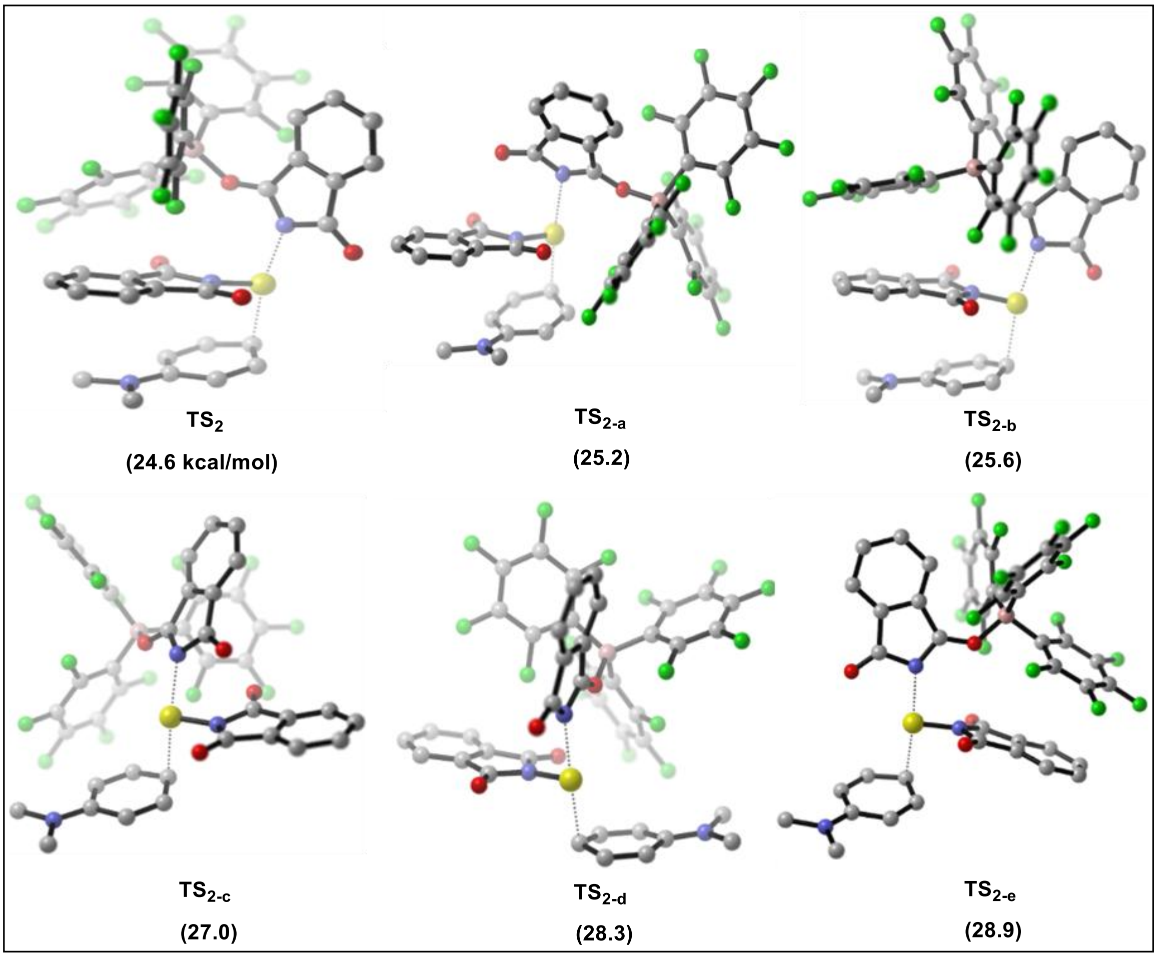

Figure S3. Optimized 3D structures of **TS2** conformers (visualized using CYLview).

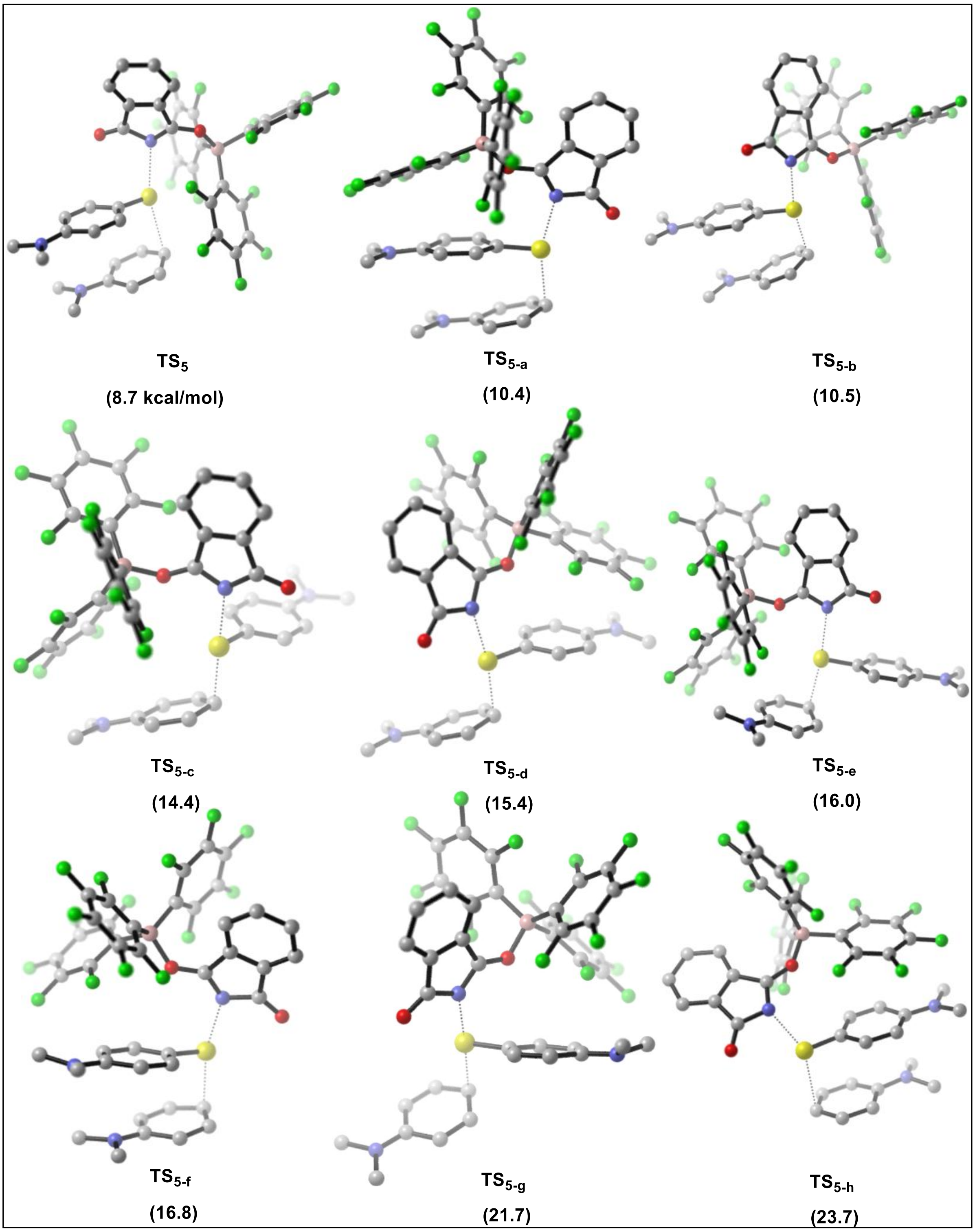

Figure S4. Optimized 3D structures of $\mathbf{TS_5}$ conformers (visualized using CYLview).

Table S1. Benchmark free energies for transition states (**TS_2, $TS_{2\text{-ortho}}$, TS_3** and **$TS_{3\text{-ortho}}$**) and intermediates (**ip_1** and **$ip_{1\text{-ortho}}$**), computed with different density functionals. ΔΔG values represent relative free-energy differences between the *ortho* and *para* pathways: $\Delta\Delta G^{\ddagger}_1 = \Delta G^{\ddagger}(\mathbf{TS_{2\text{-ortho}}}) - \Delta G^{\ddagger}(\mathbf{TS_2})$; $\Delta\Delta G_1 = \Delta G(\mathbf{ip_{1\text{-ortho}}}) - \Delta G(\mathbf{ip_1})$; $\Delta\Delta G^{\ddagger}_2 = \Delta G^{\ddagger}(\mathbf{TS_{3\text{-ortho}}}) - \Delta G^{\ddagger}(\mathbf{TS_3})$. Positive ΔΔG values indicate that the *ortho* pathway is higher in energy and thus *para* is favored; relative free energies are given in kcal mol^{-1}.

Level of Theory	**TS_2**	**$TS_{2\text{-ortho}}$**	**$\Delta\Delta G^{\ddagger}_1$**	**ip_1**	**$ip_{1\text{-ortho}}$**	**$\Delta\Delta G_1$**	TS_3	$TS3_{\text{-ortho}}$	**$\Delta\Delta G^{\ddagger}_2$**
B3LYP-D3	19.1	20.5	**1.4**	8.2	9.8	**1.6**	11.4	18.0	**6.6**
PBE1PBE-D3	21.3	22.2	**0.9**	8.6	9.8	**1.2**	10.8	16.4	**5.6**
M062X	24.6	25.8	**1.2**	7.2	8.5	**1.3**	16.5	21.4	**4.9**
ωB97XD	26.7	28.2	**1.5**	9.8	10.6	**0.8**	13.6	20.2	**6.6**
M06	32.4	32.9	**0.4**	17.6	17.9	**0.3**	23.7	29.0	**5.3**
PBE1PBE	49.0	48.4	**-0.7**	31.5	29.8	**-1.8**	37.1	36.6	**-0.6**
B3LYP	61.2	60.2	**-1.0**	42.9	39.8	**-3.1**	50.3	48.4	**-1.9**

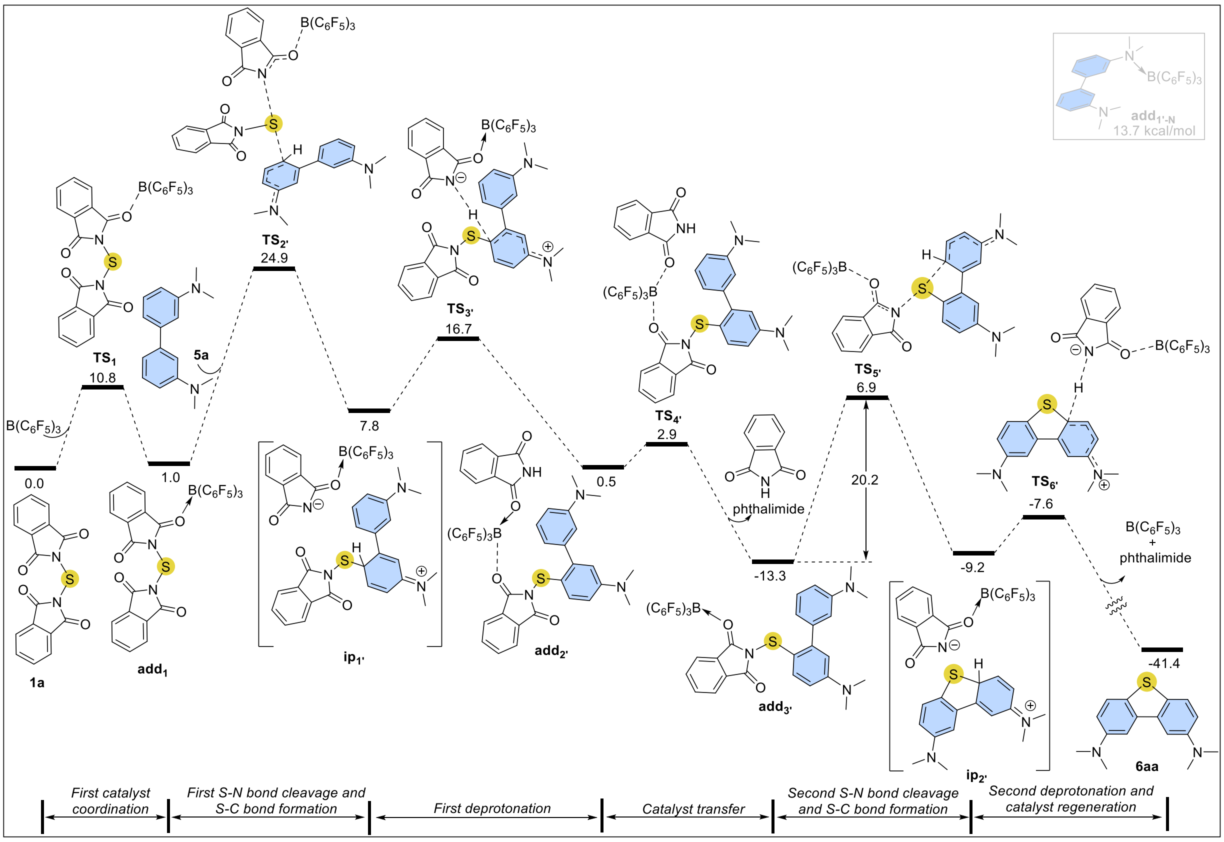

Figure S5. Free-energy profile for the $B(C_6F_5)_3$-catalyzed formation of dibenzothiophene **6aa** from biaryl substrate **5a**. Calculated in 1,2-dichloroethane at the SMD/M06-2X/def2-TZVP//SMD/M06-2X/6-31G(d) level of theory; relative free energies are given in kcal mol^{-1}.

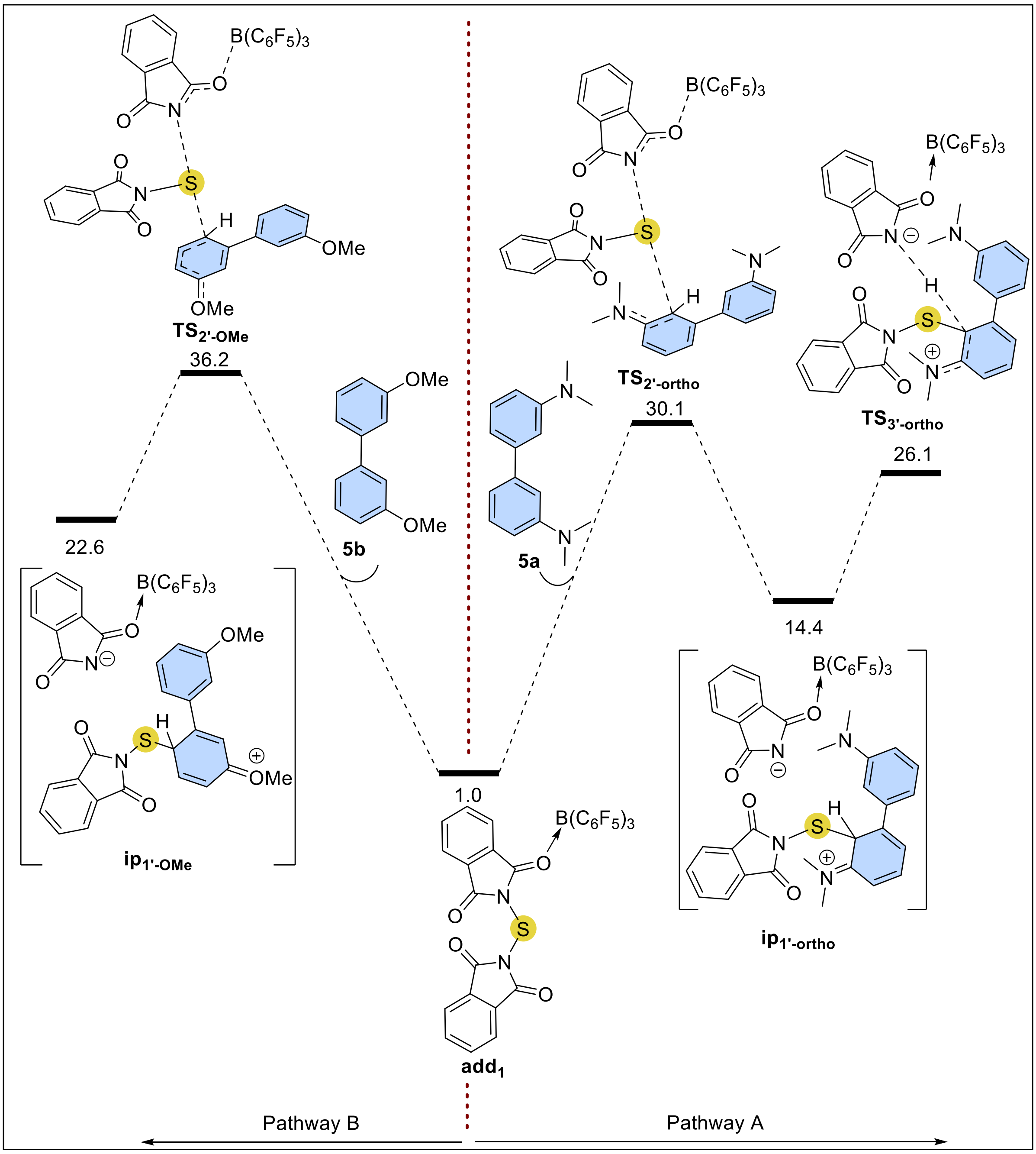

Figure S6. Free-energy profile for the *ortho* approach of **5a** (Pathway A) and formation of dibenzothiophene derivatives bearing two *para*-methoxy (OMe) substituents, starting from **5b** to form **6ab** (Pathway B). Calculated in 1,2-dichloroethane at the SMD/M06-2X/def2-TZVP//SMD/M06-2X/6-31G(d) level of theory; relative free energies are given in kcal mol^{-1}.

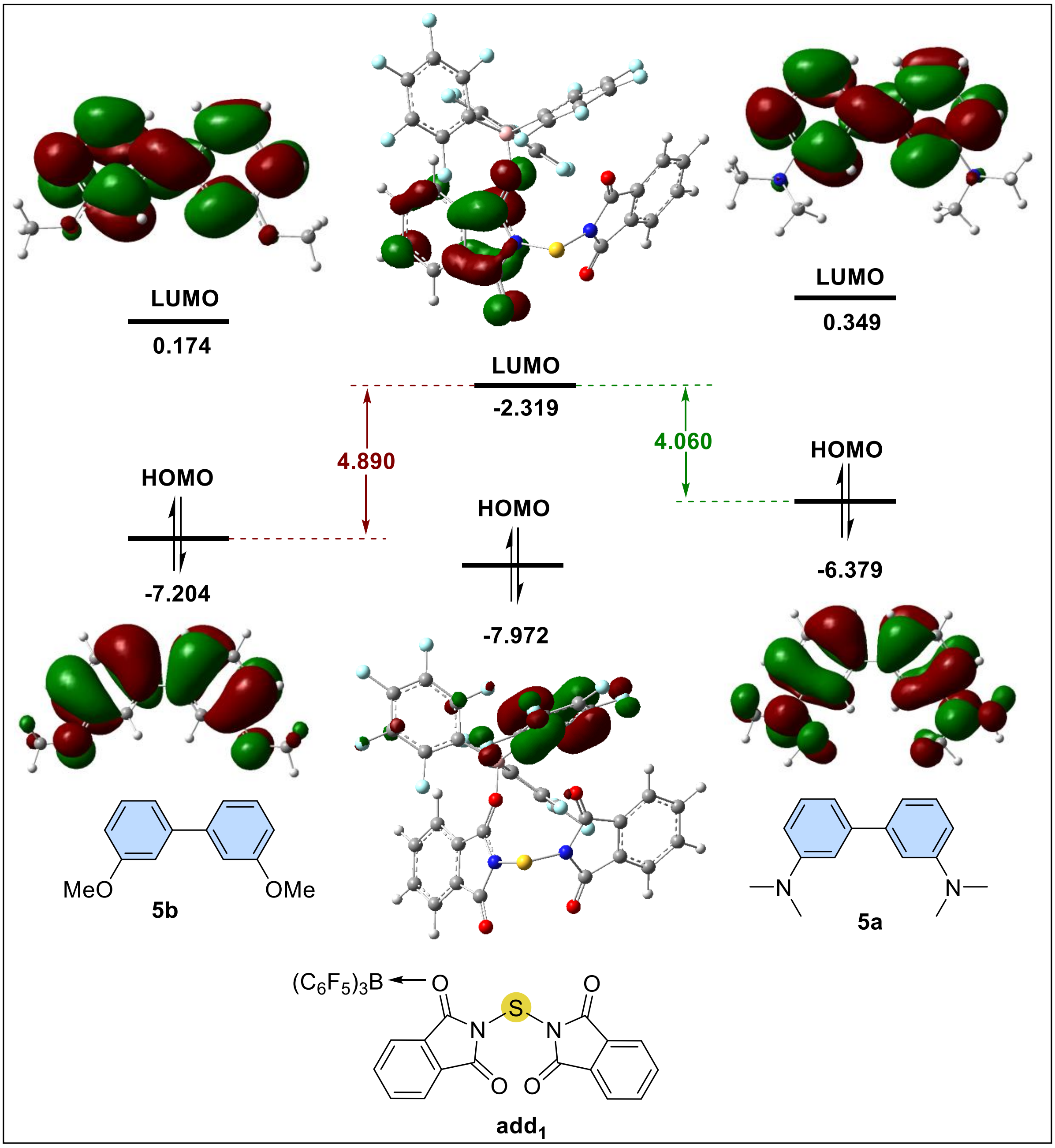

Figure S7. Spatial plots of HOMO and LUMO of **5b** (left), **add₁** (center) and **5a** (right).

6.2 Cartesian Coordinates and Total Energies for the Calculated Structures

1a

E(SMD/M06-2X/6-31g(d)) = -1422.744300 au

E(SMD/M06-2X/def2-TZVP//SMD/M06-2X/6-31g(d)) = -1423.178202 au

H(SMD/M06-2X/6-31g(d)) = -1422.510116 au

G(SMD/M06-2X/6-31g(d)) = -1422.575595 au

C	-2.015146	-1.152777	-0.631312
C	-3.093831	-0.658968	0.267574
C	-3.013939	0.727485	0.349172
C	-1.881467	1.195308	-0.494730

O	-1.488340	2.313243	-0.692342
O	-1.757489	-2.277177	-0.965740
N	-1.316306	0.014570	-1.053416
S	0.000001	0.000041	-2.113098
C	-4.956559	-0.641375	1.737878
C	-4.061176	-1.373664	0.954162
C	-3.896902	1.463406	1.121514
C	-4.875523	0.752289	1.820481
H	-5.731009	-1.161745	2.292283
H	-4.118154	-2.455271	0.884381
H	-3.827848	2.545009	1.179797
H	-5.587661	1.290032	2.438401
C	2.015125	1.152804	-0.631238
C	3.093827	0.658958	0.267607
C	3.013930	-0.727498	0.349157
C	1.881446	-1.195286	-0.494747
O	1.488326	-2.313214	-0.692413
O	1.757508	2.277212	-0.965672
N	1.316293	-0.014527	-1.053398
C	4.956579	0.641304	1.737880
C	4.061187	1.373626	0.954204
C	3.896903	-1.463450	1.121458
C	4.875538	-0.752363	1.820435
H	5.731042	1.161651	2.292290
H	4.118169	2.455234	0.884460
H	3.827845	-2.545055	1.179702
H	5.587684	-1.290130	2.438323

2a

E(SMD/M06-2X/6-31g(d)) = -366.054421 au

E(SMD/M06-2X/def2-TZVP//SMD/M06-2X/6-31g(d)) = -366.193234 au

H(SMD/M06-2X/6-31g(d)) = -365.869274 au

G(SMD/M06-2X/6-31g(d)) = -365.911937 au

C	0.543210	-1.206933	-0.030598
C	-0.186271	-0.000405	-0.088996
C	2.646601	-0.001668	0.049604
C	1.932399	-1.197569	0.032803
C	0.545735	1.205720	-0.054355
C	1.934684	1.195235	0.008617
H	3.730581	-0.002240	0.101694
H	2.459909	-2.146824	0.076505
H	0.029736	2.158314	-0.078439
H	2.464315	2.144039	0.030286
H	0.026436	-2.159254	-0.029674
C	-2.283034	-1.236333	0.053820
H	-2.123706	-1.629087	1.069823
H	-3.350448	-1.060958	-0.087666
H	-1.978302	-2.005095	-0.662580
N	-1.569178	0.003215	-0.189640
C	-2.275183	1.238731	0.097708

H	-2.016285	2.015051	-0.629204
H	-3.348078	1.057833	0.019934
H	-2.058759	1.625043	1.105186

3aa

E(SMD/M06-2X/6-31g(d)) = -1129.081607 au

E(SMD/M06-2X/def2-TZVP//SMD/M06-2X/6-31g(d)) = -1129.387437 au

H(SMD/M06-2X/6-31g(d)) = -1128.728038 au

G(SMD/M06-2X/6-31g(d)) = -1128.795803 au

S	0.013839	-2.428396	-0.008804
C	-1.388004	-1.327696	0.040469
C	-1.547546	-0.376541	1.052960
C	-2.388646	-1.443393	-0.924780
C	-2.668492	0.438187	1.098104
H	-0.777066	-0.261394	1.811151
C	-3.527970	-0.648746	-0.878396
H	-2.277478	-2.163356	-1.731092
C	-3.704094	0.307810	0.143465
H	-2.745049	1.172069	1.891692
H	-4.277183	-0.771397	-1.651286
N	-4.843450	1.081585	0.215374
C	-4.859369	2.222807	1.112054
H	-5.820453	2.729996	1.020180
H	-4.750329	1.902228	2.153378
H	-4.060521	2.944936	0.888275
C	-5.754810	1.087222	-0.913426
H	-5.274483	1.431942	-1.841005
H	-6.161234	0.085859	-1.089558
H	-6.591686	1.749209	-0.688742
C	1.398591	-1.301098	-0.052067
C	1.417673	-0.161797	-0.860281
C	2.531892	-1.596321	0.705706
C	2.532361	0.663550	-0.904830
H	0.544398	0.095582	-1.454225
C	3.662655	-0.790199	0.650959
H	2.534153	-2.466344	1.357127
C	3.697228	0.358930	-0.165685
H	2.497526	1.545831	-1.533124
H	4.517338	-1.056403	1.261264
N	4.826971	1.152732	-0.243987
C	4.706180	2.474060	-0.833396
H	3.954737	3.095327	-0.323553
H	5.671945	2.976991	-0.770366
H	4.434958	2.407747	-1.891982
C	5.883441	0.945997	0.728682
H	6.298594	-0.063218	0.641543
H	6.689597	1.653042	0.529961
H	5.539626	1.088832	1.764285

add$_1$

E(SMD/M06-2X/6-31g(d)) = -3630.288566 au

E(SMD/M06-2X/def2-TZVP//SMD/M06-2X/6-31g(d)) = -3631.682191 au
H(SMD/M06-2X/6-31g(d)) = -3629.865238 au
G(SMD/M06-2X/6-31g(d)) = -3629.994597 au

C	-3.818825	1.137452	1.358185
C	-4.510139	0.317963	0.329671
C	-3.982345	-0.970658	0.347893
C	-2.925622	-1.045650	1.390785
O	-2.251990	-1.975993	1.740571
O	-3.942152	2.300057	1.639076
N	-2.882562	0.258048	1.969117
S	-1.838205	0.699318	3.211324
C	-5.936218	-0.289151	-1.466102
C	-5.490724	0.689760	-0.573264
C	-4.406744	-1.944620	-0.539353
C	-5.402491	-1.580787	-1.450761
H	-6.706531	-0.041627	-2.189594
H	-5.892579	1.697860	-0.586166
H	-3.977947	-2.942007	-0.535580
H	-5.766066	-2.312846	-2.164685
C	0.146963	1.473620	1.471989
C	0.810955	2.716818	1.037405
C	0.181971	3.774773	1.700578
C	-0.870740	3.228864	2.591238
O	-1.624347	3.771450	3.340552
O	0.292298	0.287886	1.122474
N	-0.798980	1.794015	2.405522
C	2.292562	4.268884	0.012418
C	1.890869	2.942836	0.194689
C	0.575366	5.087146	1.530138
C	1.644511	5.322295	0.659222
H	3.131141	4.480400	-0.642573
H	2.420384	2.139870	-0.305024
H	0.076313	5.896054	2.053038
H	1.983743	6.339390	0.491907
B	0.965044	-0.369501	-0.156843
C	2.570875	-0.496934	0.051092
C	3.281813	-1.239920	-0.886343
C	3.315680	-0.041882	1.128885
C	4.635226	-1.512510	-0.784396
C	4.676037	-0.288534	1.272528
C	5.338571	-1.032566	0.311734
C	0.316786	-1.860817	-0.083262
C	0.564942	-2.618117	1.059493
C	-0.514017	-2.465431	-1.018130
C	0.052586	-3.887283	1.270338
C	-1.067648	-3.726758	-0.834510
C	-0.782167	-4.442912	0.314658
C	0.487471	0.530601	-1.428591
C	-0.865339	0.824165	-1.569831
C	1.295723	1.072787	-2.421703

C	-1.403842	1.580038	-2.595743
C	0.804901	1.838513	-3.473395
C	-0.553362	2.089100	-3.566382
F	-1.734103	0.352839	-0.656602
F	-2.715332	1.810925	-2.661673
F	2.629865	0.929308	-2.389364
F	1.635754	2.337595	-4.389223
F	-1.035487	2.819234	-4.567263
F	2.743001	0.684678	2.105140
F	5.341692	0.180686	2.329327
F	6.640671	-1.281565	0.433113
F	2.637182	-1.715312	-1.963676
F	5.265414	-2.229978	-1.715798
F	1.323423	-2.108074	2.043408
F	0.324059	-4.558091	2.392078
F	-1.313405	-5.649616	0.503337
F	-1.888997	-4.246373	-1.752736
F	-0.834136	-1.852571	-2.168869

add$_{1\text{-}N}$

E(SMD/M06-2X/6-31g(d)) = -2573.581482 au

E(SMD/M06-2X/def2-TZVP//SMD/M06-2X/6-31g(d)) = -2574.679366 au

H(SMD/M06-2X/6-31g(d)) = -2573.205397 au

G(SMD/M06-2X/6-31g(d)) = -2573.309505 au

C	-3.761731	2.155751	1.108073
C	-4.481017	2.138662	2.296224
C	-2.510626	1.545727	1.024336
C	-3.932678	1.506701	3.409023
C	-1.968657	0.902167	2.129710
H	-1.979325	1.602774	0.088584
C	-2.688027	0.892058	3.329484
H	-2.297129	0.412284	4.219162
N	-0.631454	0.222878	2.087852
C	-0.785555	-1.109383	2.777733
H	-0.858207	-0.947798	3.852720
H	-1.685395	-1.604926	2.425454
H	0.099396	-1.708273	2.575108
H	-4.472113	1.485464	4.350668
H	-5.454342	2.614957	2.358680
B	0.005768	-0.054653	0.507352
C	-1.154680	-0.937392	-0.302354
C	-1.898958	-0.427692	-1.373331
C	-1.477496	-2.272833	-0.027361
C	-2.884103	-1.124838	-2.062299
C	-2.452907	-3.007237	-0.686853
C	-3.171942	-2.430817	-1.717509
C	1.481260	-0.826895	0.629007
C	2.602615	-0.167781	1.152286
C	1.819405	-2.048809	0.032473
C	3.875852	-0.712950	1.244970

C	3.078884	-2.633170	0.102785
C	4.117982	-1.973158	0.731017
C	0.414118	1.315401	-0.333231
C	0.998370	1.072108	-1.581999
C	0.343835	2.666774	-0.014871
C	1.488809	2.050352	-2.428369
C	0.823655	3.685801	-0.832993
C	1.405766	3.380977	-2.047307
C	0.300484	0.977069	3.001937
H	-0.227224	1.174806	3.935301
H	1.156655	0.339790	3.215160
H	0.615371	1.909436	2.554915
F	2.529215	1.109095	1.566237
F	4.870245	-0.009052	1.789178
F	5.330229	-2.515469	0.795377
F	3.299636	-3.812379	-0.481960
F	0.965988	-2.726663	-0.744189
F	-0.814600	-2.981848	0.901237
F	-2.680359	-4.276148	-0.342583
F	-4.104434	-3.120300	-2.367693
F	-3.544882	-0.537435	-3.061308
F	-1.724820	0.826683	-1.829464
F	1.067685	-0.187978	-2.046394
F	-0.226129	3.100874	1.124846
F	0.713480	4.958587	-0.447217
F	1.865585	4.344869	-2.841954
F	2.026636	1.725747	-3.605097
H	-4.163566	2.647774	0.227882

add$_2$

E(SMD/M06-2X/6-31g(d)) = -3996.380821 au

E(SMD/M06-2X/def2-TZVP//SMD/M06-2X/6-31g(d)) = -3997.909590 au

H(SMD/M06-2X/6-31g(d)) = -3995.769861 au

G(SMD/M06-2X/6-31g(d)) = -3995.926005 au

C	2.160024	1.416811	-0.269157
C	2.157860	2.731299	-0.973426
C	3.062436	3.575221	-0.338310
C	3.711625	2.821375	0.773223
O	4.571535	3.189939	1.533107
O	1.506712	0.429808	-0.501999
N	3.107128	1.546622	0.768124
S	3.451575	0.316657	1.911818
C	1.563527	4.481612	-2.462645
C	1.397563	3.156341	-2.049459
C	3.241506	4.886564	-0.743281
C	2.468214	5.331459	-1.820069
H	0.975924	4.859886	-3.293813
H	0.689880	2.491125	-2.534203
H	3.946395	5.541166	-0.240520
H	2.570292	6.355734	-2.164620

C	4.572712	-0.721685	1.015941
C	5.942961	-0.442217	0.972312
C	4.094580	-1.866301	0.370304
C	6.815992	-1.273966	0.291527
H	6.327930	0.441293	1.474882
C	4.957014	-2.711662	-0.308133
H	3.032751	-2.091240	0.404836
C	6.344694	-2.435572	-0.369273
H	7.870222	-1.025119	0.274455
H	4.552191	-3.592154	-0.792373
N	7.200602	-3.262320	-1.040555
C	8.622195	-2.975164	-1.060199
H	9.134354	-3.756583	-1.621214
H	8.833029	-2.011885	-1.541898
H	9.042462	-2.950545	-0.046854
C	6.689375	-4.442329	-1.711333
H	6.226759	-5.142408	-1.003786
H	5.942370	-4.178602	-2.470239
H	7.513410	-4.953493	-2.208506
B	-1.646045	-0.025895	0.167945
O	-3.165605	-0.265027	0.613635
C	-5.399856	-1.087589	0.859418
C	-6.284753	-1.735443	0.000316
C	-5.609700	-1.885599	-1.318558
H	-3.652296	-1.199080	-1.901065
N	-4.315170	-1.309803	-1.139264
C	-4.156212	-0.830861	0.108552
O	-5.992992	-2.364820	-2.348103
C	-7.542976	-2.122004	0.418819
C	-5.722889	-0.793580	2.171027
C	-7.886814	-1.828978	1.744863
C	-6.997137	-1.178341	2.602308
H	-8.232582	-2.627599	-0.249334
H	-8.866433	-2.114430	2.114452
H	-7.299372	-0.967597	3.622909
H	-5.022236	-0.287011	2.827587
C	-1.302437	-1.062740	-1.036483
C	-1.516101	-2.425558	-0.836736
C	-0.775766	-0.733842	-2.280331
C	-1.230185	-3.403925	-1.772204
C	-0.469082	-1.682961	-3.250067
C	-0.696447	-3.024308	-2.995562
C	-0.869463	-0.358588	1.557878
C	-1.276430	0.278862	2.729129
C	0.189062	-1.244565	1.718194
C	-0.693160	0.070467	3.967490
C	0.809372	-1.475179	2.940401
C	0.369273	-0.815572	4.072288
C	-1.618159	1.549424	-0.241447
C	-0.820773	2.537023	0.324183

C	-2.463275	2.002865	-1.249515
C	-0.839884	3.864095	-0.091216
C	-2.520459	3.311937	-1.695426
C	-1.689696	4.255562	-1.108741
F	-0.537038	0.537271	-2.626711
F	-2.032726	-2.839843	0.333883
F	0.038749	-1.308375	-4.425360
F	-0.409833	-3.941437	-3.915226
F	-1.457797	-4.692359	-1.513635
F	-2.271421	1.182077	2.683784
F	-1.129606	0.720244	5.049948
F	0.961396	-1.021993	5.247695
F	1.852958	-2.305108	3.017096
F	0.687705	-1.943775	0.689684
F	-3.346183	3.667980	-2.681114
F	-3.269149	1.121309	-1.875047
F	0.073997	2.261320	1.286331
F	0.008088	4.745066	0.441031
F	-1.681934	5.512786	-1.546847

add$_3$

E(SMD/M06-2X/6-31g(d)) = -3483.461955 au

E(SMD/M06-2X/def2-TZVP//SMD/M06-2X/6-31g(d)) = -3484.791380 au

H(SMD/M06-2X/6-31g(d)) = -3482.979205 au

G(SMD/M06-2X/6-31g(d)) = -3483.109546 au

C	-0.290168	-1.399253	1.628766
C	-0.918525	-2.667365	1.198552
C	-0.331648	-3.685613	1.954141
C	0.655756	-3.079707	2.883183
O	1.396399	-3.589171	3.673471
O	-0.423633	-0.231427	1.214883
N	0.570834	-1.663667	2.647963
S	1.543246	-0.499028	3.484988
C	-2.319239	-4.281464	0.155427
C	-1.939437	-2.943312	0.299728
C	-0.701927	-5.008714	1.820530
C	-1.708731	-5.296437	0.892653
H	-3.112210	-4.531287	-0.541651
H	-2.444477	-2.171370	-0.270092
H	-0.234840	-5.786516	2.415712
H	-2.029926	-6.323508	0.752882
B	-0.924328	0.393228	-0.146266
C	-2.524574	0.660607	-0.070831
C	-3.096457	1.461404	-1.053524
C	-3.392798	0.239978	0.926100
C	-4.431263	1.828442	-1.065697
C	-4.739700	0.583114	0.954988
C	-5.261003	1.386038	-0.044688
C	-0.138764	1.824772	-0.068138
C	-0.428251	2.656863	1.013803

C	0.868543	2.286846	-0.905847
C	0.214269	3.860980	1.257609
C	1.547182	3.480399	-0.688935
C	1.222435	4.272355	0.398686
C	-0.446060	-0.599639	-1.347085
C	0.877011	-1.034116	-1.371705
C	-1.234099	-1.105581	-2.375274
C	1.401976	-1.894483	-2.320408
C	-0.752051	-1.969535	-3.352790
C	0.574196	-2.364922	-3.328623
F	1.716586	-0.606081	-0.419936
F	2.683316	-2.265472	-2.273039
F	-2.544334	-0.826446	-2.455612
F	-1.562862	-2.425344	-4.309489
F	1.045959	-3.192301	-4.257167
F	-2.964218	-0.544223	1.931751
F	-5.528807	0.149134	1.940083
F	-6.549327	1.722680	-0.033563
F	-2.330566	1.891007	-2.069555
F	-4.924947	2.593809	-2.040539
F	-1.363233	2.294758	1.906040
F	-0.117452	4.615685	2.306649
F	1.869745	5.414784	0.617132
F	2.548074	3.845016	-1.496742
F	1.270295	1.582646	-1.974445
C	2.791898	-0.176870	2.270951
C	2.858761	1.074683	1.654635
C	3.689490	-1.177199	1.883139
C	3.783397	1.317984	0.652348
H	2.172264	1.864788	1.948820
C	4.586893	-0.958923	0.852695
H	3.660351	-2.147946	2.372065
C	4.641369	0.293070	0.192042
H	3.808262	2.297281	0.189911
H	5.239833	-1.768684	0.550862
N	5.486779	0.507786	-0.867691
C	5.265828	1.683604	-1.695371
H	5.949076	1.651075	-2.544125
H	4.233667	1.731006	-2.070938
H	5.470947	2.602348	-1.136180
C	6.144190	-0.634884	-1.479832
H	6.804042	-1.134314	-0.764093
H	5.422463	-1.370181	-1.863508
H	6.759784	-0.284040	-2.308386

$B(C_6F_5)_3$

E(SMD/M06-2X/6-31g(d)) = -2207.510580 au

E(SMD/M06-2X/def2-TZVP//SMD/M06-2X/6-31g(d)) = -2208.479966 au

H(SMD/M06-2X/6-31g(d)) = -2207.323760 au

G(SMD/M06-2X/6-31g(d)) = -2207.413886 au

B	0.002087	-0.004396	-0.004544
C	1.462232	-0.579156	0.000179
C	2.521690	0.076239	0.634348
C	3.815639	-0.417888	0.652707
C	4.095982	-1.610651	0.002439
C	3.080700	-2.296393	-0.647819
C	1.795533	-1.780470	-0.631499
C	-0.229796	1.548171	-0.002004
C	-1.321834	2.139617	0.638796
C	-1.538304	3.507357	0.659122
C	-0.648300	4.345056	0.003377
C	0.447947	3.807084	-0.654428
C	0.641153	2.435610	-0.640247
C	-1.227401	-0.978889	-0.007725
C	-2.431063	-0.660831	-0.642839
C	-3.527580	-1.507032	-0.655694
C	-3.451183	-2.727060	-0.000504
C	-2.280585	-3.088042	0.649797
C	-1.199081	-2.222809	0.629117
F	2.318613	1.230462	1.275276
F	4.787622	0.239752	1.281571
F	5.330033	-2.095092	0.003983
F	3.347701	-3.439712	-1.275754
F	0.864256	-2.489978	-1.274051
F	-2.215741	1.387863	1.286693
F	-2.589079	4.021358	1.295094
F	-0.844438	5.656195	0.005763
F	1.716290	1.981280	-1.289696
F	-0.102476	-2.630518	1.273258
F	-2.205419	-4.257542	1.281802
F	-4.494139	-3.545284	0.004093
F	-4.649956	-1.158570	-1.281525
F	-2.570651	0.499687	-1.289175
F	1.302391	4.609265	-1.286224

ip1

E(SMD/M06-2X/6-31g(d)) = -3996.355716 au

E(SMD/M06-2X/def2-TZVP//SMD/M06-2X/6-31g(d)) = -3997.885205 au

H(SMD/M06-2X/6-31g(d)) = -3995.744364 au

G(SMD/M06-2X/6-31g(d)) = -3995.896478 au

C	-5.327850	1.307190	-0.981053
C	-6.633714	0.591954	-0.968143
C	-7.027660	0.400614	0.352839
C	-5.992634	0.982622	1.250677
O	-5.965794	1.033775	2.452138
O	-4.667708	1.670472	-1.918458
N	-4.983363	1.486684	0.386720
S	-3.576221	2.267304	0.917526
C	-8.594656	-0.515721	-1.717058
C	-7.402768	0.144493	-2.029142

C	-8.207271	-0.250259	0.673348
C	-8.989411	-0.710020	-0.390055
H	-9.227920	-0.882716	-2.518533
H	-7.090974	0.302315	-3.056913
H	-8.509329	-0.393553	1.706038
H	-9.921914	-1.225720	-0.183741
C	1.135070	1.744687	-0.875496
C	0.767027	2.797580	-1.866322
C	-0.104454	3.637790	-1.183800
C	-0.235996	3.049433	0.197637
O	-0.931623	3.483089	1.096176
O	1.974036	0.828934	-1.202722
N	0.546853	1.888595	0.289343
C	0.575484	4.156012	-3.798825
C	1.134806	3.030497	-3.177769
C	-0.661699	4.749519	-1.784163
C	-0.306072	4.996796	-3.117454
H	0.832611	4.380020	-4.829543
H	1.824991	2.377348	-3.703010
H	-1.342700	5.403269	-1.247470
H	-0.720850	5.859357	-3.630027
B	2.526920	-0.163736	-0.196008
C	3.718994	-0.963100	-0.997743
C	3.980703	-2.327569	-0.946365
C	4.658630	-0.219698	-1.709331
C	5.064850	-2.924414	-1.579084
C	5.751576	-0.774474	-2.359361
C	5.956254	-2.143405	-2.294106
C	3.315566	0.592085	1.025639
C	3.811584	-0.178270	2.072813
C	3.701039	1.926638	1.057842
C	4.588668	0.318912	3.105044
C	4.479890	2.470845	2.073998
C	4.926578	1.664170	3.105175
C	1.284114	-1.128264	0.268382
C	0.676248	-1.130691	1.519812
C	0.726168	-2.018356	-0.645316
C	-0.363228	-1.987544	1.864779
C	-0.310918	-2.889152	-0.346569
C	-0.856632	-2.876273	0.925807
F	1.072289	-0.296863	2.489650
F	-0.910263	-1.937524	3.083583
F	1.214752	-2.088676	-1.895138
F	-0.814540	-3.707730	-1.278843
F	-1.887774	-3.674250	1.225601
F	4.556437	1.118268	-1.766332
F	6.614985	-0.004072	-3.027530
F	7.004449	-2.698980	-2.902881
F	3.181013	-3.164791	-0.265134
F	5.256207	-4.244475	-1.496699

F	3.535357	-1.495350	2.105973
F	5.021164	-0.477222	4.087537
F	5.677354	2.170227	4.084397
F	4.807398	3.766378	2.054645
F	3.345620	2.786293	0.087648
C	-2.456101	0.764789	1.075960
C	-2.258048	0.239581	-0.293626
C	-3.098044	-0.183099	2.014108
C	-2.867362	-0.877671	-0.727595
H	-1.658582	0.834129	-0.977259
C	-3.702955	-1.308011	1.593801
H	-3.115682	0.088212	3.065136
C	-3.683286	-1.666053	0.183146
H	-2.739072	-1.203822	-1.751839
H	-4.190729	-1.961238	2.305892
H	-1.540730	1.206209	1.490689
N	-4.363238	-2.698758	-0.254945
C	-4.313492	-3.092888	-1.670551
H	-5.012965	-3.909932	-1.827956
H	-4.599031	-2.250807	-2.304156
H	-3.303779	-3.424457	-1.925673
C	-5.155635	-3.528980	0.662712
H	-4.499098	-4.002333	1.396758
H	-5.901000	-2.916208	1.174485
H	-5.661594	-4.298954	0.085998

ip$_{1\text{-ortho}}$

E(SMD/M06-2X/6-31g(d)) = -3996.348615 au

E(SMD/M06-2X/def2-TZVP//SMD/M06-2X/6-31g(d)) = -3997.880347 au

H(SMD/M06-2X/6-31g(d)) = -3995.737284 au

G(SMD/M06-2X/6-31g(d)) = -3995.892218 au

C	-6.106207	-0.233776	-0.219038
C	-6.293793	-1.622088	-0.716153
C	-5.689897	-2.503352	0.177112
C	-5.092275	-1.723882	1.294313
O	-4.491803	-2.104440	2.262903
O	-6.450014	0.812969	-0.707761
N	-5.383531	-0.362576	0.994335
S	-4.956162	0.937618	1.984611
C	-6.944536	-3.449495	-2.082651
C	-6.930461	-2.069491	-1.861148
C	-5.697014	-3.871281	-0.033128
C	-6.338561	-4.333877	-1.185436
H	-7.432574	-3.843998	-2.968174
H	-7.396715	-1.376410	-2.554046
H	-5.224475	-4.551880	0.667838
H	-6.366809	-5.399372	-1.389896
C	1.724191	2.092752	0.699145
C	1.951562	3.412354	1.359232
C	0.733328	4.071044	1.257034

C	-0.187582	3.110042	0.547574
O	-1.362610	3.314844	0.303630
O	2.684350	1.241558	0.645703
N	0.509336	1.939932	0.228562
C	2.880524	5.287609	2.473112
C	3.050149	3.991959	1.964640
C	0.552841	5.346515	1.754229
C	1.657610	5.952171	2.369481
H	3.715637	5.784411	2.957130
H	3.999831	3.472208	2.045326
H	-0.400946	5.859300	1.671649
H	1.562342	6.955151	2.774118
B	2.568089	-0.107102	-0.043561
C	4.034912	-0.804186	0.187495
C	4.795532	-1.461552	-0.771217
C	4.567764	-0.828143	1.473878
C	6.015079	-2.067568	-0.494424
C	5.782758	-1.416457	1.793378
C	6.515264	-2.041072	0.796175
C	1.508857	-1.100578	0.711024
C	1.305479	-2.370218	0.177514
C	0.875555	-0.874046	1.925030
C	0.510679	-3.342337	0.761896
C	0.066213	-1.819614	2.548476
C	-0.125823	-3.058988	1.963341
C	2.227385	0.207404	-1.615055
C	1.055604	-0.109032	-2.287969
C	3.135270	0.952593	-2.361662
C	0.791238	0.273443	-3.597030
C	2.919538	1.353460	-3.671130
C	1.729653	1.012529	-4.295706
F	0.080672	-0.817404	-1.693234
F	-0.368067	-0.055893	-4.178224
F	4.311003	1.308742	-1.816981
F	3.837499	2.064547	-4.332932
F	1.494544	1.391749	-5.552216
F	3.884283	-0.282787	2.493990
F	6.246437	-1.398297	3.046627
F	7.683481	-2.618313	1.079030
F	4.380988	-1.549408	-2.045340
F	6.708172	-2.676180	-1.461409
F	1.905728	-2.693471	-0.981710
F	0.353836	-4.540387	0.191484
F	-0.897151	-3.973849	2.550513
F	-0.549914	-1.523462	3.699035
F	1.009613	0.288305	2.582760
C	-3.347023	1.440439	1.186223
C	-3.659450	1.968152	-0.190689
C	-2.425245	0.271550	1.241624
C	-3.364450	1.120951	-1.319203

C	-2.125124	-0.433683	0.142850
H	-2.067327	-0.007732	2.228431
C	-2.635384	-0.007740	-1.145804
H	-3.690918	1.406884	-2.310032
H	-1.487008	-1.309039	0.187966
H	-2.978613	2.228258	1.848001
N	-4.257044	3.117258	-0.351385
C	-4.717637	3.548980	-1.677675
H	-5.275414	4.475609	-1.555538
H	-3.860911	3.724692	-2.333113
H	-5.375164	2.789370	-2.104259
C	-4.527813	4.071872	0.722383
H	-4.230442	5.061027	0.368795
H	-5.598849	4.070631	0.943534
H	-3.964260	3.844762	1.622865
H	-2.392318	-0.607225	-2.017722

ip$_2$

E(SMD/M06-2X/6-31g(d)) = -3849.517023 au

E(SMD/M06-2X/def2-TZVP//SMD/M06-2X/6-31g(d)) = -3850.979032 au

H(SMD/M06-2X/6-31g(d)) = -3848.845856 au

G(SMD/M06-2X/6-31g(d)) = -3848.999926 au

C	-1.114210	-1.302729	1.093609
C	-2.546632	-1.771787	0.995550
C	-2.513904	-3.066690	1.508192
C	-1.091869	-3.323185	1.920674
O	-0.678086	-4.355519	2.414622
O	-0.624107	-0.178346	0.705466
N	-0.320336	-2.197280	1.636674
S	3.387070	-2.061976	-1.658317
C	-4.896482	-2.015713	0.659705
C	-3.745743	-1.218504	0.574689
C	-3.638497	-3.860774	1.603792
C	-4.848983	-3.315396	1.158977
H	-5.845601	-1.604159	0.330441
H	-3.828292	-0.205884	0.203312
H	-3.582590	-4.864476	2.015145
H	-5.760172	-3.903163	1.211336
B	-1.225011	0.993052	-0.042607
C	-2.332556	1.805643	0.841983
C	-2.894102	2.959758	0.302953
C	-2.678805	1.548529	2.161799
C	-3.773398	3.782183	0.986405
C	-3.556937	2.346284	2.887910
C	-4.108243	3.469491	2.296613
C	0.038925	2.029138	-0.202535
C	0.808507	2.320437	0.921995
C	0.404843	2.709677	-1.357536
C	1.892926	3.183997	0.903938
C	1.481649	3.588087	-1.417812

C	2.226387	3.827942	-0.276690
C	-1.730902	0.421705	-1.495905
C	-0.802275	-0.287387	-2.254120
C	-2.984274	0.525846	-2.085098
C	-1.083054	-0.890986	-3.468124
C	-3.312629	-0.054291	-3.305732
C	-2.357373	-0.773365	-4.002354
F	0.465411	-0.408112	-1.814790
F	-0.146332	-1.590223	-4.116096
F	-3.991393	1.175222	-1.473390
F	-4.545396	0.068172	-3.802953
F	-2.657186	-1.345806	-5.166669
F	-2.180884	0.486165	2.816769
F	-3.865490	2.039773	4.150559
F	-4.951770	4.245838	2.976175
F	-2.575424	3.316318	-0.953091
F	-4.291815	4.869263	0.409501
F	0.502888	1.772964	2.107773
F	2.623708	3.398157	2.002148
F	3.290142	4.635489	-0.318218
F	1.794634	4.203823	-2.560570
F	-0.276837	2.552239	-2.502904
C	4.330219	-0.807633	-0.827675
C	3.905289	0.526700	-0.809048
C	5.558149	-1.124207	-0.235888
C	4.681953	1.510054	-0.219470
H	2.956302	0.800419	-1.267492
C	6.344150	-0.150660	0.361125
H	5.905894	-2.153801	-0.246627
C	5.921812	1.198985	0.388827
H	4.344084	2.537124	-0.248044
H	7.287551	-0.440763	0.808393
N	6.678287	2.173134	0.976408
C	6.173951	3.531629	1.051513
H	6.865121	4.134244	1.640781
H	6.078739	3.987442	0.057329
H	5.190340	3.564630	1.535809
C	7.957819	1.837062	1.569035
H	7.847208	1.127015	2.399169
H	8.637089	1.395226	0.829557
H	8.420246	2.745706	1.954211
C	2.088674	-2.460612	-0.376003
C	1.048259	-3.268987	-1.056874
C	2.712630	-3.139066	0.781612
C	0.694975	-4.500815	-0.655489
H	0.575356	-2.831846	-1.931607
C	2.368852	-4.371475	1.193089
H	3.490381	-2.593372	1.309900
C	1.307929	-5.098274	0.521061
H	-0.074469	-5.040788	-1.191952

H	2.867369	-4.816316	2.044195
N	0.895538	-6.259333	0.971076
C	-0.241351	-6.949630	0.351997
H	-0.038344	-7.150913	-0.701493
H	-0.394867	-7.895618	0.865345
H	-1.141874	-6.336116	0.449437
C	1.424479	-6.805992	2.225710
H	1.229221	-6.100690	3.037591
H	0.920400	-7.746323	2.435876
H	2.497096	-6.992175	2.137744
H	1.659601	-1.497232	-0.068657

phthalimide

E(SMD/M06-2X/6-31g(d)) = -512.904680 au

E(SMD/M06-2X/def2-TZVP//SMD/M06-2X/6-31g(d)) = -513.109007 au

H(SMD/M06-2X/6-31g(d)) = -512.778651 au

G(SMD/M06-2X/6-31g(d)) = -512.820333 au

C	1.242723	1.163102	0.000081
C	-0.177971	0.695813	0.000095
C	-0.177989	-0.695902	0.000048
C	1.242679	-1.163085	0.000033
O	1.669462	-2.293526	-0.000208
O	1.669223	2.293656	-0.000178
N	2.009828	0.000070	0.000353
C	-2.551765	0.698698	-0.000022
C	-1.355259	1.422819	-0.000038
C	-1.355285	-1.422890	0.000012
C	-2.551781	-0.698748	-0.000000
H	-3.498262	1.230164	-0.000173
H	-1.348836	2.508381	-0.000053
H	-1.348872	-2.508449	0.000071
H	-3.498294	-1.230182	0.000030
H	3.023879	-0.000279	-0.000507

TS_1

E(SMD/M06-2X/6-31g(d)) = -3630.271712 au

E(SMD/M06-2X/def2-TZVP//SMD/M06-2X/6-31g(d)) = -3631.665485 au

H(SMD/M06-2X/6-31g(d)) = -3629.850044 au

G(SMD/M06-2X/6-31g(d)) = -3629.978751 au

C	3.290618	1.062196	-1.011180
C	4.061653	0.459081	0.112460
C	4.066888	-0.924016	-0.040641
C	3.276899	-1.264467	-1.254862
O	3.081479	-2.333656	-1.779943
O	3.082485	2.224188	-1.258642
N	2.798988	-0.035556	-1.750039
S	1.800904	0.023443	-3.156465
C	5.320234	0.245010	2.110359
C	4.676439	1.075500	1.186783
C	4.707191	-1.754969	0.861470
C	5.336981	-1.142924	1.950262

H	5.813210	0.686666	2.970541
H	4.652545	2.153748	1.308934
H	4.703532	-2.833839	0.737814
H	5.843437	-1.757228	2.687957
C	-0.163742	1.301659	-1.724335
C	-0.504517	2.726992	-1.454408
C	0.250993	3.524121	-2.316944
C	1.119382	2.661425	-3.147966
O	1.897146	2.946315	-4.011471
O	-0.577332	0.262153	-1.241272
N	0.817969	1.320658	-2.727579
C	-1.462016	4.703795	-0.528890
C	-1.375592	3.309432	-0.542996
C	0.163518	4.903838	-2.321525
C	-0.710956	5.490543	-1.404083
H	-2.131067	5.182041	0.179486
H	-1.972865	2.717449	0.136844
H	0.760581	5.499811	-3.004032
H	-0.805404	6.570961	-1.366486
B	-1.194030	-0.648840	0.525631
C	-2.656312	-1.090578	0.084504
C	-3.126975	-2.395004	0.221831
C	-3.569672	-0.190774	-0.457584
C	-4.404933	-2.784082	-0.155826
C	-4.849023	-0.539393	-0.857056
C	-5.270445	-1.851584	-0.702850
C	-0.065530	-1.762097	0.486003
C	0.019120	-2.649386	-0.588650
C	0.847804	-1.979823	1.514041
C	0.930238	-3.687807	-0.645624
C	1.774179	-3.014694	1.493726
C	1.821267	-3.864331	0.402494
C	-1.002677	0.614568	1.464352
C	0.224461	1.274843	1.519267
C	-2.014829	1.175911	2.238257
C	0.448944	2.421294	2.259659
C	-1.829780	2.322047	3.002566
C	-0.593796	2.949288	3.009496
F	1.258501	0.814869	0.797454
F	1.640996	3.013734	2.255522
F	-3.234347	0.629387	2.276027
F	-2.829710	2.820551	3.727222
F	-0.407741	4.048359	3.729968
F	-3.227702	1.095907	-0.607004
F	-5.672709	0.370917	-1.376065
F	-6.495665	-2.209630	-1.070974
F	-2.358490	-3.354747	0.751139
F	-4.801758	-4.046130	0.005238
F	-0.817853	-2.525827	-1.624177
F	1.002191	-4.479117	-1.713117

F	2.742850	-4.819649	0.340295
F	2.638159	-3.170545	2.498776
F	0.861804	-1.198286	2.601421

TS_2

E(SMD/M06-2X/6-31g(d)) = -3996.329716 au

E(SMD/M06-2X/def2-TZVP//SMD/M06-2X/6-31g(d)) = -3997.857046 au

H(SMD/M06-2X/6-31g(d)) = -3995.720254 au

G(SMD/M06-2X/6-31g(d)) = -3995.870986 au

C	-3.096466	-1.553285	-0.072634
C	-3.401103	-0.777803	1.161131
C	-3.022127	0.547010	0.965480
C	-2.448651	0.686622	-0.402110
O	-1.984302	1.655493	-0.939411
O	-3.223927	-2.728818	-0.288353
N	-2.585255	-0.601167	-1.000600
S	-2.186246	-0.962398	-2.582693
C	-4.119831	-0.253267	3.362454
C	-3.947199	-1.210227	2.357014
C	-3.172651	1.498683	1.959443
C	-3.738270	1.076540	3.167237
H	-4.548331	-0.550000	4.314624
H	-4.227963	-2.248497	2.505803
H	-2.871454	2.530315	1.809752
H	-3.876725	1.794102	3.969682
C	0.806429	-1.498472	-1.327908
C	1.796609	-2.610282	-1.128623
C	1.185279	-3.742423	-1.662078
C	-0.149217	-3.315046	-2.193928
O	-0.965330	-4.001786	-2.757203
O	0.920647	-0.270878	-1.007981
N	-0.281968	-1.935055	-1.937022
C	3.722596	-3.922659	-0.637440
C	3.086170	-2.676014	-0.621699
C	1.804151	-4.976256	-1.690128
C	3.095056	-5.055190	-1.156162
H	4.729464	-4.005330	-0.240600
H	3.604001	-1.810146	-0.224878
H	1.309439	-5.843493	-2.116424
H	3.620198	-6.005188	-1.153041
B	1.779158	0.401023	0.072569
C	3.269449	0.770587	-0.484373
C	4.078994	1.592928	0.292389
C	3.790712	0.441747	-1.727498
C	5.322933	2.051945	-0.107042
C	5.034794	0.877066	-2.170937
C	5.804031	1.691082	-1.357311
C	0.978763	1.817505	0.275861
C	0.878165	2.665072	-0.825711
C	0.329884	2.270626	1.416649

C	0.208738	3.877664	-0.811697
C	-0.359127	3.476773	1.471096
C	-0.426769	4.283520	0.349876
C	1.770400	-0.569856	1.394044
C	0.550638	-1.086872	1.818441
C	2.859304	-1.001335	2.142081
C	0.389848	-1.960452	2.879424
C	2.753159	-1.877732	3.217019
C	1.510887	-2.359181	3.591863
F	-0.571531	-0.728795	1.169262
F	-0.819844	-2.410453	3.219973
F	4.113266	-0.635548	1.825686
F	3.843408	-2.266204	3.881808
F	1.394896	-3.202869	4.615101
F	3.106299	-0.339115	-2.580460
F	5.487278	0.524731	-3.377271
F	6.997922	2.120098	-1.766463
F	3.655448	1.962112	1.513064
F	6.055449	2.835020	0.688971
F	1.441954	2.316521	-1.993616
F	0.149539	4.641942	-1.905927
F	-1.094984	5.436882	0.388015
F	-0.990670	3.847228	2.592600
F	0.327578	1.555807	2.554434
C	-4.104464	-0.306236	-3.408608
C	-4.290329	1.037472	-2.943142
C	-4.960280	-1.309587	-2.838926
C	-5.058801	1.314192	-1.850787
H	-3.760510	1.842659	-3.442825
C	-5.717906	-1.057574	-1.734128
H	-4.946640	-2.309191	-3.263311
C	-5.739527	0.257183	-1.156115
H	-5.136913	2.334139	-1.496375
H	-6.303324	-1.853842	-1.292625
H	-3.775689	-0.443077	-4.437080
N	-6.376186	0.488847	-0.003794
C	-6.470922	1.841365	0.539450
H	-6.909769	1.787826	1.534346
H	-5.479551	2.292794	0.623685
H	-7.100930	2.475028	-0.093757
C	-7.073822	-0.591712	0.688880
H	-7.949873	-0.923194	0.121227
H	-6.403797	-1.442475	0.841403
H	-7.401567	-0.230604	1.662450

$TS_{2\text{-}ortho}$

E(SMD/M06-2X/6-31g(d)) = -3996.328106 au

E(SMD/M06-2X/def2-TZVP//SMD/M06-2X/6-31g(d)) = -3997.855215 au

H(SMD/M06-2X/6-31g(d)) = -3995.718836 au

G(SMD/M06-2X/6-31g(d)) = -3995.869339 au

C	-3.338703	-0.370189	0.893126
C	-3.238465	0.828682	1.765968
C	-2.658812	1.865482	1.040163
C	-2.329783	1.370250	-0.326118
O	-1.802791	1.943753	-1.240968
O	-3.781991	-1.462473	1.139545
N	-2.799344	0.024144	-0.359448
S	-2.759552	-0.972444	-1.704791
C	-3.378631	2.231030	3.674641
C	-3.607648	0.981589	3.090368
C	-2.429738	3.106849	1.608165
C	-2.802614	3.274727	2.945288
H	-3.649987	2.394901	4.712695
H	-4.051238	0.163403	3.648957
H	-1.984982	3.918200	1.041485
H	-2.637784	4.232806	3.428039
C	0.242664	-1.715504	-0.625143
C	1.036063	-2.914653	-0.190334
C	0.149444	-3.986952	-0.274830
C	-1.153936	-3.445474	-0.775033
O	-2.158088	-4.070650	-1.022006
O	0.618363	-0.505840	-0.732172
N	-0.996611	-2.057991	-0.939360
C	2.742095	-4.428048	0.498701
C	2.355375	-3.119033	0.185131
C	0.519914	-5.282702	0.023614
C	1.843643	-5.491800	0.425841
H	3.767964	-4.615174	0.799989
H	3.082861	-2.317946	0.239263
H	-0.188763	-6.101193	-0.056199
H	2.179011	-6.493403	0.675706
B	1.791927	0.244693	-0.084166
C	3.182852	0.022787	-0.908369
C	4.287579	0.791900	-0.558095
C	3.361404	-0.785796	-2.021998
C	5.498945	0.752831	-1.227932
C	4.558863	-0.860017	-2.724446
C	5.634177	-0.084707	-2.325842
C	1.337697	1.801207	-0.320706
C	1.161385	2.227316	-1.635773
C	1.042769	2.755836	0.643299
C	0.737964	3.498387	-1.987169
C	0.603674	4.037656	0.334323
C	0.443608	4.410969	-0.987795
C	1.845198	-0.205307	1.491502
C	0.654287	-0.192318	2.211612
C	2.945185	-0.639386	2.221457
C	0.531995	-0.569922	3.537438
C	2.875636	-1.031102	3.554470
C	1.662782	-0.993370	4.219417

F	-0.471883	0.224048	1.606664
F	-0.648726	-0.523439	4.157906
F	4.158137	-0.759184	1.654517
F	3.968957	-1.454819	4.192000
F	1.579944	-1.366667	5.494469
F	2.364000	-1.560452	-2.481741
F	4.676688	-1.666264	-3.782651
F	6.789399	-0.143104	-2.987515
F	4.200122	1.618077	0.497906
F	6.527721	1.507579	-0.833992
F	1.386490	1.377215	-2.651188
F	0.577593	3.839347	-3.268509
F	0.001861	5.631033	-1.294466
F	0.295586	4.900993	1.309889
F	1.163658	2.490494	1.954026
C	-4.661313	-0.318806	-2.513373
C	-4.543617	1.111130	-2.638285
C	-5.568977	-0.866959	-1.516384
C	-5.075253	1.947416	-1.703979
H	-3.953223	1.493332	-3.465565
C	-6.089041	0.041188	-0.540460
C	-5.836663	1.384188	-0.640847
H	-4.926942	3.020157	-1.757102
H	-6.698662	-0.325665	0.275287
H	-4.523806	-0.881264	-3.433745
N	-5.843882	-2.170392	-1.450802
C	-6.545202	-2.711014	-0.287826
H	-7.563054	-2.314500	-0.227359
H	-6.597423	-3.793707	-0.389384
H	-6.000577	-2.466967	0.629352
C	-5.312342	-3.140772	-2.403459
H	-5.255240	-2.712896	-3.405179
H	-4.320804	-3.487356	-2.089295
H	-5.994655	-3.990717	-2.442747
H	-6.247267	2.045716	0.117557

TS$_{2\text{-}a}$

E(SMD/M06-2X/6-31g(d)) = -3996.333516 au

E(SMD/M06-2X/def2-TZVP//SMD/M06-2X/6-31g(d)) = -3997.859262 au

H(SMD/M06-2X/6-31g(d)) = -3995.724378 au

G(SMD/M06-2X/6-31g(d)) = -3995.871554 au

C	3.212722	-0.525423	-0.114008
C	4.526203	-1.226311	-0.112539
C	5.147255	-1.020441	-1.340075
C	4.280559	-0.141391	-2.169829
O	4.453181	0.269821	-3.285030
O	2.336282	-0.562757	0.711599
N	3.169181	0.190495	-1.337972
S	1.878372	1.112453	-1.869029
C	6.373612	-2.528392	0.611531

C	5.117991	-1.980134	0.887381
C	6.385179	-1.570471	-1.629318
C	6.992847	-2.332968	-0.627579
H	6.875271	-3.122923	1.368530
H	4.624639	-2.137732	1.841546
H	6.860499	-1.413583	-2.592367
H	7.963584	-2.782505	-0.811549
C	-0.310099	-1.142923	-1.289199
C	-0.529307	-2.577582	-0.891724
C	0.566574	-3.261058	-1.415753
C	1.423879	-2.240276	-2.103107
O	2.511460	-2.429452	-2.607388
O	-1.028422	-0.123163	-1.017512
N	0.781496	-1.000521	-2.019453
C	-1.366496	-4.645272	-0.053012
C	-1.520953	-3.260531	-0.206211
C	0.731963	-4.623193	-1.272149
C	-0.262079	-5.317917	-0.572346
H	-2.129314	-5.202484	0.481674
H	-2.393501	-2.769565	0.204739
H	1.595810	-5.129866	-1.691512
H	-0.175583	-6.391333	-0.435178
B	-2.095720	0.120349	0.048389
C	-1.434673	-0.327062	1.479489
C	-0.235915	0.275655	1.858174
C	-1.899249	-1.287176	2.370856
C	0.473924	-0.058181	2.999370
C	-1.221287	-1.654987	3.527657
C	-0.022486	-1.039176	3.842644
C	-2.358149	1.736983	-0.067569
C	-2.632782	2.273746	-1.323160
C	-2.383473	2.652421	0.976891
C	-2.877563	3.620607	-1.548983
C	-2.624589	4.009309	0.795859
C	-2.870300	4.498205	-0.476008
C	-3.522784	-0.570411	-0.340313
C	-3.821905	-1.226701	-1.526463
C	-4.605709	-0.367537	0.508941
C	-5.097738	-1.682327	-1.840707
C	-5.891447	-0.804435	0.239271
C	-6.138756	-1.469482	-0.953328
F	-2.869638	-1.472906	-2.440994
F	-5.327440	-2.317534	-2.993104
F	-4.412431	0.288064	1.666271
F	-6.888265	-0.587243	1.101075
F	-7.366839	-1.899664	-1.241058
F	-3.048805	-1.950254	2.147234
F	-1.716643	-2.599353	4.332841
F	0.640392	-1.377563	4.948438
F	0.274605	1.263186	1.111138

F	1.624073	0.555918	3.298617
F	-2.685268	1.470601	-2.398219
F	-3.129093	4.074454	-2.779794
F	-3.102881	5.797331	-0.664536
F	-2.624072	4.844404	1.839192
F	-2.168912	2.267588	2.244928
C	2.741477	2.973648	-1.399561
C	4.145420	2.917851	-1.747320
C	2.430025	2.913246	0.014980
C	5.077532	2.521085	-0.845350
H	4.439289	3.140232	-2.768155
C	3.339317	2.481173	0.923863
H	1.413505	3.131239	0.323253
C	4.682021	2.177705	0.504919
H	6.110734	2.413083	-1.151403
H	3.043993	2.336019	1.954157
H	2.096198	3.579294	-2.033144
N	5.528279	1.581698	1.336132
C	6.918366	1.322745	0.959274
H	7.467221	1.017209	1.848447
H	6.973648	0.523728	0.210991
H	7.384016	2.227154	0.562840
C	5.071732	1.086299	2.637334
H	4.965102	1.908383	3.352443
H	4.114155	0.569267	2.531841
H	5.808460	0.379311	3.015862

$TS_{2\text{-}b}$

E(SMD/M06-2X/6-31g(d)) = -3996.330791 au

E(SMD/M06-2X/def2-TZVP//SMD/M06-2X/6-31g(d)) = -3997.857470 au

H(SMD/M06-2X/6-31g(d)) = -3995.721268 au

G(SMD/M06-2X/6-31g(d)) = -3995.869969 au

C	-2.616784	-0.669444	0.821302
C	-2.998593	0.754602	0.613296
C	-3.199876	0.973811	-0.748340
C	-2.873319	-0.273404	-1.491313
O	-2.842054	-0.476829	-2.674725
O	-2.402180	-1.266880	1.839844
N	-2.608068	-1.248333	-0.483517
S	-2.392082	-2.870112	-0.783903
C	-3.726049	2.968007	1.077195
C	-3.239178	1.743637	1.551095
C	-3.681845	2.179230	-1.227370
C	-3.953307	3.178547	-0.285554
H	-3.926778	3.770951	1.780161
H	-3.069516	1.569180	2.609247
H	-3.838375	2.342090	-2.289109
H	-4.330063	4.139945	-0.620651
C	0.829314	-1.940598	-0.645251
C	2.125832	-2.704377	-0.751693

C	1.756901	-4.018940	-1.035996
C	0.268417	-4.044045	-1.122524
O	-0.451942	-4.990955	-1.342288
O	0.641140	-0.710190	-0.373127
N	-0.196796	-2.740771	-0.890683
C	4.411284	-3.383991	-0.838066
C	3.466365	-2.364516	-0.659690
C	2.675364	-5.032802	-1.216975
C	4.028955	-4.696270	-1.106126
H	5.466090	-3.137593	-0.767652
H	3.806061	-1.356081	-0.473118
H	2.355399	-6.045661	-1.440994
H	4.788193	-5.460386	-1.238468
B	1.620599	0.368620	0.114646
C	2.684752	0.810415	-1.047174
C	3.569151	1.847287	-0.764195
C	2.710986	0.362864	-2.361191
C	4.454124	2.380626	-1.686439
C	3.582396	0.866706	-3.320635
C	4.460285	1.881443	-2.981084
C	0.672085	1.690511	0.309508
C	-0.051926	2.137229	-0.794293
C	0.605388	2.518578	1.423620
C	-0.765109	3.324631	-0.818914
C	-0.137065	3.693866	1.457137
C	-0.826772	4.098966	0.328685
C	2.278436	-0.170655	1.514405
C	1.400594	-0.552904	2.527380
C	3.622114	-0.299678	1.845499
C	1.798971	-1.050284	3.757105
C	4.070064	-0.797535	3.064372
C	3.152990	-1.176542	4.027733
F	0.081212	-0.409717	2.342181
F	0.900545	-1.392904	4.682697
F	4.596115	0.028150	0.976532
F	5.377737	-0.915049	3.307210
F	3.563428	-1.655670	5.200360
F	1.882632	-0.609703	-2.778412
F	3.573170	0.382735	-4.565037
F	5.300930	2.376757	-3.888524
F	3.580039	2.381444	0.468873
F	5.288451	3.366777	-1.348949
F	-0.058501	1.413517	-1.926751
F	-1.411048	3.717358	-1.920240
F	-1.571405	5.204606	0.349450
F	-0.192128	4.431454	2.569856
F	1.274390	2.229590	2.550746
C	-4.561248	-3.387731	-0.689770
C	-5.125877	-2.562423	-1.711926
C	-4.869080	-3.045164	0.664657

C	-5.753189	-1.385347	-1.414612
H	-5.004785	-2.856089	-2.750443
C	-5.487571	-1.868751	0.981593
H	-4.555174	-3.715009	1.459746
C	-5.907451	-0.968204	-0.051041
H	-6.123759	-0.761998	-2.218569
H	-5.664036	-1.623889	2.020887
H	-4.350264	-4.428667	-0.924638
N	-6.430028	0.229819	0.246695
C	-7.000407	1.070913	-0.801092
H	-7.298432	2.021807	-0.361961
H	-6.265267	1.267373	-1.585366
H	-7.880846	0.597060	-1.249280
C	-6.610407	0.644636	1.634281
H	-7.438031	0.104119	2.108117
H	-5.697014	0.471483	2.207957
H	-6.826974	1.712377	1.652743

TS2-c

E(SMD/M06-2X/6-31g(d)) = -3996.325033 au

E(SMD/M06-2X/def2-TZVP//SMD/M06-2X/6-31g(d)) = -3997.852713 au

H(SMD/M06-2X/6-31g(d)) = -3995.715429 au

G(SMD/M06-2X/6-31g(d)) = -3995.866752 au

C	3.336512	-1.741805	0.598779
C	4.578442	-2.542461	0.426024
C	5.405746	-1.890391	-0.482756
C	4.735940	-0.640362	-0.929961
O	5.107379	0.184080	-1.722408
O	2.341284	-1.998786	1.226581
N	3.526392	-0.572111	-0.184229
S	2.308673	0.557208	-0.451262
C	6.182755	-4.277510	0.644161
C	4.941831	-3.746206	1.005988
C	6.635367	-2.408346	-0.850235
C	7.014544	-3.620580	-0.267457
H	6.508488	-5.218605	1.075912
H	4.290244	-4.251990	1.711524
H	7.273491	-1.894496	-1.562220
H	7.970886	-4.063311	-0.527595
C	0.022114	-1.080997	-1.298325
C	-0.482491	-2.437190	-1.667615
C	0.620983	-3.102313	-2.202590
C	1.782698	-2.157605	-2.141779
O	2.933470	-2.379753	-2.439865
O	-0.582236	-0.109902	-0.752825
N	1.302061	-0.940183	-1.627175
C	-1.812439	-4.366203	-2.079338
C	-1.722579	-3.051057	-1.605176
C	0.543436	-4.395946	-2.676963
C	-0.703767	-5.028330	-2.604418

H	-2.769298	-4.876719	-2.038052
H	-2.601706	-2.557953	-1.207298
H	1.413089	-4.896837	-3.090734
H	-0.811089	-6.046510	-2.964876
B	-1.839348	-0.013787	0.123058
C	-1.685685	-1.177148	1.257914
C	-0.553708	-1.161175	2.074659
C	-2.542780	-2.248012	1.477368
C	-0.262155	-2.142496	3.008169
C	-2.293239	-3.252263	2.405366
C	-1.142664	-3.201630	3.172446
C	-1.722331	1.507474	0.719956
C	-1.451351	2.548187	-0.164812
C	-1.878708	1.887383	2.047310
C	-1.300167	3.867237	0.232368
C	-1.727778	3.197779	2.487797
C	-1.423303	4.192659	1.573352
C	-3.200882	-0.045804	-0.767921
C	-3.280691	-0.088335	-2.152456
C	-4.414882	0.115544	-0.108608
C	-4.485129	-0.008361	-2.844060
C	-5.636051	0.199349	-0.754275
C	-5.668447	0.138557	-2.141191
F	-2.176992	-0.233444	-2.904977
F	-4.507060	-0.068530	-4.177823
F	-4.418896	0.184399	1.233574
F	-6.771304	0.344179	-0.067277
F	-6.830791	0.216049	-2.788146
F	-3.678973	-2.391016	0.770800
F	-3.151466	-4.264345	2.556756
F	-0.885270	-4.156517	4.065124
F	0.307004	-0.137093	1.994536
F	0.838191	-2.071982	3.757865
F	-1.333136	2.303148	-1.478774
F	-1.005458	4.824908	-0.654490
F	-1.240400	5.451236	1.972604
F	-1.871636	3.503934	3.779369
F	-2.182840	0.985546	2.991871
C	3.112348	1.956978	1.063162
C	2.018624	2.883456	1.128866
C	4.302642	2.415211	0.417840
C	2.041982	4.064866	0.448872
H	1.128918	2.606467	1.691826
C	4.347543	3.598013	-0.269457
H	5.187958	1.787046	0.442908
C	3.202787	4.451333	-0.308099
H	1.190119	4.731599	0.496001
H	5.254113	3.878863	-0.789524
H	3.193313	1.206725	1.845881
N	3.208451	5.586291	-1.017617

C	2.032981	6.451590	-1.052663
H	2.208083	7.250407	-1.770946
H	1.149404	5.889298	-1.366316
H	1.841649	6.897583	-0.070611
C	4.403702	5.989579	-1.751488
H	5.243565	6.155053	-1.068812
H	4.684761	5.225343	-2.482821
H	4.195239	6.918301	-2.279663

$TS_{2\text{-}d}$

E(SMD/M06-2X/6-31g(d)) = -3996.323399 au

E(SMD/M06-2X/def2-TZVP//SMD/M06-2X/6-31g(d)) = -3997.850104 au

H(SMD/M06-2X/6-31g(d)) = -3995.713587 au

G(SMD/M06-2X/6-31g(d)) = -3995.865618 au

C	2.772535	3.356363	-1.173672
C	2.273124	4.293335	-0.130478
C	1.908071	3.557167	0.990368
C	2.159462	2.114815	0.722727
O	2.018223	1.172589	1.457599
O	3.189840	3.595936	-2.275412
N	2.661656	2.058645	-0.603666
S	2.938249	0.642934	-1.471707
C	1.595642	6.289770	0.959625
C	2.131174	5.671015	-0.172462
C	1.372806	4.158340	2.116831
C	1.220086	5.546162	2.083877
H	1.461083	7.366779	0.967099
H	2.415793	6.240905	-1.051156
H	1.075315	3.570501	2.979632
H	0.797586	6.058315	2.942641
C	-0.174374	0.317544	-1.817066
C	-1.287659	0.266412	-2.823273
C	-0.753008	0.825801	-3.982982
C	0.672313	1.184632	-3.688741
O	1.480572	1.654803	-4.451054
O	-0.164193	-0.081933	-0.608818
N	0.915419	0.856496	-2.341264
C	-3.335499	-0.094945	-3.986812
C	-2.586869	-0.217852	-2.809413
C	-1.478697	0.949007	-5.150651
C	-2.797680	0.481759	-5.136431
H	-4.356865	-0.461785	-4.001042
H	-3.029052	-0.687140	-1.939739
H	-1.038106	1.386298	-6.041349
H	-3.406917	0.558838	-6.031551
B	-1.290883	-0.475509	0.345856
C	-1.892714	-1.956493	0.015155
C	-2.841705	-2.487611	0.883213
C	-1.460178	-2.820829	-0.980827
C	-3.362964	-3.765145	0.768340

C	-1.953172	-4.112162	-1.134031
C	-2.910345	-4.587085	-0.254067
C	-0.501409	-0.622968	1.775714
C	0.593603	-1.482285	1.840894
C	-0.805895	0.021981	2.966735
C	1.338263	-1.689594	2.990134
C	-0.071666	-0.141392	4.135417
C	1.010555	-1.002696	4.148065
C	-2.380029	0.749596	0.325970
C	-1.900502	2.037425	0.552261
C	-3.750022	0.682585	0.105242
C	-2.686045	3.177390	0.556155
C	-4.581069	1.797909	0.093540
C	-4.047484	3.054046	0.322797
F	-0.596893	2.211837	0.823130
F	-2.151334	4.376646	0.797154
F	-4.360979	-0.487250	-0.157475
F	-5.888534	1.665318	-0.141888
F	-4.831682	4.130402	0.317115
F	-0.525897	-2.440885	-1.868674
F	-1.506614	-4.897856	-2.117878
F	-3.392808	-5.822514	-0.385450
F	-3.296152	-1.729896	1.895524
F	-4.284022	-4.213931	1.624436
F	0.971231	-2.176391	0.752688
F	2.367547	-2.549171	3.003352
F	1.717466	-1.185623	5.263356
F	-0.410526	0.515271	5.249487
F	-1.852878	0.858108	3.060232
C	4.919822	0.347124	-0.683912
C	5.407402	-0.518428	-1.727900
C	4.601603	-0.287177	0.568183
C	5.276350	-1.869367	-1.649519
H	5.797668	-0.068694	-2.636400
C	4.477136	-1.640154	0.675512
H	4.383417	0.334527	1.430384
C	4.742584	-2.479631	-0.456498
H	5.578989	-2.488196	-2.484462
H	4.157685	-2.075449	1.612415
H	5.284998	1.370787	-0.666937
N	4.515908	-3.791407	-0.405506
C	4.835743	-4.652279	-1.542513
H	4.590989	-5.680215	-1.282524
H	4.254156	-4.363060	-2.423369
H	5.901584	-4.598118	-1.782203
C	3.898901	-4.388454	0.777925
H	4.572052	-4.336268	1.639849
H	2.967216	-3.870242	1.017963
H	3.677972	-5.432842	0.566727

$TS_{2\text{-}e}$

E(SMD/M06-2X/6-31g(d)) = -3996.320840 au
E(SMD/M06-2X/def2-TZVP//SMD/M06-2X/6-31g(d)) = -3997.849958 au
H(SMD/M06-2X/6-31g(d)) = -3995.710917 au
G(SMD/M06-2X/6-31g(d)) = -3995.862261 au

C	-2.000895	1.547526	1.564601
C	-1.631202	2.897669	1.059185
C	-1.924797	2.961967	-0.301537
C	-2.438790	1.636551	-0.742409
O	-2.801153	1.279684	-1.831782
O	-2.009580	1.133117	2.694028
N	-2.475713	0.835431	0.431666
S	-3.289079	-0.632129	0.526659
C	-1.041826	5.197310	1.042459
C	-1.177056	4.002199	1.757776
C	-1.794059	4.137937	-1.018583
C	-1.351136	5.265428	-0.318326
H	-0.689578	6.088552	1.552691
H	-0.949845	3.945312	2.817781
H	-2.026427	4.181410	-2.077917
H	-1.233536	6.208433	-0.842927
C	-0.256141	-1.627187	-0.336702
C	0.367726	-2.974809	-0.595562
C	-0.693511	-3.879050	-0.584537
C	-1.934160	-3.094070	-0.319849
O	-3.063041	-3.506164	-0.185716
O	0.305656	-0.487285	-0.246786
N	-1.569617	-1.741196	-0.219711
C	1.841301	-4.791184	-1.063820
C	1.656907	-3.419293	-0.846239
C	-0.527895	-5.232032	-0.798684
C	0.773624	-5.685870	-1.038768
H	2.843690	-5.160169	-1.256892
H	2.510878	-2.756964	-0.882271
H	-1.376240	-5.909065	-0.783294
H	0.954751	-6.741975	-1.211463
B	1.781476	-0.135760	-0.012087
C	2.688557	-0.324496	-1.360342
C	4.026216	0.056507	-1.311335
C	2.230574	-0.692367	-2.618231
C	4.873685	0.040831	-2.406484
C	3.043373	-0.721411	-3.746028
C	4.373281	-0.352352	-3.639338
C	1.762746	1.485927	0.230831
C	1.278066	2.289747	-0.799945
C	2.322585	2.181628	1.294991
C	1.383801	3.670728	-0.816210
C	2.400105	3.569593	1.343973
C	1.930056	4.318812	0.280563
C	2.268541	-0.991442	1.295468

C	1.500226	-0.884077	2.453428
C	3.360944	-1.842128	1.409709
C	1.766889	-1.564528	3.629783
C	3.666509	-2.549610	2.567602
C	2.865744	-2.409194	3.686266
F	0.444423	-0.056872	2.465241
F	0.992130	-1.408760	4.704983
F	4.189612	-2.066717	0.374044
F	4.723520	-3.364829	2.602479
F	3.143778	-3.075760	4.805183
F	0.951416	-1.055580	-2.807507
F	2.549661	-1.090992	-4.930554
F	5.166590	-0.373621	-4.709891
F	4.548960	0.465483	-0.142854
F	6.153742	0.402830	-2.291450
F	0.701709	1.723447	-1.874020
F	0.953324	4.381563	-1.861899
F	1.980493	5.650195	0.314542
F	2.928145	4.182461	2.407246
F	2.848227	1.540478	2.350263
C	-5.081797	0.310581	1.373413
C	-5.833112	-0.878722	1.658690
C	-5.535246	1.100618	0.267814
C	-6.784971	-1.348154	0.804699
H	-5.584977	-1.450666	2.548572
C	-6.501976	0.660801	-0.590894
H	-5.068781	2.066371	0.088727
C	-7.139339	-0.602826	-0.372999
H	-7.293874	-2.276929	1.029257
H	-6.774391	1.267318	-1.444820
H	-4.587312	0.808270	2.204772
N	-8.047171	-1.074188	-1.233246
C	-8.676182	-2.374647	-1.016380
H	-9.312108	-2.602455	-1.869712
H	-7.918495	-3.158846	-0.928293
H	-9.292125	-2.367524	-0.111459
C	-8.423302	-0.296143	-2.410559
H	-8.834889	0.674730	-2.118485
H	-7.558317	-0.135869	-3.061981
H	-9.184071	-0.842757	-2.964475

TS$_3$

E(SMD/M06-2X/6-31g(d)) = -3996.340031 au

E(SMD/M06-2X/def2-TZVP//SMD/M06-2X/6-31g(d)) = -3997.864437 au

H(SMD/M06-2X/6-31g(d)) = -3995.735871 au

G(SMD/M06-2X/6-31g(d)) = -3995.886861 au

C	-3.068318	2.652741	1.344648
C	-3.927482	1.504933	0.943793
C	-3.947854	1.428412	-0.445463
C	-3.080097	2.503391	-0.997771

O	-2.792930	2.737430	-2.145912
O	-2.786826	3.053587	2.443073
N	-2.606976	3.219174	0.127179
S	-1.683715	4.642642	0.032360
C	-5.274352	-0.443892	1.074646
C	-4.587598	0.579822	1.733404
C	-4.652838	0.438535	-1.108132
C	-5.309210	-0.512293	-0.321163
H	-5.788363	-1.202380	1.656716
H	-4.554734	0.636977	2.816879
H	-4.702446	0.409302	-2.191864
H	-5.854725	-1.317172	-0.803988
C	0.701063	0.606759	1.371408
C	1.581251	-0.066932	2.381669
C	1.640073	0.817698	3.457995
C	0.832494	2.023221	3.075067
O	0.678037	3.037766	3.713080
O	0.348649	0.188093	0.219936
N	0.302347	1.792289	1.793433
C	3.087895	-1.513871	3.526259
C	2.321428	-1.239021	2.386794
C	2.394424	0.559530	4.585414
C	3.119155	-0.638173	4.610883
H	3.673387	-2.427199	3.559279
H	2.335409	-1.926295	1.548765
H	2.432706	1.262785	5.411812
H	3.725846	-0.882821	5.476932
B	0.296892	-1.226684	-0.368153
C	-0.320774	-2.207390	0.786391
C	0.198071	-3.404852	1.261604
C	-1.508399	-1.808792	1.396045
C	-0.400842	-4.152171	2.269862
C	-2.141229	-2.516941	2.403731
C	-1.578701	-3.706207	2.844244
C	-0.730778	-1.003433	-1.626083
C	-1.928607	-1.662092	-1.870633
C	-0.442380	0.030443	-2.513140
C	-2.796864	-1.296829	-2.891583
C	-1.291181	0.447142	-3.524932
C	-2.490696	-0.221236	-3.707454
C	1.735182	-1.673534	-0.987680
C	1.804143	-2.867359	-1.698477
C	2.904634	-0.927241	-0.978593
C	2.944224	-3.315658	-2.343686
C	4.070855	-1.334858	-1.614990
C	4.091711	-2.535391	-2.303223
F	0.713111	-3.647330	-1.766416
F	2.953750	-4.476681	-3.001312
F	2.970067	0.256476	-0.339315
F	5.167056	-0.570026	-1.577717

F	5.200141	-2.939308	-2.921720
F	-2.108597	-0.681153	0.983944
F	-3.280099	-2.077197	2.946550
F	-2.165048	-4.410940	3.810180
F	1.361593	-3.892182	0.795110
F	0.157474	-5.288762	2.692850
F	-2.338177	-2.683948	-1.102863
F	-3.972688	-1.918048	-3.035911
F	-3.361148	0.185784	-4.630905
F	-0.983491	1.500107	-4.283891
F	0.712269	0.711269	-2.390211
C	-0.007618	3.978400	0.143827
C	0.915278	4.756664	0.952863
C	0.563100	3.351489	-1.037875
C	2.258229	4.653119	0.793786
H	0.513508	5.357280	1.764240
C	1.903402	3.234675	-1.203869
H	-0.113902	2.884590	-1.748224
C	2.811031	3.846016	-0.264761
H	2.916738	5.185995	1.467615
H	2.288592	2.678755	-2.049323
H	-0.068604	2.979240	0.899910
N	4.121561	3.676735	-0.377892
C	5.042627	4.266772	0.594953
H	6.040952	3.877040	0.407807
H	5.062872	5.356644	0.501980
H	4.747112	3.992644	1.610655
C	4.689143	2.911740	-1.490771
H	4.348244	1.874764	-1.452075
H	4.406975	3.357370	-2.448353
H	5.773948	2.928650	-1.405586

$TS_{3\text{-ortho}}$

E(SMD/M06-2X/6-31g(d)) = -3996.327934 au

E(SMD/M06-2X/def2-TZVP//SMD/M06-2X/6-31g(d)) = -3997.856737 au

H(SMD/M06-2X/6-31g(d)) = -3995.721633 au

G(SMD/M06-2X/6-31g(d)) = -3995.874614 au

C	4.663717	-1.592923	0.038697
C	6.136004	-1.749423	0.205841
C	6.774636	-0.706167	-0.458114
C	5.740243	0.163103	-1.082994
O	5.878425	1.188786	-1.703168
O	3.770546	-2.262468	0.484544
N	4.500437	-0.443758	-0.778932
S	2.996136	0.184799	-1.269908
C	8.240801	-2.607292	0.888076
C	6.847372	-2.718926	0.891140
C	8.153536	-0.583617	-0.464957
C	8.882249	-1.558337	0.222535
H	8.836955	-3.347641	1.412154

H	6.342269	-3.529533	1.406488
H	8.645061	0.233241	-0.983370
H	9.965979	-1.501641	0.241051
C	-0.832665	1.749316	0.118092
C	-1.784187	2.619710	0.884238
C	-1.176015	3.875466	0.916441
C	0.142860	3.738021	0.217742
O	0.999931	4.581949	0.075512
O	-0.932072	0.519069	-0.192889
N	0.251658	2.418186	-0.243451
C	-3.584875	3.496277	2.174206
C	-2.991806	2.402965	1.529418
C	-1.749167	4.958930	1.551166
C	-2.983083	4.753288	2.180114
H	-4.533278	3.355370	2.682891
H	-3.473060	1.433718	1.563479
H	-1.256640	5.926322	1.566789
H	-3.472379	5.577654	2.689080
B	-2.096867	-0.464830	-0.112088
C	-3.364080	0.222188	-0.888526
C	-4.662864	0.386229	-0.423790
C	-3.151657	0.696675	-2.181729
C	-5.675972	0.982898	-1.165306
C	-4.129464	1.296341	-2.958849
C	-5.409652	1.437700	-2.444499
C	-1.518407	-1.778609	-0.904374
C	-2.183088	-2.512383	-1.879209
C	-0.262016	-2.269000	-0.554957
C	-1.633164	-3.627215	-2.500539
C	0.328609	-3.370609	-1.156243
C	-0.367160	-4.055363	-2.141014
C	-2.359822	-0.951888	1.422247
C	-3.304795	-1.950659	1.639078
C	-1.629138	-0.582838	2.543571
C	-3.550544	-2.524982	2.874351
C	-1.839612	-1.133108	3.803661
C	-2.806900	-2.108924	3.970171
F	-4.028872	-2.398697	0.599994
F	-4.477972	-3.473545	3.022335
F	-0.660540	0.348879	2.469229
F	-1.113425	-0.729817	4.849390
F	-3.024782	-2.645773	5.169899
F	-1.939883	0.565453	-2.745357
F	-3.856186	1.725788	-4.192736
F	-6.367424	2.011250	-3.170398
F	-5.012295	0.010478	0.820596
F	-6.899595	1.122878	-0.650297
F	-3.421896	-2.180012	-2.276015
F	-2.320067	-4.292348	-3.433793
F	0.173856	-5.124589	-2.725626

F	1.539948	-3.791304	-0.789308
F	0.435681	-1.680872	0.431318
C	2.723575	1.424313	0.038194
C	3.693169	2.550577	-0.042208
C	2.619299	0.737238	1.338848
C	4.590636	2.756373	1.064366
C	3.422034	1.034517	2.379823
H	1.857637	-0.035011	1.406323
C	4.446572	2.029979	2.205405
H	5.377196	3.496409	0.995323
H	3.325092	0.527271	3.333167
H	1.583816	1.845091	-0.195317
N	3.749772	3.333174	-1.097676
C	2.846460	3.245601	-2.243258
H	1.946732	2.686676	-1.999645
H	2.558561	4.262384	-2.517132
H	3.366046	2.773796	-3.082387
C	4.806885	4.339433	-1.228637
H	5.788210	3.867133	-1.140863
H	4.721989	4.796202	-2.213405
H	4.693247	5.113409	-0.465129
H	5.134936	2.215434	3.025478

TS_4

E(SMD/M06-2X/6-31g(d)) = -3996.369792 au

E(SMD/M06-2X/def2-TZVP//SMD/M06-2X/6-31g(d)) = -3997.897205 au

H(SMD/M06-2X/6-31g(d)) = -3995.759194 au

G(SMD/M06-2X/6-31g(d)) = -3995.912894 au

C	2.446700	1.521235	0.494409
C	2.985093	2.464147	-0.524770
C	4.263710	2.847334	-0.130116
C	4.617530	2.107170	1.116007
O	5.655883	2.102979	1.726251
O	1.368698	0.977777	0.543428
N	3.466872	1.356734	1.446801
S	3.412216	0.152387	2.671779
C	3.172737	3.829570	-2.458750
C	2.420872	2.926141	-1.701746
C	5.019594	3.735754	-0.874342
C	4.445810	4.234070	-2.047974
H	2.763722	4.218829	-3.386026
H	1.446184	2.583109	-2.031083
H	6.016987	4.027678	-0.561275
H	5.003795	4.936463	-2.659137
C	3.914905	-1.258712	1.720680
C	5.270340	-1.587927	1.607493
C	2.971703	-2.032699	1.034002
C	5.684554	-2.653592	0.825311
H	6.011742	-0.988751	2.129001
C	3.373872	-3.103450	0.252768

H	1.915259	-1.784237	1.105669
C	4.742077	-3.441412	0.121654
H	6.743932	-2.868695	0.751935
H	2.626549	-3.692409	-0.262727
N	5.130132	-4.493419	-0.660873
C	6.542120	-4.763237	-0.858093
H	6.648195	-5.662542	-1.465024
H	7.054999	-3.935787	-1.367350
H	7.043487	-4.941810	0.099194
C	4.152645	-5.145653	-1.514463
H	3.377918	-5.647370	-0.922733
H	3.658897	-4.431231	-2.187173
H	4.657824	-5.899301	-2.118361
B	-1.163238	0.597820	-0.004153
O	-3.342986	0.275932	-0.381495
C	-5.223876	-1.259115	-0.243383
C	-5.553736	-2.384736	-0.994027
C	-4.500864	-2.568805	-2.037477
H	-2.778569	-1.359782	-2.416005
N	-3.585140	-1.525113	-1.822677
C	-3.952667	-0.703307	-0.781942
O	-4.407436	-3.409124	-2.895871
C	-6.682401	-3.133914	-0.716751
C	-6.001987	-0.831716	0.816682
C	-7.480666	-2.710533	0.351839
C	-7.147367	-1.581555	1.104423
H	-6.935747	-4.012944	-1.300616
H	-8.375354	-3.271044	0.603434
H	-7.787485	-1.283669	1.928661
H	-5.732820	0.044730	1.397906
C	-0.655666	-0.808926	-0.533623
C	-1.152182	-2.006777	-0.022500
C	0.368566	-0.941494	-1.471397
C	-0.678755	-3.255154	-0.393904
C	0.884388	-2.171468	-1.854321
C	0.349863	-3.332642	-1.318706
C	-1.436876	0.784706	1.545099
C	-2.325142	1.751561	2.016909
C	-0.810701	0.023745	2.530477
C	-2.581651	1.957284	3.362989
C	-1.030086	0.211338	3.888057
C	-1.924675	1.182080	4.306486
C	-1.105666	1.865922	-0.950808
C	-0.738633	3.133855	-0.506309
C	-1.423290	1.778274	-2.303357
C	-0.702894	4.246189	-1.336307
C	-1.395082	2.862358	-3.166366
C	-1.033528	4.108555	-2.675028
F	0.929444	0.131970	-2.037766
F	-2.145683	-1.992602	0.876740

F	1.899779	-2.250785	-2.714753
F	0.859020	-4.514991	-1.657026
F	-1.195480	-4.365441	0.129843
F	-2.972401	2.548921	1.161304
F	-3.444108	2.895467	3.757725
F	-2.154743	1.368055	5.602325
F	-0.394838	-0.541934	4.786580
F	0.049282	-0.949318	2.213533
F	-1.712600	2.720801	-4.453107
F	-1.775464	0.598938	-2.840562
F	-0.378789	3.333863	0.765404
F	-0.336339	5.436647	-0.862153
F	-0.998995	5.161162	-3.485887

$TS_{4\text{-concerted}}$

E(SMD/M06-2X/6-31g(d)) = -4362.419411 au

E(SMD/M06-2X/def2-TZVP//SMD/M06-2X/6-31g(d)) = -4364.076698 au

H(SMD/M06-2X/6-31g(d)) = -4361.621696 au

G(SMD/M06-2X/6-31g(d)) = -4361.795808 au

C	0.950351	1.773266	1.039028
C	0.706947	3.050947	0.288745
C	1.694693	3.924991	0.740894
C	2.536544	3.166020	1.725318
O	3.525637	3.559388	2.297754
O	0.324379	0.666256	0.954782
N	1.982503	1.882524	1.857095
S	3.233681	0.042682	2.379324
C	-0.072938	4.749361	-1.188203
C	-0.184126	3.441511	-0.698993
C	1.815807	5.215136	0.265252
C	0.901937	5.625352	-0.712129
H	-0.759900	5.083807	-1.959182
H	-0.937095	2.777054	-1.105308
H	2.594385	5.877718	0.630667
H	0.959080	6.631877	-1.114629
C	4.178979	0.299725	0.944121
C	3.747536	-0.208184	-0.293064
C	5.376474	1.036104	0.972352
C	4.483301	-0.020643	-1.447167
H	2.803753	-0.740730	-0.340052
C	6.133528	1.218224	-0.170090
H	5.704691	1.476698	1.909641
C	5.730877	0.651031	-1.406346
H	4.091235	-0.400635	-2.383377
H	7.045080	1.800341	-0.105720
N	6.517460	0.747560	-2.521873
C	5.981720	0.342986	-3.811194
H	6.757628	0.461595	-4.567667
H	5.683399	-0.710260	-3.796757
H	5.110004	0.943555	-4.105107

C	7.689321	1.606645	-2.501886
H	7.428841	2.662176	-2.343592
H	8.382829	1.300725	-1.711970
H	8.207577	1.515203	-3.456540
B	-1.010357	0.288874	0.336622
O	-6.151866	0.648063	1.147163
C	-5.941025	-1.315300	-0.293067
C	-5.765839	-1.422647	-1.668791
C	-5.674516	-0.041147	-2.234849
H	-5.807079	1.823981	-1.220724
N	-5.826732	0.812621	-1.142811
C	-5.995882	0.135368	0.062250
O	-5.507484	0.303629	-3.380377
C	-5.622876	-2.651026	-2.288745
C	-5.975905	-2.429112	0.526446
C	-5.667588	-3.785769	-1.471926
C	-5.835673	-3.677058	-0.088478
H	-5.468492	-2.727485	-3.360362
H	-5.556632	-4.769489	-1.917492
H	-5.846626	-4.576534	0.519297
H	-6.085286	-2.333035	1.601639
C	-0.897906	0.113032	-1.283758
C	-1.983084	-0.440910	-1.954084
C	0.226765	0.317348	-2.068556
C	-1.982848	-0.772631	-3.297334
C	0.280972	-0.004387	-3.420833
C	-0.829413	-0.552394	-4.039419
C	-1.276630	-1.224929	0.929155
C	-2.471503	-1.699972	1.461290
C	-0.255025	-2.168383	0.863663
C	-2.636660	-2.998192	1.930403
C	-0.374276	-3.469378	1.329014
C	-1.578395	-3.889089	1.870676
C	-2.143571	1.341687	0.867778
C	-2.263630	1.536103	2.241607
C	-3.079465	2.029660	0.105272
C	-3.230833	2.335247	2.830848
C	-4.062401	2.841888	0.653578
C	-4.150799	2.987223	2.025713
F	1.345863	0.854343	-1.552215
F	-3.114559	-0.673482	-1.264451
F	1.400636	0.205952	-4.119925
F	-0.792384	-0.865797	-5.333907
F	-3.059269	-1.307958	-3.875985
F	-3.554508	-0.911139	1.548888
F	-3.805319	-3.395936	2.443305
F	-1.718851	-5.136351	2.318889
F	0.654504	-4.321202	1.249444
F	0.924667	-1.852425	0.302790
F	-4.952888	3.456213	-0.137956

F	-3.068091	1.968849	-1.240004
F	-1.428903	0.895715	3.075679
F	-3.299289	2.462951	4.157465
F	-5.103275	3.744071	2.563852
C	4.246618	-1.909325	3.012201
C	3.948160	-2.727109	1.880735
C	5.598785	-1.483910	3.169272
C	4.836429	-2.860031	0.851562
H	2.970006	-3.193597	1.807707
C	6.503159	-1.603758	2.151907
H	5.893930	-1.000288	4.096137
C	6.109012	-2.199509	0.908648
H	4.566581	-3.444068	-0.019345
H	7.508826	-1.223191	2.278438
H	3.621738	-1.994302	3.896485
N	6.908594	-2.146563	-0.166443
C	6.536825	-2.809088	-1.411374
H	5.515852	-2.542102	-1.693763
H	7.211311	-2.473011	-2.198625
H	6.612233	-3.899101	-1.322098
C	8.229175	-1.532515	-0.083164
H	8.635399	-1.440042	-1.090073
H	8.155922	-0.534169	0.355973
H	8.913925	-2.140641	0.519240

TS_5

E(SMD/M06-2X/6-31g(d)) = -3849.502139 au

E(SMD/M06-2X/def2-TZVP//SMD/M06-2X/6-31g(d)) = -3850.967143 au

H(SMD/M06-2X/6-31g(d)) = -3848.832770 au

G(SMD/M06-2X/6-31g(d)) = -3848.984791 au

C	-0.848007	2.100116	-0.439037
C	-0.753697	3.432783	-1.096490
C	0.359555	4.046347	-0.537483
C	0.924785	3.064389	0.452188
O	1.885252	3.226042	1.166998
O	-1.823146	1.332147	-0.730895
N	0.126981	1.896142	0.428267
S	1.392840	-0.135932	0.583032
C	-1.142921	5.339341	-2.447243
C	-1.532426	4.051819	-2.056545
C	0.746721	5.320004	-0.906400
C	-0.027809	5.961794	-1.881516
H	-1.717524	5.865349	-3.203292
H	-2.397390	3.560143	-2.491289
H	1.612804	5.801016	-0.462305
H	0.243000	6.961998	-2.205408
B	-2.246375	0.012241	-0.091485
C	-1.962964	0.095546	1.517512
C	-2.442629	1.207251	2.206547
C	-1.262001	-0.804692	2.306336

C	-2.232575	1.439236	3.554558
C	-1.013629	-0.610456	3.660827
C	-1.504932	0.516502	4.292866
C	-3.846337	-0.074456	-0.451764
C	-4.253507	0.135009	-1.767669
C	-4.869068	-0.408462	0.427645
C	-5.572360	0.050217	-2.188894
C	-6.202472	-0.505285	0.048032
C	-6.557728	-0.272828	-1.269391
C	-1.523101	-1.233058	-0.857103
C	-0.555118	-1.138560	-1.847335
C	-1.927634	-2.529089	-0.553932
C	0.008652	-2.249608	-2.460614
C	-1.398793	-3.665786	-1.144727
C	-0.412266	-3.520370	-2.109809
F	-0.063451	0.046998	-2.243116
F	0.991047	-2.101497	-3.358130
F	-2.858913	-2.717775	0.396901
F	-1.819830	-4.884814	-0.797454
F	0.147440	-4.590748	-2.675335
F	-0.294937	-1.504950	4.347770
F	-1.281869	0.716836	5.591403
F	-3.147740	2.139747	1.541125
F	-2.722067	2.530812	4.148825
F	-3.345192	0.415011	-2.717523
F	-5.900194	0.260630	-3.467047
F	-7.831063	-0.368442	-1.651754
F	-7.141487	-0.828701	0.941516
F	-4.617715	-0.674136	1.719180
C	2.891492	0.444597	0.081619
C	3.854191	0.923803	1.015305
C	3.262168	0.426140	-1.294008
C	5.104796	1.317749	0.618060
H	3.575035	0.983722	2.062868
C	4.506453	0.821803	-1.708894
H	2.533285	0.075914	-2.020756
C	5.486710	1.240230	-0.756096
H	5.807488	1.683072	1.356784
H	4.752836	0.783482	-2.762776
N	6.739678	1.540974	-1.145868
C	7.752504	1.885058	-0.157703
H	8.714128	1.990475	-0.657644
H	7.514663	2.828930	0.345339
H	7.838032	1.097405	0.599960
C	7.111703	1.480543	-2.553148
H	7.011412	0.462535	-2.947161
H	6.488798	2.151613	-3.153225
H	8.149883	1.792228	-2.655823
C	2.024142	-2.655252	0.612480
C	2.702438	-2.376433	1.817416

C	4.059469	-2.117207	1.829204
H	2.135420	-2.327453	2.743321
C	4.803218	-2.127698	0.619043
H	4.544083	-1.875897	2.767088
C	4.137069	-2.560617	-0.568432
C	2.789909	-2.828628	-0.557679
H	4.694422	-2.682562	-1.489461
H	2.312267	-3.154688	-1.476907
N	6.104292	-1.741601	0.586252
C	6.799369	-1.464403	1.832993
H	6.309934	-0.648048	2.375027
H	6.827580	-2.347761	2.483541
H	7.823778	-1.166840	1.608663
C	6.884696	-1.844129	-0.637658
H	7.109464	-2.887369	-0.895659
H	6.353422	-1.381911	-1.473777
H	7.825997	-1.309291	-0.500135
F	-0.735199	-1.931886	1.794420
H	0.972218	-2.919793	0.617148

$TS_{5\text{-}a}$

E(SMD/M06-2X/6-31g(d)) = -3849.503894 au

E(SMD/M06-2X/def2-TZVP//SMD/M06-2X/6-31g(d)) = -3850.965838 au

H(SMD/M06-2X/6-31g(d)) = -3848.834930 au

G(SMD/M06-2X/6-31g(d)) = -3848.985125 au

S	2.228801	2.928194	-0.762978
C	-1.046464	1.968869	-0.759745
C	-2.386420	2.655469	-0.786762
C	-2.101797	3.981754	-1.108704
C	-0.615616	4.077948	-1.283045
O	0.027318	5.068741	-1.545948
O	-0.778854	0.750683	-0.494199
N	-0.075176	2.801721	-1.078409
C	-4.704960	3.208233	-0.739826
C	-3.697485	2.242689	-0.607587
C	-3.083937	4.941573	-1.245142
C	-4.408594	4.534579	-1.048038
H	-5.739499	2.910195	-0.600600
H	-3.965495	1.218049	-0.384644
H	-2.834582	5.967306	-1.498892
H	-5.214011	5.256077	-1.143548
B	-1.625512	-0.376769	0.073735
C	-2.719013	-0.939208	-1.004374
C	-3.480466	-2.049580	-0.651730
C	-2.865462	-0.522877	-2.320554
C	-4.357654	-2.688631	-1.511119
C	-3.732989	-1.133788	-3.219418
C	-4.484125	-2.222823	-2.812293
C	-0.534915	-1.592059	0.227575
C	0.198713	-1.955889	-0.900288

C	-0.308218	-2.376853	1.349459
C	1.077305	-3.027141	-0.934347
C	0.587748	-3.439242	1.365930
C	1.282646	-3.771238	0.215889
C	-2.237937	0.136730	1.505001
C	-1.340291	0.626124	2.451577
C	-3.565438	0.156607	1.915242
C	-1.705598	1.112589	3.695457
C	-3.982377	0.639499	3.151112
C	-3.046271	1.119659	4.049668
F	-0.025426	0.611542	2.180514
F	-0.785527	1.558436	4.554504
F	-4.555210	-0.268277	1.108124
F	-5.277928	0.644512	3.473093
F	-3.426380	1.584211	5.238499
F	-2.162590	0.516703	-2.800909
F	-3.841126	-0.684477	-4.472629
F	-5.317752	-2.822072	-3.661886
F	-3.370875	-2.549579	0.591284
F	-5.068950	-3.744645	-1.108841
F	0.047248	-1.277686	-2.048664
F	1.738753	-3.329936	-2.057605
F	2.156454	-4.780094	0.228283
F	0.796839	-4.135470	2.489194
F	-0.958976	-2.151070	2.501652
C	2.610632	1.278791	-0.451311
C	3.026351	0.406116	-1.482572
C	2.602868	0.770824	0.866542
C	3.478096	-0.868643	-1.210802
C	3.030166	-0.507357	1.155491
H	2.259799	1.410953	1.673857
C	3.548546	-1.342492	0.128676
H	3.789980	-1.504686	-2.030902
H	3.004413	-0.852054	2.182681
N	4.089635	-2.554713	0.420224
C	4.679689	-3.365351	-0.633668
H	5.126982	-4.252925	-0.187368
H	3.932093	-3.688194	-1.364708
H	5.464195	-2.810965	-1.161823
C	4.121809	-3.035218	1.791777
H	4.661470	-2.339574	2.445636
H	3.113466	-3.175550	2.200442
H	4.633226	-3.996792	1.816132
C	4.761186	3.349164	0.772208
C	4.396735	3.835470	-0.511816
C	5.462459	2.182513	0.916438
C	4.990440	3.214720	-1.642749
C	5.882513	1.446138	-0.238312
H	5.700814	1.819563	1.908829
C	5.698663	2.048260	-1.522686

H	6.093970	1.570866	-2.410194
N	6.436188	0.224351	-0.130512
C	6.624161	-0.420360	1.162506
H	6.727224	-1.495630	1.003618
H	7.520344	-0.048104	1.674333
H	5.751650	-0.253792	1.796953
C	7.012851	-0.419012	-1.304923
H	7.812418	0.192709	-1.737530
H	7.434507	-1.378720	-1.008166
H	6.247143	-0.591092	-2.068600
H	4.831516	3.646789	-2.626499
H	3.996472	4.840089	-0.606089
H	4.434604	3.890483	1.655614
H	2.999512	0.754635	-2.511459

$TS_{5\text{-}b}$

E(SMD/M06-2X/6-31g(d)) = -3849.496220 au

E(SMD/M06-2X/def2-TZVP//SMD/M06-2X/6-31g(d)) = -3850.961400 au

H(SMD/M06-2X/6-31g(d)) = -3848.827320 au

G(SMD/M06-2X/6-31g(d)) = -3848.981657 au

C	0.615845	-1.408523	-1.240190
C	1.324377	-2.726596	-1.181982
C	0.400320	-3.651374	-1.665650
C	-0.843272	-2.883599	-2.026393
O	-1.869879	-3.329452	-2.480398
O	1.045103	-0.258254	-0.904385
N	-0.595773	-1.529160	-1.746465
S	-1.827470	0.395131	-1.584901
C	2.925009	-4.472114	-0.956780
C	2.608090	-3.113260	-0.832731
C	0.703065	-4.991747	-1.796895
C	1.991063	-5.396446	-1.424175
H	3.920924	-4.808901	-0.686780
H	3.353834	-2.409266	-0.478523
H	-0.025962	-5.698335	-2.181412
H	2.271611	-6.441759	-1.508849
B	2.115111	0.224206	0.053002
C	3.560010	0.326257	-0.697492
C	4.618445	0.941970	-0.035912
C	3.831549	-0.061790	-2.002393
C	5.861523	1.162560	-0.603380
C	5.064740	0.141242	-2.614756
C	6.084162	0.759668	-1.912918
C	1.621079	1.756517	0.398651
C	1.123478	2.576711	-0.612493
C	1.656140	2.347495	1.657370
C	0.663513	3.868637	-0.398044
C	1.206271	3.637573	1.913920
C	0.697809	4.404369	0.879339
C	2.058059	-0.728277	1.381971

C	0.830412	-0.879097	2.023107
C	3.107120	-1.419034	1.973276
C	0.635642	-1.656732	3.152729
C	2.960564	-2.211772	3.105724
C	1.717358	-2.327035	3.704018
F	-0.251205	-0.238889	1.542542
F	-0.570241	-1.754494	3.717446
F	4.342408	-1.390994	1.443349
F	4.007280	-2.862462	3.618245
F	1.559377	-3.080796	4.790396
F	2.905540	-0.679974	-2.755165
F	5.268566	-0.253528	-3.874098
F	7.270348	0.962521	-2.485208
F	4.448101	1.338586	1.236342
F	6.839960	1.752666	0.087420
F	1.073266	2.147801	-1.883794
F	0.182853	4.595812	-1.412721
F	0.264493	5.644219	1.104351
F	1.260849	4.143178	3.149352
F	2.136879	1.684672	2.721005
C	-3.269335	-0.399856	-1.040768
C	-3.439607	-0.735657	0.315364
C	-4.316182	-0.707280	-1.928164
C	-4.612112	-1.303181	0.775571
H	-2.625328	-0.553262	1.011286
C	-5.497607	-1.268075	-1.483650
H	-4.191939	-0.495537	-2.986835
C	-5.701468	-1.527912	-0.104489
H	-4.693468	-1.558522	1.825380
H	-6.275237	-1.490473	-2.204254
N	-6.907991	-1.975463	0.359190
C	-7.024515	-2.431583	1.734332
H	-8.065799	-2.685707	1.933882
H	-6.403206	-3.315621	1.932762
H	-6.732322	-1.641412	2.432702
C	-7.931693	-2.387661	-0.587037
H	-8.193663	-1.565370	-1.260474
H	-7.614236	-3.246062	-1.194497
H	-8.828677	-2.666223	-0.033819
C	-2.568831	2.555233	-1.357115
C	-3.737237	2.571495	-2.172063
C	-2.758503	2.553687	0.055832
C	-4.973594	2.323777	-1.644558
H	-3.630900	2.731379	-3.241253
C	-3.985057	2.304467	0.606101
H	-1.892394	2.678667	0.702001
C	-5.119430	2.075577	-0.240085
H	-5.840017	2.300189	-2.293627
H	-4.088964	2.250009	1.682514
N	-6.283689	1.643751	0.269309

C	-7.449603	1.445519	-0.583240
H	-7.172761	0.885366	-1.478933
H	-8.190547	0.866922	-0.031288
H	-7.896161	2.401599	-0.881881
C	-6.454014	1.496313	1.710207
H	-6.404185	2.466409	2.217763
H	-7.429551	1.050939	1.903717
H	-5.681690	0.840102	2.121120
H	-1.642786	2.951839	-1.763379

TS_{5-c}

E(SMD/M06-2X/6-31g(d)) = -3849.493525 au

E(SMD/M06-2X/def2-TZVP//SMD/M06-2X/6-31g(d)) = -3850.958280 au

H(SMD/M06-2X/6-31g(d)) = -3848.823743 au

G(SMD/M06-2X/6-31g(d)) = -3848.975908 au

C	-0.089921	-1.710952	0.800498
C	0.736933	-2.932386	1.079331
C	-0.059793	-3.725303	1.904599
C	-1.351469	-2.985248	2.109965
O	-2.292460	-3.326839	2.786061
O	0.205865	-0.687634	0.100457
N	-1.275132	-1.792961	1.371321
S	-2.543425	0.040863	1.656120
C	2.414513	-4.622052	1.122777
C	1.983099	-3.371876	0.661256
C	0.353001	-4.960377	2.363167
C	1.619560	-5.401857	1.961958
H	3.388155	-4.989802	0.814605
H	2.618557	-2.791195	0.002339
H	-0.283025	-5.563839	3.003393
H	1.985697	-6.365705	2.301316
B	1.543757	-0.093284	-0.299398
C	2.135932	-0.802653	-1.644860
C	3.270524	-0.259084	-2.240149
C	1.561062	-1.860880	-2.335400
C	3.823965	-0.728503	-3.418877
C	2.084575	-2.369124	-3.520077
C	3.219549	-1.797324	-4.066967
C	1.151109	1.444463	-0.720875
C	0.075218	1.659819	-1.579326
C	1.841192	2.590871	-0.346684
C	-0.309703	2.914632	-2.029327
C	1.490287	3.865529	-0.774450
C	0.415385	4.026272	-1.630130
C	2.509963	-0.146224	1.021023
C	2.017446	0.388584	2.210231
C	3.786745	-0.683620	1.114279
C	2.713898	0.383738	3.407926
C	4.522061	-0.713727	2.294059
C	3.983432	-0.173264	3.448639

F	0.800482	0.958289	2.227118
F	2.184397	0.916398	4.511843
F	4.379766	-1.257907	0.052054
F	5.737626	-1.264473	2.321458
F	4.672019	-0.194280	4.588575
F	0.462835	-2.480161	-1.871187
F	1.500632	-3.403108	-4.131277
F	3.734771	-2.272436	-5.200438
F	3.891747	0.769561	-1.638048
F	4.921191	-0.170840	-3.936905
F	-0.632494	0.619123	-2.048320
F	-1.352500	3.060375	-2.854580
F	0.085063	5.245180	-2.064448
F	2.179983	4.936991	-0.371598
F	2.914185	2.526160	0.456679
C	-3.830802	-0.774676	0.752003
C	-4.861786	-1.454755	1.404448
C	-3.809070	-0.773544	-0.646519
C	-5.856571	-2.102099	0.686419
H	-4.879964	-1.489453	2.490243
C	-4.789856	-1.424099	-1.377425
H	-3.001037	-0.263867	-1.165525
C	-5.852595	-2.097168	-0.727827
H	-6.634116	-2.625050	1.229899
H	-4.727435	-1.412333	-2.458925
N	-6.844078	-2.721525	-1.445108
C	-7.783867	-3.582253	-0.750747
H	-8.492711	-3.987176	-1.473316
H	-7.285272	-4.419820	-0.242647
H	-8.352962	-3.018246	-0.004380
C	-6.685063	-2.887554	-2.878533
H	-6.612168	-1.916235	-3.378615
H	-5.791688	-3.474754	-3.134327
H	-7.562141	-3.400966	-3.273463
C	-3.720461	1.799622	2.073688
C	-2.730900	2.593864	2.753268
C	-4.133216	2.278746	0.784447
C	-2.088718	3.630525	2.147295
H	-2.453177	2.319003	3.767601
C	-3.526968	3.333926	0.169340
H	-4.932295	1.754057	0.268481
C	-2.476896	4.053506	0.829495
H	-1.326112	4.175770	2.688865
H	-3.843269	3.629336	-0.822717
N	-1.904092	5.106603	0.237715
C	-0.806365	5.835382	0.867339
H	-0.090504	5.140107	1.309429
H	-0.289701	6.411951	0.100339
H	-1.175078	6.516988	1.642427
C	-2.500671	5.681784	-0.968656

H	-3.549489	5.936668	-0.788726
H	-1.955100	6.587048	-1.227385
H	-2.439347	4.984172	-1.807751
H	-4.469797	1.303119	2.686213

$TS_{5\text{-}d}$

E(SMD/M06-2X/6-31g(d)) = -3849.493040 au

E(SMD/M06-2X/def2-TZVP//SMD/M06-2X/6-31g(d)) = -3850.956798 au

H(SMD/M06-2X/6-31g(d)) = -3848.823824 au

G(SMD/M06-2X/6-31g(d)) = -3848.975343 au

C	-0.272245	-1.534826	-0.924339
C	0.208760	-2.962134	-0.952799
C	-0.879929	-3.700686	-1.412182
C	-1.990409	-2.728917	-1.666128
O	-3.106062	-2.981100	-2.059465
O	0.358554	-0.489219	-0.562629
N	-1.516158	-1.444162	-1.359880
S	-3.268289	0.007014	-1.363726
C	1.474634	-4.980068	-0.847518
C	1.411335	-3.591334	-0.670567
C	-0.831321	-5.067906	-1.593392
C	0.376000	-5.710163	-1.296354
H	2.405791	-5.494516	-0.631515
H	2.290598	-3.057606	-0.335593
H	-1.695495	-5.616179	-1.956072
H	0.461964	-6.784833	-1.423176
B	1.694593	-0.292435	0.145929
C	2.969157	-0.654475	-0.811015
C	4.249382	-0.429111	-0.314328
C	2.917038	-1.020284	-2.149278
C	5.406254	-0.593831	-1.057201
C	4.050774	-1.196199	-2.934570
C	5.303154	-0.982264	-2.385440
C	1.762464	1.334171	0.331276
C	1.607107	2.123981	-0.806404
C	2.026232	2.038618	1.497837
C	1.715435	3.505082	-0.805680
C	2.112588	3.425485	1.546400
C	1.961270	4.165092	0.386993
C	1.610488	-1.109213	1.564692
C	0.543798	-0.814703	2.412011
C	2.468743	-2.089852	2.046848
C	0.314776	-1.443881	3.624018
C	2.278180	-2.753200	3.254446
C	1.193855	-2.428070	4.049960
F	-0.318111	0.157602	2.073133
F	-0.726803	-1.104988	4.387944
F	3.544083	-2.487224	1.342160
F	3.132371	-3.700754	3.648122
F	0.996034	-3.052051	5.209871

F	1.742160	-1.236133	-2.765076
F	3.939117	-1.557796	-4.215311
F	6.399662	-1.142068	-3.125321
F	4.393913	-0.023796	0.959092
F	6.607908	-0.368484	-0.520192
F	1.366529	1.549442	-1.995585
F	1.551330	4.201711	-1.935276
F	2.022016	5.497228	0.426417
F	2.327990	4.054201	2.707727
F	2.222593	1.406340	2.665882
C	-2.371838	1.449879	-0.908467
C	-1.993071	2.390435	-1.877816
C	-2.152178	1.764812	0.439697
C	-1.480356	3.624558	-1.519570
H	-2.133919	2.158656	-2.930336
C	-1.618265	2.985057	0.817200
H	-2.426071	1.044877	1.205305
C	-1.310950	3.970406	-0.154466
H	-1.219221	4.330661	-2.298718
H	-1.468584	3.188717	1.871083
N	-0.862158	5.207038	0.211844
C	-0.686801	6.246246	-0.788887
H	-0.332765	7.151847	-0.296908
H	0.054982	5.957376	-1.538831
H	-1.629900	6.475374	-1.302449
C	-0.775753	5.555556	1.618194
H	-1.748119	5.463096	2.119056
H	-0.057742	4.917793	2.148715
H	-0.437203	6.587247	1.707794
C	-5.218654	1.090585	-1.492644
C	-5.984391	0.095151	-2.178946
C	-5.495786	1.267827	-0.102227
C	-6.794269	-0.775638	-1.509678
H	-5.867763	-0.004427	-3.254494
C	-6.316762	0.418361	0.587007
H	-4.997596	2.074440	0.428773
C	-6.973960	-0.656946	-0.090272
H	-7.315279	-1.550648	-2.057148
H	-6.459064	0.558492	1.650902
N	-7.741078	-1.528624	0.578406
C	-8.402686	-2.625171	-0.121487
H	-7.670550	-3.250727	-0.641364
H	-8.928737	-3.239055	0.607381
H	-9.128096	-2.246696	-0.849018
C	-7.927119	-1.395454	2.019354
H	-8.414612	-0.446714	2.266529
H	-8.556981	-2.211624	2.368517
H	-6.965705	-1.447338	2.540191
H	-4.839658	1.933605	-2.062435

TS$_{5-e}$

E(SMD/M06-2X/6-31g(d)) = -3849.493983 au
E(SMD/M06-2X/def2-TZVP//SMD/M06-2X/6-31g(d)) = -3850.957213 au
H(SMD/M06-2X/6-31g(d)) = -3848.824601 au
G(SMD/M06-2X/6-31g(d)) = -3848.974842 au

C	-0.566515	-1.586889	0.285569
C	-0.178144	-2.704576	1.209161
C	-1.382744	-3.318321	1.548148
C	-2.476622	-2.579637	0.831425
O	-3.659628	-2.816604	0.874653
O	0.178346	-0.722788	-0.286308
N	-1.870134	-1.553966	0.082880
S	-2.598675	0.427861	-0.742790
C	0.971660	-4.297272	2.557516
C	1.024009	-3.189434	1.701134
C	-1.447023	-4.412399	2.386774
C	-0.238138	-4.899391	2.898248
H	1.898245	-4.694788	2.959427
H	1.982451	-2.755005	1.446431
H	-2.397961	-4.874896	2.633235
H	-0.240454	-5.757870	3.562542
B	1.651810	-0.395186	-0.148705
C	1.857044	0.909872	-1.131161
C	1.286773	0.910562	-2.402139
C	2.622460	2.034818	-0.842333
C	1.418599	1.956677	-3.304906
C	2.781103	3.103936	-1.717072
C	2.170555	3.069105	-2.959546
C	1.913900	0.017207	1.414196
C	1.121610	1.036759	1.936995
C	2.900676	-0.443309	2.275816
C	1.306383	1.600186	3.188218
C	3.109208	0.074338	3.549468
C	2.313030	1.111204	4.006401
C	2.602412	-1.562632	-0.782239
C	2.165438	-2.705107	-1.440618
C	3.978260	-1.352269	-0.826588
C	3.026711	-3.600413	-2.065861
C	4.872661	-2.216456	-1.435128
C	4.388586	-3.355436	-2.062823
F	0.859258	-3.015185	-1.508853
F	2.547426	-4.687772	-2.674166
F	4.491422	-0.246155	-0.261338
F	6.183233	-1.962211	-1.437252
F	5.226961	-4.200110	-2.661270
F	3.248937	2.171135	0.339573
F	3.494534	4.173639	-1.353194
F	2.303270	4.086400	-3.809076
F	0.569670	-0.140733	-2.824175
F	0.805123	1.910990	-4.491114

F	0.145626	1.573614	1.184594
F	0.542091	2.617761	3.598314
F	2.509770	1.628719	5.217538
F	4.075266	-0.413730	4.330723
F	3.717940	-1.450597	1.919704
C	-4.284616	-0.064984	-0.642883
C	-5.019058	0.132551	0.533568
C	-4.915415	-0.697252	-1.721269
C	-6.337262	-0.272880	0.635118
H	-4.542329	0.612875	1.384598
C	-6.232050	-1.115525	-1.637416
H	-4.359770	-0.870799	-2.639120
C	-6.983135	-0.911847	-0.453482
H	-6.869564	-0.100547	1.562832
H	-6.682416	-1.605677	-2.491865
N	-8.284075	-1.315278	-0.365191
C	-9.026753	-1.111982	0.864380
H	-10.031587	-1.515624	0.742488
H	-8.552398	-1.624401	1.710416
H	-9.112328	-0.046386	1.111961
C	-8.919685	-1.965478	-1.495669
H	-8.928293	-1.316250	-2.379969
H	-8.410584	-2.900220	-1.762245
H	-9.951636	-2.200254	-1.236114
C	-3.016987	2.471287	-1.501159
C	-1.661189	2.798746	-1.827751
C	-3.552716	3.071173	-0.318812
C	-0.848239	3.434056	-0.935770
H	-1.255652	2.451529	-2.774897
C	-2.764870	3.745455	0.572394
H	-4.608795	2.937257	-0.100665
C	-1.365302	3.903237	0.318570
H	0.196896	3.587777	-1.172479
H	-3.201139	4.141941	1.480069
N	-0.559438	4.472677	1.223834
C	0.871323	4.609170	0.972684
H	1.058222	5.171159	0.051991
H	1.322833	5.151031	1.802214
H	1.350205	3.628063	0.891858
C	-1.090790	4.899527	2.514358
H	-1.569179	4.062184	3.030980
H	-0.269726	5.261956	3.129848
H	-1.819884	5.706499	2.389231
H	-3.695247	2.201580	-2.305198

$TS_{5\text{-}f}$

E(SMD/M06-2X/6-31g(d)) = -3849.492983 au

E(SMD/M06-2X/def2-TZVP//SMD/M06-2X/6-31g(d)) = -3850.956174 au

H(SMD/M06-2X/6-31g(d)) = -3848.823800 au

G(SMD/M06-2X/6-31g(d)) = -3848.973662 au

C	-0.717552	-1.802514	-0.476393
C	-1.691236	-2.188263	-1.560572
C	-1.447463	-3.537492	-1.806151
C	-0.330203	-3.950345	-0.898636
O	0.169470	-5.046207	-0.807034
O	-0.581838	-0.664026	0.090777
N	0.048981	-2.825246	-0.141875
S	2.192704	-2.986046	0.628183
C	-3.352262	-2.220904	-3.270912
C	-2.640834	-1.501738	-2.302686
C	-2.140710	-4.259633	-2.757419
C	-3.114332	-3.576513	-3.493988
H	-4.104985	-1.706108	-3.859738
H	-2.848115	-0.448353	-2.158948
H	-1.929689	-5.310811	-2.928207
H	-3.684260	-4.102509	-4.253439
B	-1.635573	0.439164	0.281880
C	-1.040299	1.299509	1.557257
C	0.130798	2.037096	1.377674
C	-1.569432	1.378220	2.842262
C	0.756884	2.758231	2.383377
C	-0.976218	2.092436	3.877948
C	0.201024	2.781985	3.651778
C	-1.659332	1.457860	-0.998663
C	-0.871120	1.352655	-2.137240
C	-2.389600	2.636913	-0.900272
C	-0.803453	2.341508	-3.111056
C	-2.363045	3.644510	-1.850921
C	-1.553706	3.496216	-2.967810
C	-3.059069	-0.307688	0.607731
C	-4.286437	-0.087064	-0.004737
C	-3.067329	-1.329780	1.556202
C	-5.433150	-0.819636	0.283764
C	-4.182146	-2.086713	1.875881
C	-5.382180	-1.826356	1.230651
F	-4.429045	0.829420	-0.978145
F	-6.577229	-0.564211	-0.355671
F	-1.941622	-1.613663	2.230776
F	-4.118134	-3.046775	2.800495
F	-6.470608	-2.537546	1.518620
F	-1.538527	2.116391	5.088712
F	0.782850	3.471653	4.631352
F	0.712581	2.094888	0.169400
F	1.886841	3.435796	2.143932
F	-0.104468	0.270965	-2.354174
F	-0.005460	2.192838	-4.173597
F	-1.500601	4.454514	-3.892523
F	-3.089749	4.754329	-1.698033
F	-3.166941	2.837374	0.177009
C	2.667322	-1.435293	0.057198

C	2.786625	-0.333949	0.929407
C	2.961501	-1.230364	-1.305709
C	3.202934	0.898371	0.476302
H	2.539289	-0.463627	1.979725
C	3.344189	0.006062	-1.781465
H	2.863304	-2.062476	-1.998347
C	3.501252	1.104559	-0.896032
H	3.255913	1.723700	1.175487
H	3.527648	0.125981	-2.842132
N	3.913909	2.321871	-1.354106
C	3.852982	3.476897	-0.470895
H	4.263497	4.340566	-0.994050
H	2.823962	3.701513	-0.164908
H	4.454874	3.310213	0.427913
C	3.970293	2.561238	-2.788088
H	4.681611	1.882622	-3.269476
H	2.988181	2.431665	-3.263991
H	4.310625	3.582181	-2.961128
C	4.370505	-3.708863	1.294552
C	4.730686	-2.687197	2.211613
C	5.424491	-1.579123	1.802245
H	4.404656	-2.763141	3.245080
C	5.818730	-1.440750	0.433195
H	5.646235	-0.793826	2.513991
C	5.654490	-2.571556	-0.428225
C	4.966521	-3.673645	0.005464
H	6.059027	-2.553302	-1.432577
H	4.822519	-4.514028	-0.667436
N	6.329492	-0.283746	-0.027754
C	6.609028	0.819420	0.882437
H	5.725680	1.056739	1.480147
H	7.442083	0.582355	1.555287
H	6.873972	1.698074	0.295005
C	6.792773	-0.169090	-1.404588
H	7.726633	-0.722602	-1.562725
H	6.034070	-0.545785	-2.095104
H	6.967797	0.883673	-1.627526
F	-2.717210	0.767188	3.172507
H	3.966742	-4.643854	1.670132

TS$_{5\text{-}g}$

E(SMD/M06-2X/6-31g(d)) = -3849.485196 au

E(SMD/M06-2X/def2-TZVP//SMD/M06-2X/6-31g(d)) = -3850.949506 au

H(SMD/M06-2X/6-31g(d)) = -3848.816534 au

G(SMD/M06-2X/6-31g(d)) = -3848.964646 au

C	0.388363	1.067674	-0.911842
C	-0.172234	1.931423	-2.012134
C	0.937174	2.540944	-2.594835
C	2.147559	2.041757	-1.869228
O	3.299248	2.334374	-2.084631

O	-0.237998	0.309157	-0.098772
N	1.706650	1.150702	-0.874050
S	3.256848	0.119383	0.345361
C	-1.559383	3.047120	-3.599352
C	-1.442796	2.165477	-2.517086
C	0.838170	3.408571	-3.663738
C	-0.443644	3.663911	-4.162904
H	-2.544833	3.249227	-4.007299
H	-2.332183	1.699372	-2.112387
H	1.721979	3.866538	-4.097153
H	-0.572045	4.343316	-4.999648
B	-1.701374	0.365420	0.355492
C	-2.688285	-0.374510	-0.718005
C	-4.027296	-0.547014	-0.386455
C	-2.289566	-1.007061	-1.888627
C	-4.928762	-1.261116	-1.159723
C	-3.155534	-1.738873	-2.691404
C	-4.484528	-1.867971	-2.325207
C	-1.703290	-0.575170	1.707297
C	-1.446463	-1.939950	1.566563
C	-1.941963	-0.168933	3.015895
C	-1.395038	-2.831786	2.627355
C	-1.902118	-1.030180	4.107170
C	-1.621119	-2.370681	3.914498
C	-2.037543	1.944108	0.652680
C	-3.102777	2.692715	0.168155
C	-1.138801	2.668783	1.435994
C	-3.274088	4.048436	0.428333
C	-1.267986	4.017229	1.722957
C	-2.352128	4.716010	1.213258
F	-4.028601	2.152863	-0.644232
F	-4.316259	4.707755	-0.083100
F	-0.080124	2.045163	1.977657
F	-0.373022	4.645702	2.488511
F	-2.498338	6.013705	1.473297
F	-1.013300	-0.954447	-2.306677
F	-2.707525	-2.335579	-3.800775
F	-5.330035	-2.566048	-3.082898
F	-4.496763	-0.001126	0.747913
F	-6.206678	-1.384179	-0.791798
F	-1.249732	-2.465039	0.347005
F	-1.142758	-4.130549	2.425930
F	-1.580437	-3.209003	4.948964
F	-2.137684	-0.573198	5.339720
F	-2.241102	1.104706	3.314882
C	2.533103	-1.425730	-0.151012
C	2.463683	-1.758327	-1.505896
C	1.978672	-2.304675	0.781556
C	1.802794	-2.901957	-1.925428
H	2.898597	-1.089879	-2.244648

C	1.371459	-3.483739	0.378892
H	2.006844	-2.059524	1.840709
C	1.228243	-3.792552	-0.991393
H	1.731574	-3.100575	-2.988117
H	0.932536	-4.124450	1.133470
N	0.542056	-4.915682	-1.407579
C	0.067606	-4.953127	-2.781956
H	-0.523087	-5.858606	-2.925144
H	-0.556655	-4.081463	-3.029698
H	0.904033	-4.989648	-3.486587
C	-0.233883	-5.649173	-0.421012
H	0.418879	-6.054933	0.358356
H	-0.997338	-5.022257	0.058768
H	-0.724143	-6.489848	-0.913391
C	4.857559	-0.838892	1.484831
C	5.476409	0.316302	2.071800
C	5.586361	-1.481670	0.430829
C	6.578831	0.899052	1.523390
H	5.011506	0.770348	2.942748
C	6.702784	-0.925726	-0.125444
H	5.201105	-2.415132	0.029485
C	7.220336	0.310542	0.379456
H	6.983922	1.800436	1.965122
H	7.190096	-1.420763	-0.955382
N	8.276421	0.900346	-0.190123
C	8.788525	2.168143	0.323616
H	9.166874	2.053944	1.344430
H	9.605073	2.500560	-0.314577
H	8.005309	2.932338	0.315459
C	8.935234	0.288246	-1.340601
H	8.232656	0.171091	-2.171532
H	9.752140	0.931952	-1.661314
H	9.344378	-0.692510	-1.078577
H	4.233599	-1.454902	2.126111

TS_{5-h}

E(SMD/M06-2X/6-31g(d)) = -3849.489042 au

E(SMD/M06-2X/def2-TZVP//SMD/M06-2X/6-31g(d)) = -3850.947441 au

H(SMD/M06-2X/6-31g(d)) = -3848.820015 au

G(SMD/M06-2X/6-31g(d)) = -3848.967449 au

S	1.900334	2.535417	-1.370021
C	-1.256632	1.753254	-1.252079
C	-2.367114	2.675340	-0.845412
C	-2.139768	3.845979	-1.567042
C	-0.886760	3.623743	-2.362647
O	-0.326295	4.433007	-3.066922
O	-1.019256	0.567264	-0.854533
N	-0.437591	2.314092	-2.127328
C	-4.270797	3.698688	0.154265
C	-3.428487	2.584396	0.038642

C	-2.960002	4.950561	-1.461950
C	-4.047182	4.859774	-0.583815
H	-5.113277	3.654458	0.837530
H	-3.611531	1.700976	0.634267
H	-2.761855	5.853195	-2.032025
H	-4.720630	5.703633	-0.469728
B	-1.623609	-0.518714	0.006825
C	-3.081397	-0.969367	-0.660357
C	-4.357867	-0.724115	-0.145921
C	-3.115264	-1.629543	-1.892086
C	-5.542522	-1.103382	-0.765057
C	-4.272771	-2.030299	-2.545949
C	-5.505395	-1.767181	-1.977066
C	-0.469082	-1.718446	-0.145070
C	0.363291	-1.838760	-1.271245
C	-0.203103	-2.677054	0.832911
C	1.300528	-2.851497	-1.435974
C	0.760158	-3.673958	0.723907
C	1.515348	-3.772441	-0.426468
C	-1.738443	-0.006510	1.569283
C	-1.091833	1.123772	2.062539
C	-2.489670	-0.688238	2.528458
C	-1.175747	1.554675	3.383011
C	-2.602458	-0.296174	3.851404
C	-1.939279	0.841037	4.286645
F	-0.321107	1.900697	1.274677
F	-0.527229	2.653822	3.776913
F	-3.168299	-1.795880	2.196011
F	-3.344096	-0.999446	4.711101
F	-2.038093	1.234465	5.555255
F	-1.983359	-1.921508	-2.547130
F	-4.201338	-2.660501	-3.720371
F	-6.629872	-2.137723	-2.584225
F	-4.553071	-0.069495	1.014585
F	-6.717078	-0.823132	-0.195702
F	0.320261	-0.984989	-2.300116
F	2.031550	-2.924578	-2.554861
F	2.450057	-4.717208	-0.546522
F	0.967608	-4.527998	1.732801
F	-0.871806	-2.704055	1.996050
C	2.513658	1.045062	-0.965509
C	3.294893	0.293428	-1.909582
C	2.352017	0.471097	0.344198
C	3.898046	-0.879712	-1.575220
C	2.945893	-0.706527	0.691850
H	1.724782	0.995764	1.057882
C	3.773266	-1.412256	-0.246863
H	4.485721	-1.405054	-2.317321
H	2.775244	-1.120224	1.678723
N	4.380791	-2.557930	0.079772

C	5.074000	-3.351449	-0.935842
H	5.349840	-4.309232	-0.497549
H	4.418242	-3.537258	-1.789763
H	5.981037	-2.843217	-1.278429
C	4.310509	-3.099359	1.433545
H	4.436406	-2.307170	2.173097
H	3.356532	-3.608982	1.613302
H	5.118757	-3.818790	1.561807
C	4.035742	3.670751	0.660359
C	4.202895	4.050184	-0.678245
C	4.628472	2.524626	1.156798
C	5.064705	3.295632	-1.486515
C	5.422429	1.701043	0.317077
H	4.467359	2.255569	2.193571
C	5.665189	2.146619	-1.009421
H	6.318785	1.581514	-1.663100
N	5.921139	0.510098	0.770755
C	5.872922	0.220283	2.195241
H	6.301079	-0.767983	2.366345
H	6.441253	0.954199	2.783196
H	4.839629	0.212376	2.554530
C	6.890116	-0.227415	-0.024065
H	7.855590	0.293183	-0.091600
H	7.049931	-1.203125	0.439440
H	6.509448	-0.393082	-1.034942
H	5.250589	3.603602	-2.511644
H	3.750513	4.961508	-1.055662
H	3.413663	4.272797	1.316464
H	3.407454	0.692460	-2.913504

TS_6

E(SMD/M06-2X/6-31g(d)) = -3849.505700 au

E(SMD/M06-2X/def2-TZVP//SMD/M06-2X/6-31g(d)) = -3850.967362 au

H(SMD/M06-2X/6-31g(d)) = -3848.840271 au

G(SMD/M06-2X/6-31g(d)) = -3848.995029 au

C	0.626425	-0.518063	-0.910271
C	0.541703	-0.121831	-2.359058
C	1.650759	-0.720657	-2.957264
C	2.358712	-1.495312	-1.885933
O	3.348961	-2.179857	-2.008669
O	-0.142752	-0.195771	0.058491
N	1.656899	-1.308472	-0.688146
S	3.089267	-2.690100	2.646730
C	-0.093961	0.735110	-4.491402
C	-0.360234	0.603485	-3.121729
C	1.923216	-0.600430	-4.304712
C	1.029523	0.152962	-5.074953
H	-0.783062	1.303280	-5.108380
H	-1.253228	1.051449	-2.706198
H	2.792935	-1.077494	-4.746061

H	1.205684	0.277120	-6.138911
B	-1.253437	0.845939	0.166709
C	-2.602342	0.374829	-0.622809
C	-3.735192	1.179083	-0.543502
C	-2.798343	-0.851474	-1.244039
C	-4.966675	0.830951	-1.073077
C	-4.016879	-1.246767	-1.782823
C	-5.107966	-0.399000	-1.700775
C	-1.638551	0.795877	1.761546
C	-1.939597	-0.440901	2.324511
C	-1.729038	1.869224	2.638966
C	-2.249402	-0.630419	3.662043
C	-2.048696	1.728397	3.984750
C	-2.304650	0.470180	4.503047
C	-0.601678	2.279926	-0.285677
C	0.569883	2.676769	0.356213
C	-1.041529	3.160760	-1.265584
C	1.276572	3.826328	0.047147
C	-0.366679	4.327232	-1.610922
C	0.804309	4.661455	-0.954079
F	1.058503	1.930180	1.360564
F	2.402432	4.135166	0.698750
F	-2.151161	2.909661	-1.982685
F	-0.835892	5.118561	-2.578071
F	1.465178	5.772192	-1.272994
F	-1.797840	-1.745393	-1.354786
F	-4.141399	-2.441983	-2.368104
F	-6.282730	-0.758272	-2.215822
F	-3.653687	2.361804	0.087971
F	-6.015200	1.651135	-0.975727
F	-1.944673	-1.543697	1.552861
F	-2.500432	-1.853552	4.137229
F	-2.606714	0.320778	5.792032
F	-2.111114	2.797975	4.781965
F	-1.501767	3.125768	2.226275
C	3.952610	-1.354556	1.839703
C	3.380755	-0.081546	1.764740
C	5.198371	-1.561543	1.243701
C	4.023117	0.956241	1.108739
H	2.403946	0.093655	2.206877
C	5.858704	-0.531091	0.587813
H	5.659718	-2.544664	1.286091
C	5.287810	0.759028	0.506053
H	3.537051	1.923461	1.062949
H	6.819556	-0.735051	0.130432
N	5.935183	1.788153	-0.132400
C	5.231984	3.037684	-0.352264
H	5.882425	3.717988	-0.902549
H	4.307148	2.896174	-0.929940
H	4.969597	3.515073	0.598112

C	7.140981	1.510331	-0.888411
H	7.928619	1.112911	-0.238422
H	6.967299	0.787368	-1.698174
H	7.507584	2.438999	-1.326507
C	1.955287	-3.214235	1.322694
C	2.416724	-4.236452	0.394891
C	0.538387	-3.262270	1.640615
C	1.554506	-4.969356	-0.352576
H	3.486728	-4.331305	0.231296
C	-0.339202	-3.977513	0.892001
H	0.166861	-2.616463	2.431323
C	0.131381	-4.822039	-0.174367
H	1.939612	-5.666053	-1.086017
H	-1.398481	-3.906313	1.099349
N	-0.723685	-5.449988	-0.972412
C	-0.242248	-6.313649	-2.051576
H	0.303544	-7.170782	-1.646810
H	-1.098350	-6.676910	-2.616333
H	0.411901	-5.754110	-2.725925
C	-2.167514	-5.269856	-0.808636
H	-2.495111	-5.629148	0.170749
H	-2.432375	-4.214436	-0.917878
H	-2.679925	-5.840611	-1.580082
H	1.901217	-2.269277	0.540406

5a

E(SMD/M06-2X/6-31g(d)) = -730.932326 au

E(SMD/M06-2X/def2-TZVP//SMD/M06-2X/6-31g(d)) = -731.202831 au

H(SMD/M06-2X/6-31g(d)) = -730.581874 au

G(SMD/M06-2X/6-31g(d)) = -730.645160 au

C	-1.408784	2.209152	-0.547946
C	-0.740659	1.049863	-0.137345
C	-2.790065	2.173985	-0.710916
C	-1.468332	-0.119376	0.098616
C	-3.518487	1.013571	-0.479396
H	-3.317924	3.069972	-1.026568
C	-2.865133	-0.172091	-0.081866
H	-0.933923	-0.992883	0.453506
H	-4.593804	1.033354	-0.608944
N	-3.570012	-1.347293	0.123463
C	-2.903265	-2.441310	0.805243
H	-3.598667	-3.276943	0.893282
H	-2.038853	-2.787455	0.230020
H	-2.557918	-2.164738	1.813012
C	-5.014648	-1.272063	0.246426
H	-5.336057	-0.620887	1.073338
H	-5.465610	-0.900480	-0.678867
H	-5.405720	-2.275200	0.422058
C	0.734697	1.059858	0.063675
C	1.366792	2.184365	0.606771

C	1.494780	-0.062537	-0.275728
C	2.743346	2.158516	0.806609
C	2.891070	-0.095964	-0.087931
H	0.987367	-0.913486	-0.714994
C	3.503523	1.043007	0.475977
H	4.571443	1.061296	0.657210
N	3.634992	-1.205139	-0.457541
C	2.930244	-2.442524	-0.739002
H	3.660549	-3.220888	-0.963343
H	2.310825	-2.776986	0.107116
H	2.282972	-2.332733	-1.614935
C	4.997057	-1.315112	0.032554
H	5.054122	-1.308739	1.131702
H	5.427697	-2.248528	-0.332095
H	5.615217	-0.495613	-0.347096
H	3.239194	3.020179	1.245482
H	-0.852024	3.117780	-0.754595
H	0.783447	3.051961	0.898805

6aa

E(SMD/M06-2X/6-31g(d)) = -1127.908856 au

E(SMD/M06-2X/def2-TZVP//SMD/M06-2X/6-31g(d)) = -1128.211012 au

H(SMD/M06-2X/6-31g(d)) = -1127.578696 au

G(SMD/M06-2X/6-31g(d)) = -1127.642657 au

S	0.000010	2.996744	0.059153
C	-1.252554	1.755845	-0.003282
C	-0.730095	0.454755	-0.058615
C	-2.631584	1.954298	0.014646
C	-1.586800	-0.647997	-0.098770
C	-3.478796	0.858178	-0.028224
H	-3.051365	2.954580	0.068129
C	-2.979101	-0.467802	-0.097968
H	-1.157785	-1.642425	-0.131013
H	-4.546832	1.036234	-0.006757
N	-3.845116	-1.550042	-0.174329
C	-3.298702	-2.874651	0.055669
H	-4.104206	-3.606827	-0.014419
H	-2.556245	-3.126025	-0.708571
H	-2.822650	-2.971147	1.043614
C	-5.240397	-1.344052	0.172681
H	-5.373036	-0.963560	1.196748
H	-5.714089	-0.641036	-0.519218
H	-5.766921	-2.295271	0.084440
C	0.730038	0.454711	-0.058702
C	1.252538	1.755784	-0.003522
C	1.586799	-0.647994	-0.098807
C	2.631543	1.954325	0.014214
C	2.979099	-0.467783	-0.098310
H	1.157803	-1.642441	-0.130644
C	3.478782	0.858217	-0.028747

H	4.546813	1.036304	-0.007431
N	3.845212	-1.550032	-0.174730
C	3.298868	-2.874597	0.055713
H	4.104229	-3.606883	-0.015173
H	2.823645	-2.971102	1.044035
H	2.555870	-3.125952	-0.707974
C	5.240243	-1.344024	0.173424
H	5.371958	-0.962899	1.197364
H	5.766715	-2.295357	0.086206
H	5.714707	-0.641496	-0.518455
H	3.051262	2.954631	0.067683

add$_{1'\text{-}N}$

E(SMD/M06-2X/6-31g(d)) = -2938.465483 au

E(SMD/M06-2X/def2-TZVP//SMD/M06-2X/6-31g(d)) = -2939.693115 au

H(SMD/M06-2X/6-31g(d)) = -2937.924124 au

G(SMD/M06-2X/6-31g(d)) = -2938.046496 au

C	-5.046428	-1.666509	0.981497
C	-3.957302	-0.808667	1.169498
C	-6.078136	-1.273351	0.135437
C	-3.920347	0.423675	0.512726
C	-6.045287	-0.053795	-0.528475
H	-6.921888	-1.938018	-0.028584
C	-4.958484	0.828896	-0.350832
H	-3.081722	1.086695	0.700773
H	-6.862610	0.206817	-1.189856
N	-4.911903	2.052268	-0.993650
C	-3.641121	2.751890	-1.041487
H	-3.732008	3.616379	-1.700734
H	-3.359209	3.120506	-0.049020
H	-2.829835	2.109008	-1.417146
C	-5.873426	2.320391	-2.046892
H	-5.802010	1.602866	-2.878091
H	-6.895101	2.290678	-1.655346
H	-5.698465	3.323717	-2.436846
C	-2.828718	-1.216489	2.047821
C	-3.041780	-1.978151	3.197881
C	-1.520731	-0.840067	1.716551
C	-1.962817	-2.348708	3.992849
C	-0.441876	-1.218764	2.499803
H	-1.382034	-0.250468	0.825998
C	-0.667258	-1.975971	3.653077
H	0.146769	-2.281915	4.299711
N	0.963785	-0.829226	2.152708
C	1.832894	-2.043484	2.366427
H	1.967673	-2.204354	3.435362
H	1.360102	-2.916997	1.928316
H	2.805726	-1.855674	1.917447
H	-2.127245	-2.929377	4.895079
H	-5.069497	-2.639216	1.462507

H	-4.050278	-2.262063	3.483124
B	1.195237	-0.271196	0.533389
C	0.605050	-1.479704	-0.454998
C	-0.551568	-1.344111	-1.233489
C	1.208021	-2.733668	-0.623672
C	-1.090476	-2.339040	-2.039903
C	0.707643	-3.755329	-1.417920
C	-0.459294	-3.563731	-2.134918
C	2.809985	0.072422	0.291272
C	3.427719	1.151510	0.938930
C	3.625856	-0.483682	-0.702372
C	4.752425	1.529224	0.768167
C	4.957099	-0.138451	-0.906917
C	5.538580	0.863978	-0.154302
C	0.481085	1.185011	0.187358
C	0.748540	1.665362	-1.100194
C	-0.306987	2.048473	0.940725
C	0.305695	2.877809	-1.600238
C	-0.765101	3.280797	0.485120
C	-0.466866	3.701516	-0.795391
C	1.444771	0.131811	3.211320
H	1.164341	-0.270821	4.185277
H	2.529954	0.184884	3.155111
H	1.002707	1.109871	3.081336
F	2.736150	1.955794	1.764849
F	5.254377	2.552905	1.461424
F	6.808355	1.210407	-0.343032
F	5.664613	-0.745274	-1.862137
F	3.158592	-1.348823	-1.610194
F	2.376036	-3.038278	-0.033350
F	1.358942	-4.916851	-1.504511
F	-0.955594	-4.530873	-2.900347
F	-2.212114	-2.110713	-2.723937
F	-1.274612	-0.207846	-1.247043
F	1.438621	0.900262	-1.963414
F	-0.722995	1.737869	2.182968
F	-1.543348	4.031461	1.269371
F	-0.949659	4.851470	-1.260618
F	0.592318	3.243554	-2.850197

add$_{2'}$

E(SMD/M06-2X/6-31g(d)) = -4361.257466 au

E(SMD/M06-2X/def2-TZVP//SMD/M06-2X/6-31g(d)) = -4362.912850 au

H(SMD/M06-2X/6-31g(d)) = -4360.481167 au

G(SMD/M06-2X/6-31g(d)) = -4360.655784 au

C	-3.395597	2.776867	0.701817
C	-4.056987	4.109490	0.600438
C	-4.698117	4.197175	-0.630417
C	-4.472338	2.926068	-1.375155
O	-4.868963	2.607996	-2.466688

O	-2.763610	2.315547	1.614843
N	-3.675466	2.123549	-0.524557
S	-3.092487	0.587028	-0.957899
C	-4.806932	6.293422	1.150532
C	-4.094313	5.147721	1.515291
C	-5.409249	5.325809	-1.001777
C	-5.453739	6.380412	-0.086016
H	-4.861458	7.130991	1.838786
H	-3.592245	5.070752	2.474556
H	-5.908740	5.386849	-1.963561
H	-6.000967	7.283464	-0.337187
C	-4.348485	-0.465470	-0.222557
C	-5.503237	0.043753	0.364290
C	-4.129331	-1.852270	-0.199174
C	-6.425368	-0.790209	0.984145
H	-5.701913	1.111044	0.351704
C	-5.052808	-2.682761	0.435935
C	-6.213692	-2.180317	1.054129
H	-7.312380	-0.342987	1.416324
H	-4.854241	-3.748390	0.422778
N	-7.107205	-3.018177	1.702605
C	-8.420804	-2.492522	2.027391
H	-9.007217	-3.278841	2.504411
H	-8.342371	-1.662806	2.736967
H	-8.966342	-2.137575	1.139914
C	-7.003814	-4.446529	1.463935
H	-7.097712	-4.705267	0.398262
H	-6.046649	-4.832896	1.827845
H	-7.795516	-4.955026	2.015588
B	2.349480	0.325556	-0.161141
O	2.824186	1.499644	0.752068
C	4.002651	2.468660	2.616568
C	4.812280	1.925168	3.613228
C	4.891016	0.456218	3.388468
H	3.970386	-0.714882	1.831126
N	4.082425	0.215933	2.231534
C	3.562091	1.359510	1.755713
O	5.473606	-0.402264	3.986625
C	5.391710	2.716747	4.584727
C	3.735213	3.823080	2.539922
C	5.126932	4.090881	4.522147
C	4.317268	4.632303	3.521834
H	6.023462	2.293023	5.358579
H	5.561383	4.750722	5.266079
H	4.135899	5.701827	3.505358
H	3.107404	4.236870	1.757553
C	3.642842	-0.443924	-0.798032
C	4.948259	0.027969	-0.856767
C	3.416438	-1.621887	-1.505021
C	5.970549	-0.639675	-1.524021

C	4.401921	-2.324017	-2.175076
C	5.697075	-1.824079	-2.185591
C	1.600321	1.030889	-1.418337
C	2.172561	2.130300	-2.050456
C	0.456904	0.527085	-2.029052
C	1.632131	2.730320	-3.178874
C	-0.107853	1.087326	-3.166928
C	0.479894	2.203098	-3.741187
C	1.379714	-0.577093	0.795085
C	0.109227	-0.108340	1.134920
C	1.713589	-1.803580	1.351127
C	-0.785940	-0.828123	1.910374
C	0.868443	-2.544246	2.165693
C	-0.392889	-2.050637	2.438059
F	2.172340	-2.128343	-1.547278
F	5.300908	1.173431	-0.245088
F	4.118232	-3.463148	-2.809076
F	6.662962	-2.476731	-2.827930
F	7.207550	-0.140605	-1.533623
F	3.321614	2.647137	-1.584102
F	2.217028	3.795075	-3.734341
F	-0.048960	2.757709	-4.831078
F	-1.206801	0.558961	-3.710161
F	-0.161057	-0.549695	-1.533364
F	1.260380	-3.715988	2.667928
F	2.940335	-2.339435	1.149477
F	-0.295571	1.083661	0.681137
F	-2.015435	-0.384279	2.154065
F	-1.246846	-2.749949	3.188754
C	-2.931760	-2.464042	-0.845011
C	-2.670379	-2.255149	-2.204266
C	-2.074274	-3.260511	-0.086087
C	-1.542664	-2.838778	-2.771553
H	-3.349283	-1.660277	-2.808547
C	-0.921717	-3.844962	-0.647851
H	-2.300678	-3.395378	0.967029
C	-0.674888	-3.620021	-2.015994
H	-1.332475	-2.686325	-3.826788
H	0.189561	-4.062167	-2.497158
N	-0.058998	-4.599148	0.145102
C	1.119748	-5.171595	-0.477540
H	1.760055	-5.582542	0.306432
H	0.885866	-5.975514	-1.194353
H	1.686846	-4.395582	-0.997557
C	-0.655886	-5.411340	1.196869
H	-1.411303	-6.108413	0.804221
H	0.132364	-5.989644	1.681428
H	-1.120821	-4.793095	1.967481

add$_{3'}$

E(SMD/M06-2X/6-31g(d)) = -3848.332660 au

E(SMD/M06-2X/def2-TZVP//SMD/M06-2X/6-31g(d)) = -3849.795831 au
H(SMD/M06-2X/6-31g(d)) = -3847.684947 au
G(SMD/M06-2X/6-31g(d)) = -3847.838714 au

C	0.044421	-0.801448	0.590001
C	-0.143660	-1.307512	1.970368
C	1.086768	-1.826745	2.383300
C	2.067999	-1.654910	1.283629
O	3.226329	-1.945499	1.215057
O	-0.747088	-0.253667	-0.207734
N	1.334494	-1.012199	0.227225
S	2.041992	-0.572854	-1.291039
C	-1.052652	-1.851836	4.101870
C	-1.233114	-1.299710	2.828603
C	1.275660	-2.370293	3.637523
C	0.173658	-2.381895	4.499872
H	-1.889385	-1.861603	4.792503
H	-2.191459	-0.878402	2.554190
H	2.240186	-2.766119	3.938161
H	0.276654	-2.802591	5.494722
B	-2.303475	-0.086641	-0.185575
C	-2.741545	1.023323	0.919392
C	-4.091367	1.350181	1.010754
C	-1.892885	1.807869	1.688979
C	-4.587313	2.346585	1.834384
C	-2.345178	2.819869	2.527387
C	-3.700876	3.088745	2.602710
C	-2.613022	0.571345	-1.646022
C	-1.916938	1.717151	-2.021018
C	-3.578435	0.149796	-2.552392
C	-2.120058	2.388093	-3.216487
C	-3.818373	0.793997	-3.760202
C	-3.082813	1.917928	-4.096395
C	-2.896250	-1.594760	-0.013043
C	-2.470971	-2.563785	-0.920826
C	-3.805749	-2.049491	0.933607
C	-2.900974	-3.880185	-0.906626
C	-4.262364	-3.361292	0.989011
C	-3.811009	-4.282378	0.059793
F	-1.604908	-2.225329	-1.889175
F	-2.460226	-4.754718	-1.811675
F	-4.272683	-1.237657	1.898386
F	-5.126534	-3.735809	1.933447
F	-4.239166	-5.541456	0.098149
F	-0.559783	1.622814	1.661729
F	-1.482169	3.535557	3.250967
F	-4.151665	4.053826	3.401439
F	-4.975676	0.675339	0.258988
F	-5.894261	2.606545	1.890943
F	-1.004532	2.243970	-1.184543
F	-1.419093	3.483104	-3.516769

F	-3.304498	2.546997	-5.249003
F	-4.757892	0.342358	-4.593828
F	-4.349496	-0.917734	-2.299522
C	2.950948	0.859529	-0.768047
C	4.356073	0.869370	-0.664239
C	2.225603	1.987380	-0.363484
C	4.981540	1.995000	-0.130346
C	2.848681	3.096006	0.174982
H	1.144206	1.982326	-0.462191
C	4.256846	3.120188	0.322736
H	6.064315	1.997227	-0.089440
H	2.245755	3.944752	0.473457
N	4.893169	4.197040	0.871190
C	6.343354	4.237019	0.909704
H	6.658635	5.152373	1.410049
H	6.744998	3.386954	1.472764
H	6.782536	4.221366	-0.096919
C	4.135887	5.390939	1.200805
H	3.667851	5.838522	0.313910
H	3.348956	5.169551	1.930252
H	4.809125	6.125206	1.643074
C	5.212824	-0.252191	-1.137838
C	6.196600	-0.760534	-0.288870
C	5.081032	-0.753861	-2.436495
C	7.061494	-1.794040	-0.701202
H	6.269976	-0.353447	0.712953
C	5.939776	-1.764072	-2.858938
H	4.344143	-0.334677	-3.115823
C	6.918247	-2.280191	-2.018172
H	5.857185	-2.152755	-3.870094
H	7.571932	-3.060258	-2.389781
N	8.010585	-2.315957	0.159511
C	8.299221	-1.599850	1.387925
H	7.419840	-1.572691	2.039773
H	9.092417	-2.122594	1.923655
H	8.624013	-0.563985	1.206463
C	9.055601	-3.157394	-0.393124
H	9.641857	-2.646029	-1.171312
H	9.730112	-3.455776	0.410214
H	8.633142	-4.069222	-0.827376

ip1'

E(SMD/M06-2X/6-31g(d)) = -4361.233707 au

E(SMD/M06-2X/def2-TZVP//SMD/M06-2X/6-31g(d)) = -4362.894552 au

H(SMD/M06-2X/6-31g(d)) = -4360.456540 au

G(SMD/M06-2X/6-31g(d)) = -4360.629103 au

C	-3.352604	-2.004898	-0.023787
C	-4.701283	-2.609792	-0.203589
C	-5.369170	-1.918057	-1.209653
C	-4.482745	-0.837780	-1.721199

O	-4.690573	-0.016895	-2.577437
O	-2.483038	-2.270372	0.766280
N	-3.280030	-0.962254	-0.980742
S	-1.973337	0.100837	-1.099225
C	-6.609646	-3.982580	0.121957
C	-5.301904	-3.650974	0.484284
C	-6.665746	-2.237829	-1.577796
C	-7.279425	-3.289973	-0.892672
H	-7.117256	-4.793253	0.634878
H	-4.778079	-4.180833	1.273501
H	-7.181096	-1.693061	-2.363655
H	-8.294350	-3.576223	-1.151109
C	1.711725	-1.578096	1.412126
C	1.305913	-2.871528	2.033896
C	0.115874	-2.593760	2.691029
C	-0.146390	-1.129718	2.453402
O	-1.071204	-0.490209	2.914076
O	2.807678	-1.528889	0.743251
N	0.868798	-0.598244	1.635831
C	1.170233	-5.135596	2.718622
C	1.864359	-4.135598	2.024656
C	-0.579354	-3.570389	3.376581
C	-0.027525	-4.858632	3.381249
H	1.567470	-6.145788	2.739639
H	2.790746	-4.345981	1.499174
H	-1.513722	-3.351524	3.884637
H	-0.540225	-5.659480	3.905317
B	3.315199	-0.306909	0.006084
C	4.752786	-0.795777	-0.618480
C	5.953868	-0.098741	-0.578084
C	4.799173	-1.998320	-1.320091
C	7.128201	-0.565375	-1.156972
C	5.949057	-2.502676	-1.909578
C	7.127406	-1.777733	-1.824908
C	2.374210	0.031806	-1.287600
C	2.720295	1.113543	-2.093217
C	1.334136	-0.748460	-1.775666
C	2.065684	1.450308	-3.264938
C	0.651690	-0.450129	-2.951763
C	1.009243	0.659299	-3.695480
C	3.463206	0.902174	1.102329
C	2.791822	2.116403	1.108007
C	4.288432	0.700946	2.205086
C	2.906879	3.059601	2.121273
C	4.442096	1.612993	3.237853
C	3.740038	2.807852	3.196814
F	1.947430	2.453951	0.113993
F	2.215171	4.203860	2.062253
F	5.008127	-0.430058	2.298152
F	5.258410	1.356230	4.263833

F	3.867068	3.700392	4.178953
F	3.683922	-2.730970	-1.473873
F	5.930895	-3.668590	-2.562241
F	8.244617	-2.240001	-2.387298
F	6.045503	1.091479	0.036894
F	8.256121	0.147004	-1.077178
F	3.750793	1.895705	-1.724197
F	2.438108	2.513354	-3.984774
F	0.358111	0.959679	-4.819924
F	-0.364267	-1.220356	-3.356600
F	0.922811	-1.856791	-1.141596
C	-2.154641	1.070653	0.489525
C	-3.450072	1.801707	0.534636
C	-0.945613	1.927828	0.487941
C	-3.500687	3.132126	0.283857
C	-1.003274	3.252221	0.279858
H	0.004814	1.418370	0.628476
C	-2.291763	3.900629	0.075432
H	-4.452772	3.646952	0.332627
H	-0.093278	3.838034	0.268644
H	-2.070658	0.325089	1.286378
N	-2.350037	5.175918	-0.239285
C	-3.628327	5.843154	-0.511015
H	-3.424872	6.764828	-1.052759
H	-4.262668	5.208029	-1.129520
H	-4.138989	6.079537	0.427313
C	-1.136203	6.002209	-0.305015
H	-0.586835	5.943101	0.636318
H	-0.498044	5.671894	-1.128508
H	-1.432670	7.034957	-0.472896
C	-4.660384	1.028131	0.887695
C	-5.841966	1.215293	0.167480
C	-4.610619	0.118000	1.953193
C	-7.006921	0.482373	0.477625
H	-5.830624	1.895168	-0.676907
C	-5.774906	-0.563906	2.297774
H	-3.695952	-0.037841	2.517847
C	-6.953192	-0.397738	1.582596
H	-5.760447	-1.258792	3.132819
H	-7.825305	-0.977242	1.862576
N	-8.139459	0.607103	-0.287399
C	-9.316364	-0.173870	0.036215
H	-9.642277	0.018452	1.064499
H	-10.128233	0.117828	-0.630795
H	-9.145094	-1.254803	-0.072823
C	-8.056914	1.279335	-1.571122
H	-7.923915	2.362697	-1.454922
H	-7.220143	0.896657	-2.171101
H	-8.983431	1.109706	-2.120425

$ip_{1'\text{-}ortho}$

E(SMD/M06-2X/6-31g(d)) = -4361.236976 au
E(SMD/M06-2X/def2-TZVP//SMD/M06-2X/6-31g(d)) = -4362.891375 au
H(SMD/M06-2X/6-31g(d)) = -4360.459891 au
G(SMD/M06-2X/6-31g(d)) = -4360.625045 au

C	-5.841339	0.140370	-0.909182
C	-5.673350	1.619609	-0.890248
C	-4.457949	1.947182	-1.487293
C	-3.782419	0.691740	-1.904510
O	-2.694144	0.516662	-2.392549
O	-6.742349	-0.540838	-0.499447
N	-4.671034	-0.352107	-1.553027
S	-4.240435	-1.976142	-1.677272
C	-6.056400	3.914473	-0.419788
C	-6.497742	2.589620	-0.347311
C	-4.012779	3.255465	-1.570645
C	-4.837867	4.241910	-1.022804
H	-6.669768	4.705290	0.000492
H	-7.438053	2.328315	0.128105
H	-3.062284	3.495682	-2.037822
H	-4.528289	5.281565	-1.064220
C	0.792721	-0.467674	1.393134
C	0.023475	-0.013105	2.592275
C	-0.776216	-1.093755	2.939422
C	-0.465652	-2.164749	1.925326
O	-0.931526	-3.286562	1.902341
O	1.663903	0.329526	0.894564
N	0.458388	-1.672707	0.990523
C	-0.962165	1.286039	4.311281
C	-0.033879	1.192959	3.264962
C	-1.685875	-1.018683	3.975999
C	-1.774613	0.203628	4.656861
H	-1.057432	2.218266	4.860283
H	0.597227	2.031569	2.984003
H	-2.318594	-1.861429	4.239545
H	-2.491561	0.315189	5.464611
B	2.752875	0.140768	-0.171761
C	2.906199	-1.436472	-0.551077
C	3.269491	-2.340591	0.443913
C	2.546151	-2.019517	-1.756222
C	3.272140	-3.713713	0.285487
C	2.538331	-3.395709	-1.964503
C	2.886487	-4.249515	-0.935993
C	2.324412	1.103253	-1.423956
C	3.221111	1.900382	-2.125102
C	0.996851	1.224894	-1.823909
C	2.836266	2.805012	-3.107455
C	0.564183	2.133164	-2.779356
C	1.496551	2.933118	-3.424324
C	4.067847	0.789788	0.572170

C	5.339222	0.233149	0.636610
C	3.951191	2.034526	1.187110
C	6.410823	0.835523	1.283563
C	4.993036	2.671254	1.847018
C	6.236954	2.064595	1.896952
F	5.611237	-0.943734	0.047811
F	7.607931	0.242700	1.310889
F	2.788933	2.711062	1.144965
F	4.810701	3.863972	2.422012
F	7.255176	2.658531	2.520028
F	2.112837	-1.284255	-2.794950
F	2.128705	-3.901266	-3.136513
F	2.836442	-5.569882	-1.111975
F	3.585913	-1.879824	1.665378
F	3.607352	-4.525685	1.291863
F	4.542200	1.830105	-1.889591
F	3.745262	3.551724	-3.739740
F	1.101149	3.804553	-4.352686
F	-0.725613	2.255777	-3.098241
F	0.063688	0.416054	-1.284237
C	-2.953507	-2.044871	-0.300292
C	-3.635600	-1.774938	1.010474
C	-2.391050	-3.423581	-0.469009
C	-4.100752	-2.832195	1.718273
C	-3.058641	-4.490075	0.222661
C	-3.882717	-4.178255	1.257872
H	-4.622074	-2.687003	2.658069
H	-2.854455	-5.522057	-0.032578
H	-2.211331	-1.284883	-0.545638
C	-3.824386	-0.372421	1.441786
C	-4.932609	-0.042658	2.237683
C	-2.889009	0.615993	1.109251
C	-5.060627	1.255059	2.717683
H	-5.695493	-0.783971	2.448865
C	-3.031602	1.945776	1.566853
C	-4.131952	2.239052	2.404037
H	-4.274772	3.241606	2.789192
N	-1.392656	-3.620142	-1.289369
C	-0.783412	-4.953342	-1.388297
H	-1.496265	-5.664578	-1.812005
H	0.086882	-4.892231	-2.038215
H	-0.475354	-5.279642	-0.391547
C	-0.727682	-2.516971	-2.001036
H	-0.063856	-2.004691	-1.296349
H	-0.154376	-2.944033	-2.822237
H	-1.460142	-1.820229	-2.412609
H	-4.360146	-4.986892	1.803510
H	-5.915070	1.516127	3.335717
N	-2.134225	2.919383	1.213023
C	-1.119100	2.656152	0.211931

H	-1.542653	2.124610	-0.647930
H	-0.716606	3.608107	-0.140716
H	-0.286140	2.055749	0.604580
C	-2.268861	4.251936	1.763039
H	-1.428284	4.860209	1.428295
H	-3.200816	4.736948	1.438277
H	-2.257567	4.234368	2.860124
H	-1.999862	0.355687	0.543027

ip2'

E(SMD/M06-2X/6-31g(d)) = -3848.335307 au

E(SMD/M06-2X/def2-TZVP//SMD/M06-2X/6-31g(d)) = -3849.795016 au

H(SMD/M06-2X/6-31g(d)) = -3847.687417 au

G(SMD/M06-2X/6-31g(d)) = -3847.835751 au

S	-2.653529	0.030167	-2.689146
C	1.010876	1.773337	0.801709
C	2.385351	2.132841	1.306928
C	2.250164	3.444386	1.755563
C	0.815333	3.825781	1.522853
O	0.315215	4.897262	1.804408
O	0.635112	0.660244	0.274412
N	0.144193	2.750694	0.933407
C	4.674488	2.186192	1.971746
C	3.601410	1.477420	1.412877
C	3.295849	4.152966	2.310759
C	4.530629	3.500335	2.411568
H	5.637657	1.694122	2.065612
H	3.746000	0.452212	1.099251
H	3.161328	5.173375	2.657199
H	5.382521	4.017611	2.842152
B	1.340376	-0.642995	-0.044488
C	1.937560	-1.397742	1.277228
C	2.565292	-2.627780	1.102378
C	1.739848	-1.026673	2.600532
C	3.025236	-3.416272	2.143165
C	2.182734	-1.786658	3.678170
C	2.831771	-2.987314	3.448588
C	0.109700	-1.617288	-0.533787
C	-1.017223	-1.715939	0.281544
C	0.098510	-2.447970	-1.647351
C	-2.085879	-2.559378	0.020175
C	-0.957589	-3.298581	-1.953526
C	-2.059016	-3.352007	-1.116518
C	2.414473	-0.322482	-1.243987
C	1.920612	0.269410	-2.404377
C	3.786098	-0.543744	-1.261391
C	2.699906	0.643917	-3.485739
C	4.612365	-0.183816	-2.320742
C	4.066832	0.414805	-3.442461
F	0.597154	0.484725	-2.529632

F	2.149556	1.210491	-4.562650
F	4.418055	-1.105760	-0.214071
F	5.926740	-0.409049	-2.259135
F	4.843402	0.770765	-4.464234
F	1.101903	0.113678	2.914399
F	1.977291	-1.372167	4.931216
F	3.261099	-3.728445	4.469656
F	2.738496	-3.102278	-0.143151
F	3.633560	-4.581403	1.907291
F	-1.095781	-0.996408	1.413635
F	-3.133035	-2.610774	0.846180
F	-3.086371	-4.154371	-1.401406
F	-0.916808	-4.066925	-3.046064
F	1.132574	-2.473353	-2.504144
C	-3.900445	-0.365770	-1.498064
C	-3.994742	0.536794	-0.430367
C	-4.759137	-1.460815	-1.549921
C	-4.938247	0.357988	0.583169
C	-5.700248	-1.637120	-0.546882
H	-4.694379	-2.182527	-2.359070
C	-5.820978	-0.734257	0.539975
H	-4.958782	1.062492	1.406717
H	-6.348146	-2.503765	-0.602366
N	-6.780493	-0.927996	1.516868
C	-6.707888	-0.118395	2.718945
H	-7.535664	-0.388536	3.375542
H	-5.763457	-0.258134	3.266592
H	-6.810385	0.944056	2.475550
C	-7.395071	-2.239726	1.633259
H	-8.076586	-2.232496	2.484618
H	-7.983449	-2.478749	0.741878
H	-6.653691	-3.038110	1.784152
C	-3.067034	1.652520	-0.577977
C	-2.003035	1.346193	-1.582390
C	-3.118009	2.859620	0.028892
C	-1.429266	2.544854	-2.247415
H	-1.171956	0.884103	-1.015414
C	-2.254334	3.925346	-0.426401
H	-3.867392	3.065046	0.784442
C	-1.537905	3.757699	-1.689998
H	-1.056943	4.611870	-2.148818
N	-2.157080	5.047925	0.243249
C	-2.732588	5.176000	1.584054
H	-2.216279	5.981053	2.101585
H	-3.801255	5.403763	1.521407
H	-2.576711	4.249583	2.137931
C	-1.421687	6.199367	-0.289851
H	-1.848291	6.507095	-1.247033
H	-1.512364	7.019932	0.418176
H	-0.365378	5.950864	-0.408580

H	-0.839589	2.406232	-3.148568

TS$_{2'\text{-ortho}}$

E(SMD/M06-2X/6-31g(d)) = -4361.206113 au

E(SMD/M06-2X/def2-TZVP//SMD/M06-2X/6-31g(d)) = -4362.86255998 au

H(SMD/M06-2X/6-31g(d)) = -4360.431755 au

G(SMD/M06-2X/6-31g(d)) = -4360.59961 au

C	1.44713300	-3.15558500	0.71295700
C	0.88202100	-3.79293000	-0.50671000
C	0.75592300	-2.82101400	-1.49451200
C	1.18176900	-1.50724700	-0.93947100
O	1.14600800	-0.42182000	-1.45315200
O	1.70940300	-3.64814500	1.78073400
N	1.65481800	-1.79502800	0.37198300
S	2.33350000	-0.67673500	1.42108200
C	0.08539900	-5.43774300	-2.02225100
C	0.55047900	-5.11634900	-0.74309100
C	0.31865700	-3.13185000	-2.76944500
C	-0.02521100	-4.46415100	-3.01960300
H	-0.18979400	-6.46300400	-2.24860900
H	0.65086700	-5.87032600	0.03114300
H	0.24028000	-2.36884400	-3.53801200
H	-0.39090100	-4.74546900	-4.00279300
C	-0.88387300	-0.29454800	1.92016600
C	-1.88140400	-0.30295300	3.05488400
C	-1.10551800	-0.38207700	4.21271800
C	0.32542000	-0.47645100	3.78396700
O	1.30183800	-0.55878800	4.49365000
O	-1.07875000	-0.22827800	0.66545000
N	0.34593900	-0.44483800	2.38403600
C	-3.83457600	-0.34617500	4.42699200
C	-3.26641500	-0.30984700	3.14646300
C	-1.65217700	-0.42120200	5.47900100
C	-3.04767300	-0.38993100	5.57578700
H	-4.91609200	-0.34506300	4.52003600
H	-3.90727100	-0.29830600	2.27550800
H	-1.01964300	-0.48159200	6.35923200
H	-3.52261600	-0.41576000	6.55156700
B	-2.01411700	0.44627900	-0.34477500
C	-1.17560000	1.63345000	-1.09218000
C	-0.00543500	2.21899300	-0.62416500
C	-1.73574900	2.25123200	-2.20524700
C	0.58796500	3.31647200	-1.23798500
C	-1.16407300	3.32704200	-2.86467400
C	0.01683100	3.86386500	-2.37309600
C	-2.45206500	-0.80532700	-1.30245700
C	-3.20039600	-1.83148000	-0.73165800
C	-2.09146300	-1.03030600	-2.62513400
C	-3.58693900	-2.98567500	-1.39156800
C	-2.47330100	-2.16492400	-3.33435700

C	-3.20876600	-3.15671600	-2.71411500
C	-3.26269500	1.22768200	0.37181900
C	-2.98285500	2.16411400	1.36367600
C	-4.60643900	1.12392100	0.03249500
C	-3.94613200	2.91040000	2.02213000
C	-5.60785300	1.85368700	0.66426900
C	-5.27690000	2.75026100	1.66644800
F	-1.70890600	2.34809000	1.75755500
F	-3.61081600	3.77280600	2.98299700
F	-5.01813900	0.29249200	-0.93610200
F	-6.88469300	1.70501200	0.30475500
F	-6.22555700	3.45755500	2.27743200
F	-2.90715200	1.79458000	-2.68796300
F	-1.74090100	3.85983100	-3.94749100
F	0.58733500	4.90848100	-2.97816600
F	0.60923000	1.77771000	0.47815100
F	1.69424300	3.86152100	-0.72456100
F	-3.60266300	-1.71888200	0.54743500
F	-4.30365100	-3.92606600	-0.77105300
F	-3.52026400	-4.27377600	-3.37014100
F	-2.04076000	-2.35291500	-4.58654600
F	-1.30165700	-0.18335100	-3.30018100
C	3.67780200	1.32176200	-2.07136900
C	4.42568300	0.83412900	-0.99268900
C	3.81274400	2.65779400	-2.43066100
C	5.28151400	1.68807800	-0.29051200
C	4.66816300	3.51130300	-1.74478400
C	5.41423400	3.04681800	-0.64174600
H	5.88391300	1.28071000	0.51272600
H	4.74192800	4.54475200	-2.06149600
H	2.98220900	0.67254600	-2.59131400
C	4.35079800	-0.59816300	-0.61977900
C	4.23622200	-1.58451100	-1.56445400
C	4.34740000	-0.96827700	0.78535500
C	4.25808600	-2.94671200	-1.16250800
H	4.18076600	-1.33714700	-2.61922100
C	4.56860000	-2.35752300	1.16405300
H	4.73808600	-0.21515800	1.46566800
C	4.43868700	-3.34202500	0.13876000
H	4.49162100	-4.39649300	0.37769900
N	4.79488200	-2.69950000	2.43296400
C	4.78568000	-4.10866600	2.82047400
H	5.59662900	-4.65062200	2.32506900
H	4.92871300	-4.17470800	3.89755400
H	3.82645200	-4.56681500	2.56073100
C	4.91629900	-1.69734500	3.48840700
H	3.93585000	-1.28623600	3.75533600
H	5.35310200	-2.17076900	4.36654900
H	5.57912000	-0.88641800	3.17612900
N	6.24392400	3.89298600	0.07215400

C	6.58823500	5.17723900	-0.51088300
H	7.27177500	5.69826600	0.16065300
H	7.07225500	5.07683200	-1.49380100
H	5.69856200	5.80384200	-0.62854800
C	7.17305500	3.31658800	1.02516200
H	7.88874700	2.62383300	0.55673300
H	7.73224300	4.12099500	1.50440700
H	6.63623100	2.77540800	1.81085900
H	3.22750000	3.05576800	-3.25607100
H	4.16054200	-3.71348700	-1.92696800

TS$_{2'}$

E(SMD/M06-2X/6-31g(d)) = -4361.216779 au

E(SMD/M06-2X/def2-TZVP//SMD/M06-2X/6-31g(d)) = -4362.872075 au

H(SMD/M06-2X/6-31g(d)) = -4360.442443 au

G(SMD/M06-2X/6-31g(d)) = -4360.607426 au

C	1.841722	-2.840185	1.521879
C	1.866093	-3.461121	0.171503
C	1.932217	-2.448657	-0.781081
C	1.852754	-1.127875	-0.089912
O	1.765461	-0.029694	-0.571601
O	1.789191	-3.365592	2.602470
N	1.895610	-1.435810	1.300811
S	1.905629	-0.340191	2.568765
C	1.991256	-5.099446	-1.541762
C	1.881562	-4.800356	-0.180710
C	2.072795	-2.735642	-2.129339
C	2.102310	-4.085073	-2.497836
H	2.004350	-6.136123	-1.862856
H	1.817844	-5.581074	0.570922
H	2.163723	-1.946979	-2.869769
H	2.200708	-4.349035	-3.546515
C	-1.210222	-0.809423	1.899605
C	-2.447157	-1.449795	2.485133
C	-2.136793	-1.661036	3.829464
C	-0.730465	-1.195602	4.044628
O	-0.098723	-1.212779	5.073486
O	-0.967422	-0.448582	0.704244
N	-0.259065	-0.714028	2.812843
C	-4.567395	-2.477962	2.871220
C	-3.666677	-1.880527	1.979389
C	-3.013087	-2.251559	4.716836
C	-4.256371	-2.654190	4.217762
H	-5.528329	-2.816394	2.496440
H	-3.934047	-1.781920	0.935565
H	-2.738838	-2.402435	5.756460
H	-4.979215	-3.120167	4.880077
B	-1.673280	0.192456	-0.488125
C	-1.145344	1.732458	-0.639621
C	-0.464032	2.474914	0.318986

C	-1.552819	2.450248	-1.759032
C	-0.185968	3.830250	0.165577
C	-1.279799	3.792323	-1.961556
C	-0.589576	4.489129	-0.981922
C	-1.269521	-0.792474	-1.728417
C	-1.726506	-2.106767	-1.697240
C	-0.461546	-0.490135	-2.818360
C	-1.456532	-3.055680	-2.669191
C	-0.178682	-1.404832	-3.826954
C	-0.669699	-2.695436	-3.751394
C	-3.284419	0.352458	-0.241086
C	-3.715152	0.995541	0.915683
C	-4.299047	-0.021767	-1.113571
C	-5.045883	1.204108	1.238608
C	-5.647767	0.170763	-0.834557
C	-6.023726	0.783644	0.349573
F	-2.804407	1.408392	1.817764
F	-5.392675	1.797612	2.381629
F	-4.024578	-0.609879	-2.287142
F	-6.583806	-0.218722	-1.702638
F	-7.312230	0.969981	0.629497
F	-2.265256	1.823560	-2.715218
F	-1.673935	4.418571	-3.075598
F	-0.307507	5.782445	-1.152929
F	-0.079197	1.931570	1.479982
F	0.465612	4.504551	1.116406
F	-2.511141	-2.506795	-0.679834
F	-1.923673	-4.302513	-2.566764
F	-0.339684	-3.595876	-4.676992
F	0.657567	-1.075811	-4.818457
F	0.116954	0.710137	-2.963246
C	3.432676	2.609498	0.178732
C	4.064178	2.076481	1.305901
C	3.200588	3.992542	0.058870
C	4.474917	2.911963	2.349465
C	3.650209	4.824754	1.108041
C	4.266756	4.283258	2.227780
H	4.984079	2.506453	3.218181
H	3.506833	5.897502	1.056387
H	3.092439	1.925185	-0.588366
C	4.321564	0.618825	1.374156
C	4.748878	-0.070349	0.267700
C	4.064749	-0.093831	2.603195
C	4.987691	-1.478587	0.318198
H	4.901389	0.468521	-0.658968
C	4.539908	-1.444983	2.698565
H	4.036095	0.489945	3.521876
C	4.948766	-2.134882	1.597704
H	5.244223	-3.172911	1.686179
N	2.543601	4.514498	-1.038900

C	2.626664	5.944202	-1.276464
H	3.666561	6.291816	-1.366156
H	2.098284	6.180966	-2.200117
H	2.144498	6.501463	-0.467537
C	2.282731	3.647156	-2.172005
H	1.737024	4.212007	-2.930365
H	3.205585	3.253846	-2.625747
H	1.656621	2.796411	-1.875824
H	4.599460	4.950089	3.018241
N	5.237121	-2.166756	-0.801735
C	5.247966	-1.493698	-2.096892
H	6.113164	-0.828164	-2.187998
H	5.296978	-2.245749	-2.883052
H	4.333489	-0.906956	-2.229840
C	5.477831	-3.605554	-0.754493
H	4.643134	-4.119018	-0.266252
H	5.570500	-3.979953	-1.772656
H	6.401950	-3.832967	-0.213088
H	4.494906	-1.943301	3.662520

$TS_{3'}$

E(SMD/M06-2X/6-31g(d)) = -4361.213149 au

E(SMD/M06-2X/def2-TZVP//SMD/M06-2X/6-31g(d)) = -4362.873315 au

H(SMD/M06-2X/6-31g(d)) = -4360.442514 au

G(SMD/M06-2X/6-31g(d)) = -4360.615633 au

C	-4.907819	1.491390	-0.750201
C	-5.882534	2.585558	-0.477400
C	-5.212523	3.624998	0.160631
C	-3.784089	3.234772	0.333239
O	-2.893407	3.839948	0.874664
O	-5.094540	0.412120	-1.252240
N	-3.665876	1.961739	-0.268368
S	-2.207682	1.075314	-0.370304
C	-7.902477	3.827872	-0.386765
C	-7.234797	2.658838	-0.763109
C	-5.863789	4.785706	0.540727
C	-7.229169	4.872558	0.253401
H	-8.963020	3.928418	-0.595029
H	-7.750777	1.843932	-1.260671
H	-5.335076	5.592150	1.038722
H	-7.778651	5.766460	0.531014
C	1.149921	-0.981894	1.362675
C	2.112981	-1.979991	1.926137
C	1.391484	-2.664182	2.905349
C	0.001623	-2.100233	2.908885
O	-0.928914	-2.436278	3.604063
O	1.318865	-0.117304	0.444418
N	-0.038576	-1.088250	1.934655
C	3.991034	-3.372008	2.375859
C	3.420392	-2.332408	1.631056

C	1.943085	-3.691563	3.643669
C	3.272101	-4.036132	3.368602
H	5.014694	-3.667168	2.168483
H	3.996255	-1.850098	0.850793
H	1.365361	-4.214302	4.399571
H	3.746906	-4.838378	3.924588
B	2.578608	0.411617	-0.251973
C	3.134961	-0.650532	-1.358578
C	4.172358	-0.251810	-2.197189
C	2.556867	-1.868984	-1.683978
C	4.630230	-1.001987	-3.267060
C	2.973915	-2.650320	-2.755031
C	4.017821	-2.215083	-3.551944
C	1.997915	1.681622	-1.109152
C	0.869223	1.483964	-1.899261
C	2.530827	2.963284	-1.164270
C	0.257654	2.485050	-2.637290
C	1.962679	3.992041	-1.906400
C	0.812271	3.755197	-2.639900
C	3.620336	0.871187	0.918929
C	3.149606	1.738120	1.903641
C	4.956051	0.515490	1.053100
C	3.923348	2.214549	2.949252
C	5.769827	0.965665	2.085921
C	5.250878	1.824232	3.039388
F	1.878824	2.168622	1.853201
F	3.409980	3.044270	3.859924
F	5.537094	-0.341470	0.194383
F	7.042607	0.572024	2.169074
F	6.015769	2.264826	4.036484
F	1.532683	-2.368924	-0.967447
F	2.362120	-3.806420	-3.026262
F	4.434187	-2.952536	-4.580216
F	4.771129	0.930174	-1.976417
F	5.633383	-0.567978	-4.033106
F	0.307695	0.263998	-1.971877
F	-0.855989	2.239371	-3.332718
F	0.251118	4.734442	-3.348159
F	2.516969	5.207235	-1.915113
F	3.647543	3.280810	-0.490609
C	-2.421460	-0.050390	1.022081
C	-3.024810	-1.362373	0.785742
C	-2.748010	0.592877	2.280758
C	-3.632305	-2.023068	1.815721
C	-3.352280	-0.067344	3.297053
H	-2.412932	1.617351	2.424831
C	-3.772600	-1.432606	3.115388
H	-3.965471	-3.040382	1.652312
H	-3.502112	0.429430	4.246768
H	-1.239952	-0.446872	1.377037

N	-4.263541	-2.134028	4.131318
C	-4.607789	-3.545551	3.965697
H	-4.936924	-3.940618	4.924313
H	-5.417267	-3.666984	3.240475
H	-3.733633	-4.112400	3.630554
C	-4.361711	-1.542179	5.465050
H	-3.368421	-1.272335	5.837905
H	-4.995186	-0.651660	5.447927
H	-4.806264	-2.270414	6.140485
C	-2.801325	-2.065323	-0.499902
C	-1.528207	-2.069259	-1.077035
C	-3.854148	-2.755279	-1.101248
C	-1.328587	-2.786437	-2.251528
H	-0.704467	-1.533807	-0.617077
C	-3.665905	-3.466710	-2.302211
H	-4.833557	-2.700413	-0.641213
C	-2.372066	-3.468707	-2.865187
H	-2.176685	-3.998841	-3.789427
N	-4.716218	-4.145452	-2.902677
C	-6.066997	-3.804665	-2.489956
H	-6.773237	-4.386517	-3.083473
H	-6.230072	-4.060703	-1.438746
H	-6.290166	-2.735018	-2.623490
C	-4.547488	-4.575688	-4.280255
H	-3.744111	-5.313995	-4.361062
H	-5.468780	-5.054698	-4.614620
H	-4.319479	-3.739960	-4.958925
H	-0.338682	-2.820809	-2.697914

TS$_{3'\text{-ortho}}$

E(SMD/M06-2X/6-31g(d)) = -4361.207751 au

E(SMD/M06-2X/def2-TZVP//SMD/M06-2X/6-31g(d)) = -4362.863007 au

H(SMD/M06-2X/6-31g(d)) = -4360.437730 au

G(SMD/M06-2X/6-31g(d)) = -4360.605512 au

C	4.650546	-0.864598	1.163996
C	6.132289	-0.701670	1.221796
C	6.433888	0.656836	1.245134
C	5.157099	1.425306	1.210447
O	4.979082	2.619846	1.206210
O	3.992474	-1.870231	1.169898
N	4.136338	0.458214	1.155557
S	2.483261	0.833316	0.990641
C	8.446013	-1.223651	1.284837
C	7.121648	-1.668640	1.242005
C	7.742169	1.108455	1.288335
C	8.750664	0.140701	1.308142
H	9.253863	-1.948742	1.299295
H	6.875164	-2.725636	1.218946
H	7.972498	2.169010	1.306344
H	9.789944	0.452159	1.340998

C	-1.154460	1.220436	-1.327606
C	-2.235587	1.714898	-2.238646
C	-1.587634	2.536050	-3.163063
C	-0.127981	2.508065	-2.832699
O	0.777828	3.086819	-3.384234
O	-1.232906	0.504798	-0.279955
N	0.030181	1.664941	-1.720618
C	-4.298125	2.154772	-3.344252
C	-3.601657	1.499060	-2.320335
C	-2.262972	3.185241	-4.176529
C	-3.646571	2.987086	-4.252593
H	-5.369386	2.003734	-3.431290
H	-4.133270	0.839456	-1.647208
H	-1.738231	3.817864	-4.885784
H	-4.217861	3.479061	-5.033354
B	-2.379951	-0.222469	0.439634
C	-3.086704	-1.349590	-0.507402
C	-4.079622	-2.141981	0.062550
C	-2.669045	-1.758050	-1.765611
C	-4.644201	-3.240451	-0.562581
C	-3.192629	-2.863834	-2.425913
C	-4.189989	-3.608924	-1.822140
C	-1.586959	-1.089274	1.581104
C	-0.516172	-1.881836	1.172121
C	-1.922147	-1.201104	2.924057
C	0.202108	-2.709368	2.022537
C	-1.229275	-2.014473	3.812345
C	-0.161482	-2.771377	3.359554
C	-3.364083	0.927297	1.057287
C	-2.810514	1.864975	1.926276
C	-4.719539	1.108300	0.811518
C	-3.507960	2.927550	2.475287
C	-5.464593	2.155370	1.342154
C	-4.855025	3.073967	2.178656
F	-1.522880	1.745374	2.293215
F	-2.902506	3.795510	3.288451
F	-5.398002	0.277192	-0.000221
F	-6.760017	2.281472	1.048310
F	-5.551929	4.086018	2.689861
F	-1.703403	-1.094050	-2.427477
F	-2.718018	-3.218625	-3.622873
F	-4.705888	-4.672239	-2.436351
F	-4.513584	-1.851435	1.300315
F	-5.594889	-3.960591	0.036127
F	-0.156483	-1.889716	-0.121524
F	1.206577	-3.460692	1.571608
F	0.502708	-3.563049	4.201490
F	-1.590620	-2.083170	5.096858
F	-2.956726	-0.518129	3.439732
C	2.504267	1.748702	-0.592225

C	3.416040	1.190394	-1.606431
C	2.479103	3.219880	-0.437828
C	4.122475	2.025279	-2.422548
C	3.327038	4.021293	-1.264901
C	4.089896	3.431759	-2.226745
H	4.736117	1.613882	-3.216331
H	3.348669	5.096385	-1.148790
H	1.303026	1.574256	-1.118337
C	3.607183	-0.276391	-1.751652
C	2.545835	-1.176606	-1.685112
C	4.918464	-0.752886	-1.922176
C	2.763385	-2.564575	-1.792748
H	1.538484	-0.806855	-1.515217
C	5.142501	-2.119927	-2.001026
H	5.750927	-0.056274	-1.946827
C	4.084528	-3.020723	-1.938974
H	4.288630	-4.082359	-2.018834
N	1.612226	3.814613	0.376918
C	0.530862	3.150347	1.103914
H	0.750968	3.110311	2.173495
H	-0.380959	3.735521	0.949043
H	0.346929	2.144350	0.734758
C	1.636241	5.268527	0.557636
H	1.291925	5.777922	-0.347941
H	0.970501	5.518058	1.382830
H	2.642277	5.607303	0.812315
H	4.701085	4.060477	-2.868154
N	1.688445	-3.453031	-1.701592
C	0.523105	-3.207817	-2.536926
H	0.647897	-3.649678	-3.539385
H	-0.360959	-3.654238	-2.070332
H	0.345230	-2.141051	-2.650896
C	1.986638	-4.863286	-1.570725
H	1.062551	-5.386674	-1.308658
H	2.381448	-5.313093	-2.497442
H	2.708680	-5.025398	-0.766070
H	6.155879	-2.495373	-2.114293

$TS_{4'}$

E(SMD/M06-2X/6-31g(d)) = -4361.243201 au

E(SMD/M06-2X/def2-TZVP//SMD/M06-2X/6-31g(d)) = -4362.900155 au

H(SMD/M06-2X/6-31g(d)) = -4360.468722 au

G(SMD/M06-2X/6-31g(d)) = -4360.640771 au

C	-0.982935	-3.538921	-1.563887
C	0.228979	-4.200175	-1.003369
C	0.602645	-3.530783	0.158603
C	-0.316229	-2.373207	0.354455
O	-0.272902	-1.493147	1.179485
O	-1.603454	-3.810525	-2.557801
N	-1.273831	-2.469046	-0.679041

S	-2.390050	-1.245018	-1.057341
C	2.117209	-5.624204	-0.796942
C	0.974176	-5.250899	-1.510800
C	1.718212	-3.909441	0.887387
C	2.474743	-4.972741	0.387141
H	2.736339	-6.436626	-1.164163
H	0.687596	-5.753119	-2.429365
H	1.993307	-3.400466	1.802406
H	3.362288	-5.293438	0.923737
C	-3.766631	-1.682946	0.005874
C	-4.966538	-0.970776	-0.140999
C	-3.650835	-2.611089	1.036667
C	-6.012041	-1.197933	0.754900
C	-4.702236	-2.847404	1.911683
H	-2.727802	-3.164187	1.176678
C	-5.917921	-2.145926	1.792017
H	-6.922926	-0.627494	0.612602
H	-4.562933	-3.581252	2.696174
N	-6.979073	-2.391347	2.646409
C	-8.090852	-1.458699	2.654581
H	-8.830486	-1.799352	3.380505
H	-7.785302	-0.435093	2.919774
H	-8.578701	-1.427655	1.675390
C	-6.717318	-3.130466	3.868170
H	-6.379466	-4.146958	3.642863
H	-5.957964	-2.646617	4.500750
H	-7.644226	-3.210156	4.437490
C	-5.153062	0.036610	-1.227182
C	-5.487177	1.350767	-0.894784
C	-5.017293	-0.337697	-2.568579
C	-5.700594	2.326696	-1.890499
H	-5.558606	1.610449	0.155053
C	-5.207242	0.624098	-3.556557
H	-4.775843	-1.363440	-2.829165
C	-5.535122	1.935226	-3.235451
H	-5.100486	0.349994	-4.602562
H	-5.664454	2.654642	-4.035001
N	-6.054777	3.623931	-1.561278
C	-5.891301	4.654402	-2.571341
H	-6.561004	4.481038	-3.419649
H	-6.155184	5.618981	-2.135376
H	-4.860309	4.711057	-2.950912
C	-5.939544	4.036975	-0.174591
H	-6.597809	3.437967	0.462372
H	-4.913133	3.945794	0.206908
H	-6.256176	5.077493	-0.089798
C	6.315497	2.647303	0.244475
C	6.191408	1.931802	-1.059965
C	4.835600	1.771714	-1.340928
C	4.067870	2.341392	-0.199613

O	2.855302	2.357005	-0.013446
O	7.302988	2.983044	0.846022
N	4.992165	2.866943	0.665802
C	6.746771	0.826127	-3.084077
C	7.172701	1.466431	-1.914626
C	4.403374	1.157467	-2.504361
C	5.388790	0.680025	-3.375400
H	7.483695	0.439334	-3.780642
H	8.226410	1.590633	-1.685920
H	3.348077	1.049304	-2.731840
H	5.091796	0.184837	-4.294643
H	4.747976	3.305037	1.548979
B	1.555721	0.779316	0.447932
C	0.238859	1.459806	-0.118484
C	-0.906153	1.646423	0.652317
C	0.169644	1.963371	-1.415556
C	-2.036236	2.302231	0.182923
C	-0.936778	2.627444	-1.918667
C	-2.045703	2.810968	-1.104502
C	1.810886	0.902070	2.015853
C	2.215379	-0.149169	2.832047
C	1.593629	2.110657	2.681476
C	2.407331	-0.026496	4.201237
C	1.779491	2.275830	4.044439
C	2.188712	1.195990	4.812499
C	2.290754	-0.355425	-0.392935
C	3.581191	-0.785376	-0.077420
C	1.723248	-1.001550	-1.490864
C	4.244332	-1.802159	-0.744314
C	2.351719	-2.025374	-2.189260
C	3.614403	-2.441451	-1.803088
F	4.270442	-0.172243	0.896658
F	5.476693	-2.159863	-0.387808
F	4.219819	-3.433958	-2.443806
F	1.726931	-2.630588	-3.199042
F	0.491188	-0.694243	-1.916959
F	-0.972279	1.205503	1.911340
F	1.209772	1.821993	-2.248606
F	-0.936485	3.114042	-3.160664
F	-3.099133	3.485435	-1.556715
F	-3.095526	2.465488	0.978665
F	1.165908	3.185487	2.011328
F	1.555942	3.456477	4.623621
F	2.369260	1.334743	6.121759
F	2.804350	-1.073313	4.926130
F	2.453538	-1.362795	2.320722

$TS_{5'}$

E(SMD/M06-2X/6-31g(d)) = -3848.309856 au

E(SMD/M06-2X/def2-TZVP//SMD/M06-2X/6-31g(d)) = -3849.767623 au

H(SMD/M06-2X/6-31g(d)) = -3847.663671 au

G(SMD/M06-2X/6-31g(d)) = -3847.811988 au

S	1.910770	-1.143159	-2.893767
C	-0.938364	-1.103092	-1.756862
C	-2.180362	-1.926423	-1.900560
C	-2.164661	-2.384662	-3.217200
C	-0.899375	-1.888363	-3.846673
O	-0.495992	-2.109251	-4.961895
O	-0.514688	-0.454322	-0.752422
N	-0.225534	-1.114374	-2.879422
C	-4.183710	-3.158877	-1.529472
C	-3.178339	-2.325664	-1.024015
C	-3.149781	-3.206001	-3.727126
C	-4.179573	-3.583554	-2.857878
H	-4.979341	-3.485499	-0.867381
H	-3.193151	-2.033923	0.019664
H	-3.115364	-3.552224	-4.755388
H	-4.975743	-4.229291	-3.214694
B	-1.204089	0.037996	0.522402
C	-1.233802	-1.140873	1.651151
C	-1.637577	-0.809491	2.940907
C	-0.790347	-2.446487	1.492453
C	-1.624305	-1.694304	4.005059
C	-0.758535	-3.369833	2.532404
C	-1.174782	-2.990938	3.796486
C	-0.158758	1.184182	1.064171
C	1.168283	0.828362	1.297845
C	-0.462898	2.510615	1.356133
C	2.121559	1.703091	1.801828
C	0.467017	3.420801	1.839900
C	1.767999	3.013919	2.071126
C	-2.671952	0.644391	0.112305
C	-2.740430	1.587537	-0.911033
C	-3.902898	0.326261	0.673506
C	-3.914038	2.164702	-1.365722
C	-5.107811	0.878916	0.253304
C	-5.115103	1.806623	-0.772585
F	-1.607054	2.003127	-1.499232
F	-3.897888	3.067055	-2.348950
F	-4.012623	-0.599050	1.643249
F	-6.255698	0.514886	0.829064
F	-6.258932	2.348213	-1.185268
F	-0.371109	-2.899640	0.298013
F	-0.327551	-4.616017	2.320057
F	-1.150910	-3.863407	4.803573
F	-2.086599	0.433369	3.184357
F	-2.033473	-1.319360	5.219547
F	1.585653	-0.425486	1.052691
F	3.384877	1.306075	2.011352
F	2.672784	3.880713	2.526232
F	0.122559	4.693137	2.062588

F	-1.704204	2.996569	1.193766
C	2.025032	0.566312	-2.355788
C	3.180215	0.871348	-1.625599
C	1.071681	1.561037	-2.542795
C	3.377927	2.121038	-1.052914
C	1.253177	2.813651	-1.965707
H	0.168891	1.359644	-3.108118
C	2.394946	3.121705	-1.189898
H	4.293072	2.298834	-0.499470
H	0.481064	3.558732	-2.116260
N	2.533711	4.342757	-0.560052
C	3.847294	4.746270	-0.093494
H	3.765070	5.722987	0.385125
H	4.232394	4.045959	0.653494
H	4.578072	4.815096	-0.912606
C	1.543362	5.374086	-0.796739
H	1.531317	5.722722	-1.840299
H	0.541432	5.007892	-0.546566
H	1.758247	6.224142	-0.147394
C	4.065977	-0.293755	-1.507859
C	4.078348	-1.109125	-2.691400
C	4.554756	-0.738937	-0.309410
C	4.683653	-2.404573	-2.576851
C	5.115427	-2.046917	-0.207114
H	4.402682	-0.132025	0.573332
C	5.162324	-2.868788	-1.389249
H	5.595187	-3.859955	-1.336322
N	5.539328	-2.514830	0.972478
C	5.460523	-1.673047	2.163097
H	5.879269	-2.217089	3.007606
H	6.031151	-0.749864	2.022554
H	4.419524	-1.415064	2.388014
C	6.030545	-3.883744	1.102655
H	6.934510	-4.032587	0.503770
H	6.271385	-4.071883	2.147272
H	5.268921	-4.602420	0.784605
H	4.763495	-3.026973	-3.463766
H	4.194592	-0.599009	-3.647073

TS$_{6'}$

E(SMD/M06-2X/6-31g(d)) = -3848.323922 au

E(SMD/M06-2X/def2-TZVP//SMD/M06-2X/6-31g(d)) = -3849.784763 au

H(SMD/M06-2X/6-31g(d)) = -3847.682091 au

G(SMD/M06-2X/6-31g(d)) = -3847.832050 au

S	2.202139	0.603134	-1.910516
C	-0.239399	-1.227572	1.032654
C	-1.168360	-2.179922	1.715919
C	-0.363273	-3.250348	2.100318
C	1.030313	-2.942754	1.635934
O	2.008213	-3.642346	1.766215

O	-0.497815	-0.089486	0.525122
N	1.003187	-1.682616	1.007338
C	-3.041042	-3.261420	2.707826
C	-2.520637	-2.158885	2.018979
C	-0.863167	-4.339001	2.786562
C	-2.230990	-4.332435	3.085142
H	-4.097717	-3.278814	2.954933
H	-3.170114	-1.336829	1.739527
H	-0.221006	-5.163423	3.080555
H	-2.669651	-5.169694	3.618897
B	-1.776321	0.495496	-0.083026
C	-2.758477	1.149806	1.039425
C	-3.887927	1.833715	0.603165
C	-2.524480	1.207979	2.405831
C	-4.750625	2.512952	1.446085
C	-3.361283	1.879825	3.290269
C	-4.479221	2.537771	2.807203
C	-1.154950	1.733049	-0.958532
C	-0.319309	2.628636	-0.295649
C	-1.290380	1.953734	-2.321448
C	0.369426	3.648749	-0.931765
C	-0.607321	2.955705	-3.002864
C	0.236185	3.802203	-2.303353
C	-2.435229	-0.698249	-0.988996
C	-1.615679	-1.343405	-1.911860
C	-3.725293	-1.207614	-0.915736
C	-2.010970	-2.413528	-2.695373
C	-4.172088	-2.276754	-1.685727
C	-3.310803	-2.884804	-2.582041
F	-0.349531	-0.918145	-2.079589
F	-1.160497	-2.987857	-3.550097
F	-4.622976	-0.711954	-0.047062
F	-5.422470	-2.725801	-1.557097
F	-3.723404	-3.910934	-3.322878
F	-1.462151	0.592740	2.950541
F	-3.093247	1.899655	4.598695
F	-5.291809	3.184503	3.642211
F	-4.181585	1.834858	-0.708132
F	-5.827761	3.141545	0.969531
F	-0.132398	2.518933	1.028804
F	1.169752	4.472998	-0.248831
F	0.918799	4.757271	-2.936408
F	-0.758517	3.107526	-4.321251
F	-2.089735	1.179143	-3.072644
C	3.456496	1.395195	-0.964316
C	4.230143	0.523400	-0.183501
C	3.714924	2.765859	-0.925471
C	5.258379	1.006485	0.630728
C	4.737439	3.242776	-0.122792
H	3.131951	3.463269	-1.519770

C	5.540062	2.380230	0.670122
H	5.822317	0.304302	1.233411
H	4.918977	4.310638	-0.109791
N	6.567851	2.885933	1.447605
C	7.171820	2.006314	2.430984
H	7.942626	2.556014	2.972400
H	6.440640	1.618537	3.156363
H	7.655292	1.154150	1.942001
C	6.591319	4.312701	1.721450
H	7.429055	4.528566	2.385389
H	6.744443	4.885005	0.801242
H	5.665772	4.665603	2.199769
C	3.842782	-0.866215	-0.372697
C	2.613754	-0.977631	-1.142870
C	4.457646	-1.991692	0.113978
C	2.353771	-2.257929	-1.770443
H	1.848251	-1.155543	-0.149420
C	4.002762	-3.280323	-0.292632
H	5.319386	-1.898391	0.763531
C	2.983904	-3.369203	-1.315814
H	2.705844	-4.336038	-1.714681
N	4.521299	-4.389033	0.239055
C	5.414586	-4.293163	1.387945
H	5.580762	-5.291271	1.789521
H	6.381183	-3.864551	1.103774
H	4.958706	-3.671681	2.165579
C	4.056159	-5.707908	-0.177732
H	4.205825	-5.853213	-1.250835
H	4.632813	-6.464948	0.351008
H	2.994982	-5.838538	0.061415
H	1.559471	-2.344417	-2.505681

2a'-OMe

E(SMD/M06-2X/6-31g(d)) = -692.072454 au

E(SMD/M06-2X/def2-TZVP//SMD/M06-2X/6-31g(d)) = -692.338019 au

H(SMD/M06-2X/6-31g(d)) = -691.807434 au

G(SMD/M06-2X/6-31g(d)) = -691.863275 au

C	-1.416042	1.821553	-0.511292
C	-0.740079	0.672096	-0.072248
C	-2.797608	1.803366	-0.644188
C	-1.477057	-0.472391	0.226744
C	-3.539900	0.658980	-0.347333
H	-3.316644	2.691808	-0.992426
C	-2.869264	-0.485071	0.090056
H	-0.989206	-1.371734	0.591507
H	-4.617944	0.670946	-0.458371
C	-4.890380	-1.707748	0.299331
H	-5.371792	-0.983869	0.967057
H	-5.213352	-1.524014	-0.731865
H	-5.178370	-2.717747	0.593105

C	0.740080	0.672117	0.072317
C	1.416035	1.821621	0.511247
C	1.477071	-0.472374	-0.226627
C	2.797598	1.803451	0.644140
C	2.869294	-0.485006	-0.090077
H	0.989216	-1.371749	-0.591305
C	3.539907	0.659065	0.347314
H	4.617936	0.671034	0.458450
C	4.890371	-1.707879	-0.299394
H	5.213412	-1.524027	0.731751
H	5.178239	-2.717944	-0.593068
H	5.371845	-0.984160	-0.967260
H	3.316629	2.691914	0.992332
H	-0.855413	2.714380	-0.769984
H	0.855397	2.714467	0.769851
O	3.479274	-1.653118	-0.414631
O	-3.479287	-1.653132	0.414666

ip1'-OME

E(SMD/M06-2X/6-31g(d)) = -4322.347315 au

E(SMD/M06-2X/def2-TZVP//SMD/M06-2X/6-31g(d)) = -4324.002889 au

H(SMD/M06-2X/6-31g(d)) = -4321.656839 au

G(SMD/M06-2X/6-31g(d)) = -4321.823931 au

C	-3.560609	-1.855678	-0.054058
C	-4.933720	-2.391314	-0.257171
C	-5.561844	-1.652615	-1.255183
C	-4.621720	-0.613006	-1.751624
O	-4.774993	0.221002	-2.603707
O	-2.710810	-2.178828	0.733920
N	-3.433561	-0.792800	-0.987967
S	-2.082443	0.198480	-1.096283
C	-6.919750	-3.648582	0.067397
C	-5.593122	-3.399251	0.426502
C	-6.878037	-1.888789	-1.617532
C	-7.551423	-2.907251	-0.937016
H	-7.472666	-4.430830	0.577663
H	-5.099239	-3.963348	1.211323
H	-7.364466	-1.299802	-2.389362
H	-8.584146	-3.127710	-1.189343
C	1.458368	-1.497545	1.449778
C	1.044385	-2.760531	2.125900
C	-0.154382	-2.454291	2.753229
C	-0.418567	-1.006904	2.435408
O	-1.362065	-0.354760	2.839830
O	2.563826	-1.482682	0.796903
N	0.610041	-0.508762	1.615057
C	0.902841	-4.989887	2.913563
C	1.604438	-4.022516	2.182118
C	-0.857294	-3.398736	3.475071
C	-0.303222	-4.684248	3.547680

H	1.301357	-5.997054	2.987238
H	2.537493	-4.255058	1.678256
H	-1.798446	-3.158614	3.960547
H	-0.821212	-5.460627	4.102445
B	3.110596	-0.297447	0.026920
C	4.544168	-0.838697	-0.560959
C	5.760754	-0.169179	-0.523700
C	4.570233	-2.060630	-1.229139
C	6.930916	-0.679192	-1.073901
C	5.714771	-2.607519	-1.790392
C	6.908992	-1.908357	-1.709835
C	2.196451	0.023412	-1.289650
C	2.581122	1.069066	-2.124951
C	1.142231	-0.744721	-1.766048
C	1.948811	1.385852	-3.314551
C	0.477787	-0.462213	-2.956284
C	0.874302	0.611652	-3.731418
C	3.272763	0.939084	1.090330
C	2.642277	2.173929	1.048067
C	4.068289	0.743603	2.216157
C	2.763576	3.141003	2.037938
C	4.227955	1.678844	3.227008
C	3.563496	2.892809	3.139054
F	1.832370	2.510969	0.024031
F	2.106762	4.301856	1.931863
F	4.750672	-0.405149	2.355624
F	5.011531	1.425563	4.278506
F	3.695308	3.806153	4.100790
F	3.439087	-2.769906	-1.376862
F	5.677159	-3.789067	-2.413002
F	8.020980	-2.411954	-2.246354
F	5.872451	1.035408	0.059055
F	8.074322	0.008273	-0.998044
F	3.628122	1.834489	-1.768451
F	2.359175	2.413349	-4.064102
F	0.241549	0.895705	-4.869944
F	-0.562199	-1.211082	-3.340976
F	0.692850	-1.820079	-1.102162
C	-2.279323	1.286352	0.436523
C	-3.550468	2.048435	0.373396
C	-1.035975	2.077703	0.386610
C	-3.529422	3.366108	0.021395
C	-1.030729	3.392162	0.079917
H	-0.114190	1.528141	0.569102
C	-2.289907	4.042604	-0.151240
H	-4.438172	3.953266	-0.061001
H	-0.104431	3.950962	0.035606
H	-2.227097	0.587960	1.279636
C	-1.200106	6.102587	-0.649203
H	-0.658345	6.165326	0.296788

H	-0.571087	5.680898	-1.435605
H	-1.567200	7.083515	-0.942491
C	-4.802093	1.345533	0.705230
C	-5.970518	1.614814	-0.011192
C	-4.823797	0.406105	1.752040
C	-7.153330	0.933222	0.287328
H	-5.965027	2.303880	-0.850314
C	-6.015666	-0.234297	2.064257
H	-3.931201	0.191072	2.332179
C	-7.180493	0.010021	1.338591
H	-6.041132	-0.950407	2.880067
H	-8.088358	-0.527786	1.586700
C	-9.436601	0.558367	-0.228887
H	-9.799457	0.807266	0.774613
H	-10.147126	0.921777	-0.971477
H	-9.332877	-0.528567	-0.322482
O	-2.384135	5.295135	-0.484628
O	-8.209862	1.215578	-0.507670

TS$_{2'\text{-OMe}}$

E(SMD/M06-2X/6-31g(d)) = -4322.336796 au

E(SMD/M06-2X/def2-TZVP//SMD/M06-2X/6-31g(d)) = -4323.988026 au

H(SMD/M06-2X/6-31g(d)) = -4321.647986 au

G(SMD/M06-2X/6-31g(d)) = -4321.806710 au

C	1.890393	-2.633320	1.647000
C	1.744183	-3.354507	0.354151
C	1.829685	-2.428568	-0.681863
C	1.936993	-1.059922	-0.103879
O	1.945576	0.002745	-0.663586
O	1.902052	-3.066111	2.768430
N	2.075279	-1.263233	1.300076
S	2.351190	-0.068214	2.437297
C	1.639110	-5.121014	-1.230294
C	1.637039	-4.713815	0.107750
C	1.855423	-2.822565	-2.008055
C	1.758333	-4.193262	-2.269565
H	1.554790	-6.176869	-1.467771
H	1.564467	-5.430436	0.919915
H	1.936430	-2.095318	-2.810930
H	1.755425	-4.539644	-3.298889
C	-1.124327	-0.326804	2.032232
C	-2.393525	-0.575527	2.822891
C	-2.004717	-0.440078	4.155733
C	-0.532239	-0.158145	4.153658
O	0.172005	0.026033	5.121042
O	-0.955673	-0.361943	0.760612
N	-0.094351	-0.134256	2.824241
C	-4.597881	-1.099131	3.579907
C	-3.699083	-0.933968	2.517171
C	-2.877595	-0.603335	5.212262

C	-4.203393	-0.928987	4.905056
H	-5.625790	-1.370088	3.358917
H	-4.036870	-1.092946	1.502155
H	-2.540458	-0.490488	6.238337
H	-4.927828	-1.063351	5.702268
B	-1.676846	0.117508	-0.490543
C	-0.936261	1.465308	-1.054335
C	-0.047153	2.270347	-0.352772
C	-1.334402	1.967509	-2.288833
C	0.435002	3.476603	-0.853753
C	-0.862610	3.148523	-2.835712
C	0.035084	3.911568	-2.104561
C	-1.571005	-1.175461	-1.490060
C	-2.203806	-2.353670	-1.103304
C	-0.842779	-1.280817	-2.668221
C	-2.151418	-3.544109	-1.808616
C	-0.776618	-2.448916	-3.420560
C	-1.418952	-3.591629	-2.984467
C	-3.212272	0.611405	-0.192660
C	-3.419339	1.588212	0.777892
C	-4.358229	0.215178	-0.871485
C	-4.661522	2.103585	1.109153
C	-5.625439	0.705486	-0.575201
C	-5.779290	1.652666	0.423641
F	-2.370733	2.050132	1.484724
F	-4.793516	3.020213	2.069808
F	-4.304063	-0.688099	-1.861947
F	-6.693740	0.277557	-1.252117
F	-6.986967	2.130632	0.719010
F	-2.233241	1.278170	-3.018095
F	-1.272636	3.567115	-4.038323
F	0.488588	5.066937	-2.596100
F	0.369453	1.943935	0.874917
F	1.260042	4.235706	-0.124184
F	-2.940283	-2.366582	0.022320
F	-2.778173	-4.636459	-1.364231
F	-1.295820	-4.733195	-3.661204
F	0.008894	-2.508276	-4.502520
F	-0.099870	-0.267279	-3.137953
C	4.022028	2.372027	-0.484056
C	4.561627	1.914678	0.719031
C	3.958686	3.741678	-0.751047
C	5.035729	2.834474	1.668262
C	4.443691	4.658373	0.187270
C	4.979120	4.192140	1.387140
H	5.479466	2.490836	2.596896
H	4.400560	5.724706	-0.000467
H	3.607223	1.679528	-1.209352
C	4.663546	0.466772	0.982523
C	4.952944	-0.430133	-0.011588

C	4.373901	-0.044004	2.322481
C	5.068758	-1.812295	0.281111
H	5.104843	-0.117109	-1.039644
C	4.732232	-1.417198	2.615465
H	4.534460	0.658431	3.142375
C	5.019642	-2.301186	1.619942
H	5.207847	-3.345661	1.836550
C	3.378519	5.466350	-2.265398
H	4.391215	5.885653	-2.270556
H	2.955609	5.532608	-3.267139
H	2.750221	6.026973	-1.565240
H	5.358999	4.907669	2.109733
C	5.318091	-4.009082	-0.602529
H	5.354585	-4.411677	-1.612958
H	6.224739	-4.280829	-0.056287
H	4.429431	-4.374749	-0.081535
H	4.676657	-1.750590	3.647117
O	5.245028	-2.584830	-0.764818
O	3.404243	4.086166	-1.937698

7. References

(1) Gao, W.-C.; Tian, J.; Shang, Y.-Z.; Jiang, X. Steric and Stereoscopic Disulfide Construction for Cross-Linkage via N-Dithiophthalimides. *Chem. Sci.* **2020**, *11*, 3903–3908.

(2) Dong, Y.; Takata, Y.; Yoshigoe, Y.; Sekine, K.; Kuninobu, Y. Lewis Acid-Catalyzed Synthesis of Silafluorene Derivatives from Biphenyls and Dihydrosilanes via a Double Sila-Friedel–Crafts Reaction. *Chem. Commun.* **2019**, *55*, 13303–13306.

(3) Shibahara, F.; Kanai, T.; Yamaguchi, E.; Kamei, A.; Yamauchi, T.; Murai, T. Copper-Catalyzed C−H Bond Direct Chalcogenation of Aromatic Compounds Leading to Diaryl Sulfides, Selenides, and Diselenides by Using Elemental Sulfur and Selenium as Chalcogen Sources Under Oxidative Conditions. *Chem. Asian J.* **2014**, *9*, 237–244.

(4) Prasad, D. J. C.; Sekar, G. Cu-Catalyzed One-Pot Synthesis of Unsymmetrical Diaryl Thioethers by Coupling of Aryl Halides Using a Thiol Precursor. *Org. Lett.* **2011**, *13*, 1008–1011.

(5) Wang, X.; Chen, J.-Q.; Yang, X.-X.; Hao, E.-J.; Dong, Z.-B. Synthesis of Diaryl Sulfides by Using Tetramethylthiuram Monosulfide (TMTM) as Organosulfur Source: A Practical C(Sp^2)−S Bond Construction. *Eur. J. Org. Chem.* **2022**, *2022*, e202200015.

(6) Bell, K. H. Chlorosulfination of Aromatic Methyl Ethers with Thionyl Chloride. *Aust. J. Chem.* **1985**, *38*, 1209–1221.

(7) Cironis, N.; Yuan, K.; Thomas, S. P.; Ingleson, M. J. XtalFluor-E Enabled Regioselective Synthesis of Di-Indole Sulfides by C3−H Sulfenylation of Indoles. *Eur. J. Org. Chem.* **2022**, *2022*, e202101394.

(8) Gaussian 09, Revision D.01, Frisch, M. J.; Trucks, G. W.; Schlegel, H. B.; Scuseria, G. E.; Robb, M. A.; Cheeseman, J. R.; Scalmani, G.; Barone, V.; Mennucci, B.; Petersson, G. A.; Nakatsuji, H.; Caricato, M.; Li, X.; Hratchian, H. P.; Izmaylov, A. F.; Bloino, J.; Zheng, G.; Sonnenberg, J. L.; Hada, M.; Ehara, M.; Toyota, K.; Fukuda, R.; Hasegawa, J.; Ishida, M.; Nakajima, T.; Honda, Y.; Kitao, O.; Nakai, H.; Vreven, T.; Montgomery, J. A.; Peralta, Jr., J. E.; Ogliaro, F.; Bearpark, M.; Heyd, J. J.; Brothers, E.; Kudin, K. N.; Staroverov, V. N.; Keith, T.; Kobayashi, R.; Normand, J.; Raghavachari, K.; Rendell, A.; Burant, J. C.; Iyengar, S. S.; Tomasi, J.; Cossi, M.; Rega, N.; Millam, J. M.; Klene, M.; Knox, J. E.; Cross, J. B.; Bakken, V.; Adamo, C.; Jaramillo, J.; Gomperts, R.; Stratmann, R. E.; Yazyev, O.; Austin, A. J.; Cammi, R.; Pomelli, C.; Ochterski, J. W.; Martin, R. L.; Morokuma, K.; Zakrzewski, V. G.; Voth, G. A.; Salvador, P.; Dannenberg, J. J.; Dapprich, S.; Daniels, A. D.; Farkas, O.; Foresman, J. B.; Ortiz, J. V.; Cioslowski, J.; and Fox, D. J. Gaussian, Inc., Wallingford CT, **2013**.

(9) Hariharan, P. C.; Pople, J. A. The Influence of Polarization Functions on Molecular Orbital Hydrogenation Energies. *Theoret. Chim. Acta* **1973**, *28*, 213–222.

(10) Marenich, A. V.; Cramer, C. J.; Truhlar, D. G. Universal Solvation Model Based on Solute Electron Density and on a Continuum Model of the Solvent Defined by the Bulk Dielectric Constant and Atomic Surface Tensions. *J. Phys. Chem. B* **2009**, *113*, 6378–6396.

(11) Weigend, F.; Furche, F.; Ahlrichs, R.Gaussian Basis Sets of Quadruple Zeta Valence Quality for Atoms H–Kr. *J. Chem. Phys.* **2003**, *119*, 12753–12762.

(12) Ochterski, J. W. Thermochemistry in Gaussian, Gaussian, Inc., Wallingford, CT, **2000**.

(13) Legault, C. Y. CYLview, 1.0b; Université de Sherbrooke: Québec, Canada, **2009**; http://www.cylview.org

8. NMR Spectra

Figure S8: ^{1}**H NMR** (400 MHz, $CDCl_3$, 298 K) spectrum of **1a**.

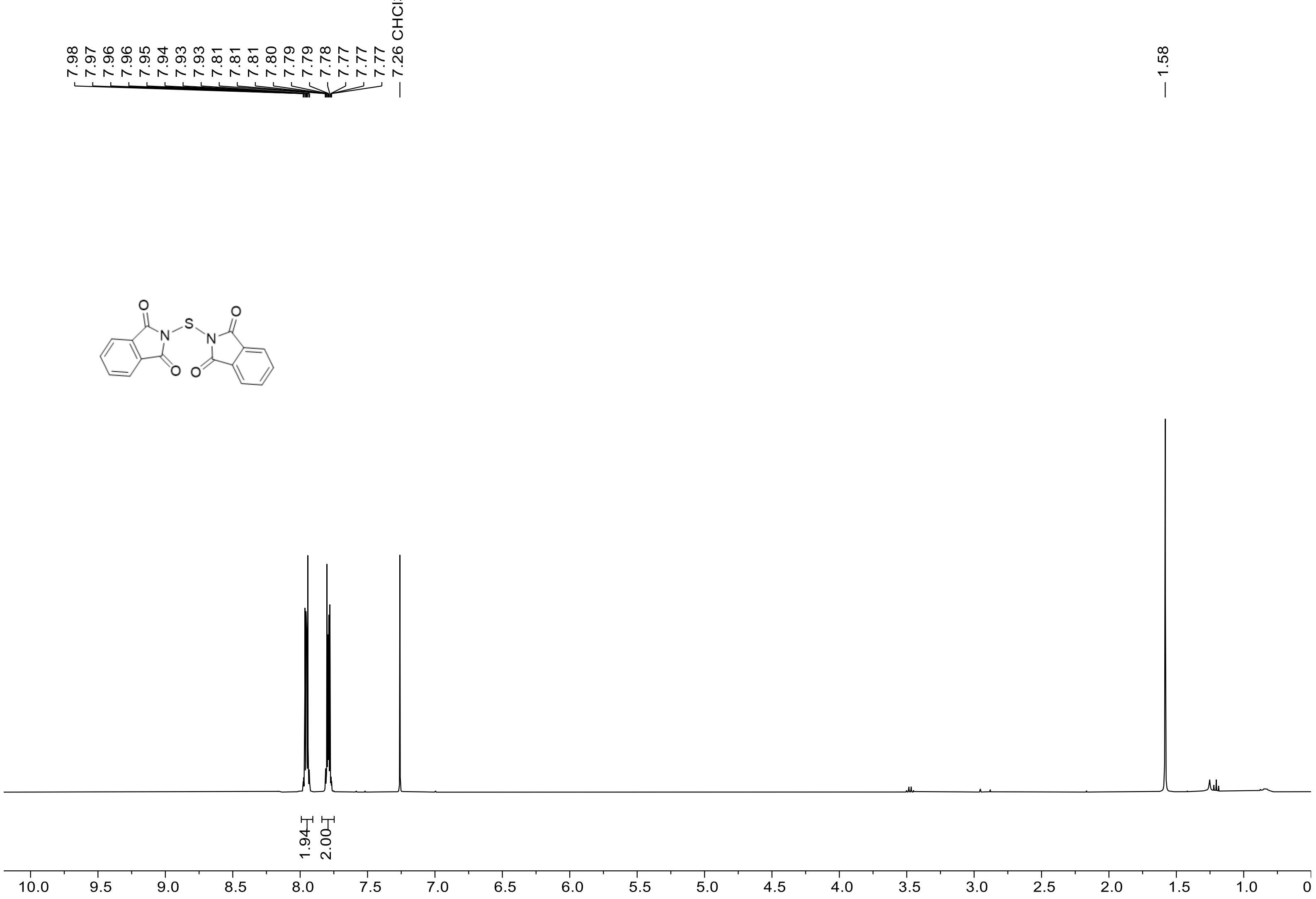

Figure S9: **^{13}C NMR** (101 MHz, $CDCl_3$, 298 K) spectrum of **1a**.

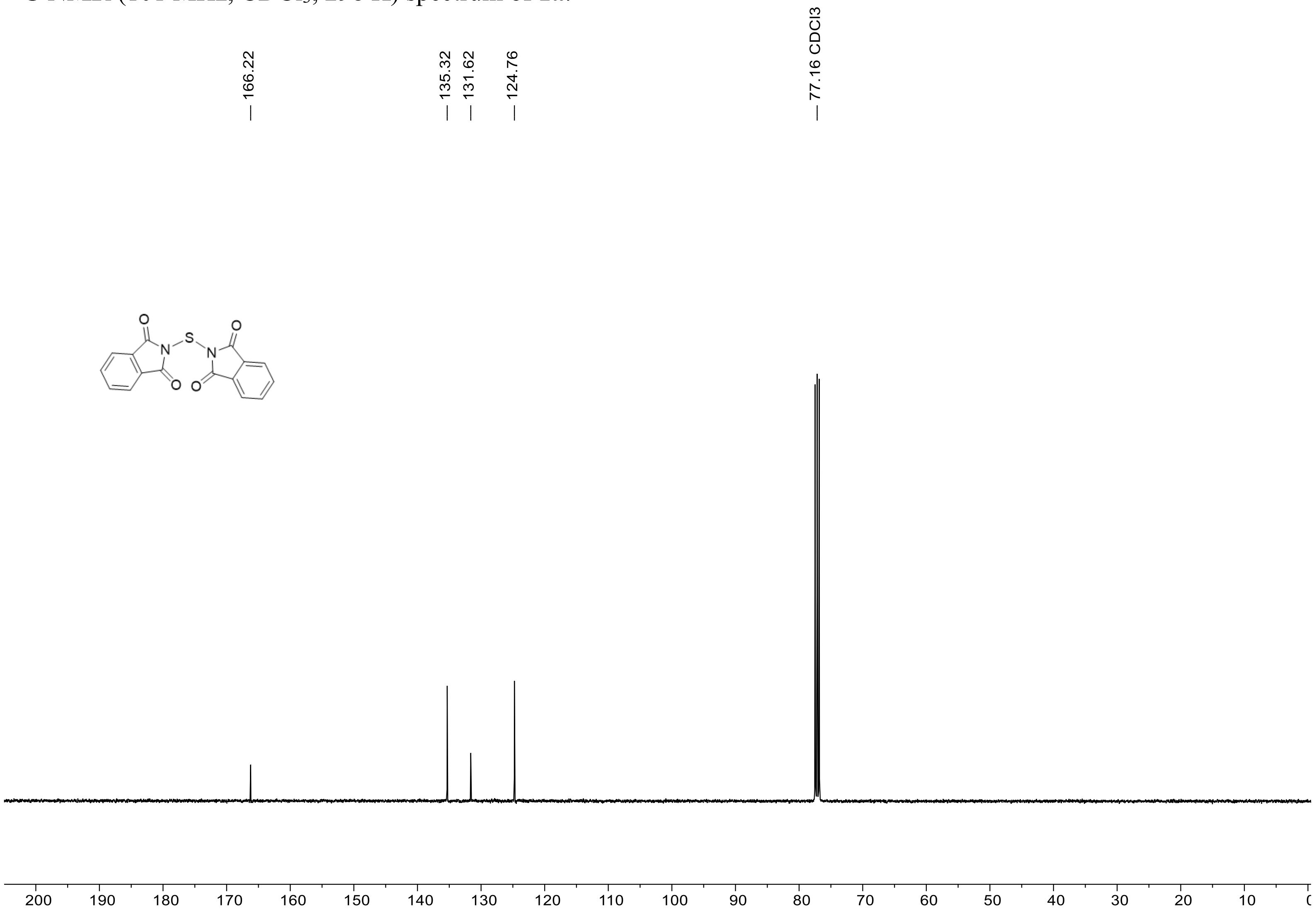

Figure S10: **^{1}H NMR** (400 MHz, $CDCl_3$, 298 K) spectrum of **5a**.

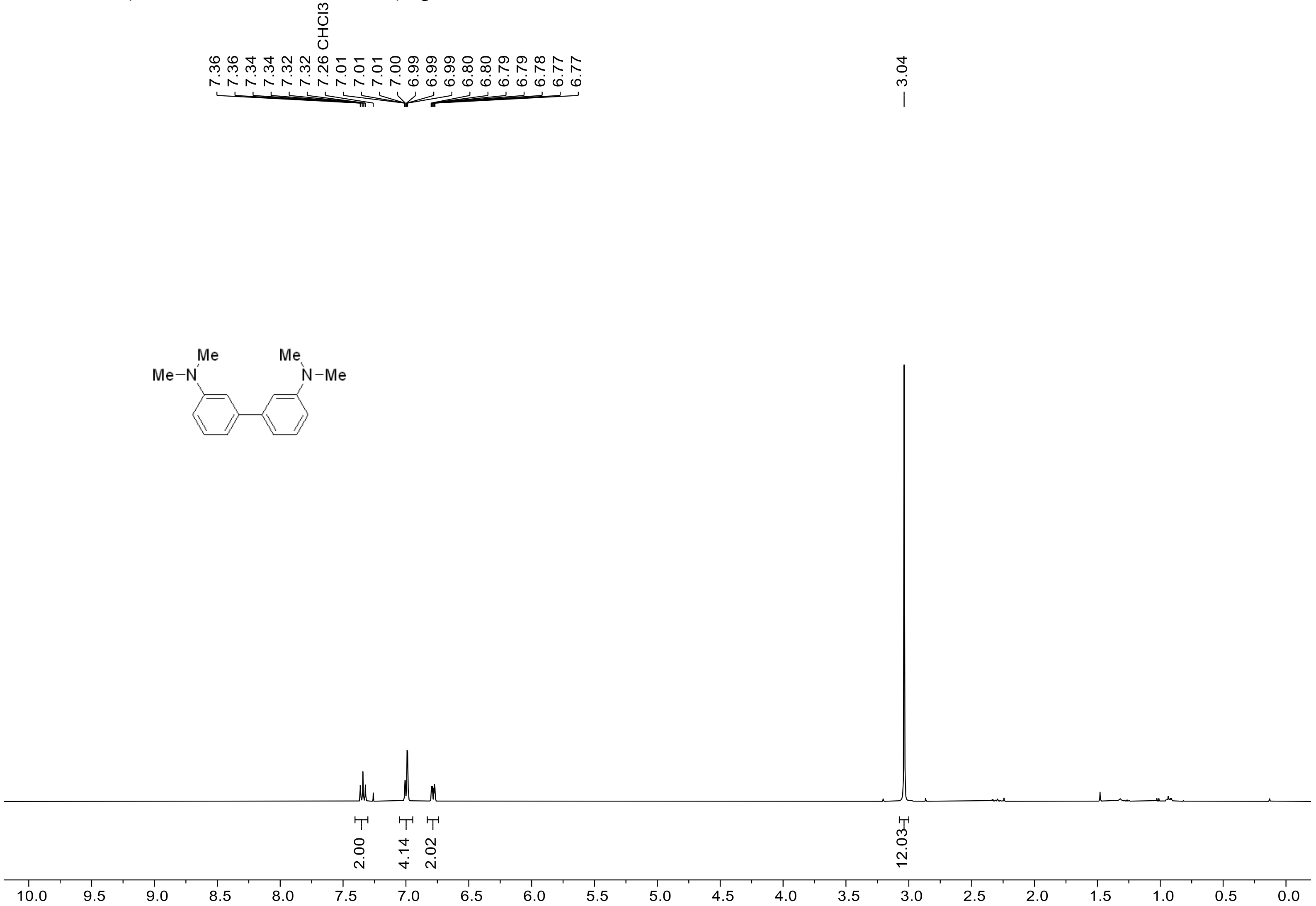

Figure S11: **^{13}C NMR** (101 MHz, $CDCl_3$, 298 K) spectrum of **5a**.

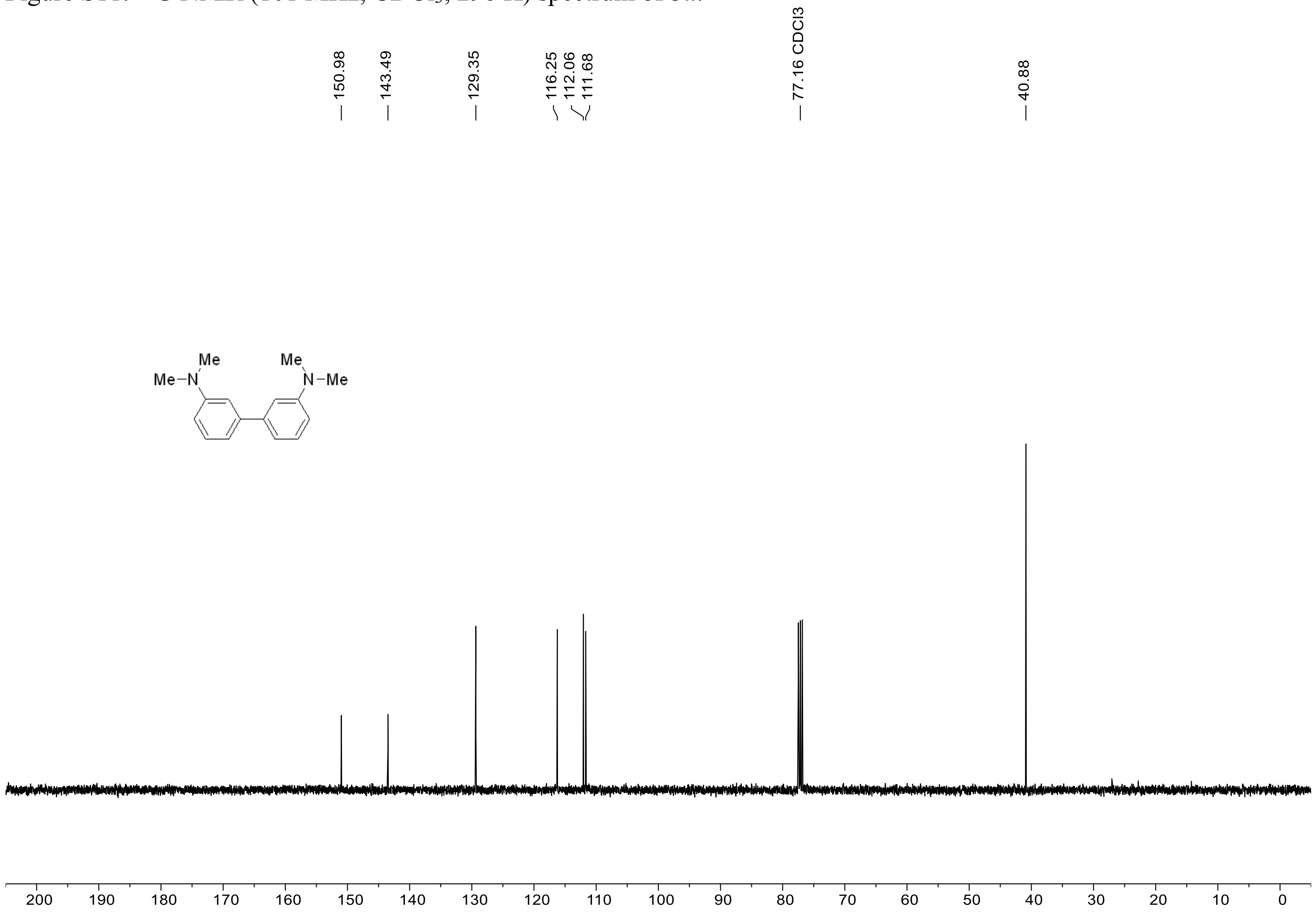

Figure S12: **^{1}H NMR** (400 MHz, $CDCl_3$, 298 K) spectrum of **3aa**.

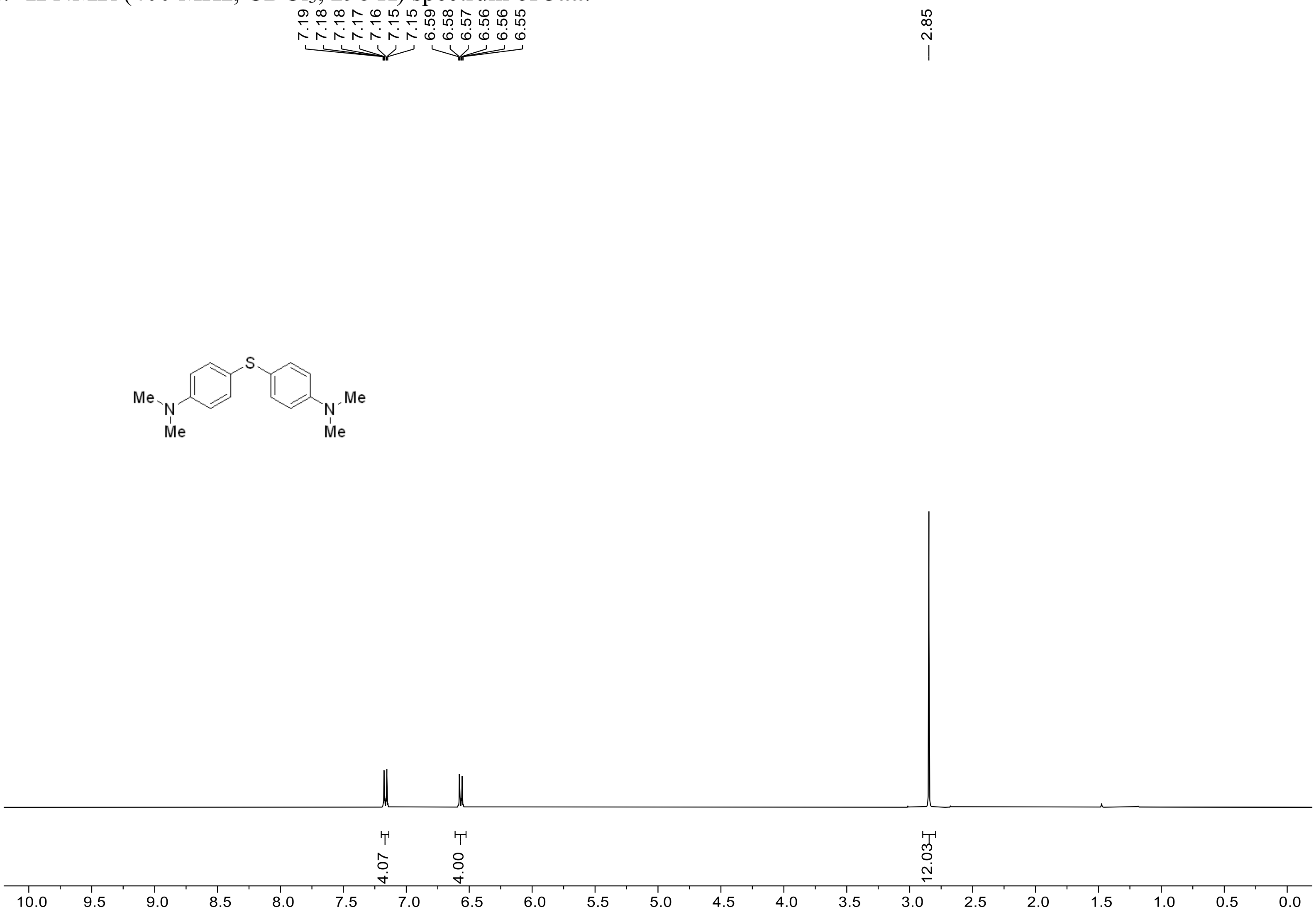

Figure S13: **^{13}C NMR** (101 MHz, $CDCl_3$, 298 K) spectrum of **3aa**.

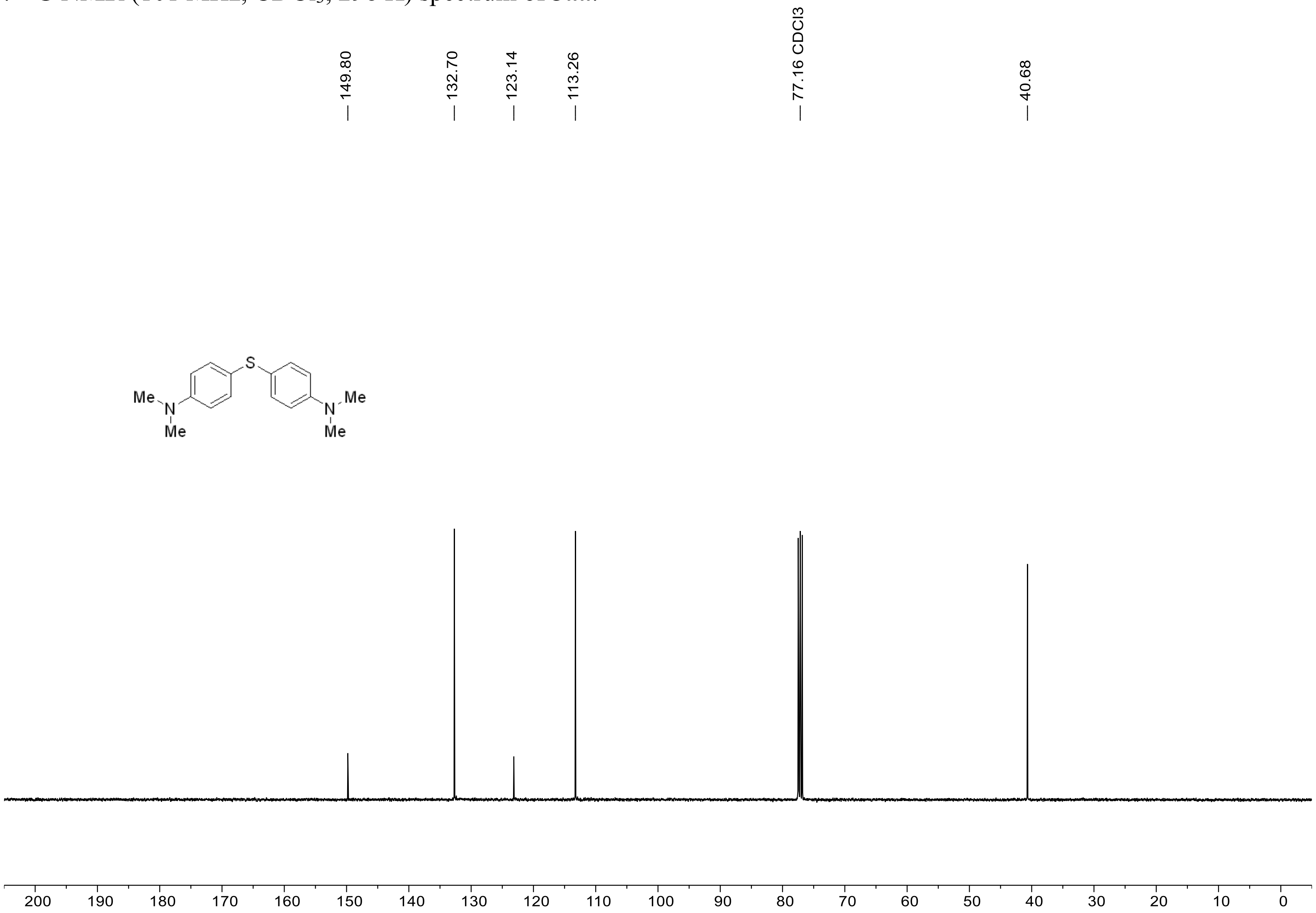

Figure S14: **^{1}H NMR** (400 MHz, $CDCl_3$, 298 K) spectrum of **3ab**.

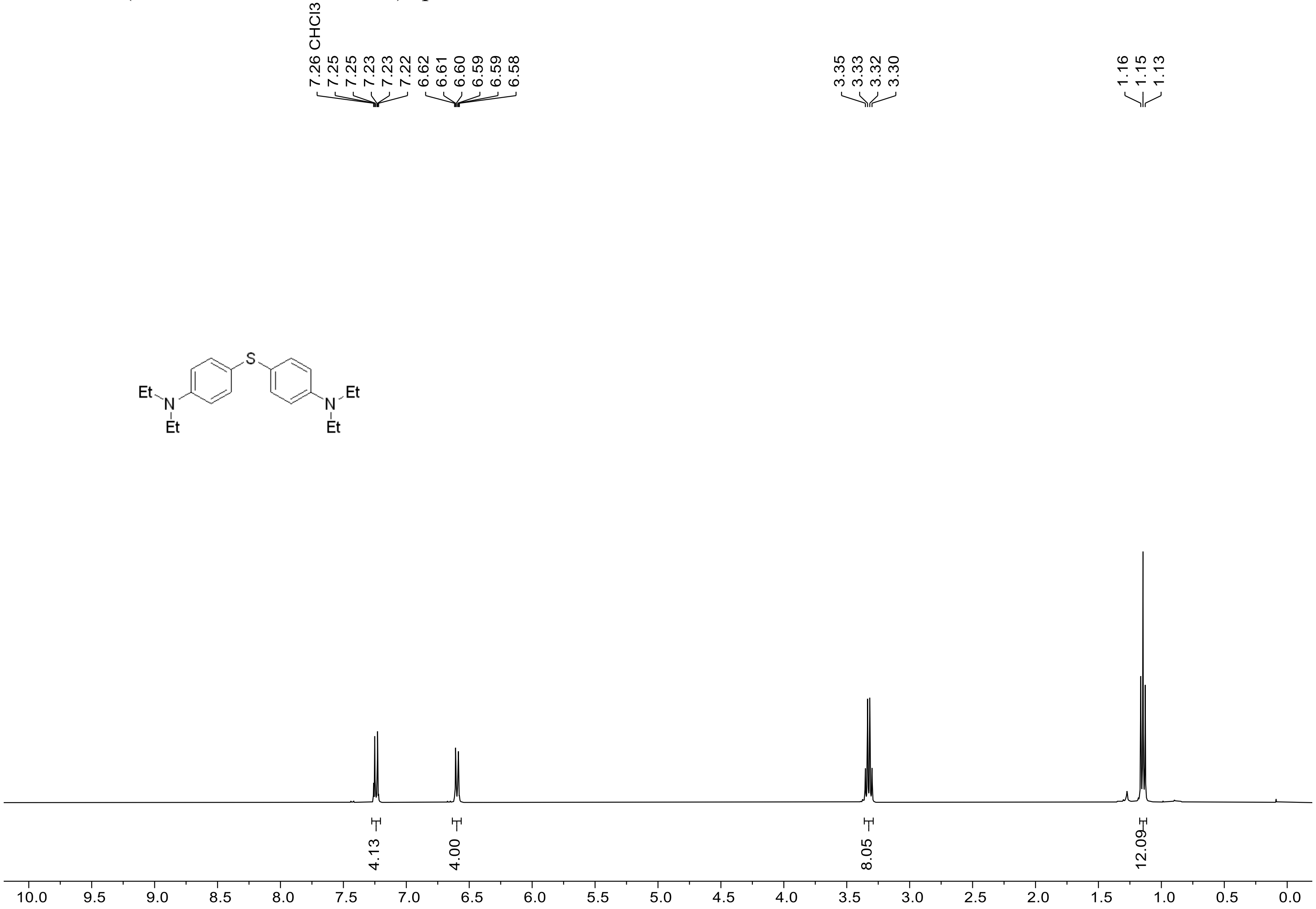

Figure S15: **^{13}C NMR** (101 MHz, $CDCl_3$, 298 K) spectrum of **3ab**.

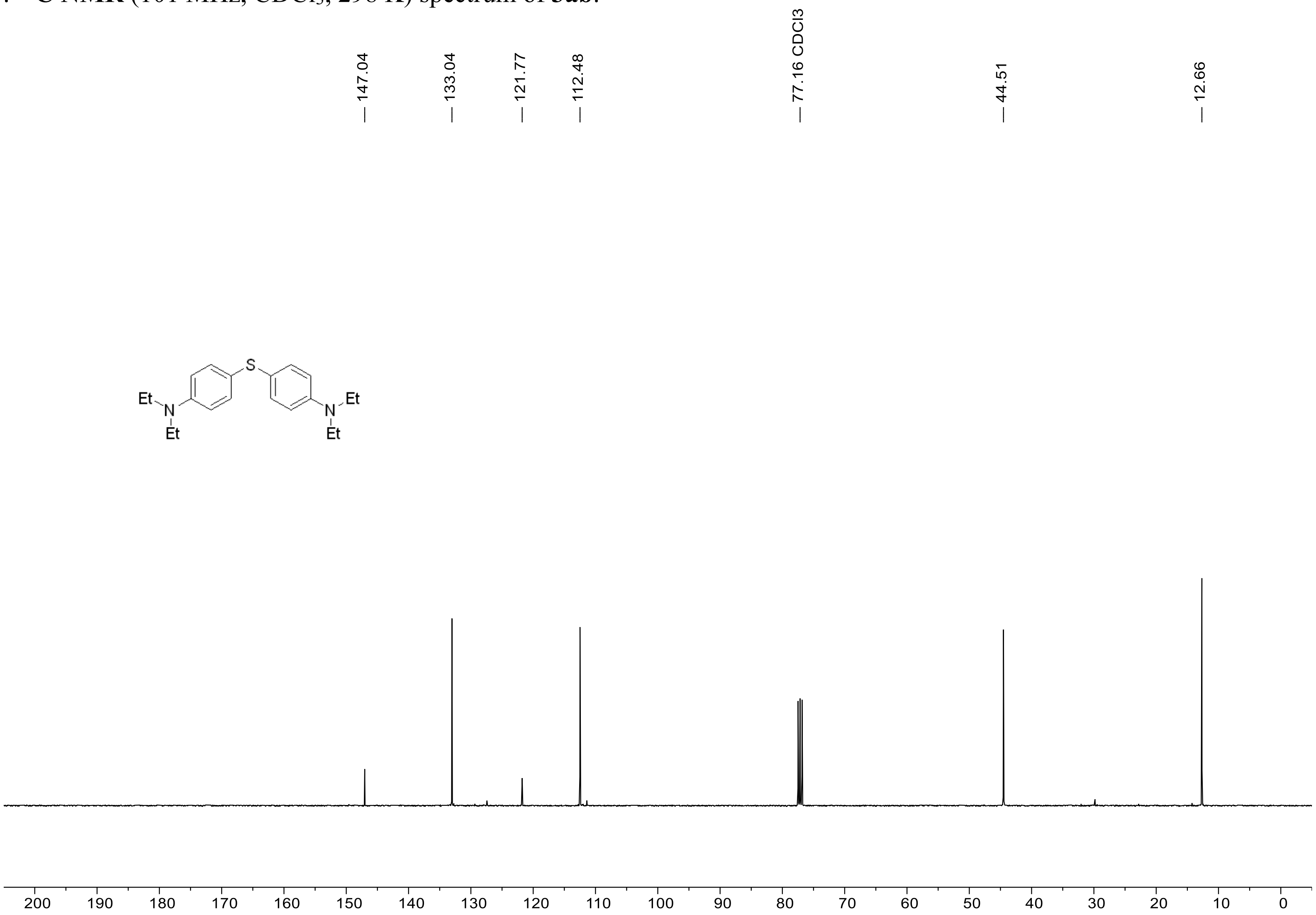

Figure S16: **¹H NMR** (400 MHz, $CDCl_3$, 298 K) spectrum of **3ac**.

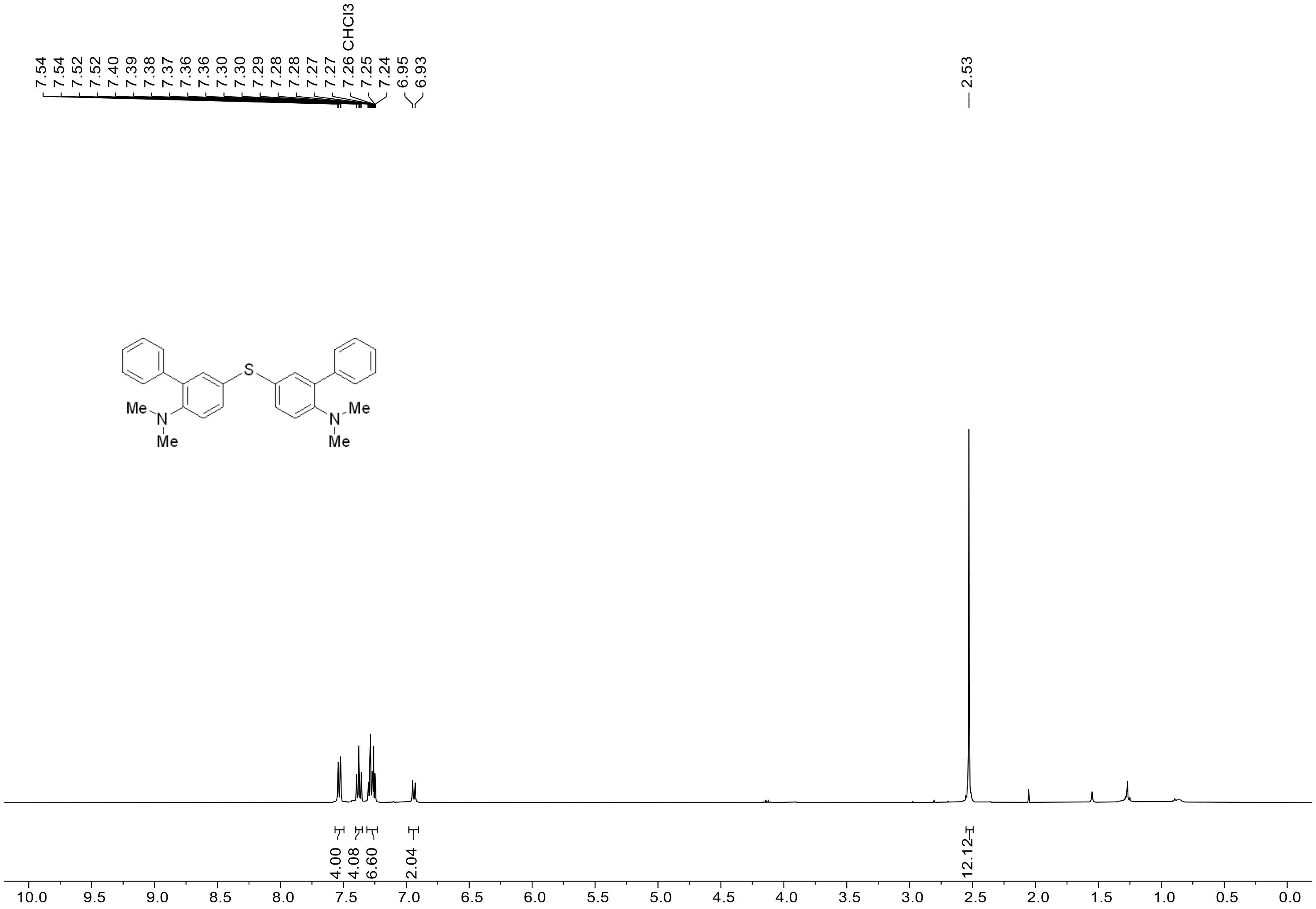

Figure S17: **^{13}C NMR** (101 MHz, $CDCl_3$, 298 K) spectrum of **3ac**.

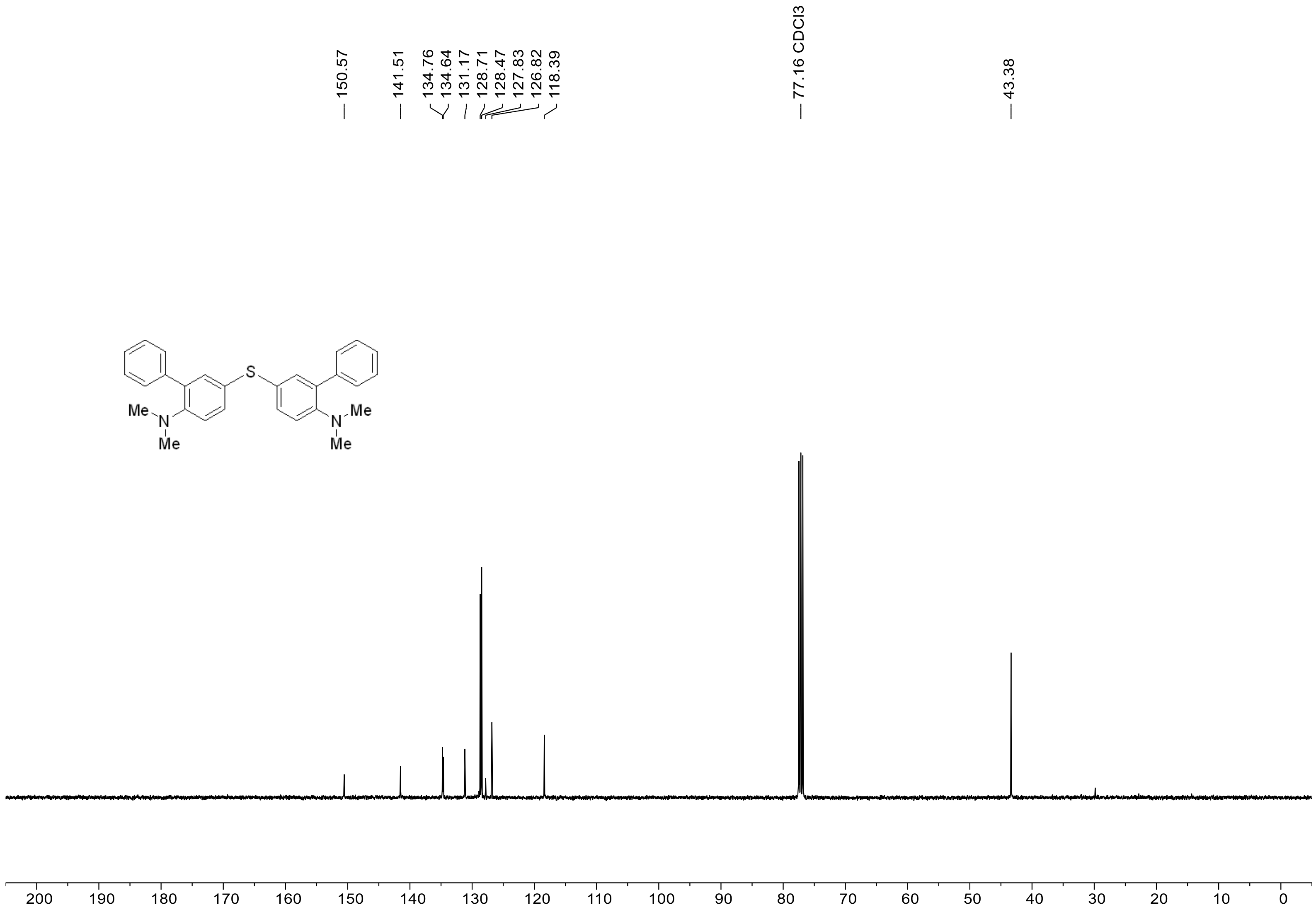

Figure S18: **^{1}H NMR** (400 MHz, $CDCl_3$, 298 K) spectrum of **3ad**.

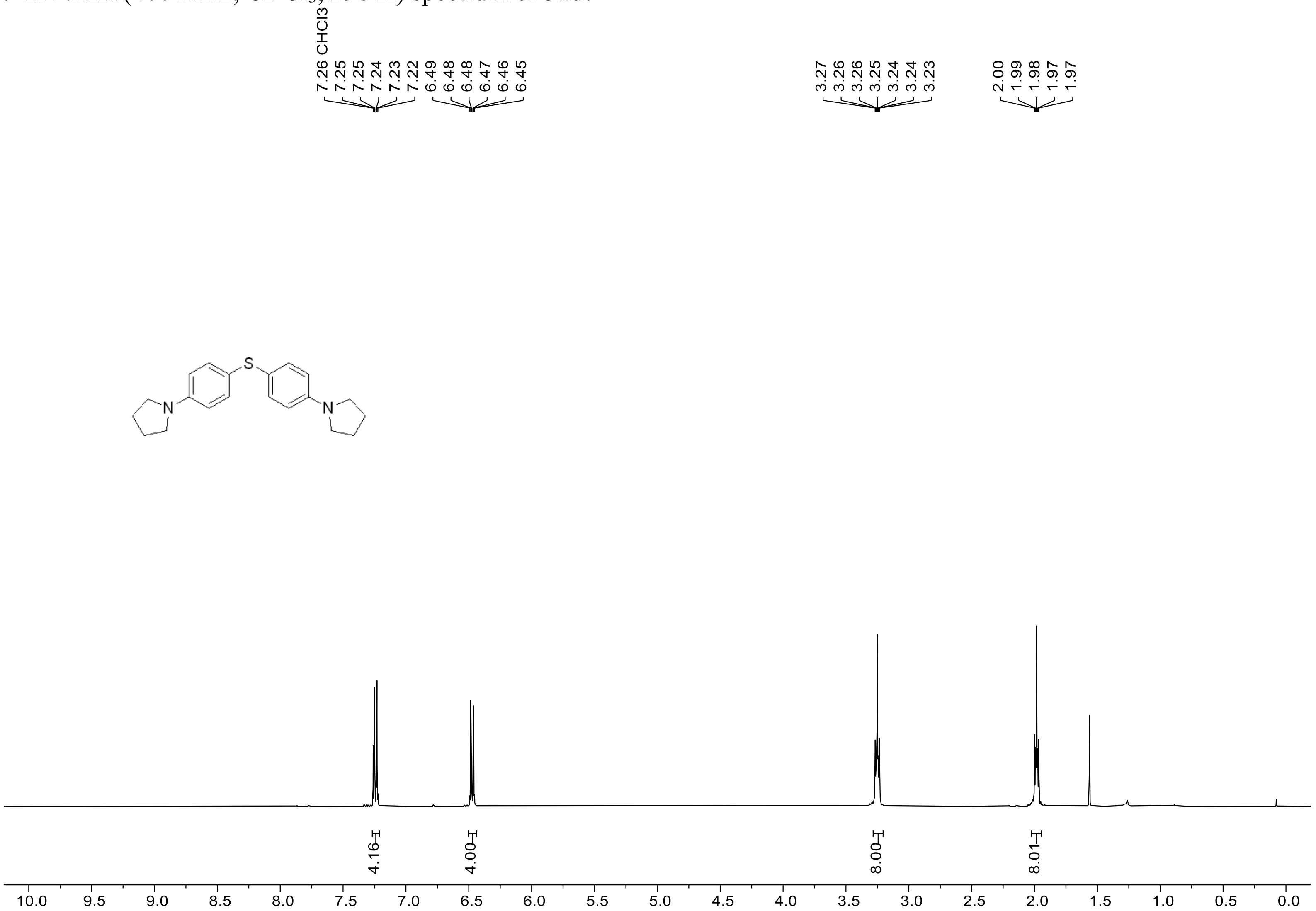

Figure S19: **^{13}C NMR** (101 MHz, $CDCl_3$, 298 K) spectrum of **3ad**.

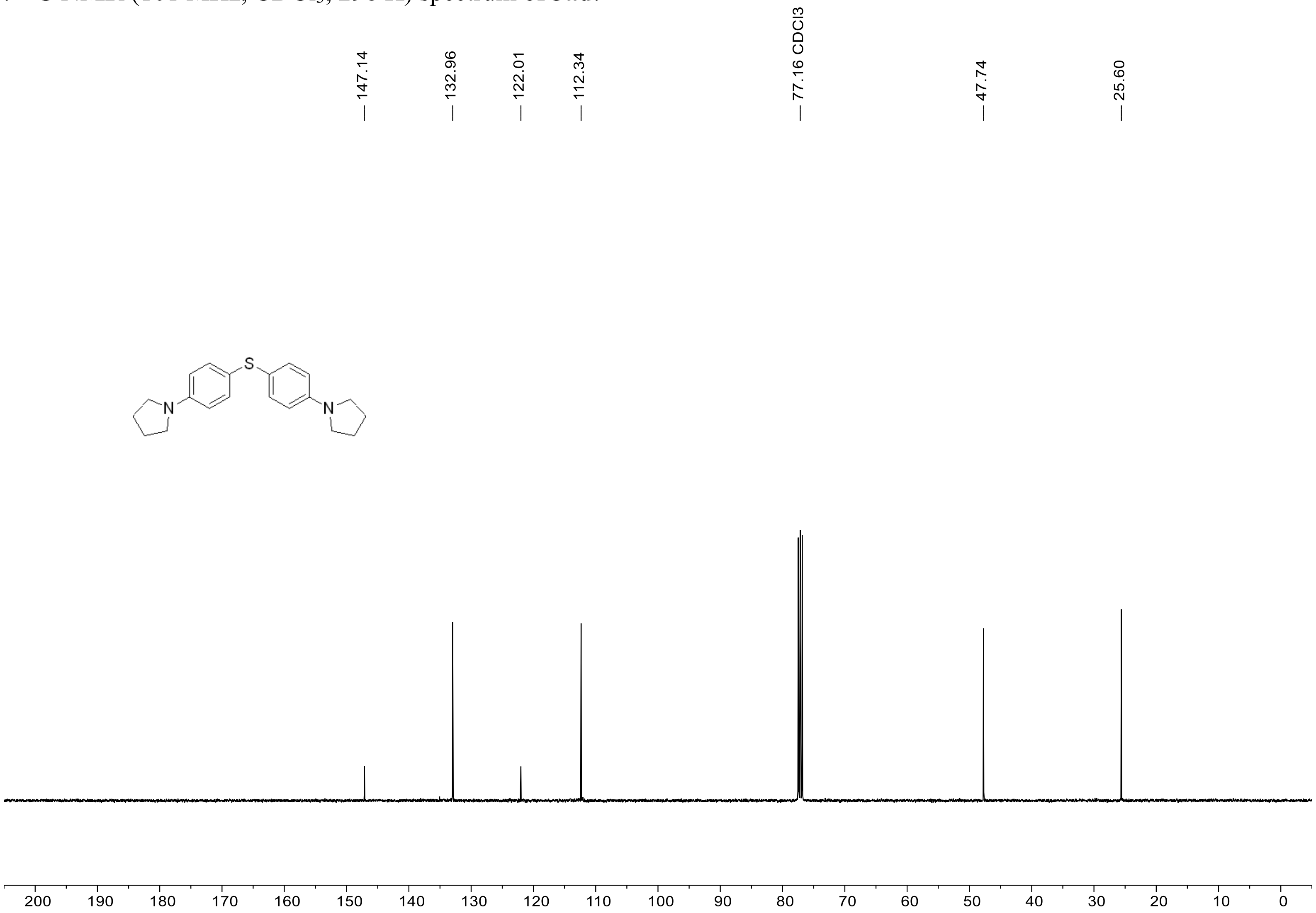

Figure S20: **^{1}H NMR** (400 MHz, $CDCl_3$, 298 K) spectrum of **3ae**.

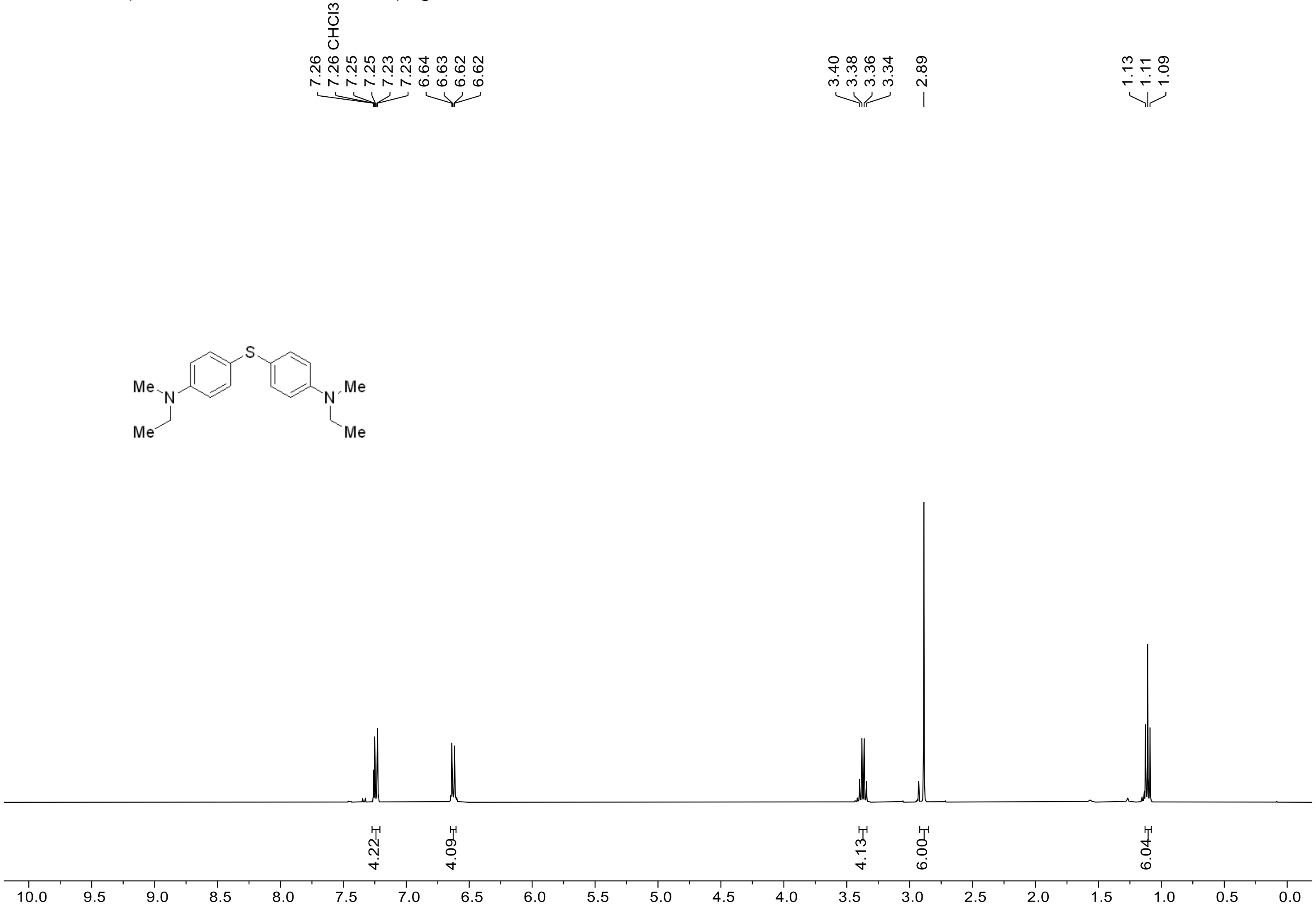

Figure S21: **^{13}C NMR** (101 MHz, $CDCl_3$, 298 K) spectrum of **3ae**.

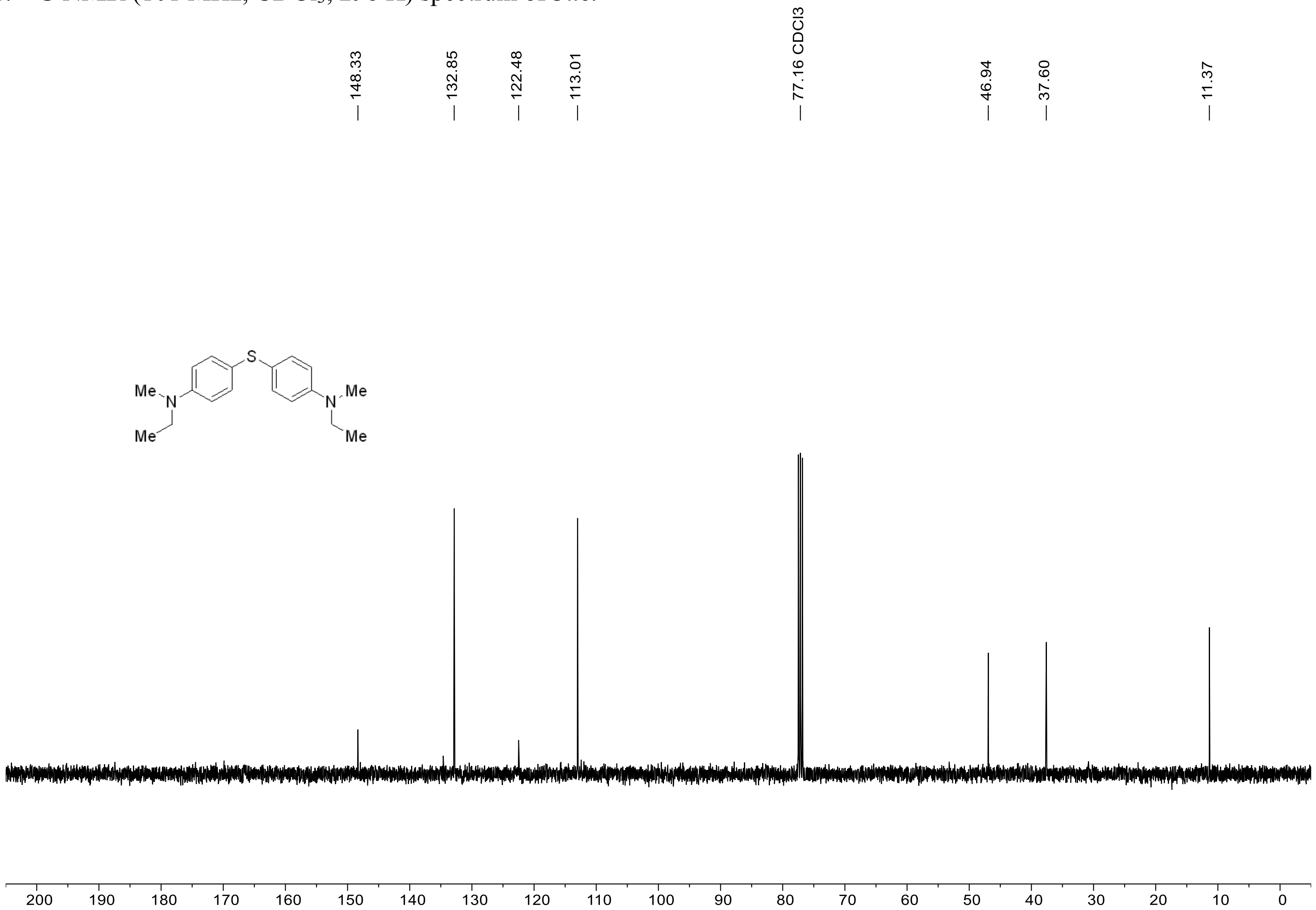

Figure S22: **^{1}H NMR** (400 MHz, $CDCl_3$, 298 K) spectrum of **3af**.

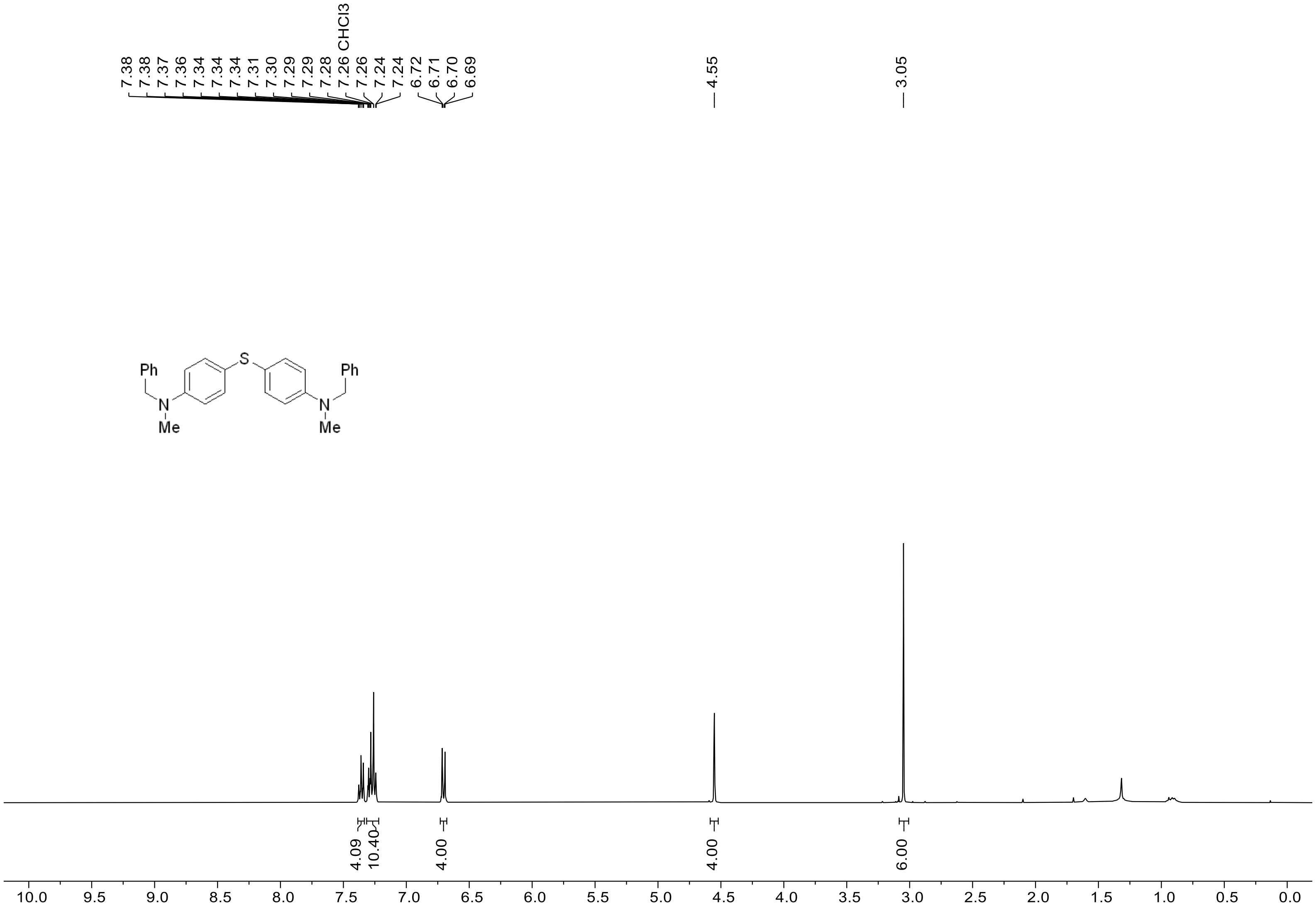

Figure S23: **^{13}C NMR** (101 MHz, $CDCl_3$, 298 K) spectrum of **3af**.

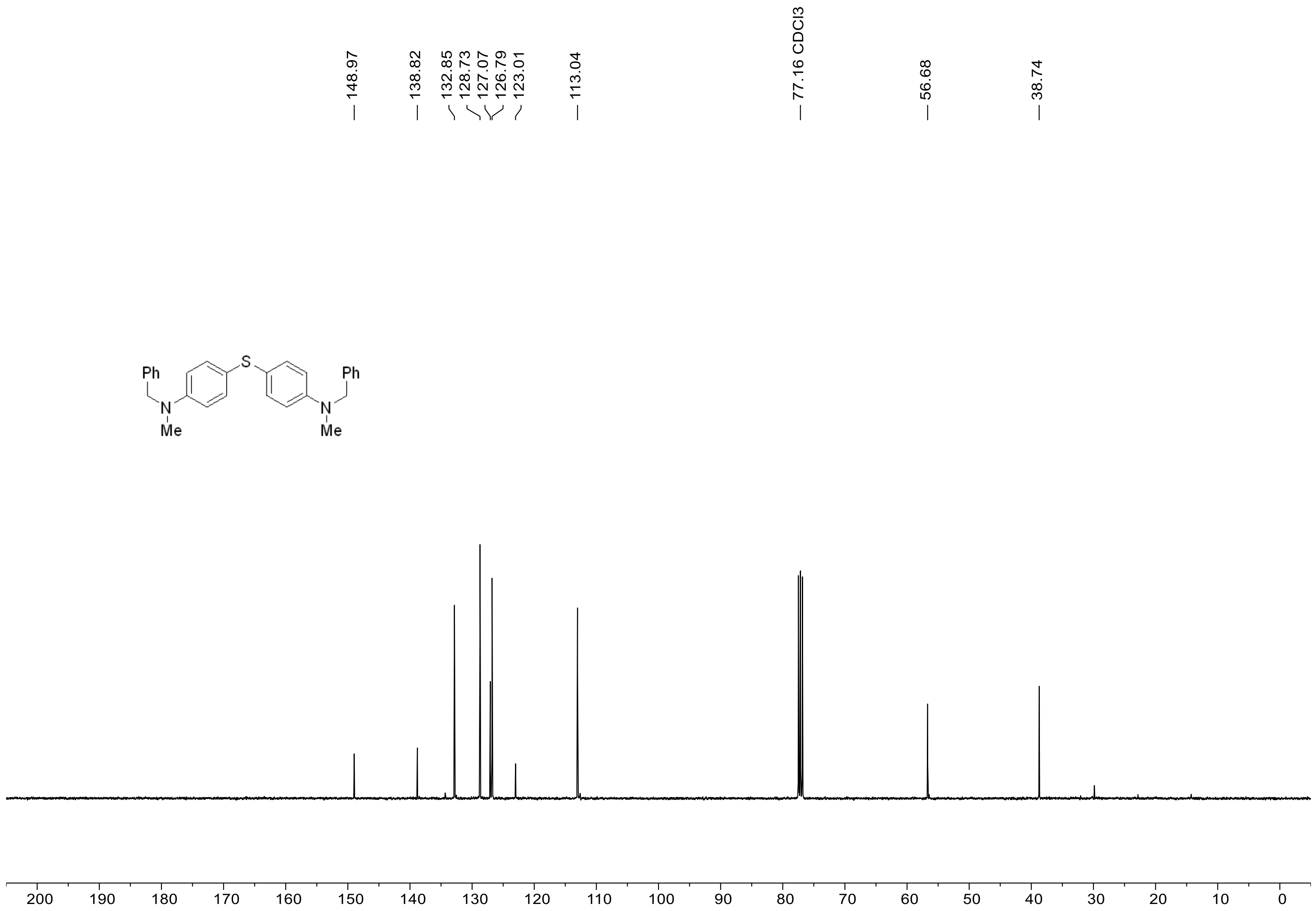

Figure S24: **^{1}H NMR** (400 MHz, $CDCl_3$, 298 K) spectrum of **3ag**.

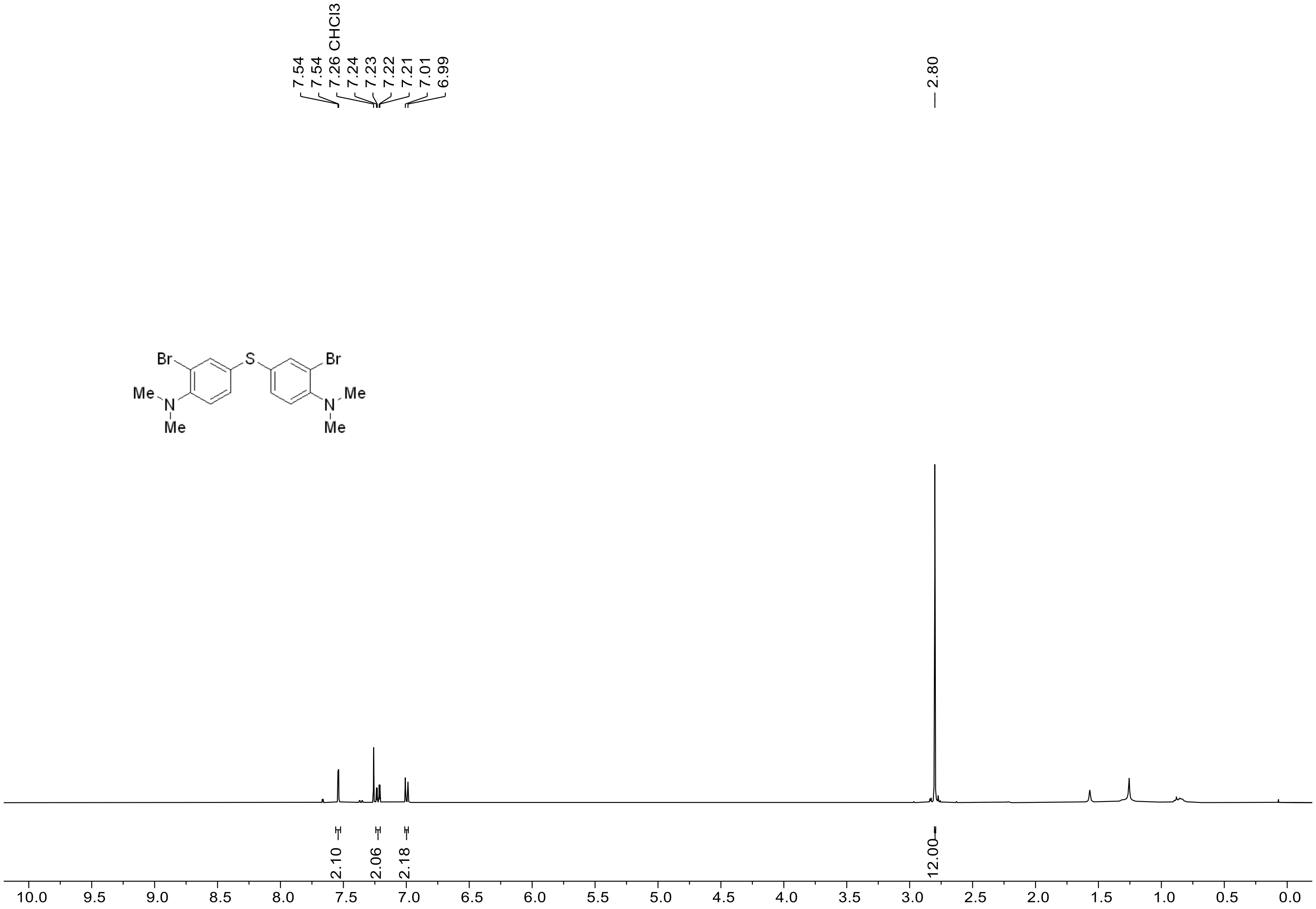

Figure S25: **^{13}C NMR** (101 MHz, $CDCl_3$, 298 K) spectrum of **3ag**.

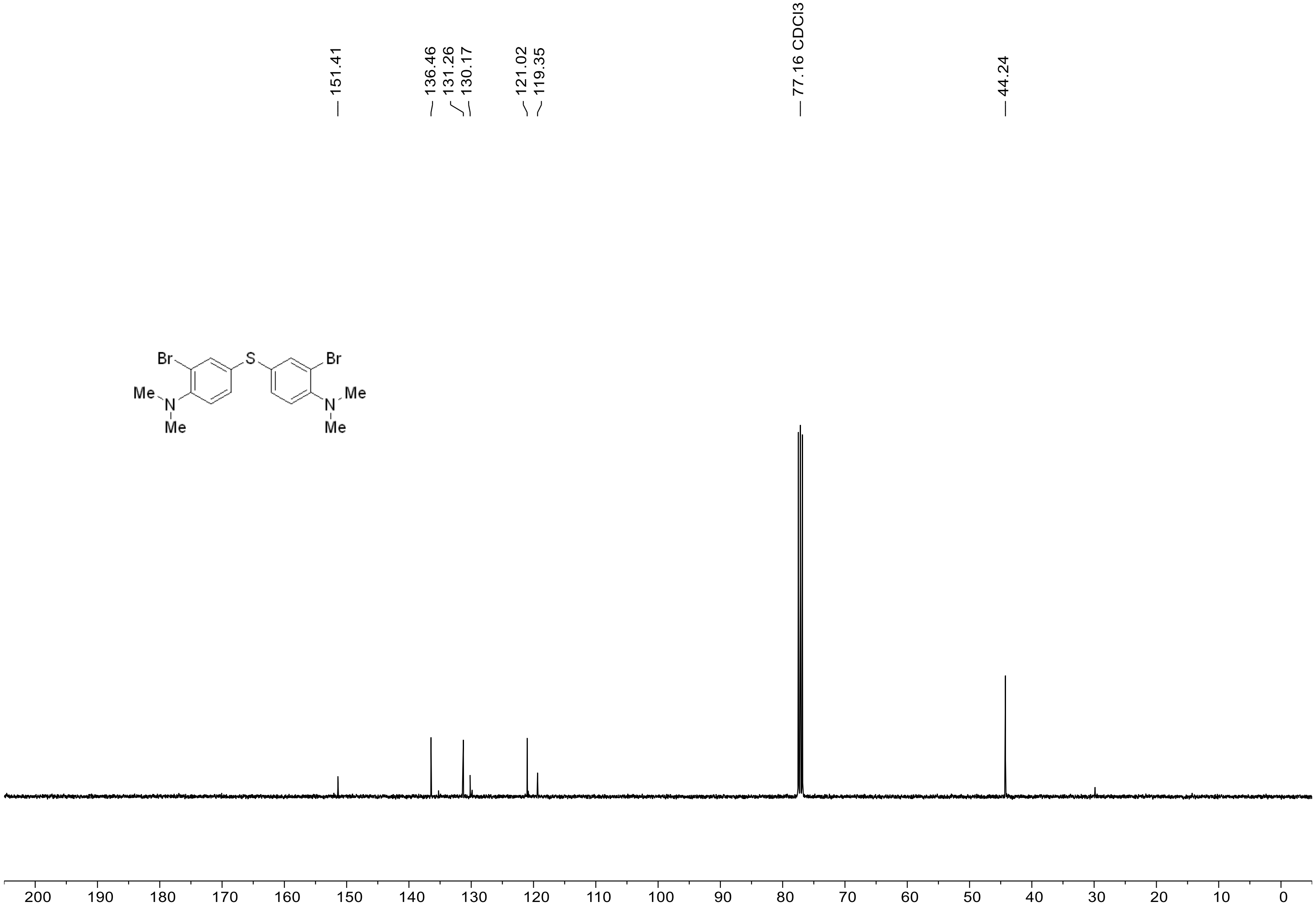

Figure S26: **^{1}H NMR** (400 MHz, $CDCl_3$, 298 K) spectrum of **3ah**.

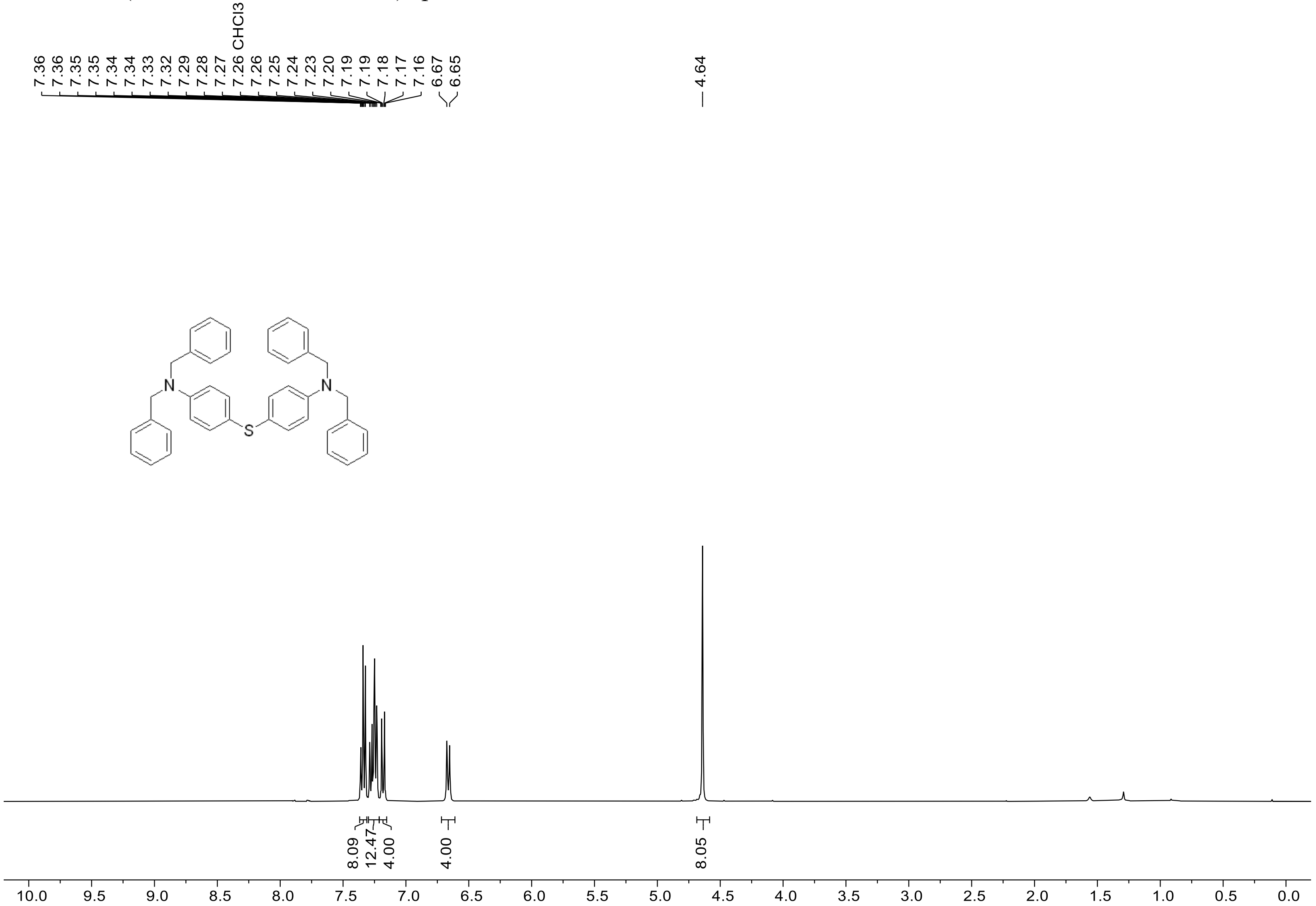

Figure S27: **^{13}C NMR** (101 MHz, $CDCl_3$, 298 K) spectrum of **3ah**.

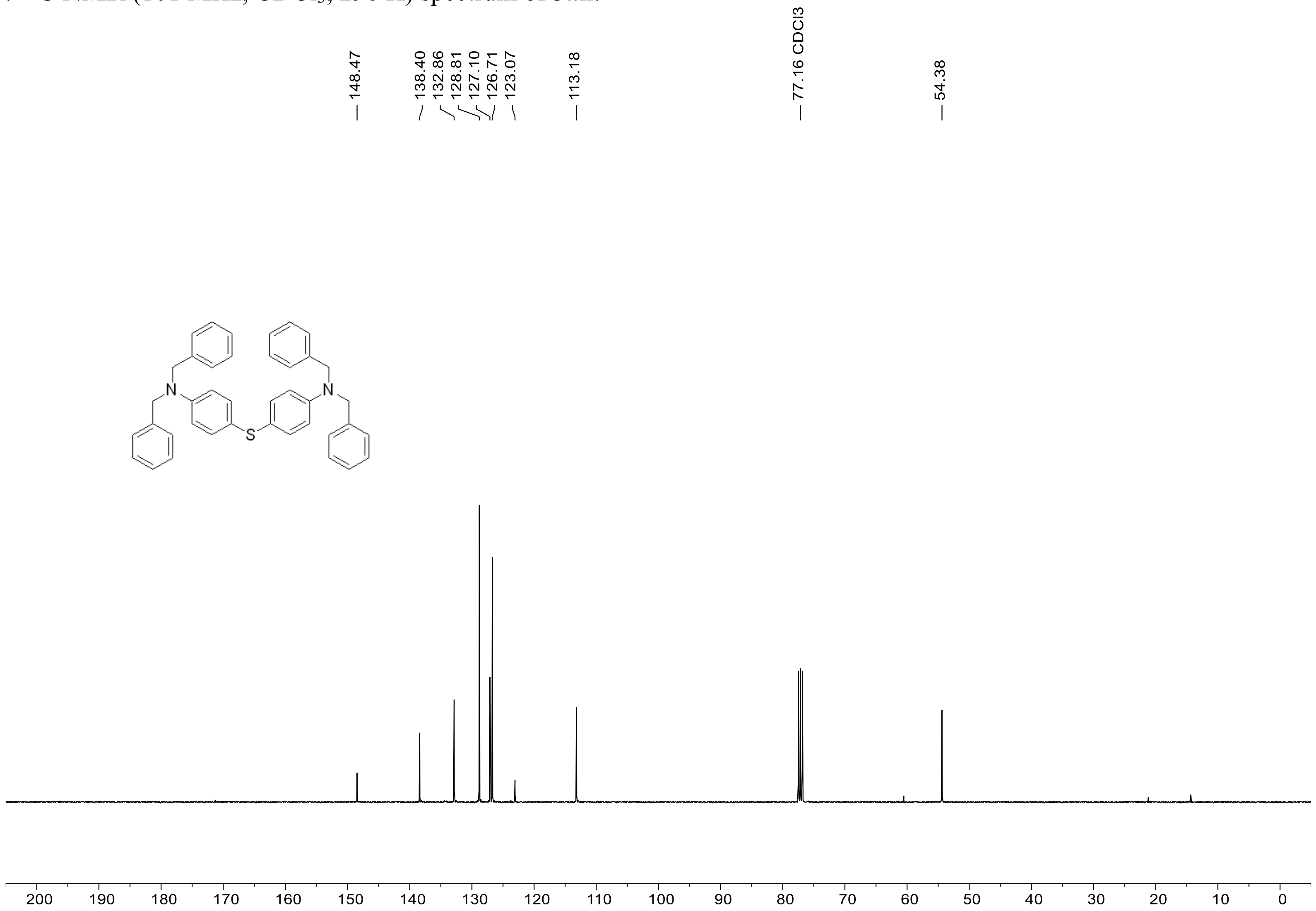

Figure S28: **^{1}H NMR** (400 MHz, $CDCl_3$, 298 K) spectrum of **3ai**.

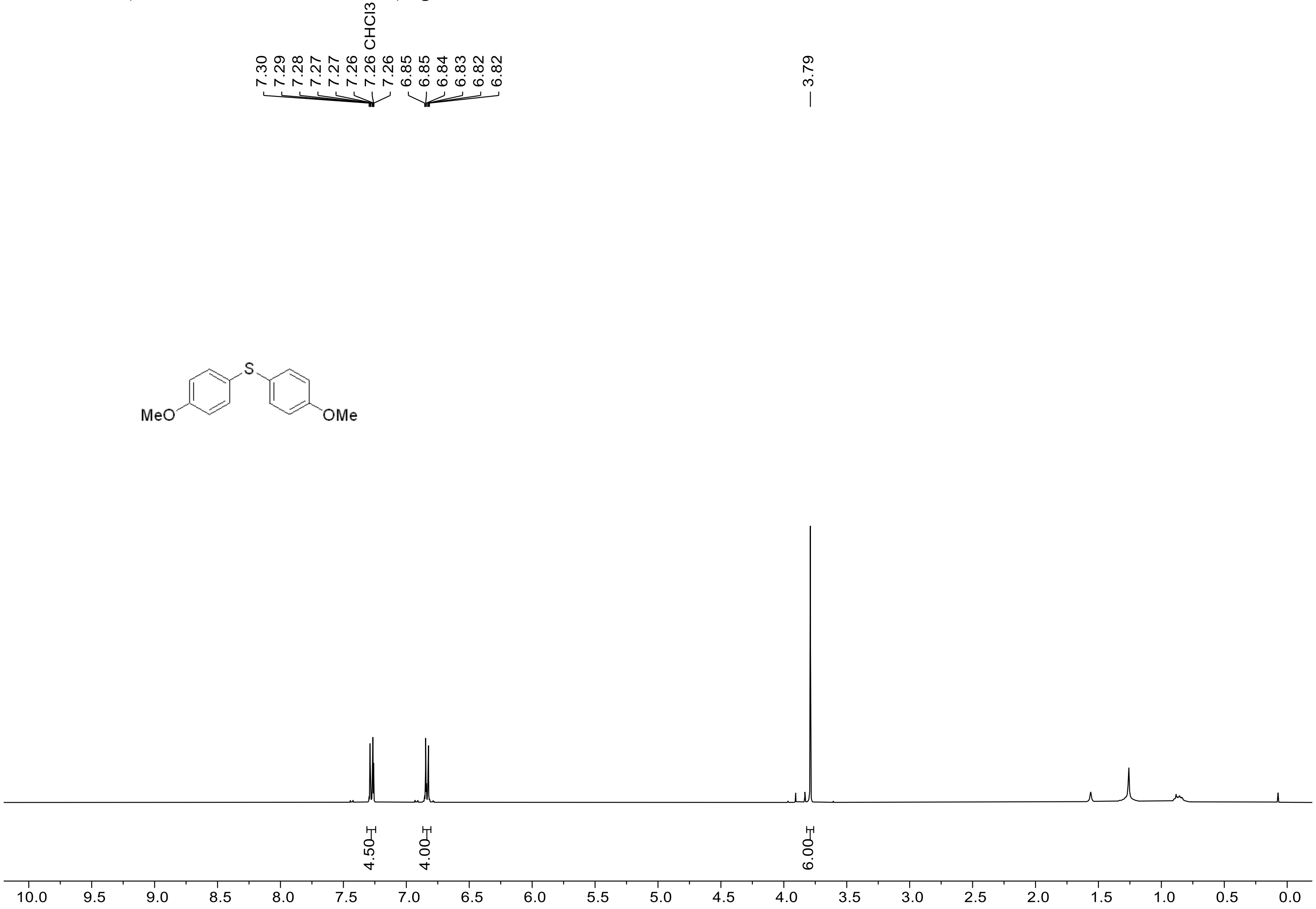

Figure S29: **^{13}C NMR** (101 MHz, $CDCl_3$, 298 K) spectrum of **3ai**.

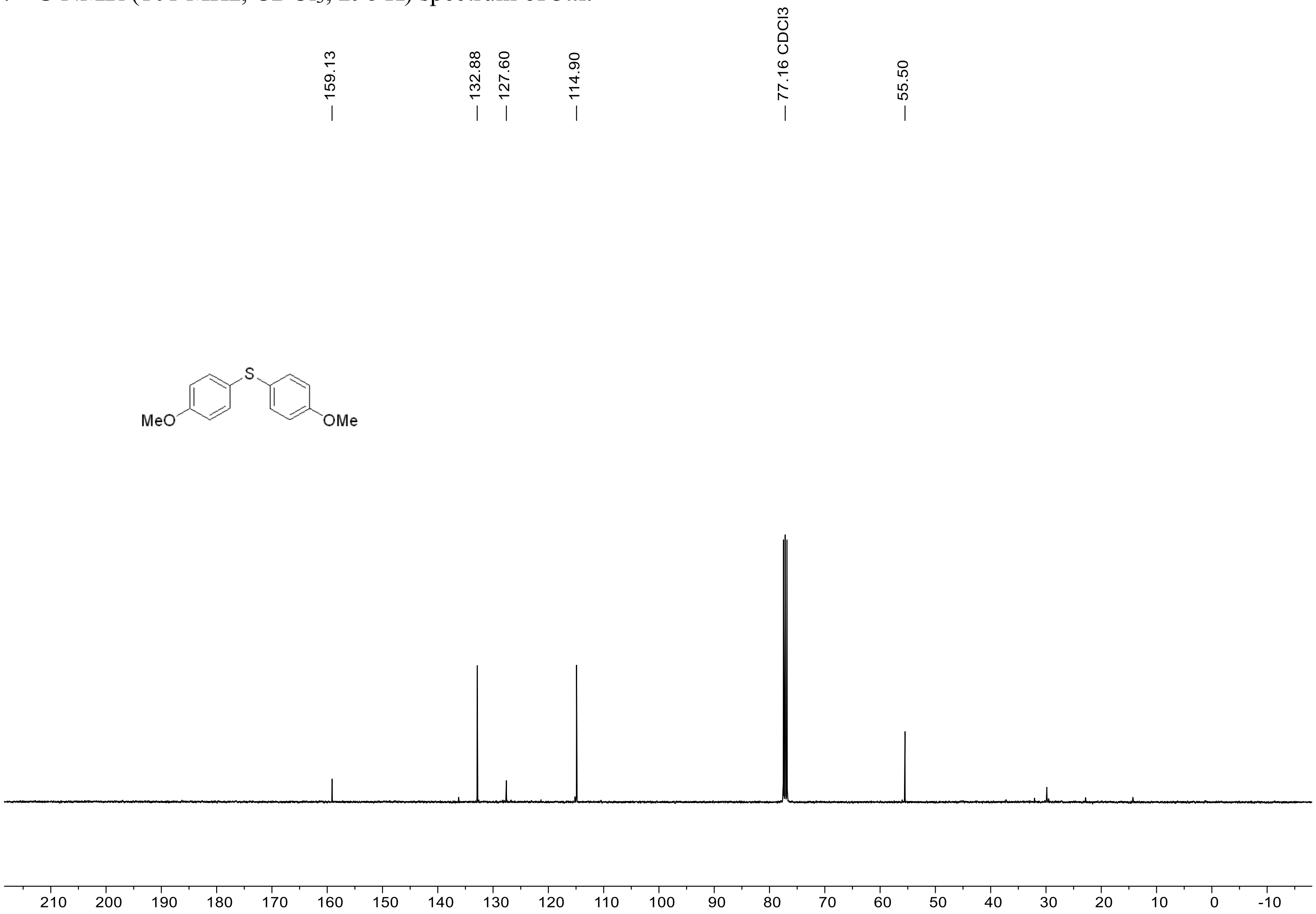

Figure S30: **^{1}H NMR** (400 MHz, $CDCl_3$, 298 K) spectrum of **3aj**.

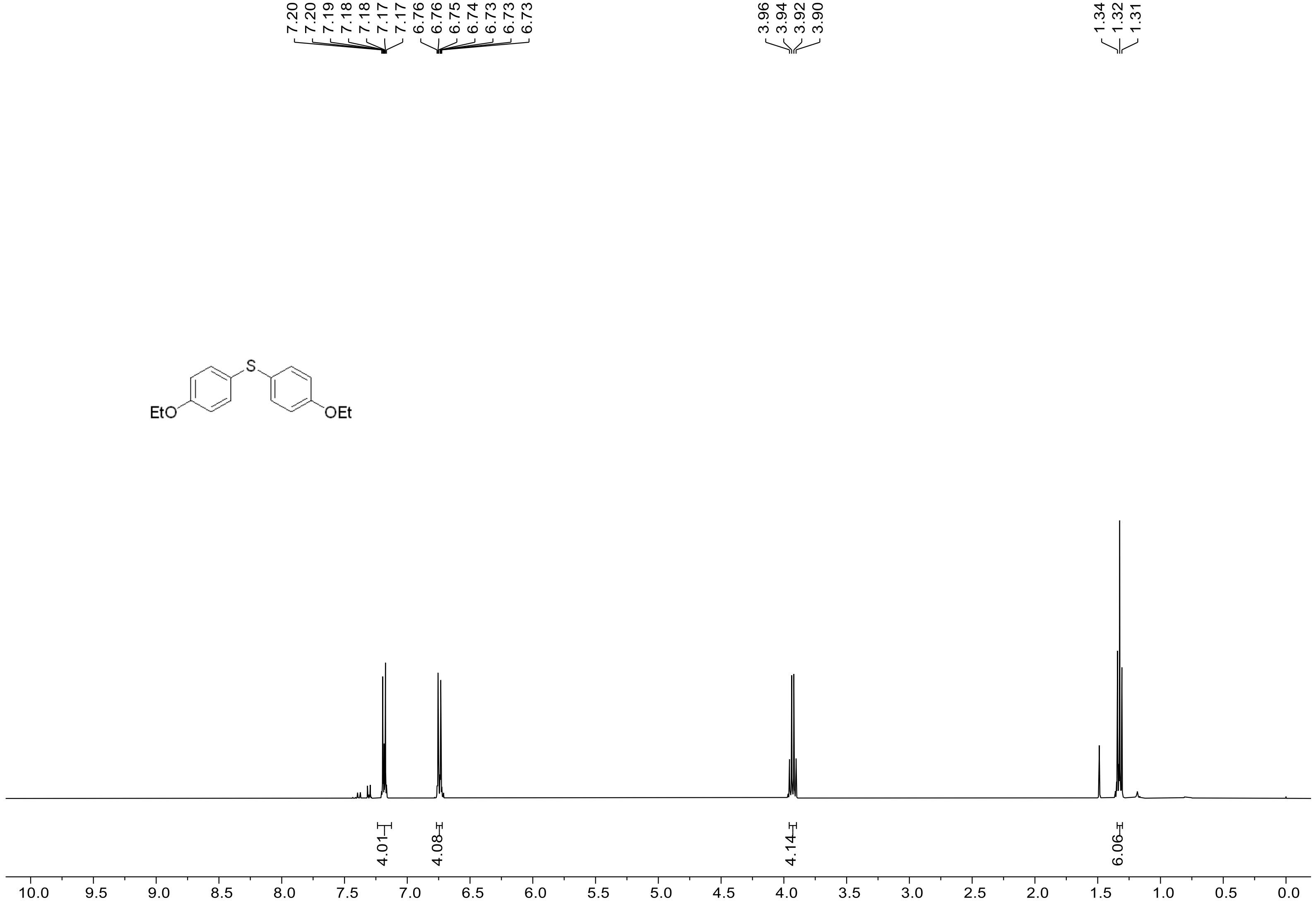

Figure S31: **^{13}C NMR** (101 MHz, $CDCl_3$, 298 K) spectrum of **3aj**.

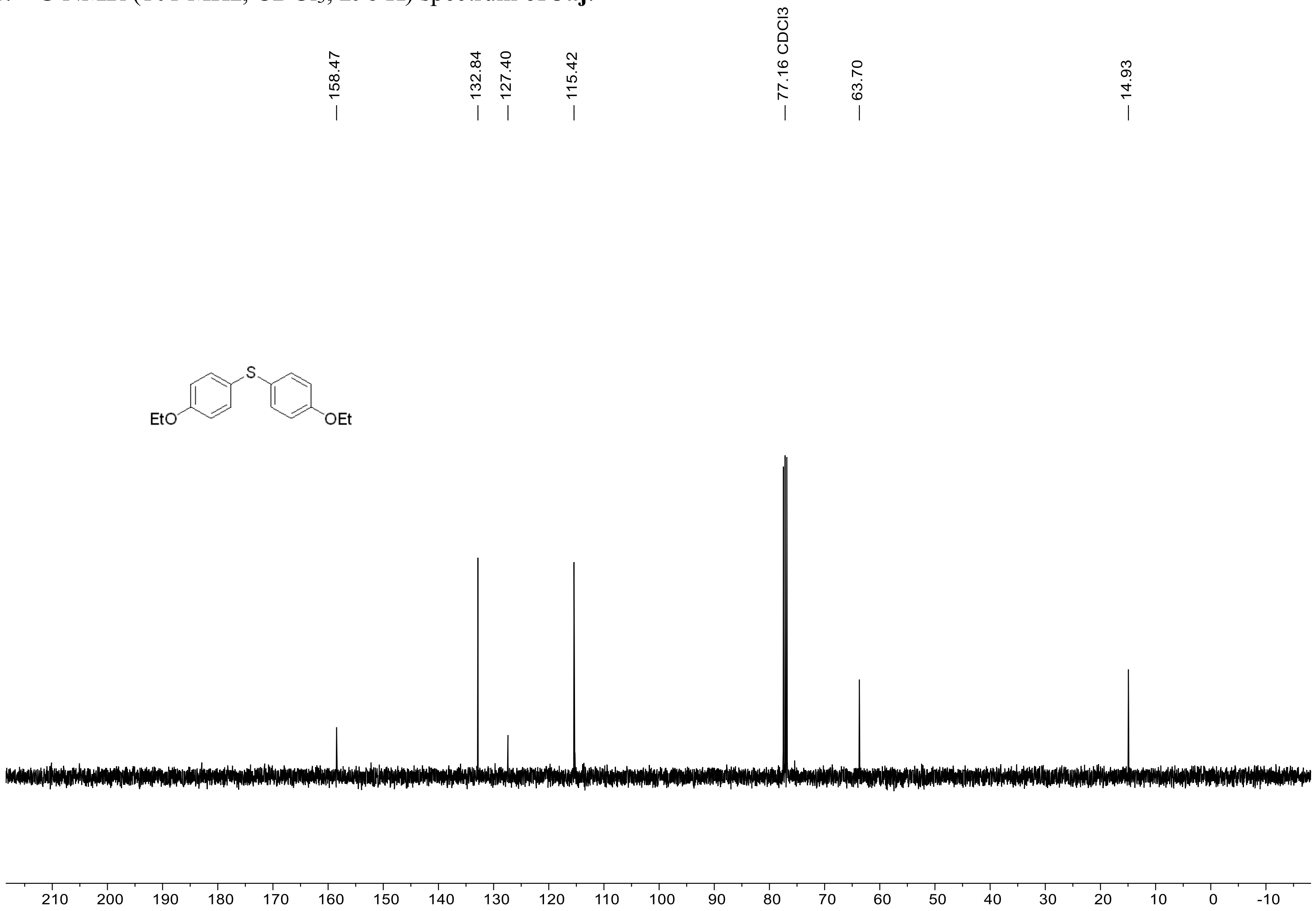

S32: ^{1}**H NMR** (400 MHz, $CDCl_3$, 298 K) spectrum of **3ak**.

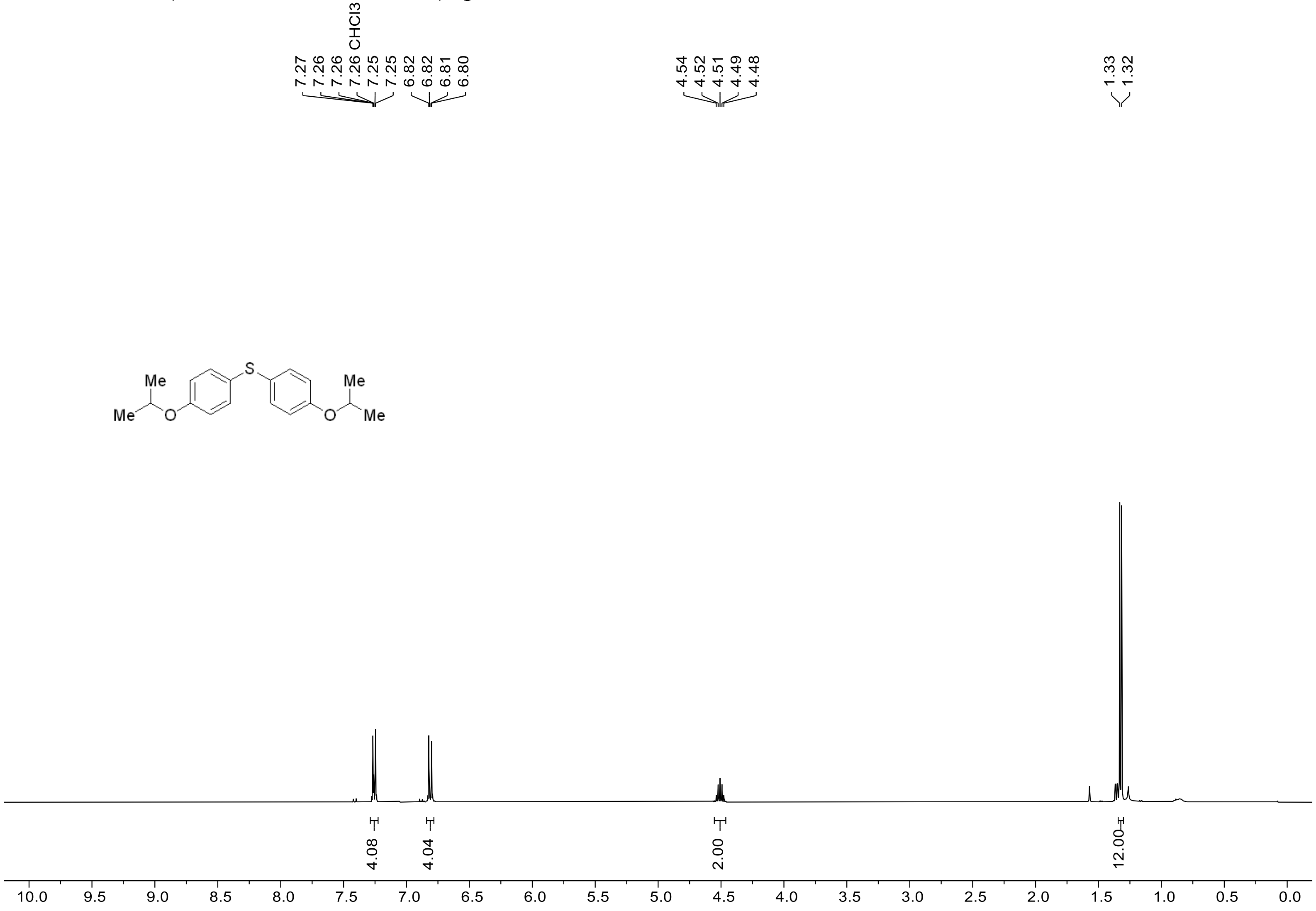

Figure S33: **^{13}C NMR** (101 MHz, $CDCl_3$, 298 K) spectrum of **3ak**.

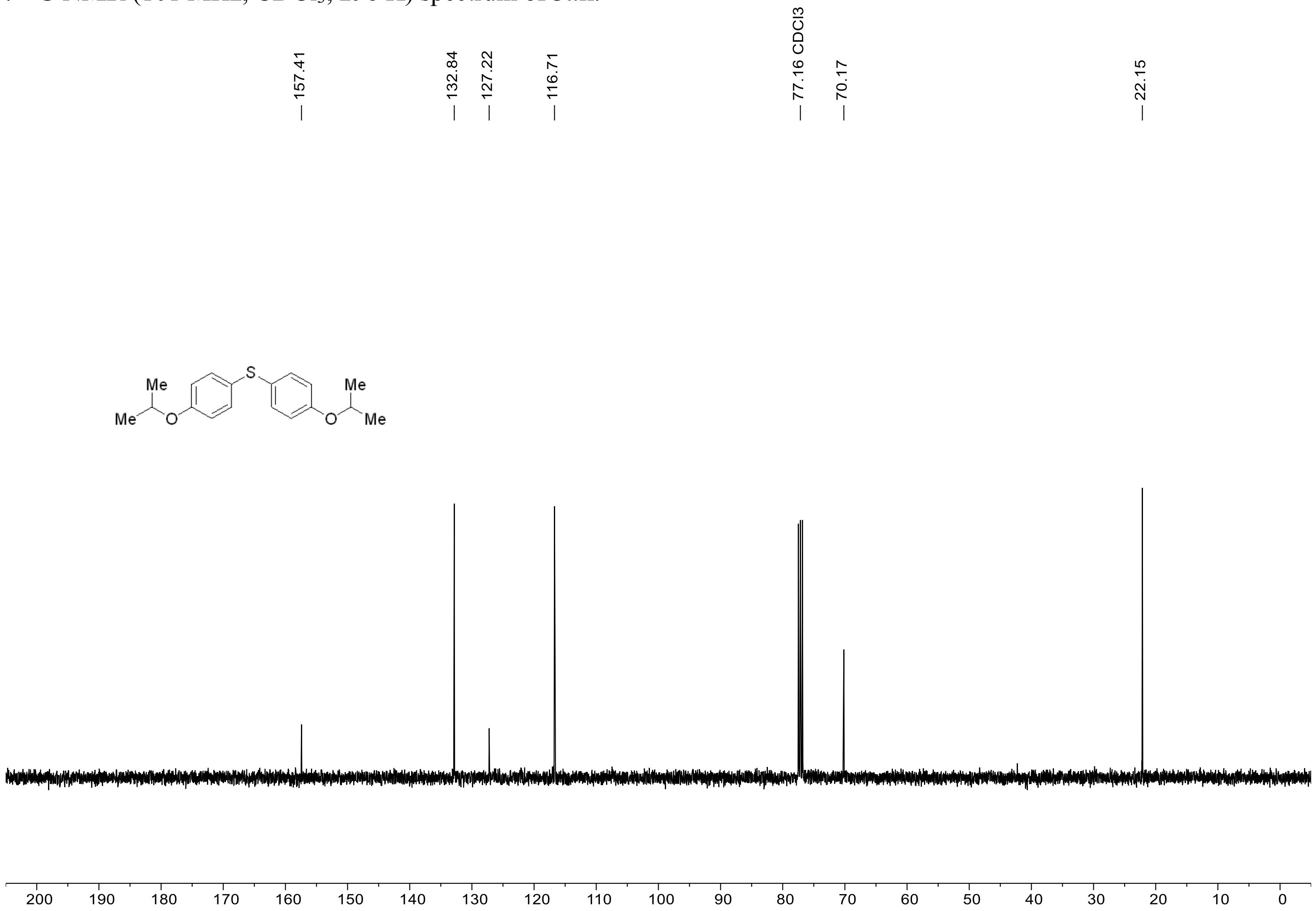

Figure S34: **^{1}H NMR** (400 MHz, $CDCl_3$, 298 K) spectrum of **3al**.

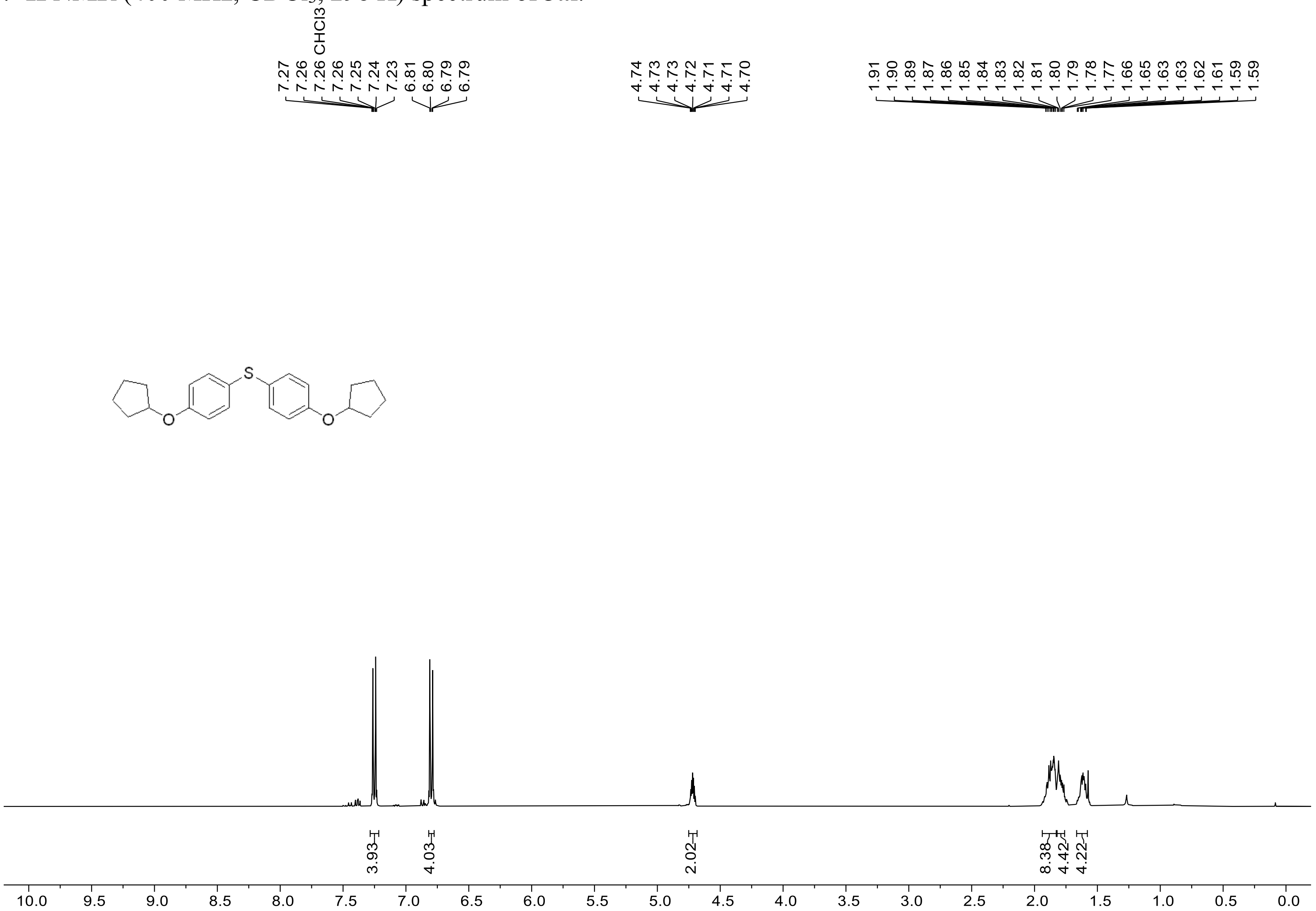

Figure S35: **^{13}C NMR** (101 MHz, $CDCl_3$, 298 K) spectrum of **3al**.

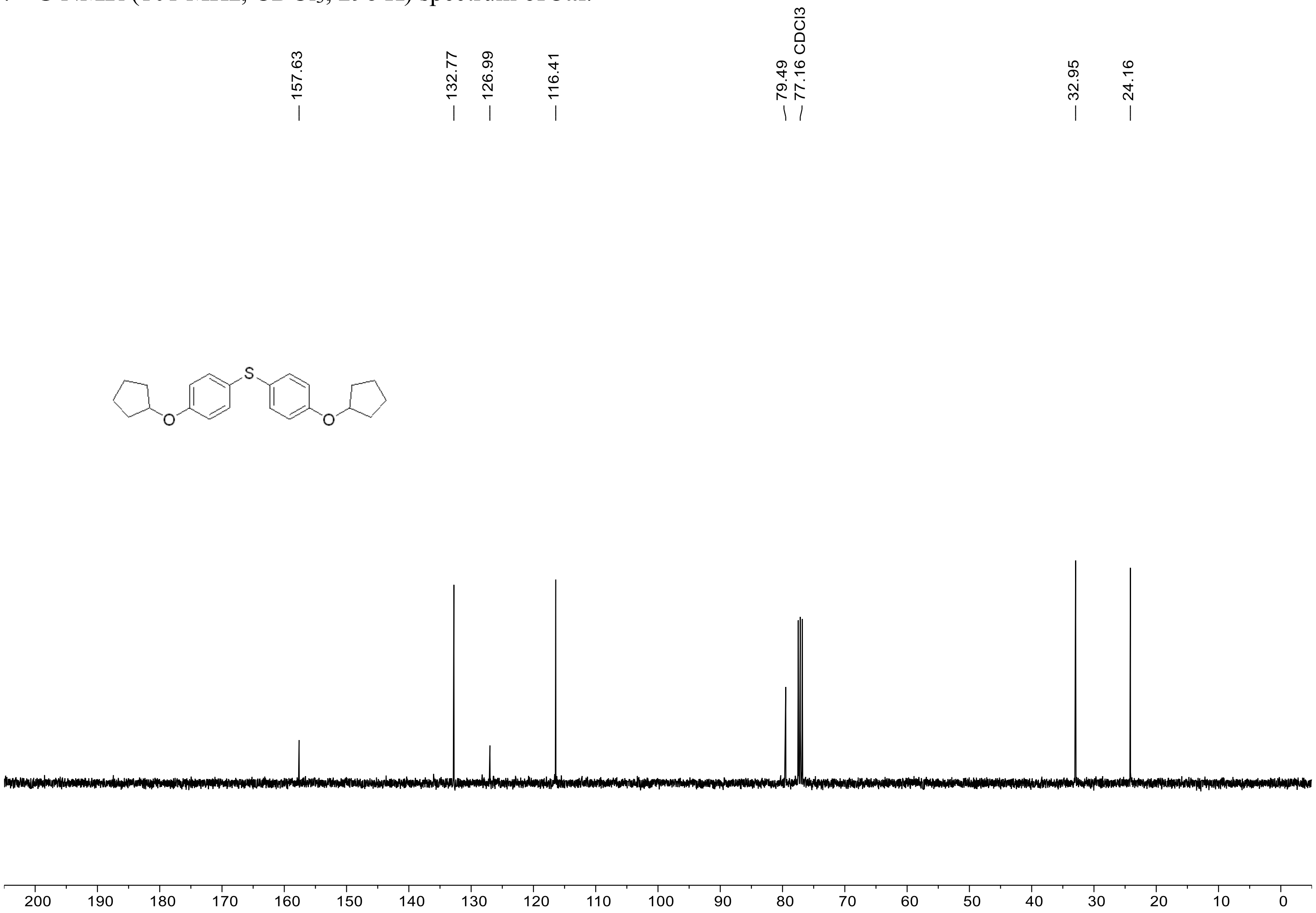

Figure S36: **^{1}H NMR** (400 MHz, $CDCl_3$, 298 K) spectrum of **3am**.

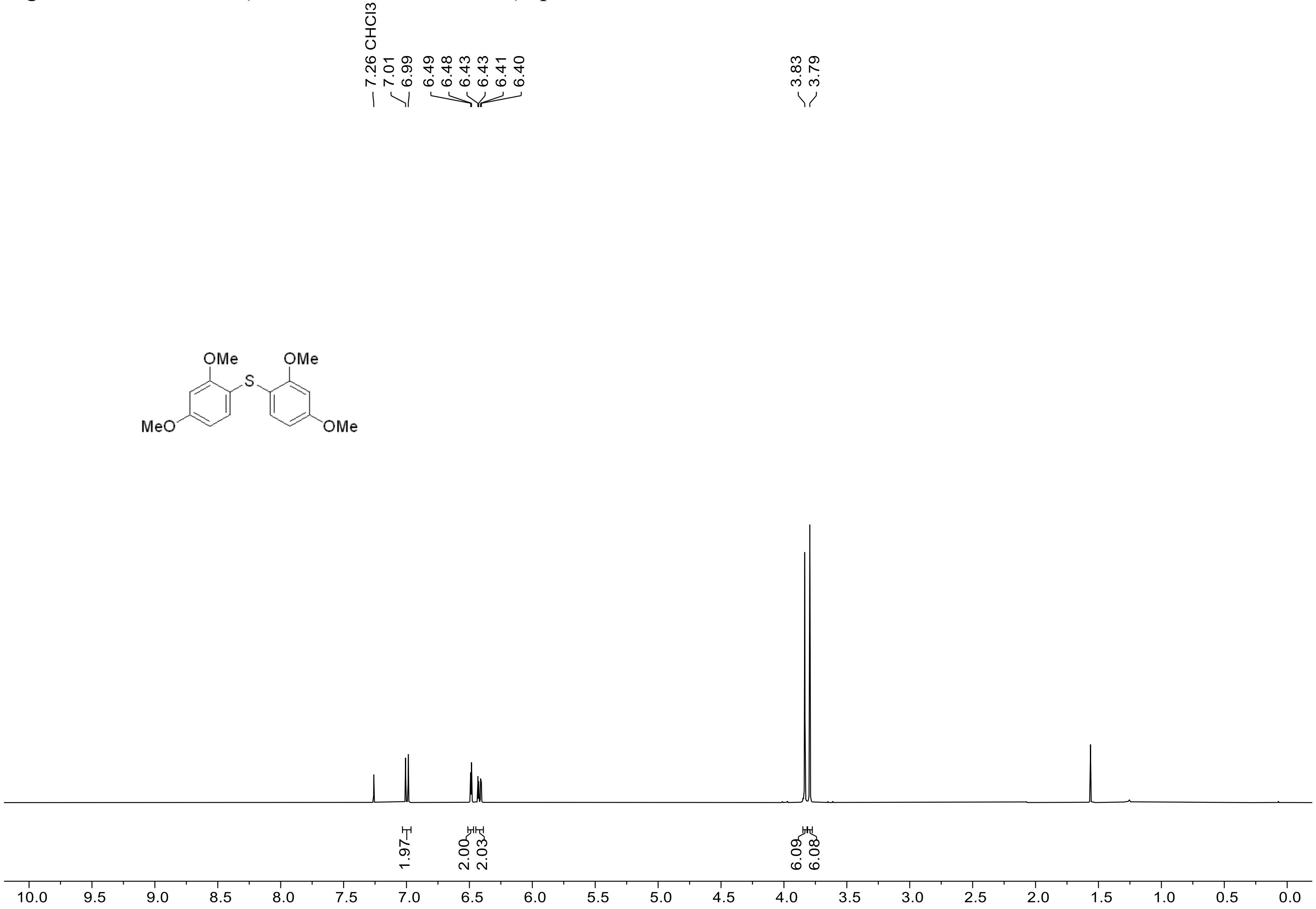

Figure S37: **^{13}C NMR** (101 MHz, $CDCl_3$, 298 K) spectrum of **3am**.

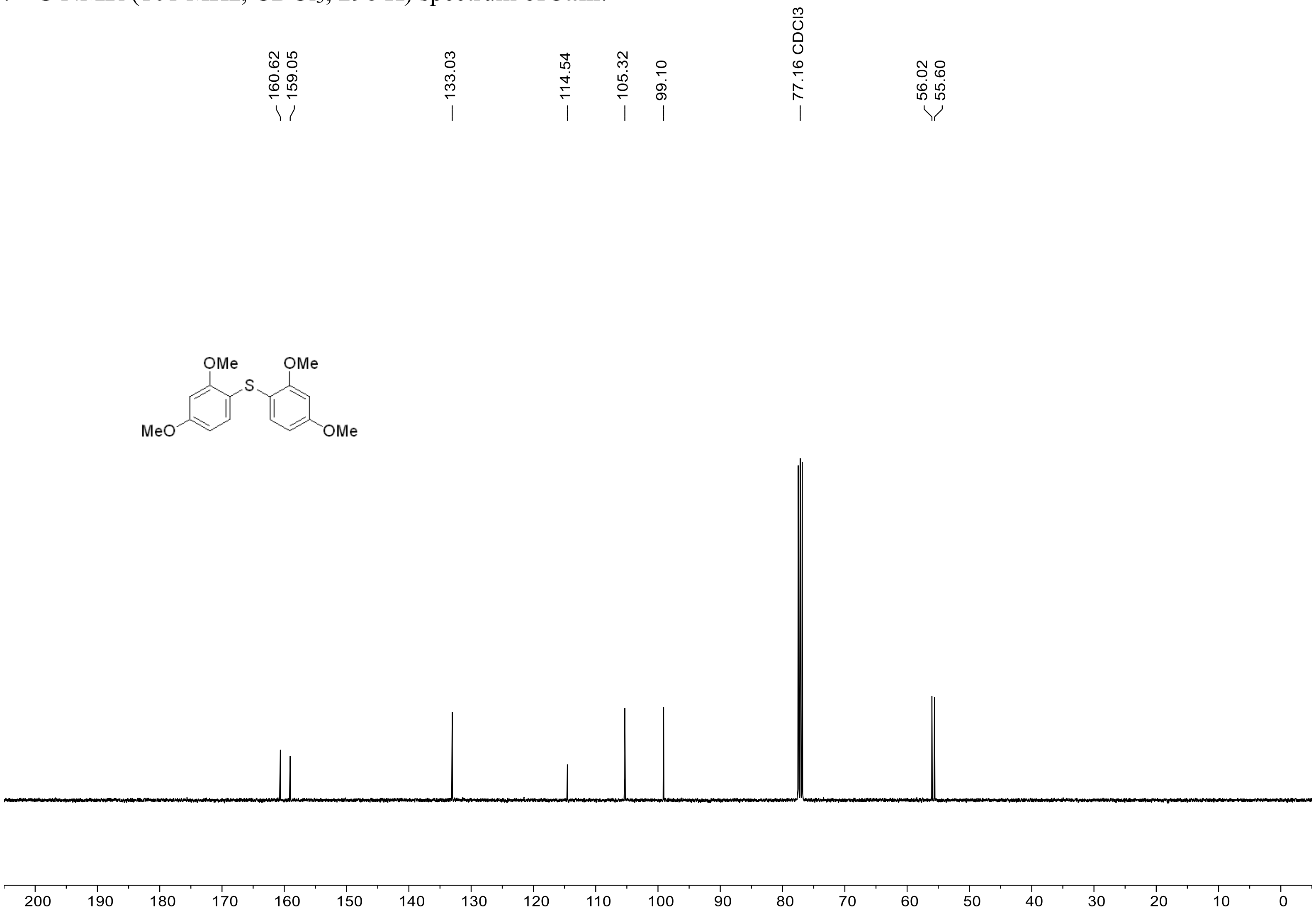

Figure S38: **^{1}H NMR** (400 MHz, $CDCl_3$, 298 K) spectrum of **3an**.

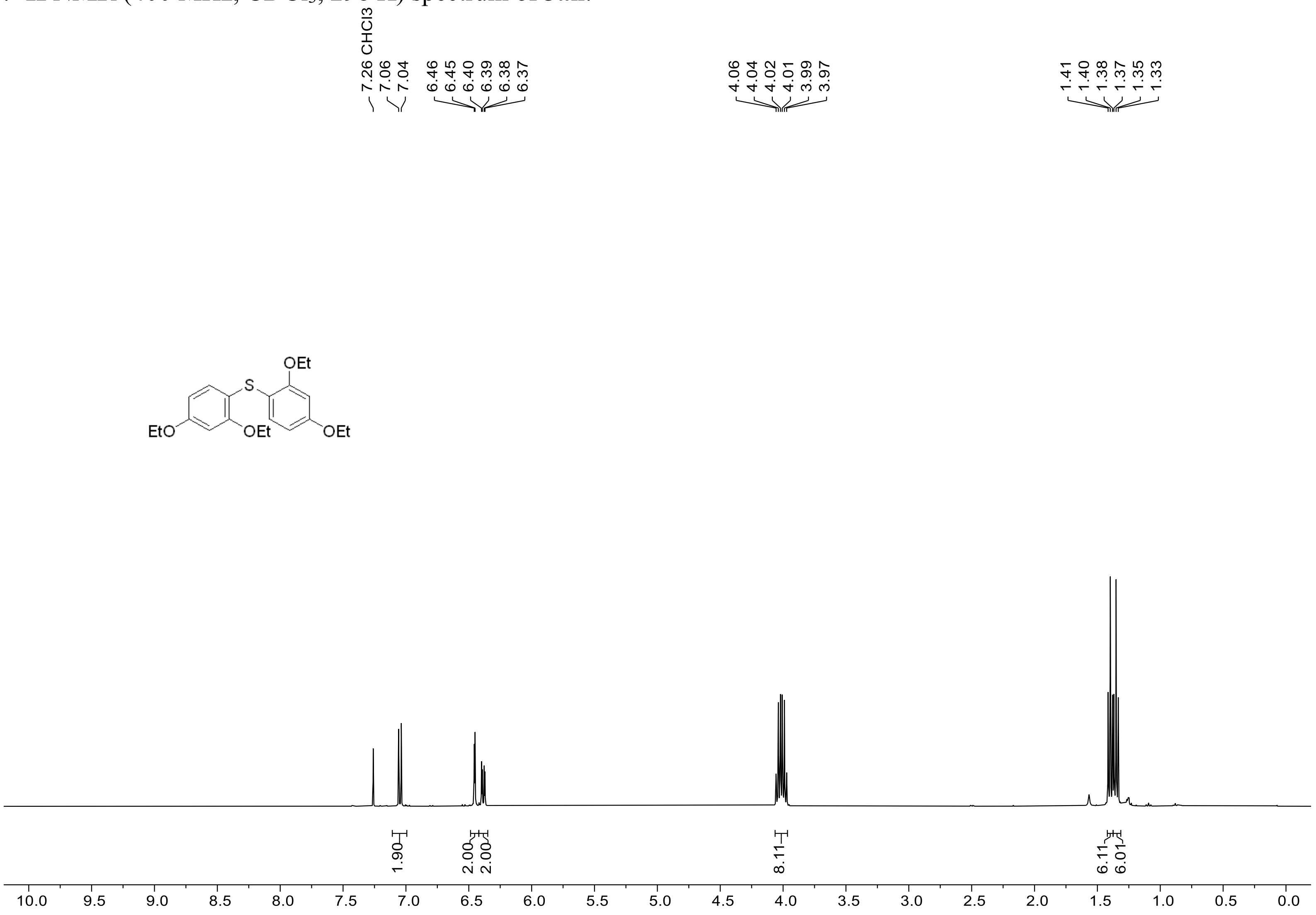

Figure S39: **^{13}C NMR** (101 MHz, $CDCl_3$, 298 K) spectrum of **3an**.

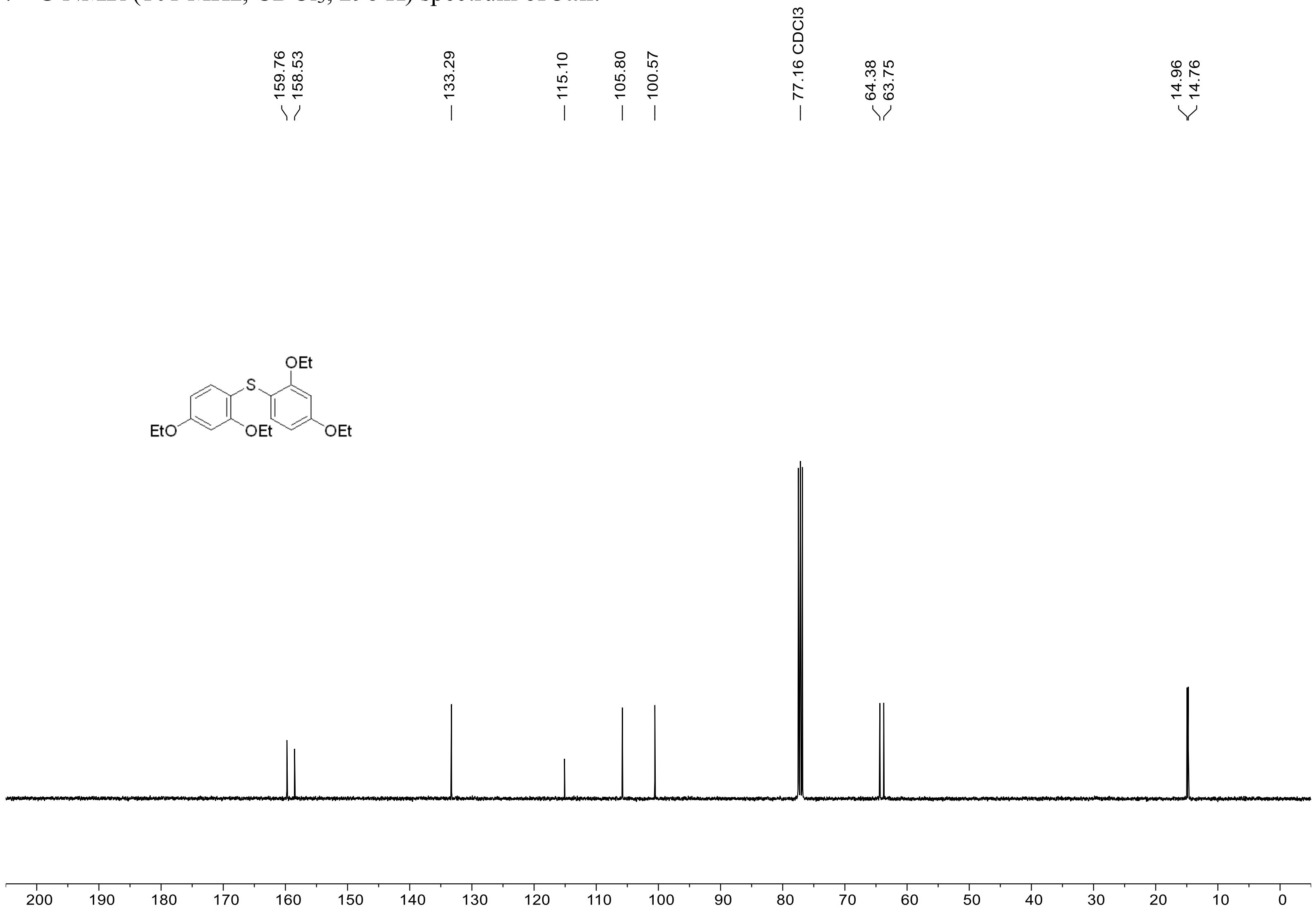

Figure S40: **^{1}H NMR** (400 MHz, $CDCl_3$, 298 K) spectrum of **3ao**.

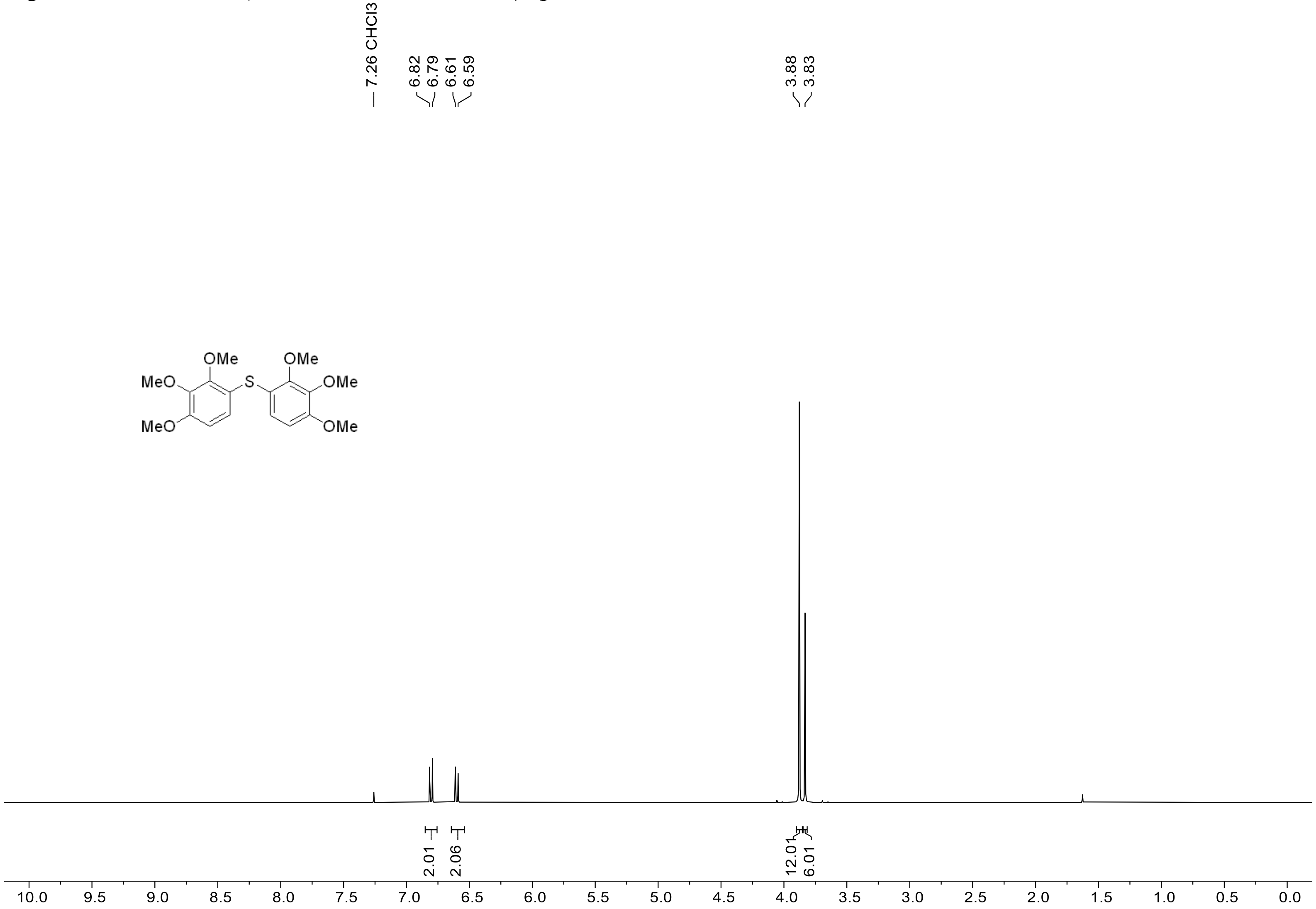

Figure S41: **^{13}C NMR** (101 MHz, $CDCl_3$, 298 K) spectrum of **3ao**.

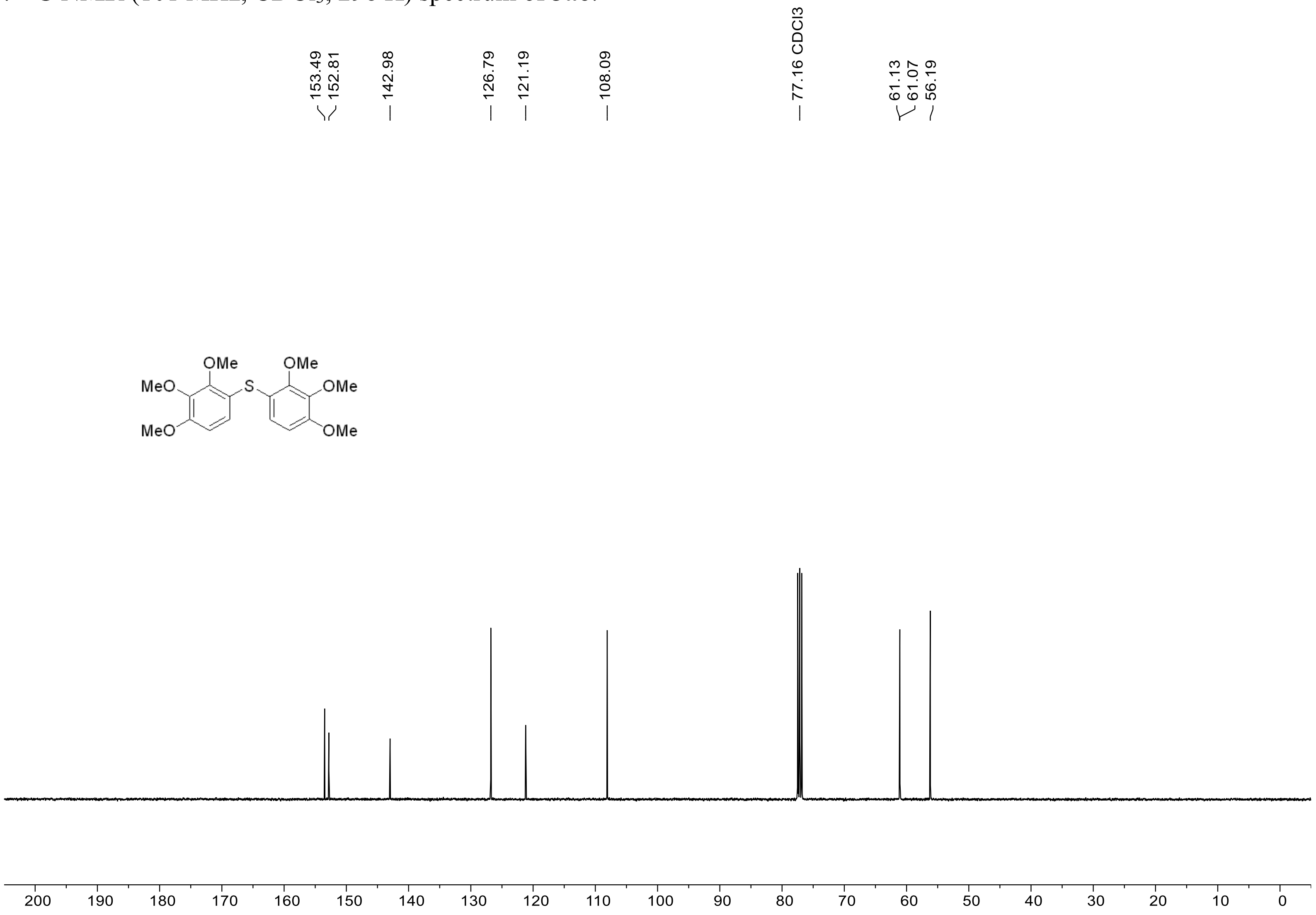

Figure S42: **^{1}H NMR** (400 MHz, $CDCl_3$, 298 K) spectrum of **3ap**.

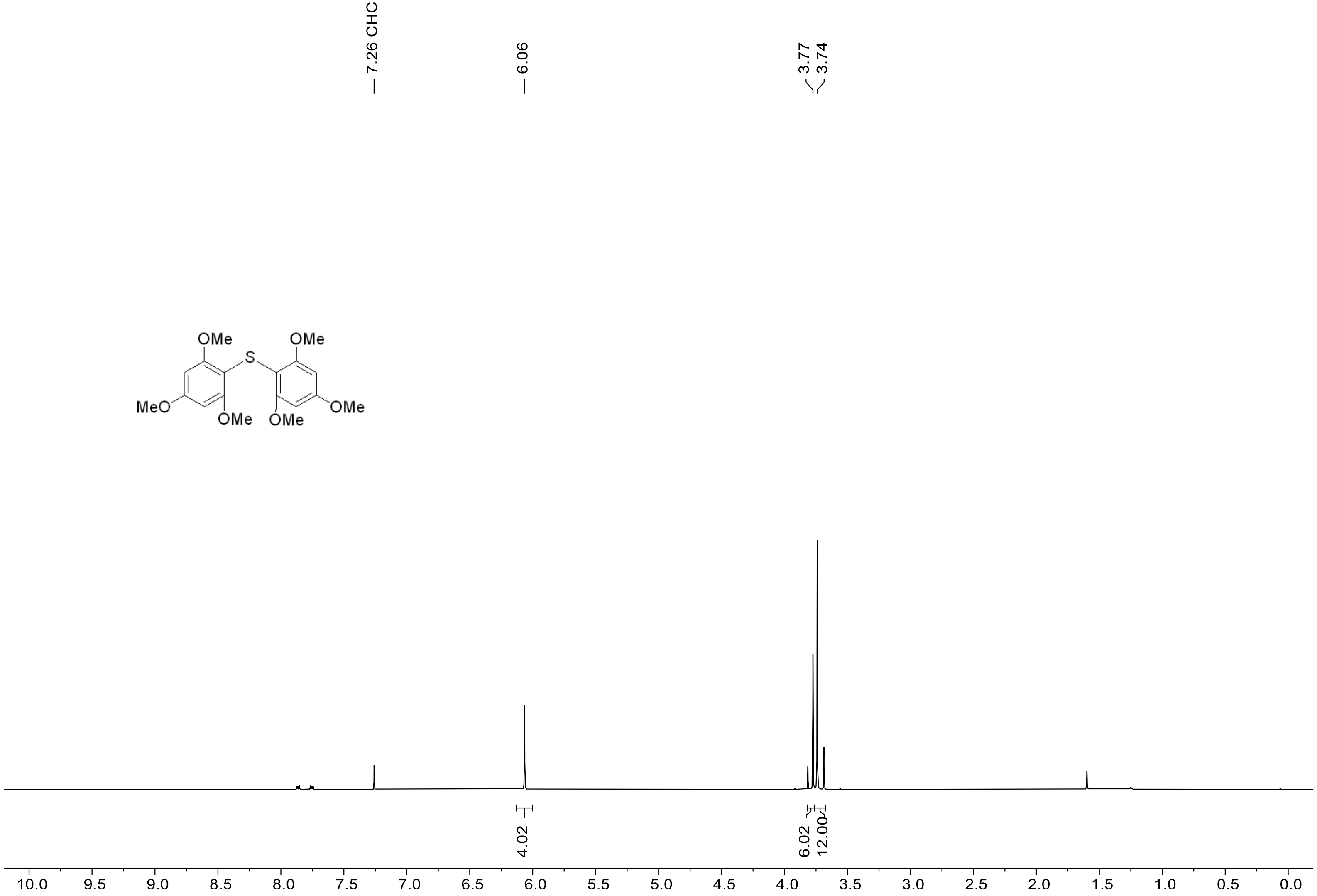

Figure S43: **^{13}C NMR** (101 MHz, $CDCl_3$, 298 K) spectrum of **3ap**.

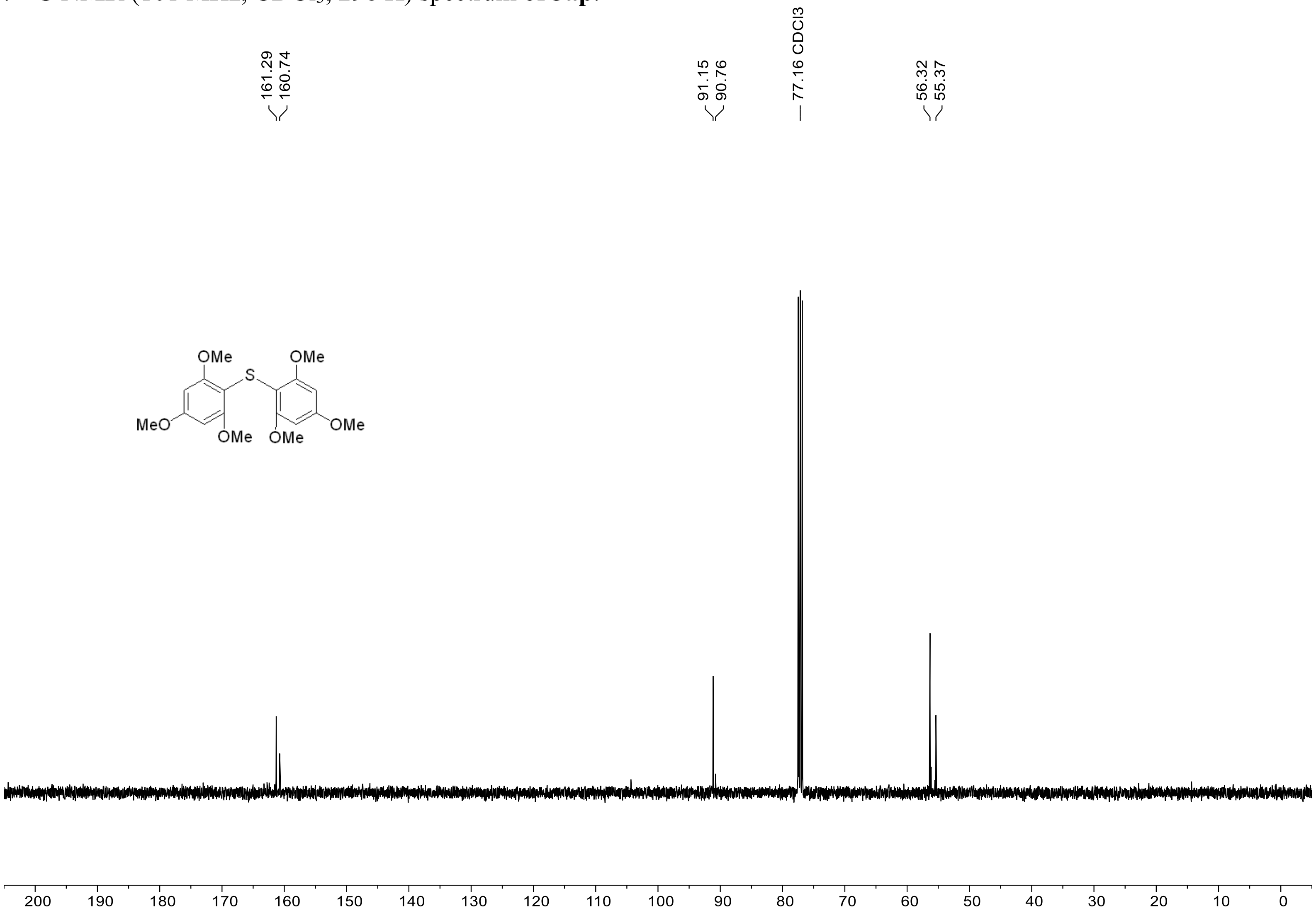

Figure S44: **^{1}H NMR** (400 MHz, $CDCl_3$, 298 K) spectrum of **3aq**.

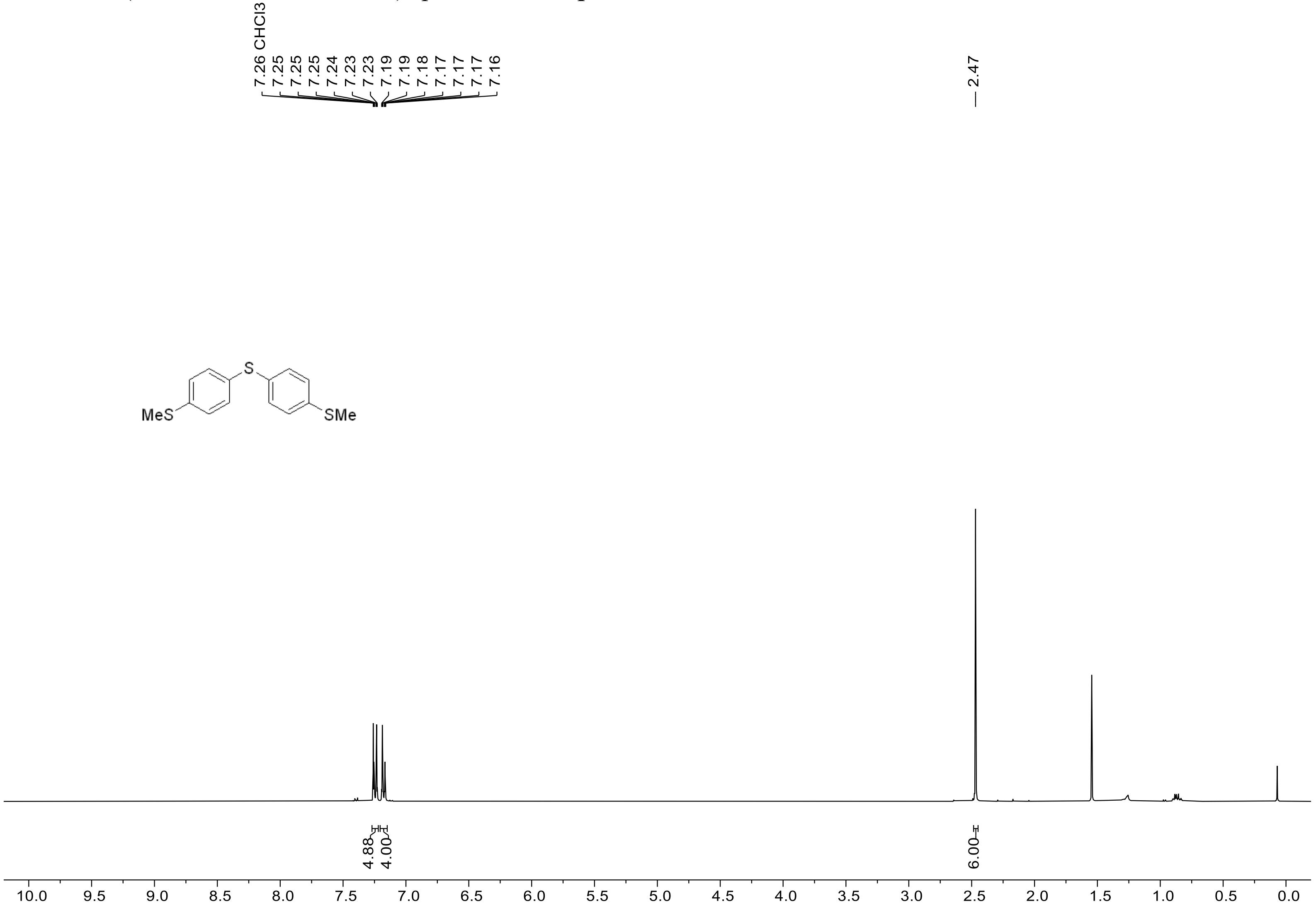

Figure S45: **^{13}C NMR** (101 MHz, $CDCl_3$, 298 K) spectrum of **3aq**.

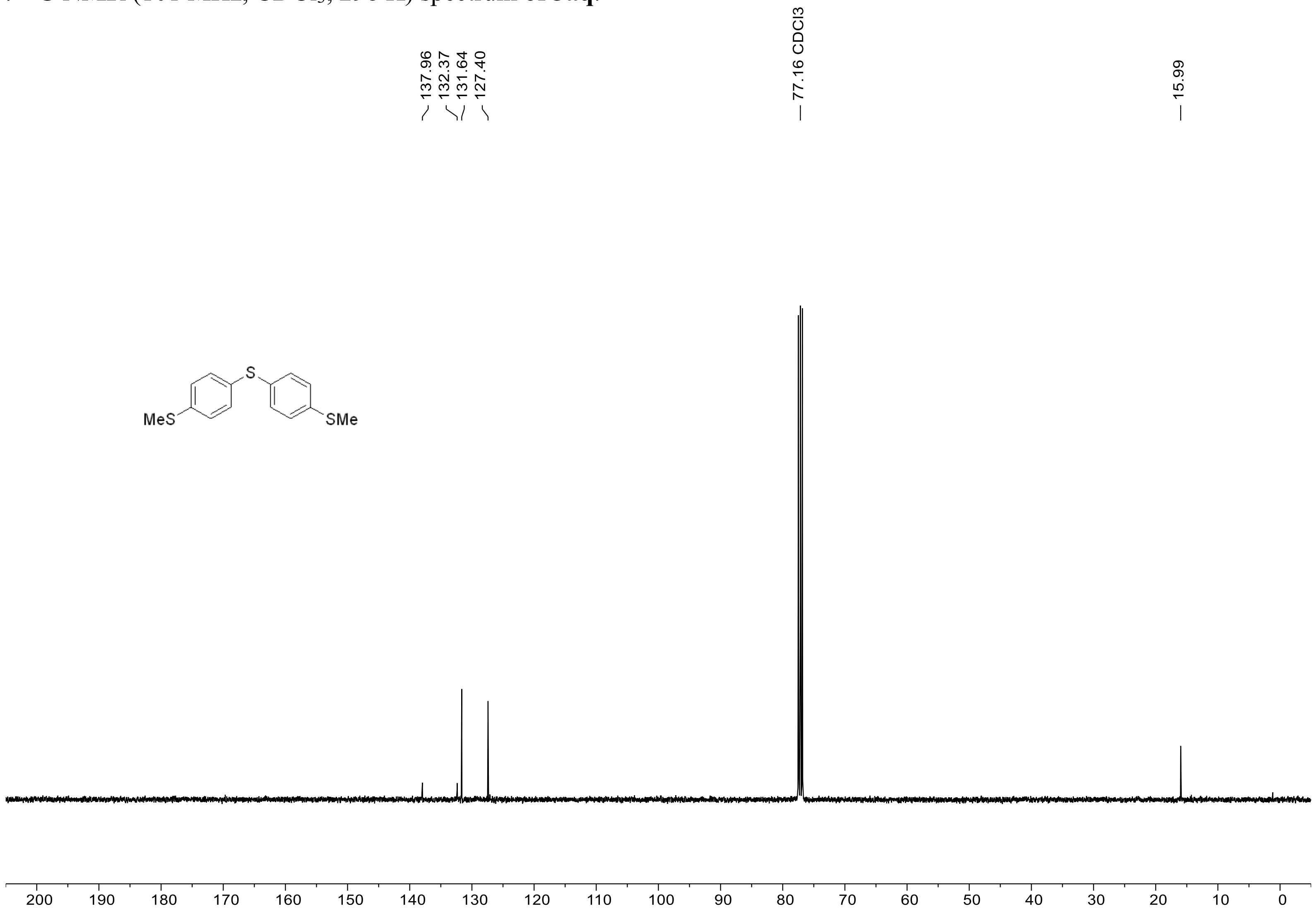

Figure S46: **^{1}H NMR** (400 MHz, $CDCl_3$, 298 K) spectrum of **3ar**.

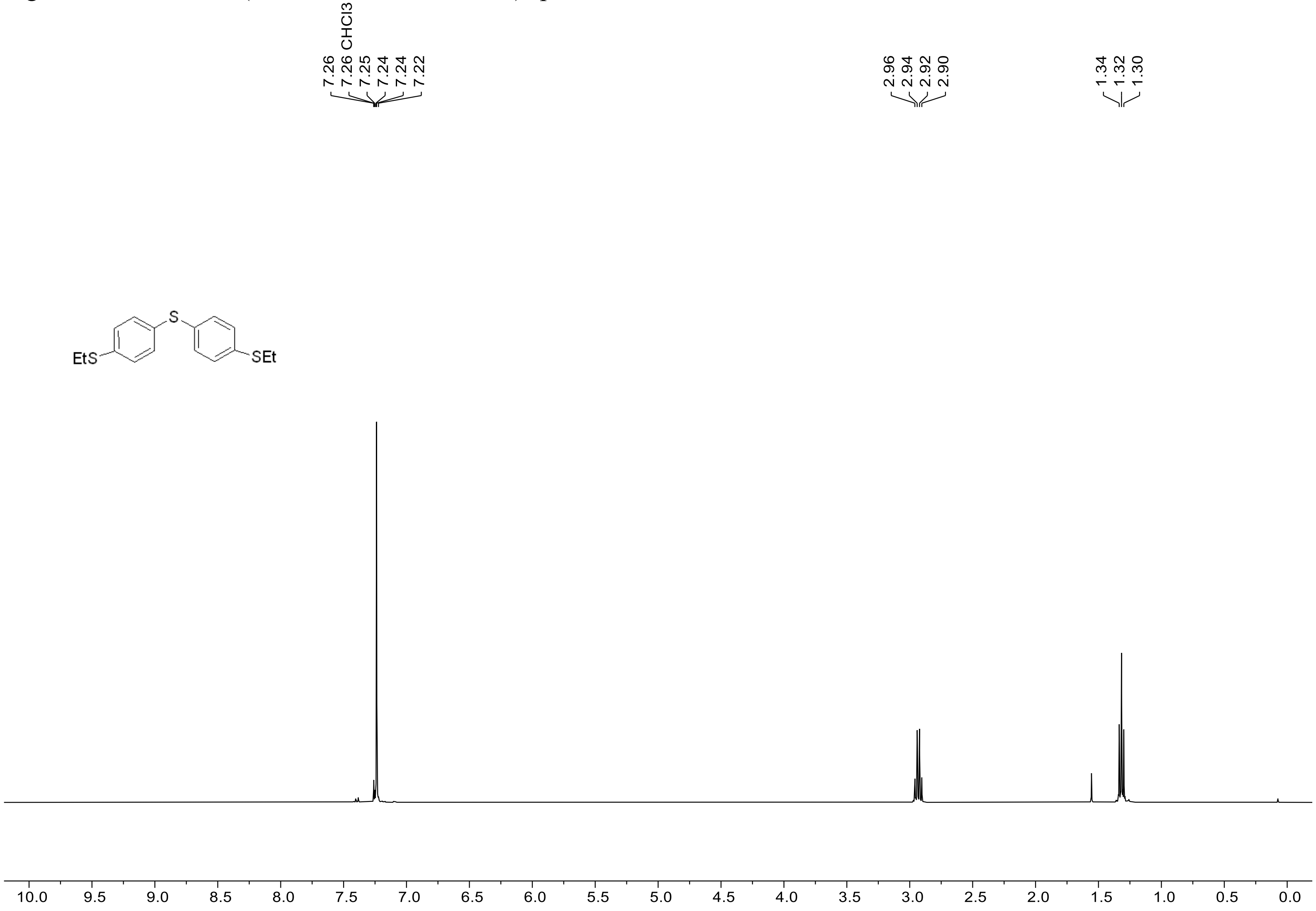

Figure S47: ^{13}**C NMR** (101 MHz, $CDCl_3$, 298 K) spectrum of **3ar**.

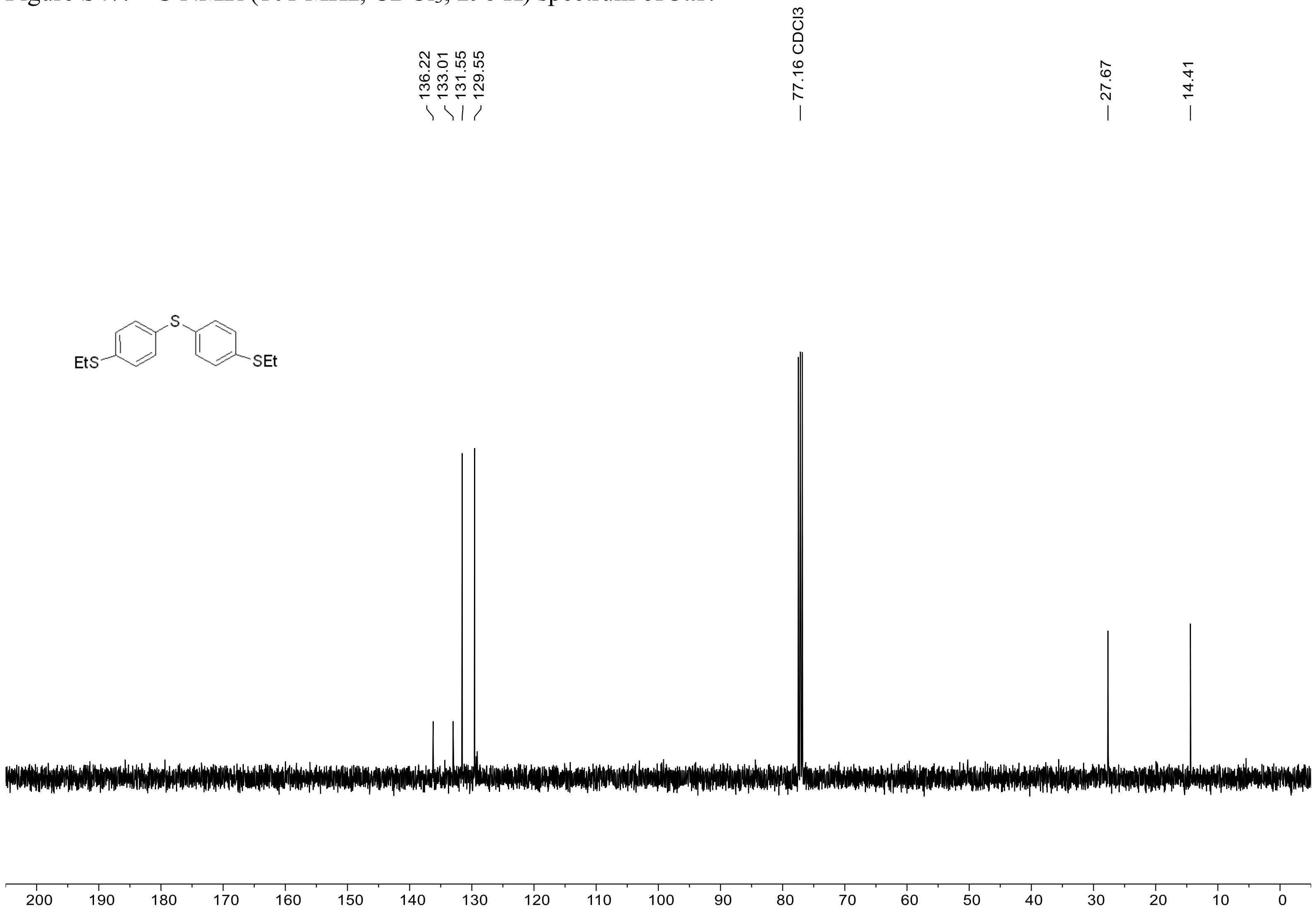

Figure S48: **¹H NMR** (400 MHz, $CDCl_3$, 298 K) spectrum of **3as**.

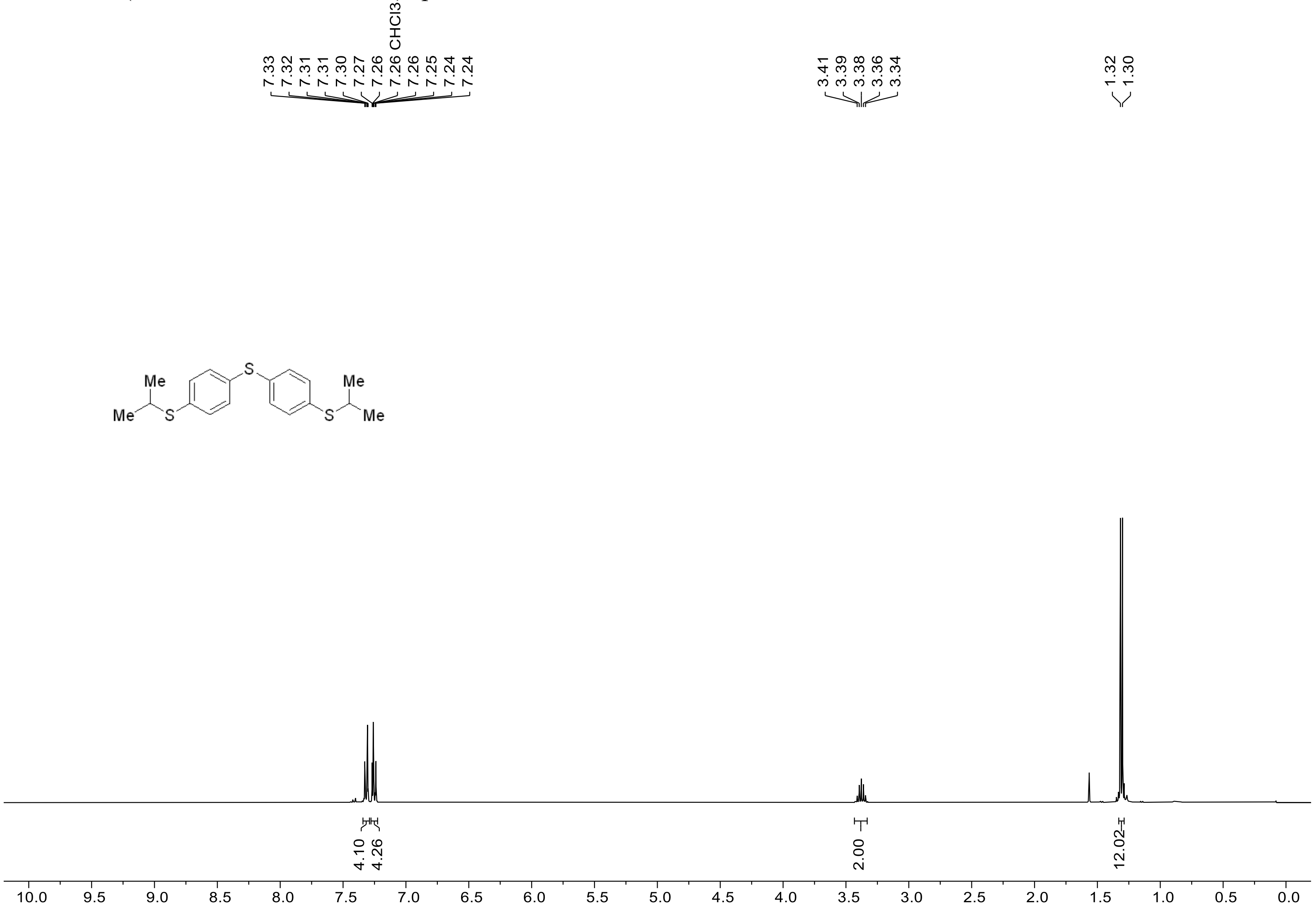

Figure S49: ^{13}**C NMR** (101 MHz, $CDCl_3$, 298 K) spectrum of **3as**.

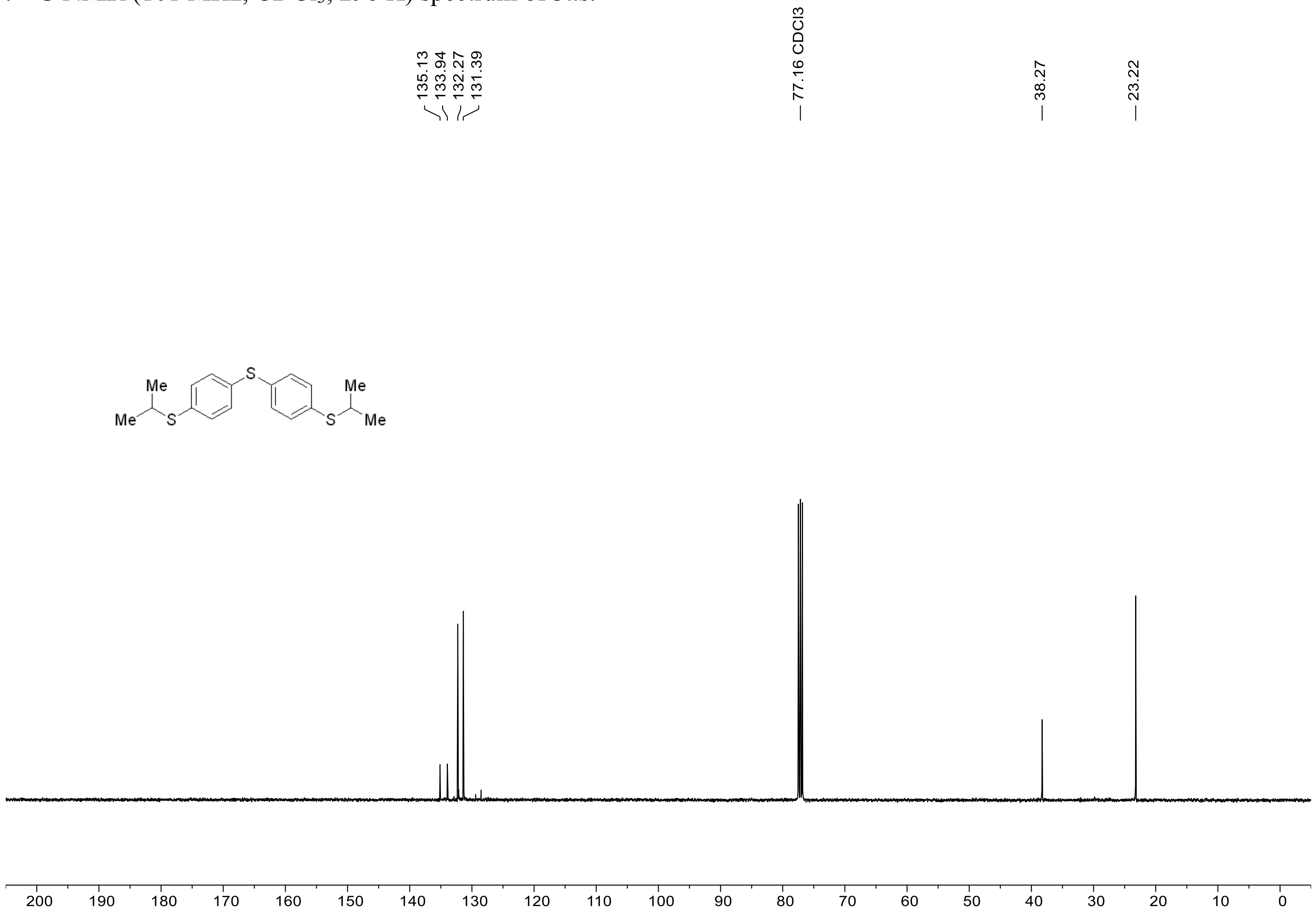

Figure S50: **^{1}H NMR** (400 MHz, $CDCl_3$, 298 K) spectrum of **3at**.

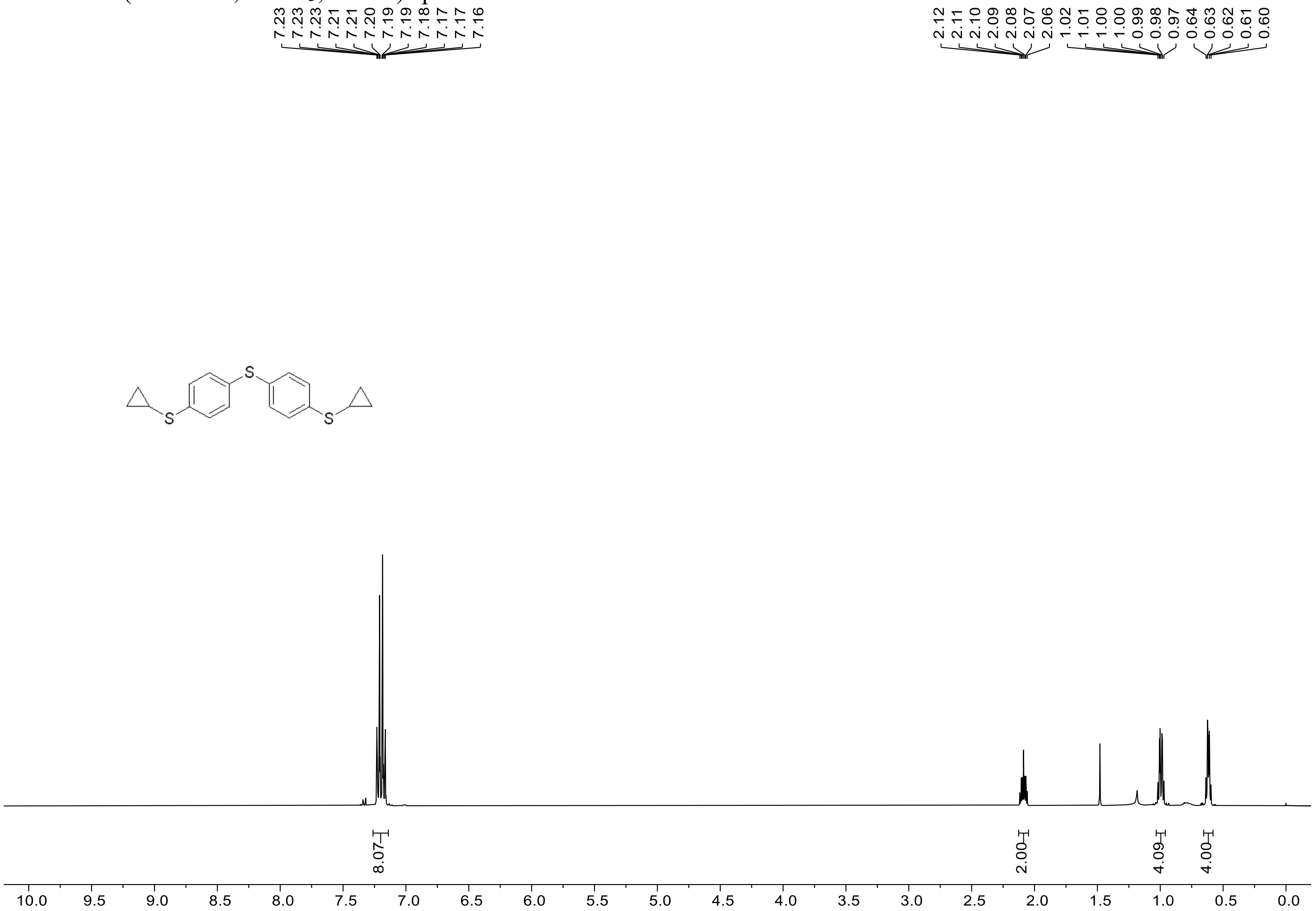

Figure S51: **^{13}C NMR** (101 MHz, $CDCl_3$, 298 K) spectrum of **3at**.

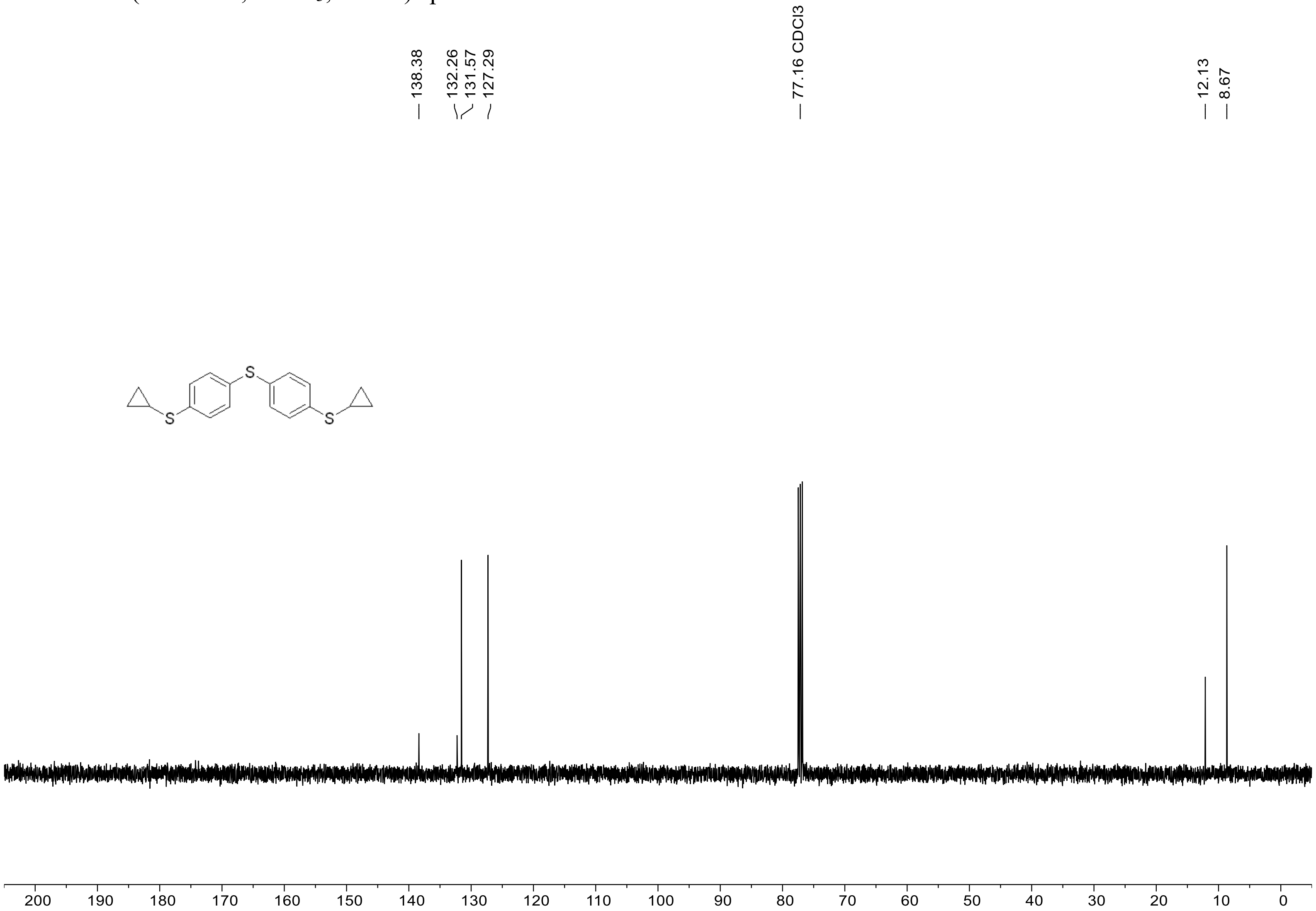

Figure S52: **^{1}H NMR** (400 MHz, $CDCl_3$, 298 K) spectrum of **3au**.

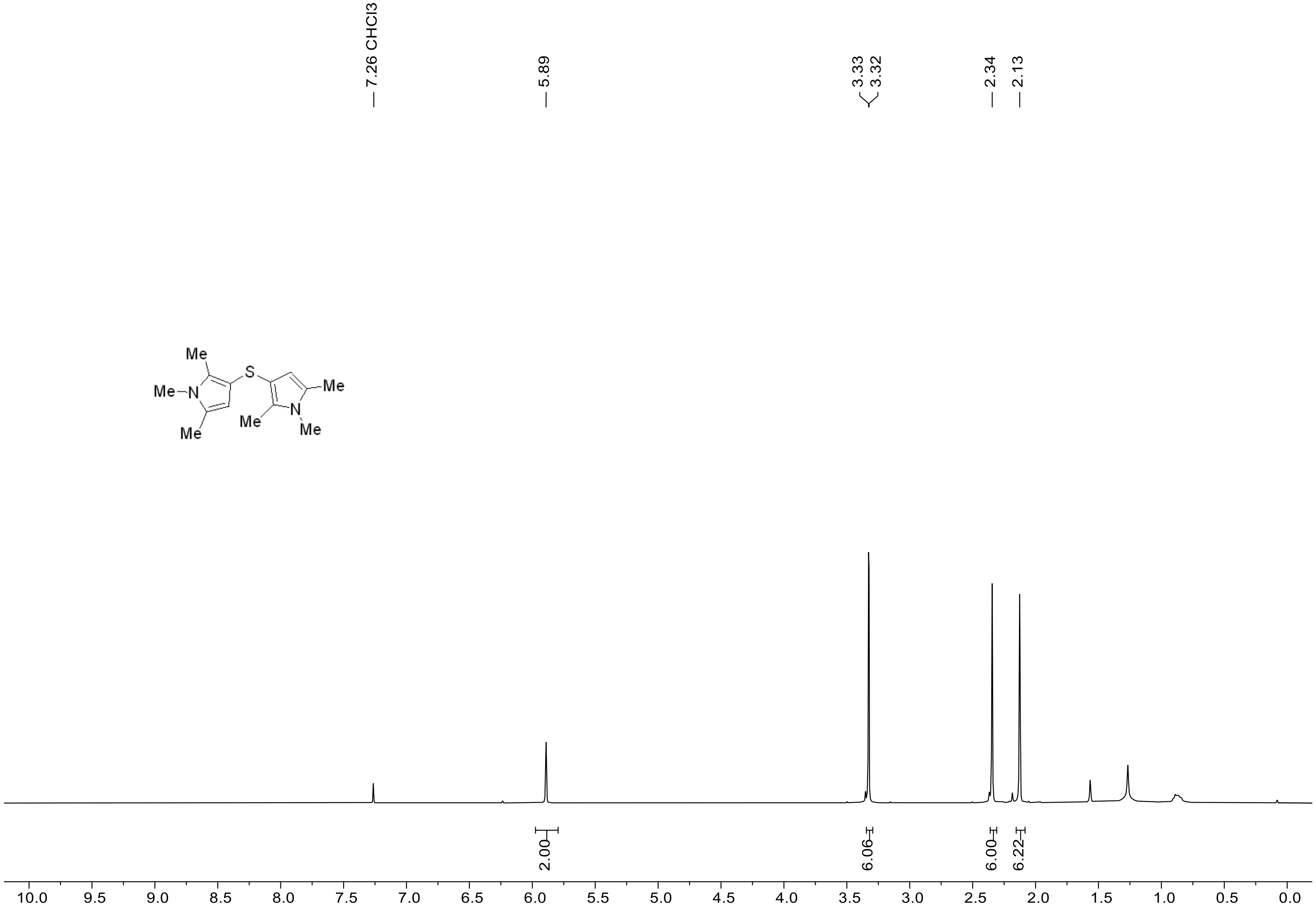

Figure S53: **^{13}C NMR** (101 MHz, $CDCl_3$, 298 K) spectrum of **3au**.

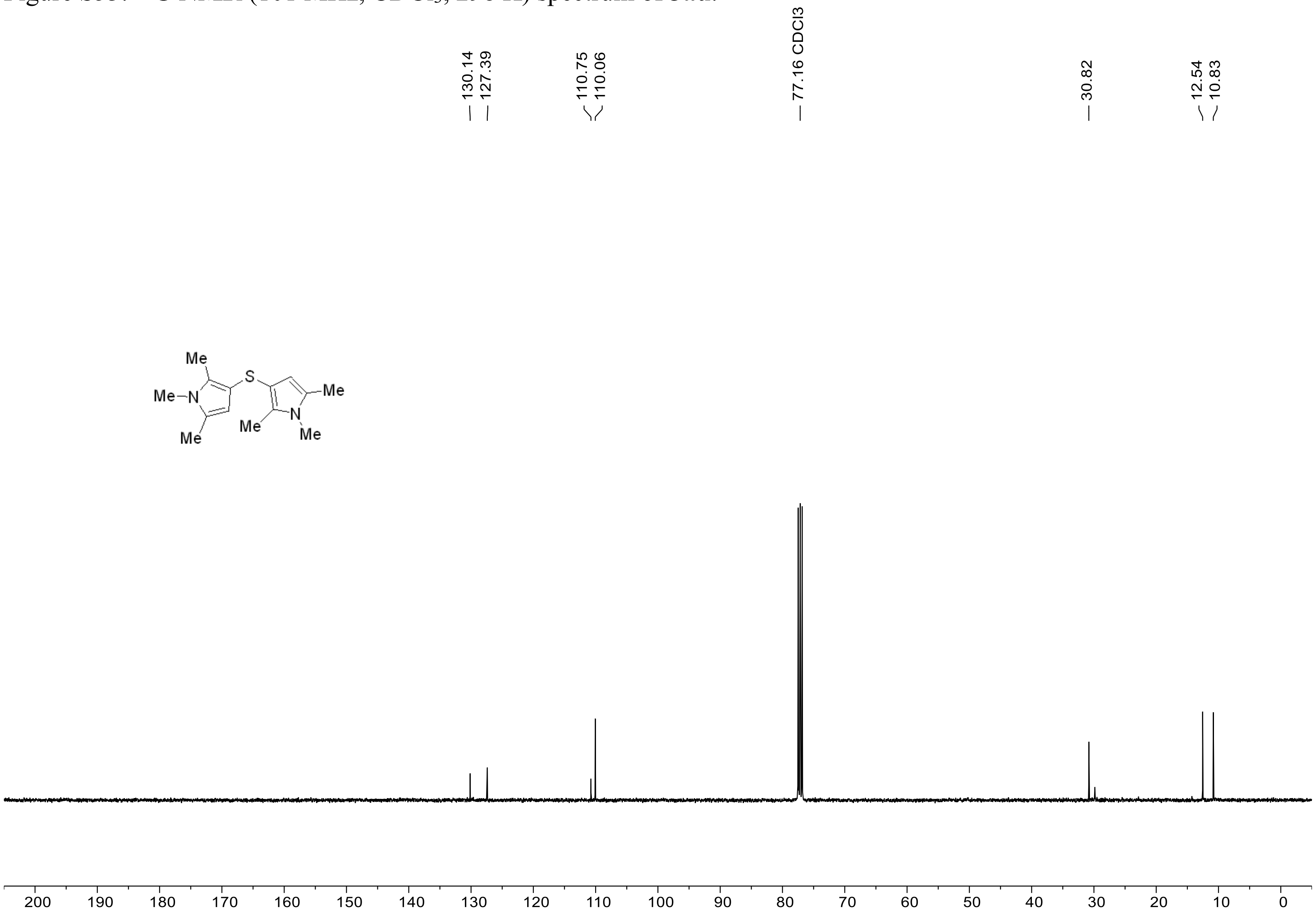

Figure S54: **^{1}H NMR** (400 MHz, $CDCl_3$, 298 K) spectrum of **3av**.

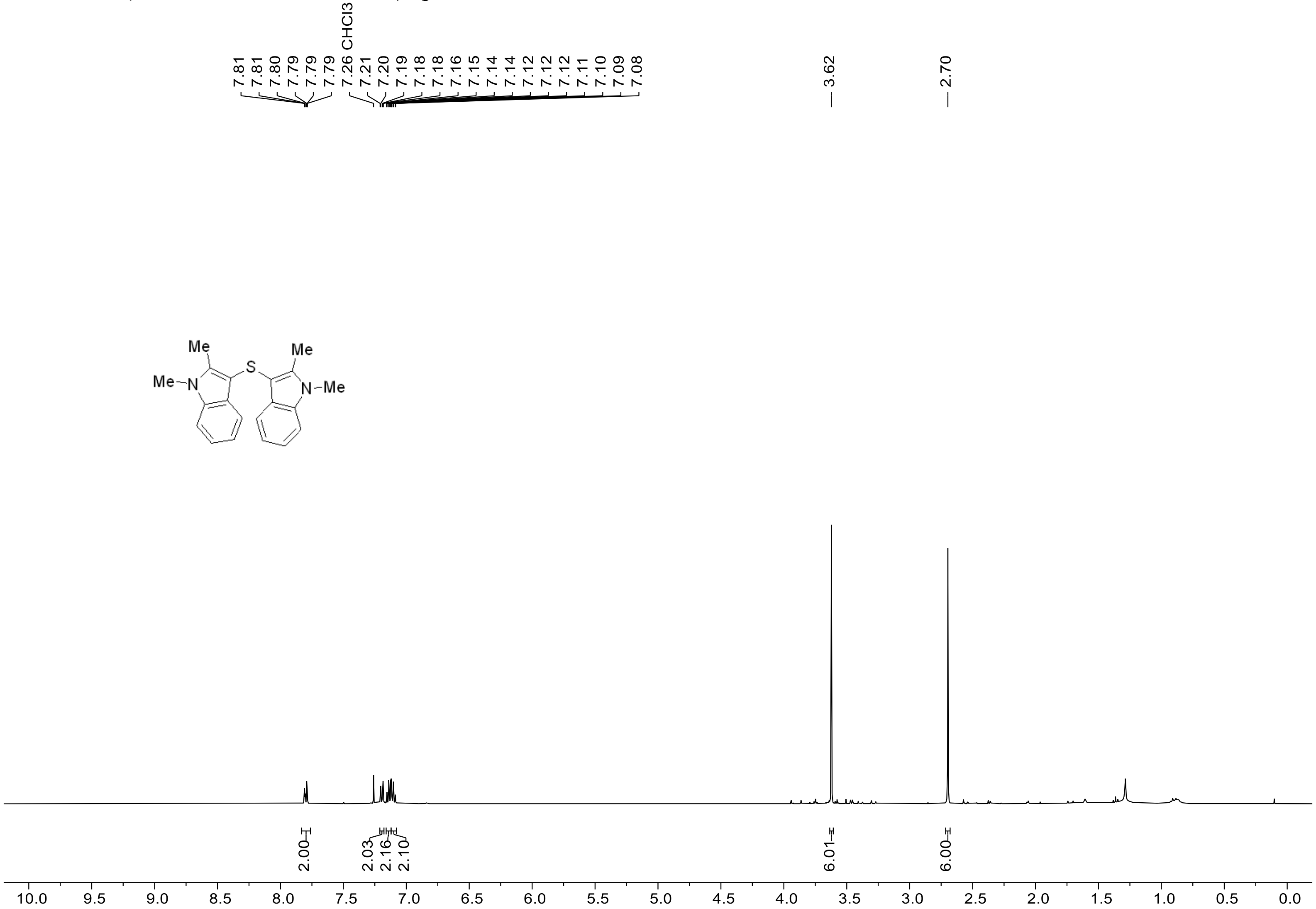

Figure S55: ^{13}**C NMR** (101 MHz, $CDCl_3$, 298 K) spectrum of **3av**.

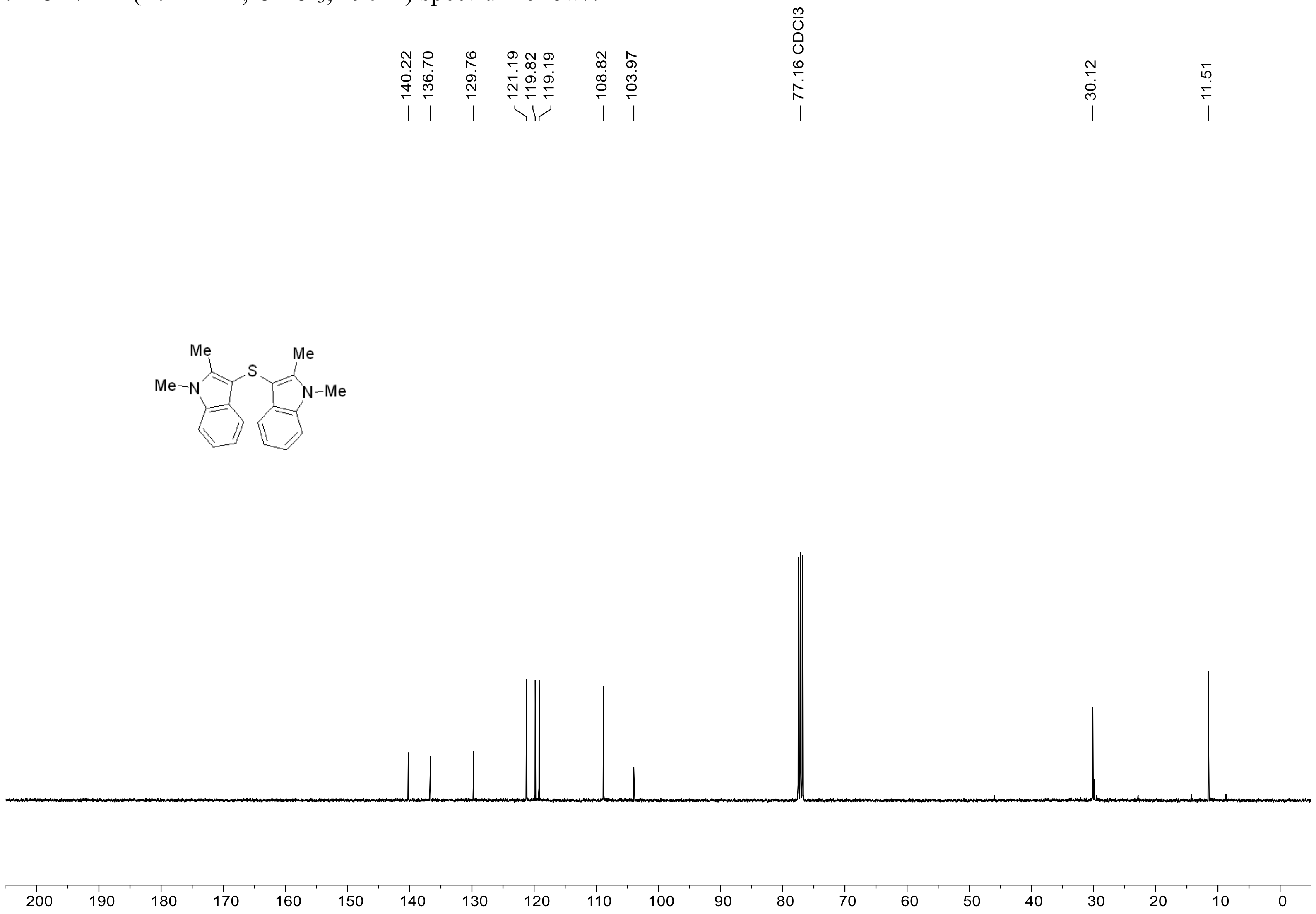

Figure S56: ^{1}**H NMR** (400 MHz, $CDCl_3$, 298 K) spectrum of **3aw**.

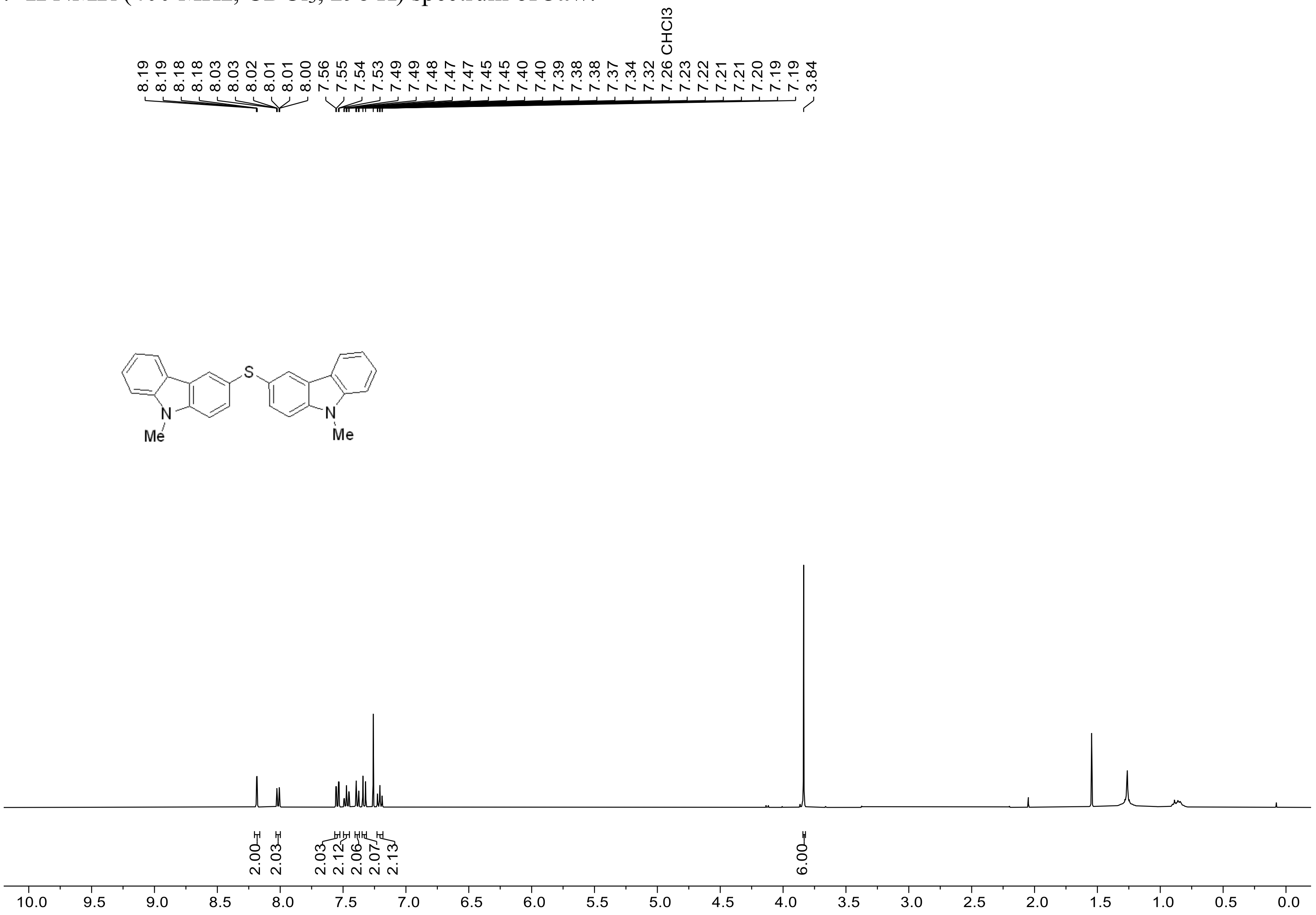

Figure S57: ^{13}**C NMR** (101 MHz, $CDCl_3$, 298 K) spectrum of **3aw**.

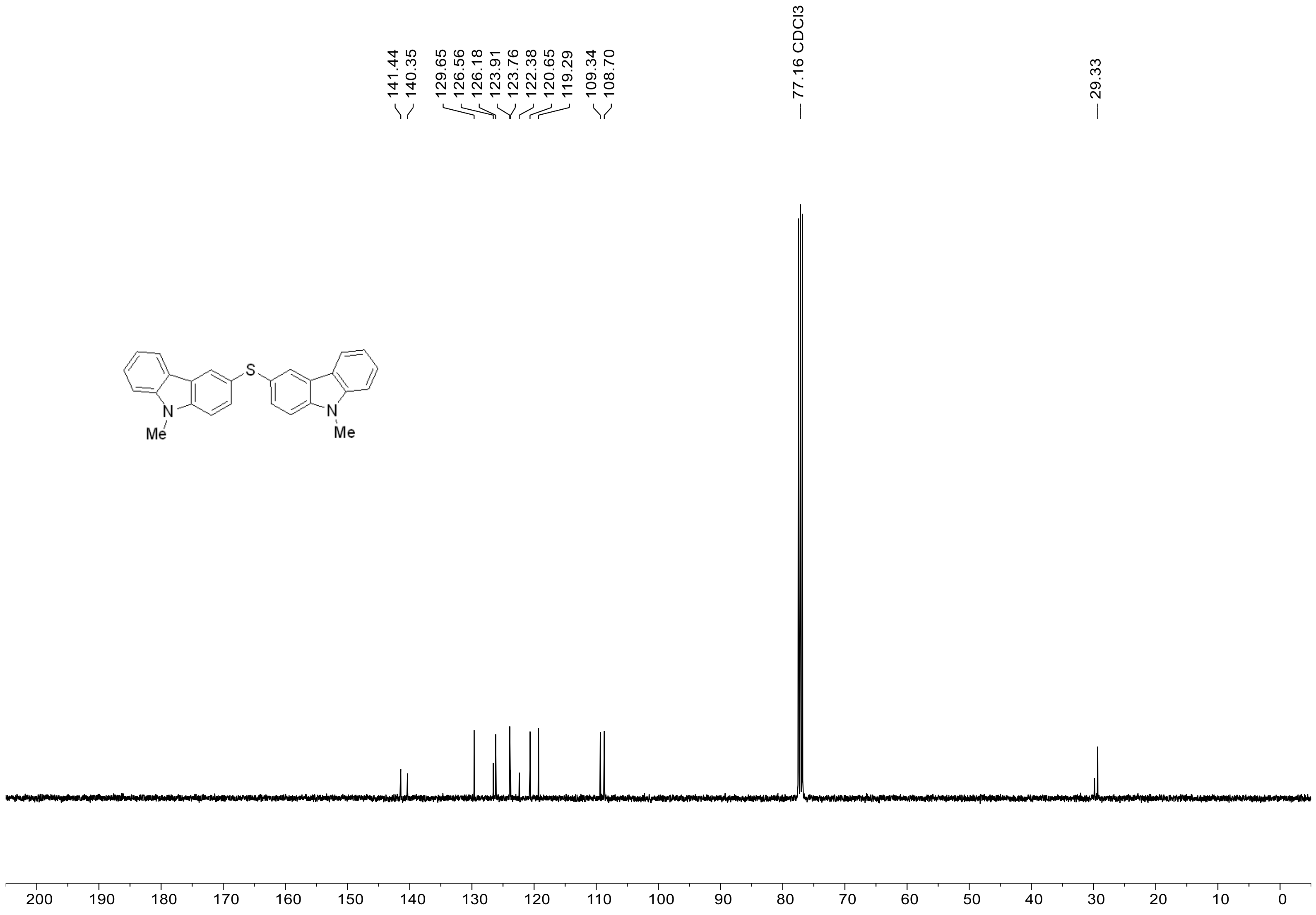

Figure S58: **^{1}H NMR** (400 MHz, $CDCl_3$, 298 K) spectrum of **3ax**.

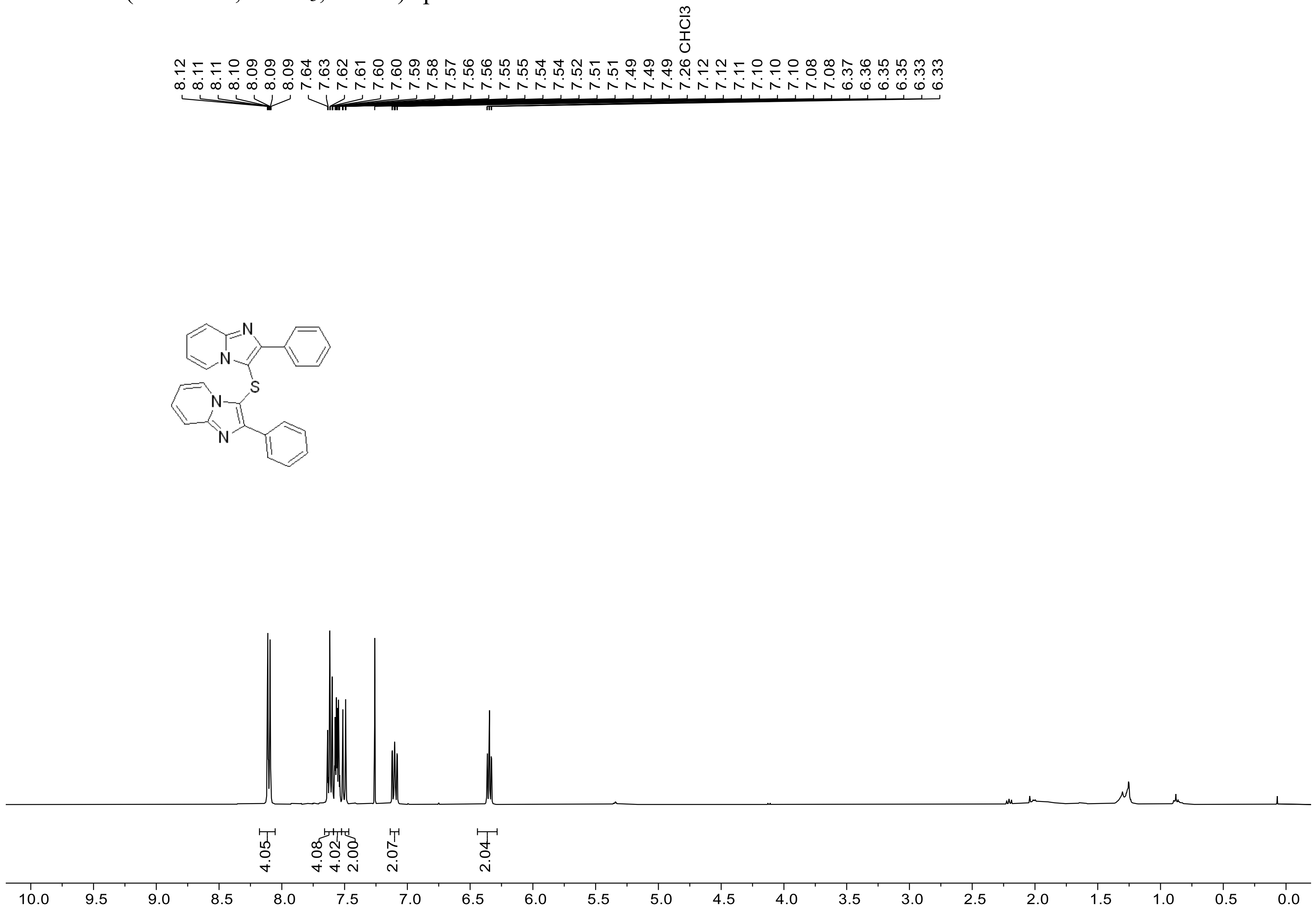

Figure S59: **^{13}C NMR** (101 MHz, $CDCl_3$, 298 K) spectrum of **3ax**.

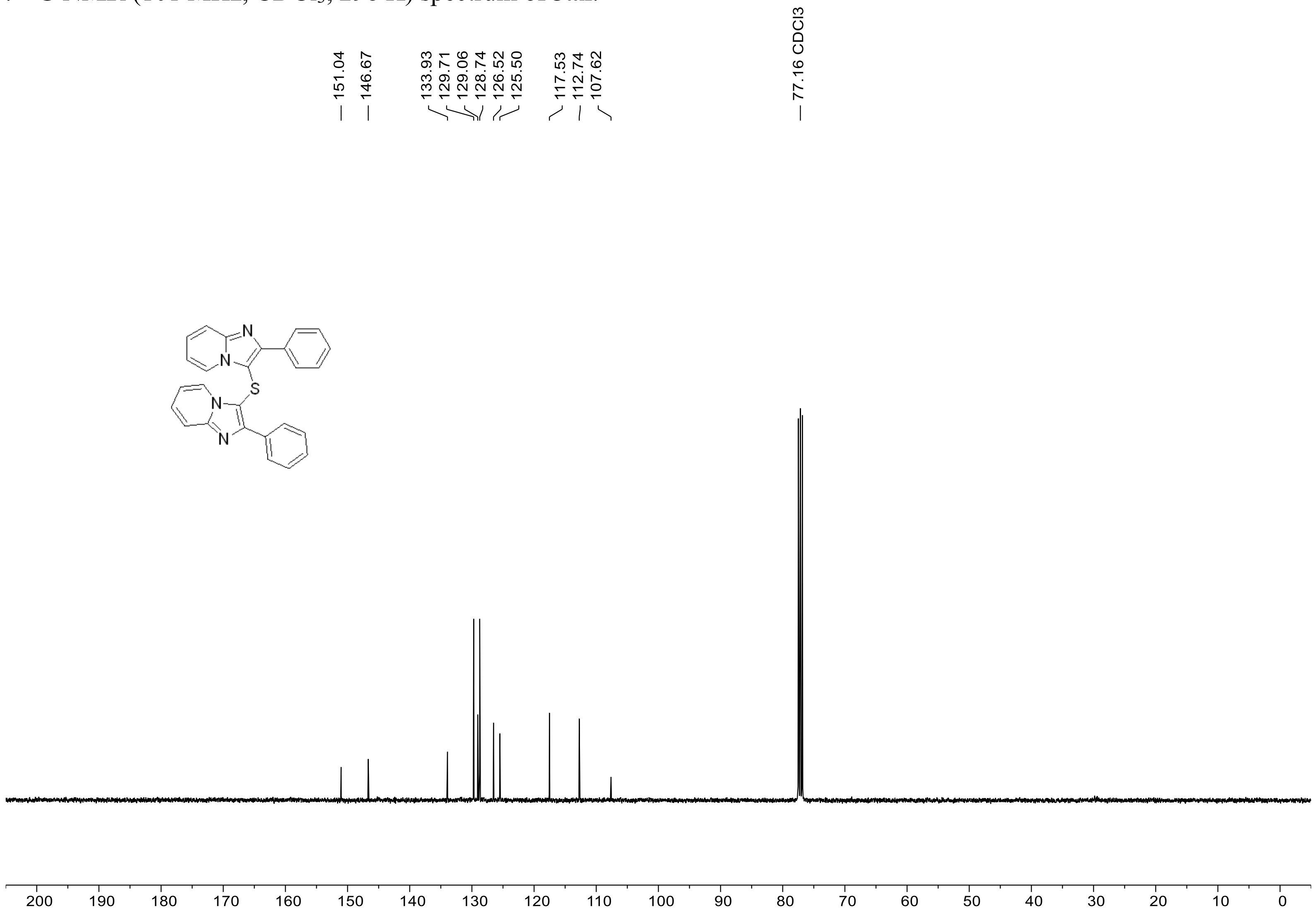

Figure S60: **^{1}H NMR** (400 MHz, $CDCl_3$, 298 K) spectrum of **4**.

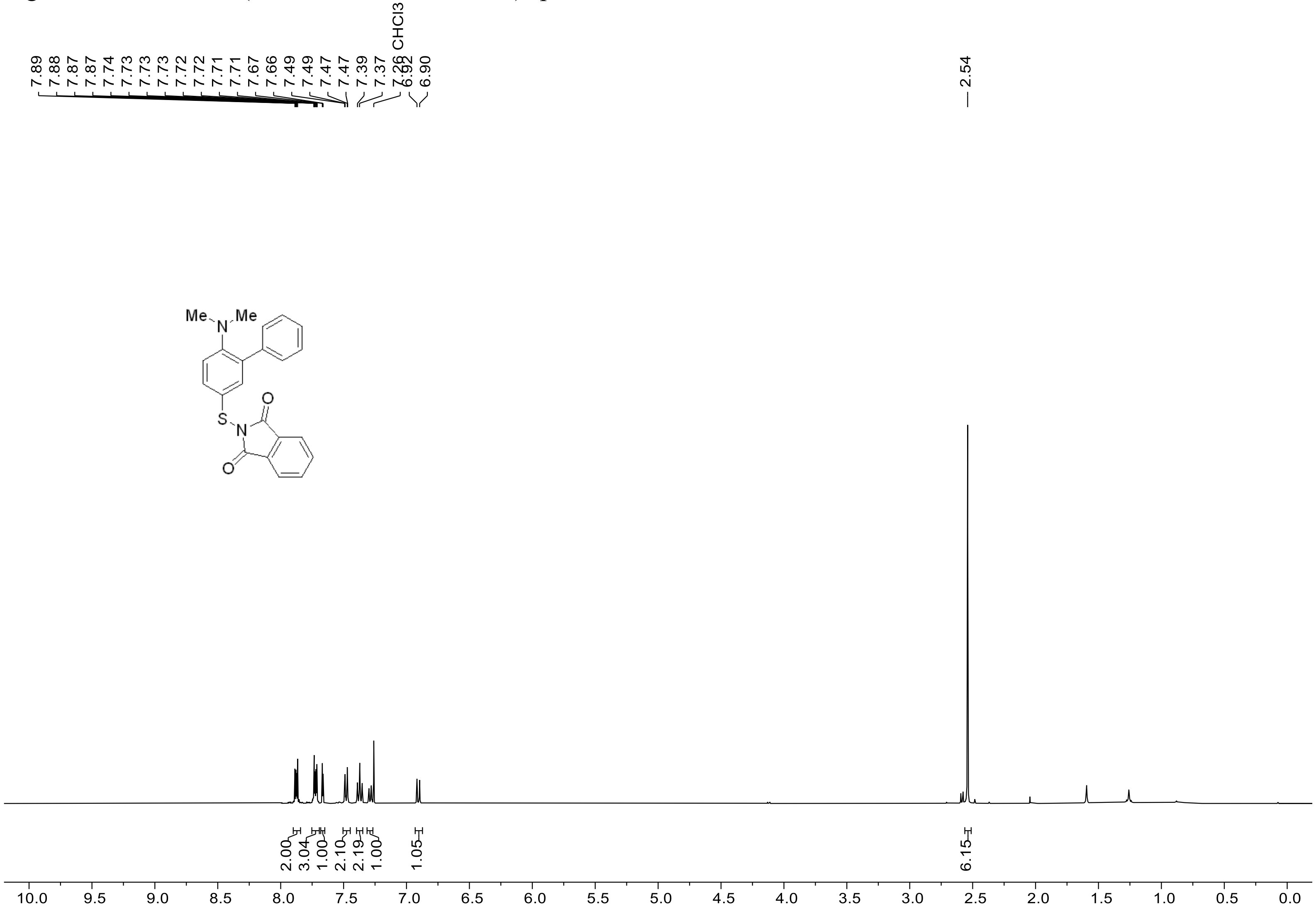

Figure S61: **^{13}C NMR** (101 MHz, $CDCl_3$, 298 K) spectrum of **4**.

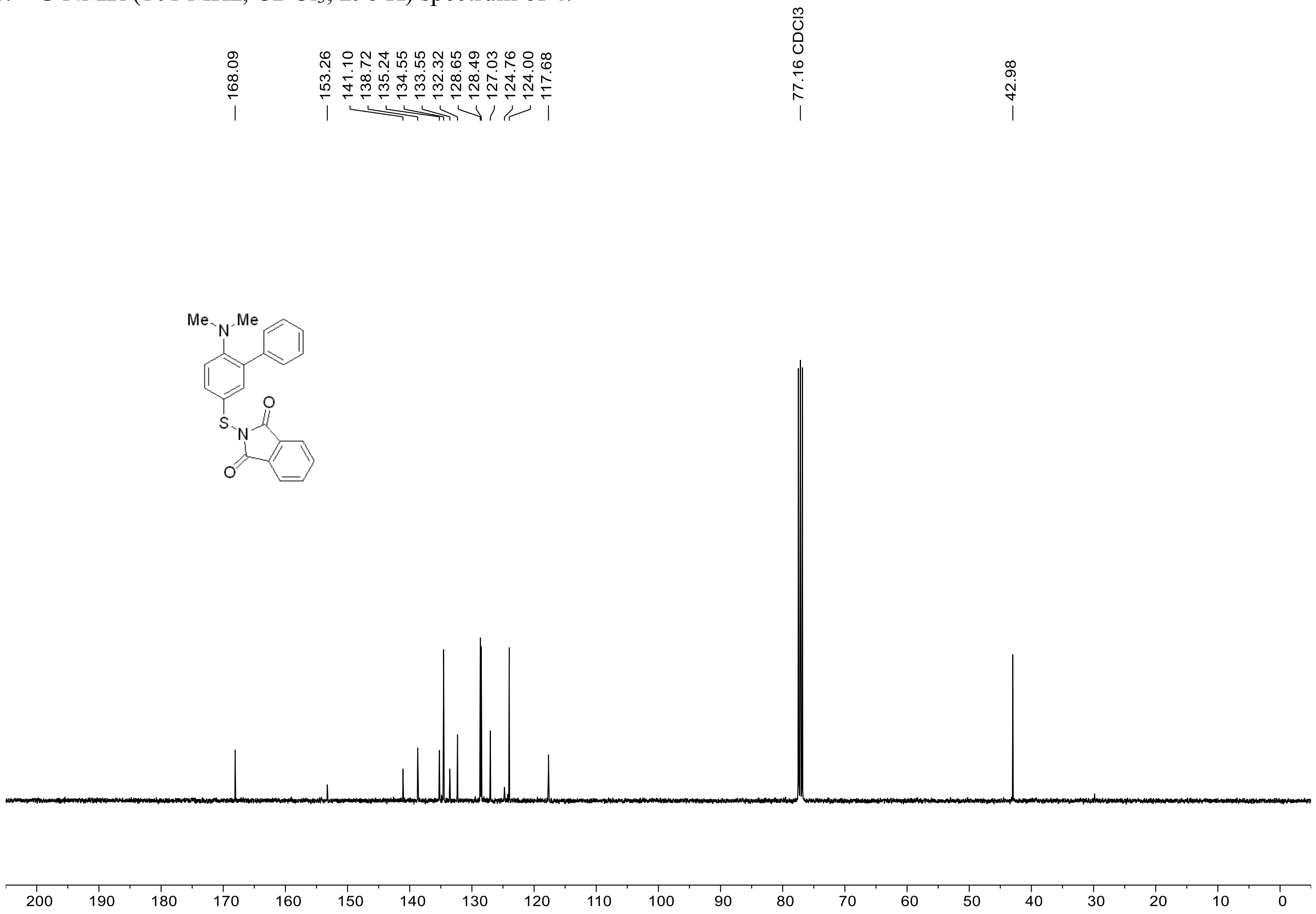

Figure S62: **^{1}H NMR** (400 MHz, $CDCl_3$, 298 K) spectrum of **6aa**.

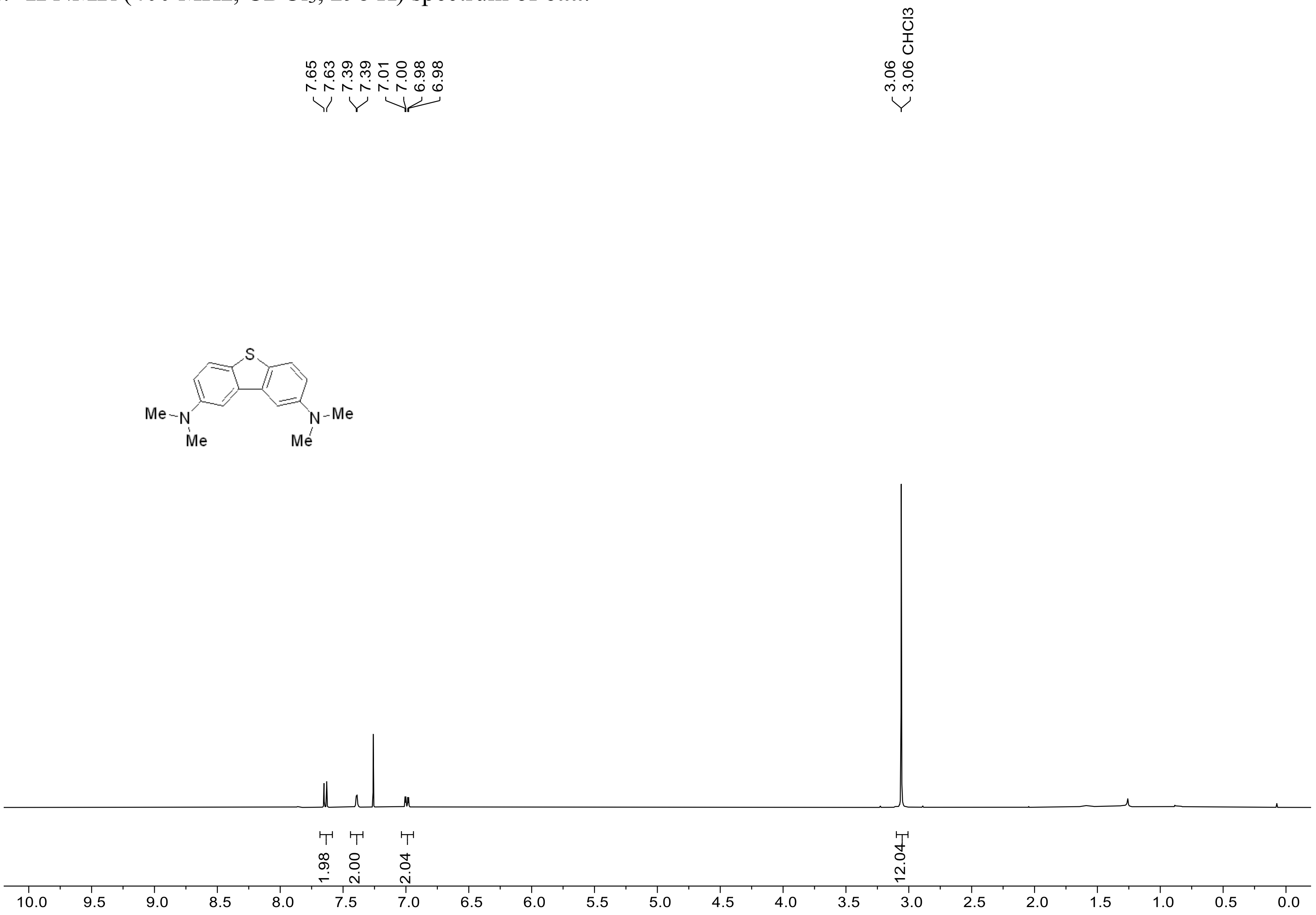

Figure S63: **^{13}C NMR** (101 MHz, $CDCl_3$, 298 K) spectrum of **6aa**.

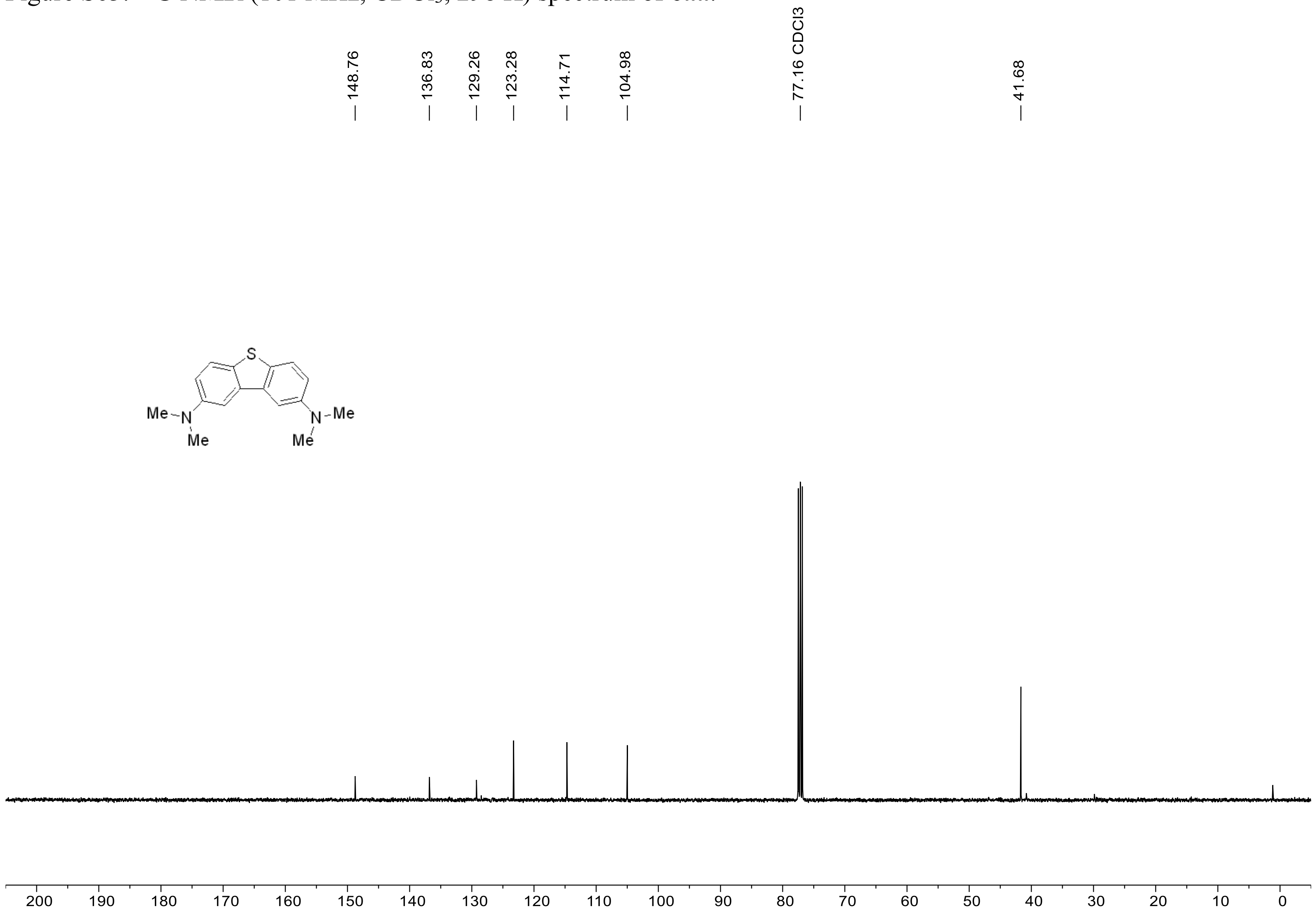

Figure S64: **^{1}H NMR** (400 MHz, $CDCl_3$, 298 K) spectrum of **3aa'**.

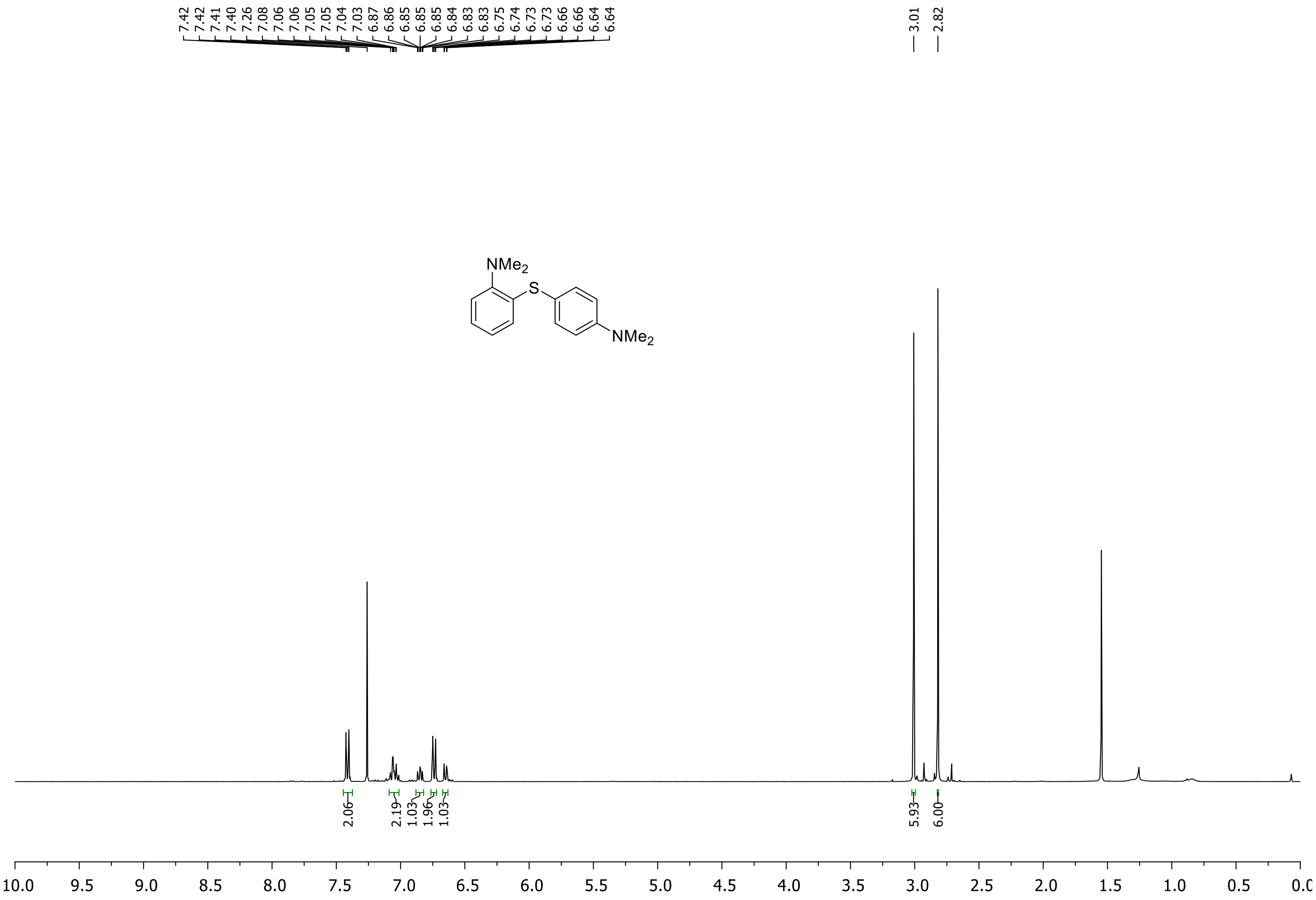

Figure S65: **^{13}C NMR** (101 MHz, $CDCl_3$, 298 K) spectrum of **3aa'**.

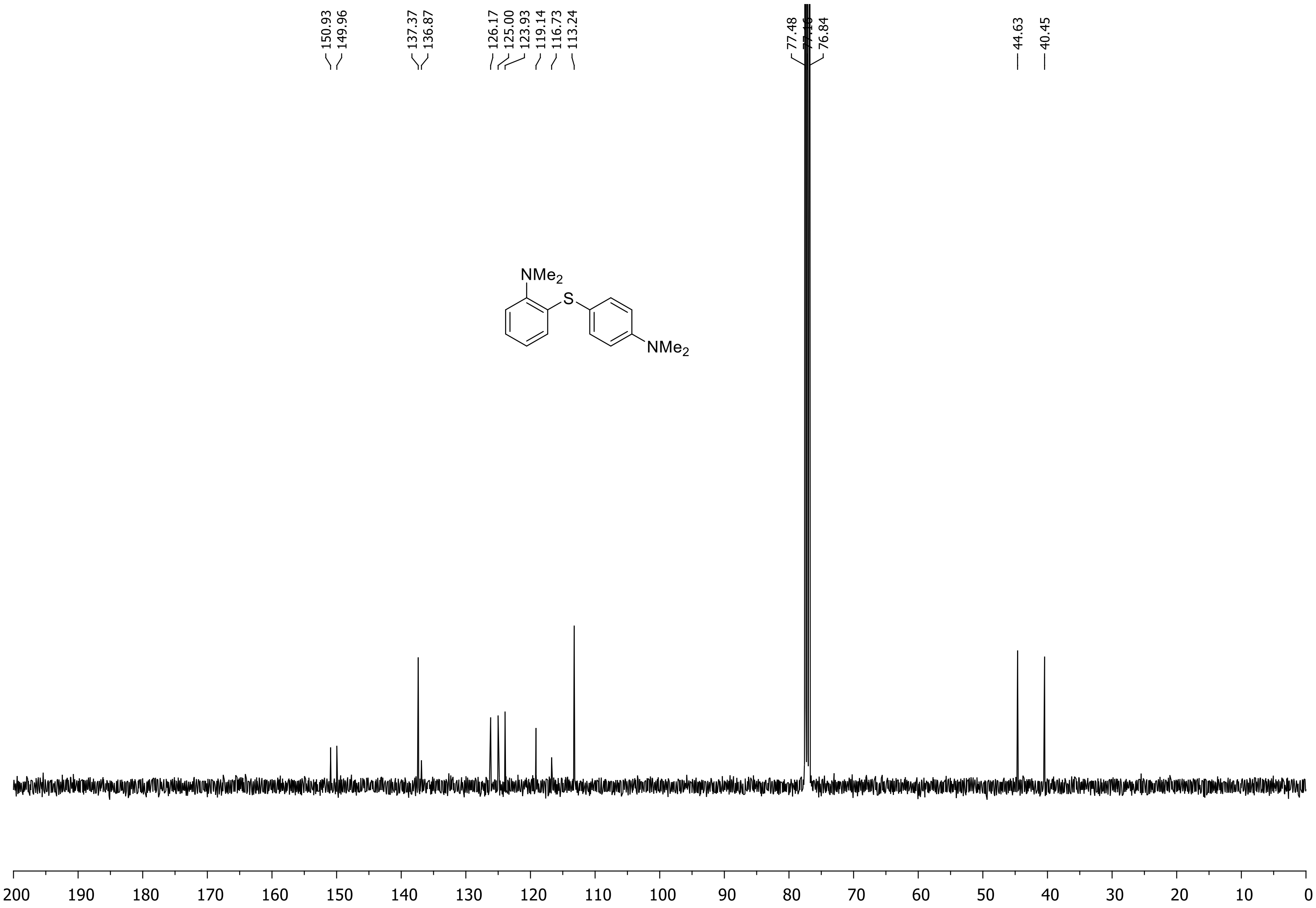

9. Kinetic Studies

Kinetic studies were carried out to confirm the rate law. The reaction was followed for 2 hours using ^{1}H NMR spectroscopy for solutions of 0.1 M **1a**, 20 mol% of $B(C_6F_5)_3$, and 0.12 M **2a** in $CDCl_3$ at 45 °C with 0.1 M $ClCH_2CH_2Cl$ as an internal standard. Concentrations of $B(C_6F_5)_3$ and **2a** were doubled to evaluate the reaction order. Doubling the concentration of **1a** was not possible because of the insufficient solubility of **1a**. Data were analysed using the Reaction Monitoring tool in MestreNova (version 16), with sufficiently well resolved peaks (indicated in Figure S66) integrated automatically over the time course of an experiment.

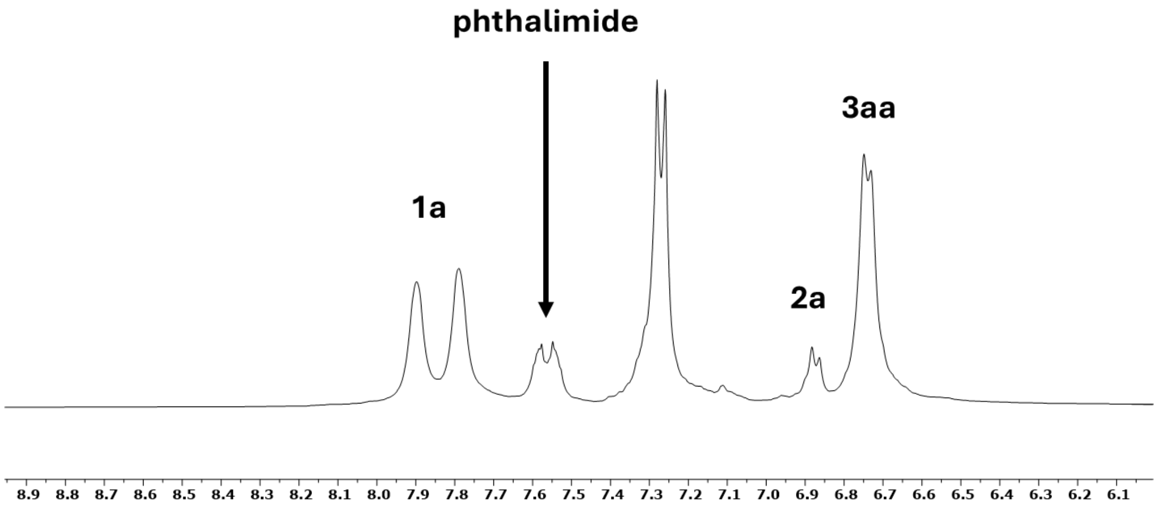

Figure S66: Example NMR spectrum showing the regions automatically integrated to obtain concentrations as a function of time.

The resulting data were plotted in Origin (OriginLab Corporation), as summarised in Figure S67. Figure S67 shows that in all reactions, the phthalimide by-product (•) started to precipitate after some time, resulting in unstable kinetic traces. Initial rate kinetics were obtained by plotting the concentrations of **1a**, **2a** and phthalimide as a function of time up to 1000 seconds and fitting a straight line to the resulting data (Table S2). Data for formation of **3aa** were excluded from initial rate analysis because the early timepoints show significant scatter because of the proximity of the signal for **3aa** to the signal for **2a**.

A	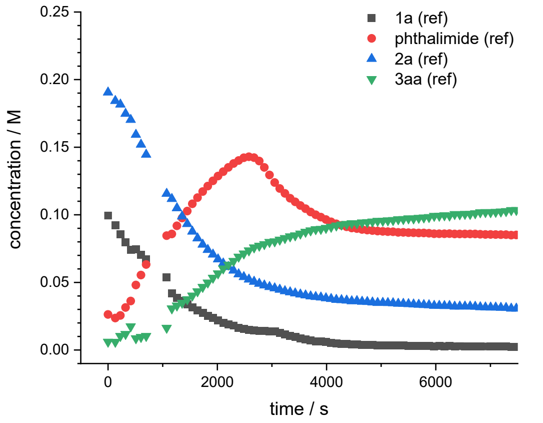
B	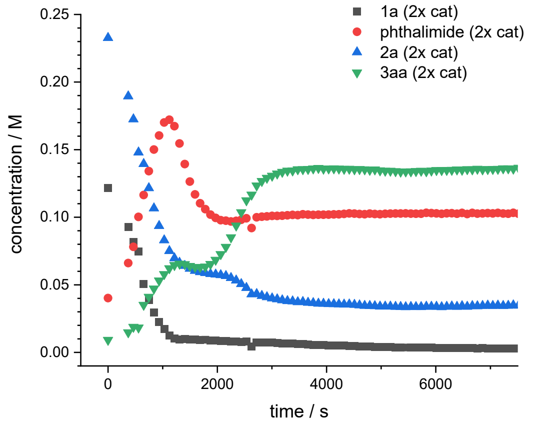
C	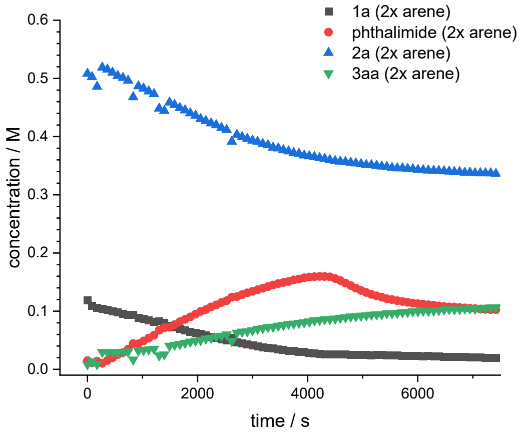

Figure 67: Concentrations of **1a**, phthalimide, **2a** and **3aa** as a function of time for 0.1 M **1a**, 20 mol% of $B(C_6F_5)_3$ and 0.12 M **2a** (panel A), 0.1 M **1a**, 40 mol% of $B(C_6F_5)_3$ and 0.12 M **2a** (panel B) and 0.1 M **1a**, 20 mol% of $B(C_6F_5)_3$ and 0.24 M **2a** (panel C), all in $CDCl_3$ at 45 °C with 0.1 M $ClCH_2CH_2Cl$ as an internal standard.

Table S2. Initial rates for the reaction[a]

	d[phthalimide]/d*t* (10^{-5} M s^{-1})	d[1a]/d*t* (10^{-5} M s^{-1})	d[2a]/d*t* (10^{-5} M s^{-1})	average rate[e] (10^{-5} M s^{-1})
reference[b]	8.2	-4.3	-7.5	4.1
2 × catalyst[c]	16.2	-10.3	-14.4	8.5
2 × **2a**[d]	4.8	-2.3	-3.1	2.1

a. in $CDCl_3$ at 45 °C with 0.1 M $ClCH_2CH_2Cl$ as an internal standard, data up to 1300 s included.
b. 0.1 M **1a**, 20 mol% of $B(C_6F_5)_3$ and 0.12 M **2a**
c. 0.1 M **1a**, 40 mol% of $B(C_6F_5)_3$ and 0.12 M **2a**
d. 0.1 M **1a**, 20 mol% of $B(C_6F_5)_3$ and 0.24 M **2a**
e. Rates normalized to reaction coefficient of 1.
